# Oxidation Under Reductive Conditions: From Benzylic Ethers to Acetals with Perfect Atom‐Economy by Titanocene(III) Catalysis

**DOI:** 10.1002/anie.202013561

**Published:** 2021-01-15

**Authors:** Pierre Funk, Ruben B. Richrath, Fabian Bohle, Stefan Grimme, Andreas Gansäuer

**Affiliations:** ^1^ Kekulé-Institut für Organische Chemie und Biochemie Universität Bonn Gerhard Domagk-Str. 1 53121 Bonn Germany; ^2^ Mulliken Center for Theoretical Chemistry Institut für Physikalische und Theoretische Chemie Universität Bonn Beringstraße 4 53115 Bonn Germany

**Keywords:** computational chemistry, oxidation, radicals, reaction mechanisms, titanium

## Abstract

Described here is a titanocene‐catalyzed reaction for the synthesis of acetals and hemiaminals from benzylic ethers and benzylic amines, respectively, with pendant epoxides. The reaction proceeds by catalysis in single‐electron steps. The oxidative addition comprises an epoxide opening. An H‐atom transfer, to generate a benzylic radical, serves as a radical translocation step, and an organometallic oxygen rebound as a reductive elimination. The reaction mechanism was studied by high‐level dispersion corrected hybrid functional DFT with implicit solvation. The low‐energy conformational space was searched by the efficient CREST program. The stereoselectivity was deduced from the lowest lying benzylic radical structures and their conformations are controlled by hyperconjugative interactions and steric interactions between the titanocene catalyst and the aryl groups of the substrate. An interesting mechanistic aspect is that the oxidation of the benzylic center occurs under reducing conditions.

## Graphical abstract

Oxidations with reducing agents? Fact and not fiction in a reaction featuring titanocene(III) catalysis, H‐atom transfer, and an organometallic oxygen rebound. Acetals and hemiaminals can be readily prepared from benzylic ethers and amines, respectively, under mild reaction conditions and in high yields. The structures of the radical intermediates were elucidated by the CREST program.

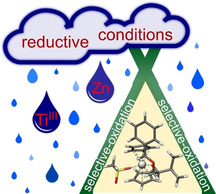

## Introduction

Catalysis in single electron steps^[1]^ or metallo‐radical catalysis^[2]^ is a concept that merges the concepts of radical chemistry with those of transition metal catalysis. In this manner, the advantages of both fields, such as the ease of radical generation, the high functional group tolerance of radical reactions and the ability of a metal complex to control the pertinent selectivities of transformation can be combined to attractive novel catalytic processes. An essential aspect for the success of catalysis in single electron steps is the availability of oxidative additions and reductions in single electron steps for radical and product generation.^[3]^ Both steps can be regarded as homolytic substitution reactions^[4]^ and require an efficient shuttling of the catalyst between neighboring oxidation states.^[5]^ During the radical translocation, a new bond and radical is formed. This step sets the scene for the ensuing reductive elimination.

## Results and Discussion

**Scheme 1:**
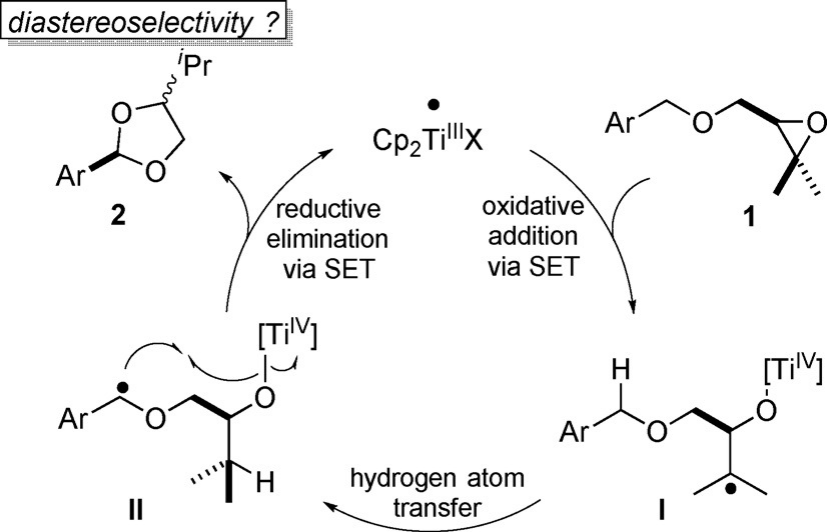
Atom‐economic transformation of benzylic ethers into acetals by titanocene(III) catalysis in single‐electron steps (SET).

Here, we demonstrate how radical translocation via H‐atom abstraction from a benzylic C−H bond by an alkyl radical can be included in a process proceeding via titanocene(III) catalysis in single electron steps (Scheme 1). The overall reaction constitutes an atom‐economical oxidation at the benzylic position that proceeds under reducing conditions. The oxidative addition occurs by a titanocene(III) mediated epoxide opening^[6]^ through electron transfer to yield **I**. From **I**, radical translocation occurs via the pivotal H‐atom abstraction^[7]^ to yield **II**. The driving force for this step arises from the increased strength of the newly formed C−H bond.^[8]^ In **II**, the stage is set for the reductive elimination of Cp_2_TiX that forms the desired product, acetal **2**. In this step that can be regarded as an organometallic oxygen rebound, a second stereocenter is formed.^[9]^ It remains to be seen how selective the ring formation is.

Our results for finding suitable reaction conditions for the reaction of **1 a** are summarized in Table 1. The active titanocene(III) catalysts were prepared by in situ reduction of the titanocene(IV) precatalysts with Zn. Without Zn, no reaction was observed. The use of Mn is possible but less convenient since the reduction of Ti^IV^ requires more time.[^6^, ^10^] In THF, Zn‐(CH_3_C_5_H_4_)_2_TiCl_2_ and Zn‐Cp_2_TiCl_2_ resulted in the consumption of **1 a**. However, hardly any **2 a** (2 % and 26 %, respectively) was formed as judged by quantitative ^1^H‐NMR analysis of the reaction mixture. While Zn‐Cp_2_Ti(O_2_CCF_3_)_2_ gave an improved conversion to **2 a**, the isolated yield was only 41 %. Zn‐Cp_2_Ti(O_3_SCH_3_)_2_ ^[10]^ (O_3_SCH_3_=OMs) provided the best catalyst and allowed the isolation of **2 a** in 67 % yield. Other solvents than THF lead to inferior results. The superiority of Zn‐Cp_2_Ti(O_3_SCH_3_)_2_ over Zn‐(CH_3_C_5_H_4_)_2_TiCl_2_ and Zn‐Cp_2_TiCl_2_ suggests that the reductive elimination is the rate determining step of the catalytic cycle because epoxide opening is faster with more electron rich catalysts whereas reductive eliminations are slowed down. In our case, the reductive elimination constitutes a homolytic substitution at a Ti−O bond by a benzylic radical. Due to the high stabilization of this radical, this step seems particularly difficult.^[4]^

**Table 1:** Screening of different catalysts and solvents to find the most suitable reaction conditions for the conversion of **1 a**. 
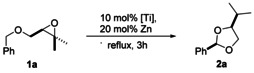

[Ti]	Solvent	Yield [%]	Conversion [%]^[a]^
(CH_3_C_5_H_4_)_2_TiCl_2_^[b]^	THF	2^[a]^	42
Cp_2_TiCl_2_^[b]^	THF	26^[a]^	75
Cp_2_Ti(O_2_CCF_3_)_2_	THF	41, 61^[a]^	91
Cp_2_Ti(O_3_SCH_3_)_2_	THF	67	100
Cp_2_Ti(O_3_SCH_3_)_2_	1,4‐dioxane	38^[a]^	96
Cp_2_Ti(O_3_SCH_3_)_2_	acetone	33^[a]^	41

[a] Yields and conversion are determined by quantitative ^1^H NMR spectroscopy. [b] Requires 50 mol % Collidine*HCl to prevent a decomposition of the catalyst.

**Scheme 2:**
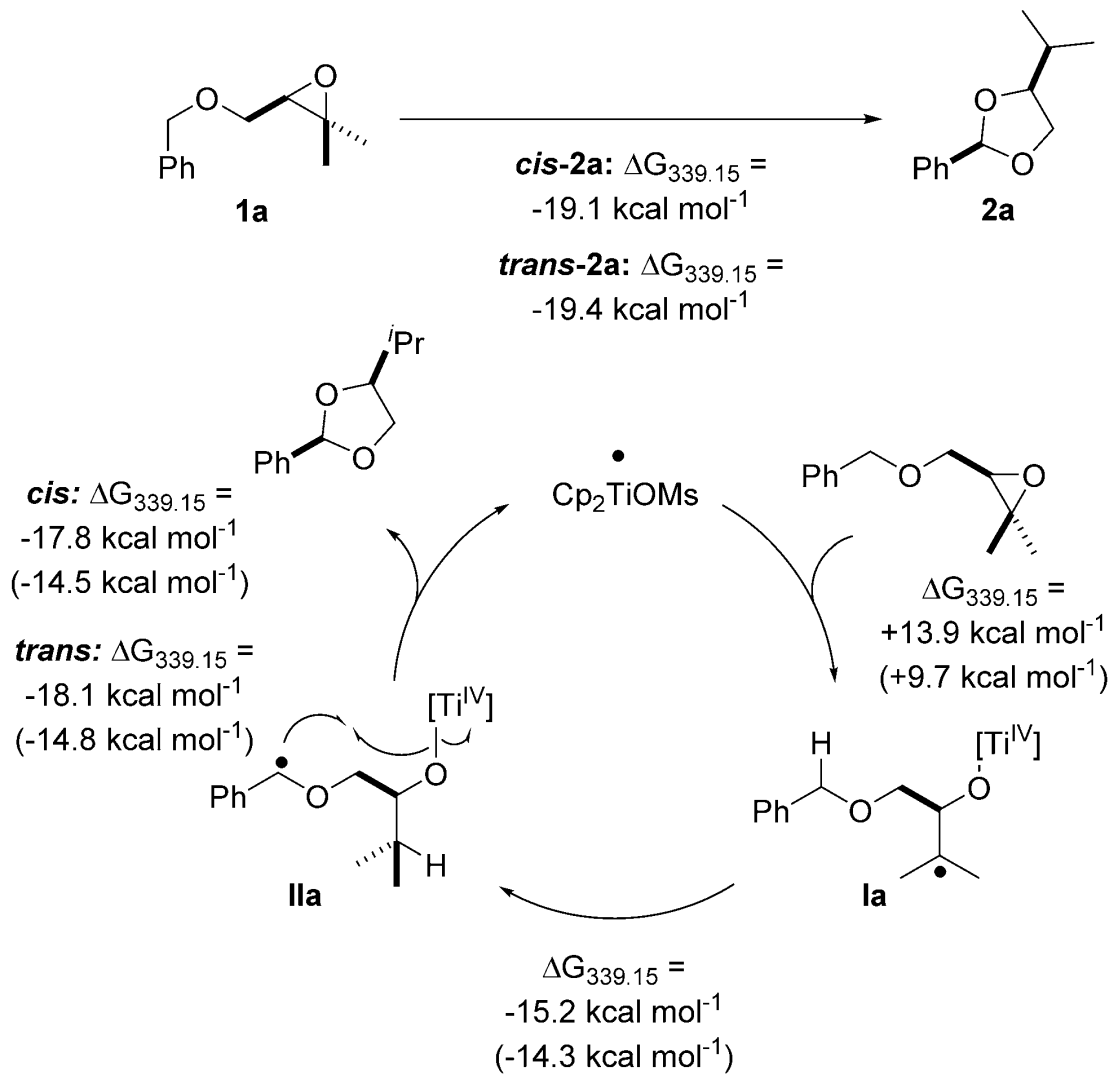
Atom‐economic transformation of benzylic ethers into acetals by titanocene(III) catalysis in single electron steps calculated at PW6B95‐D4/def2‐QZVP + COSMO‐RS(THF)// PBEh‐3c/DCOSMO‐RS(THF) level. Values within parentheses are calculated for the catalyst Cp_2_TiCl.

We employed Cp_2_Ti(O_3_SCH_3_)_2_ that is a stronger oxidant than Cp_2_TiCl_2_ due to the more electron withdrawing mesylate ligand.^[10d]^ In this manner, additional driving force for the reductive elimination is provided. The results summarized in Table 1 therefore demonstrate that the tuning of the redox‐properties of the titanocenes is a crucial and powerful tool to adjust the efficiency of the steps in the catalytic cycle.^[10]^ Remarkably, **2 a** is formed as a single diastereoisomer (structure determined by 2D‐NMR spectroscopy, see the Supporting Information).^[11]^ To understand this particular feature of the reaction and the thermodynamics of the catalytic cycle (Scheme 2), these were studied computationally by applying the following protocol.

All initial geometries were prepared manually and meta‐dynamics‐based conformer searches for all involved geometries were performed using the CREST^[12]^ code in combination with the generic force field GFN‐FF(GBSA[THF])^[13]^ with implicit solvation as implemented in the freely available xtb code.[^14d^, ^14e^, ^14f^] In case of metal containing structures, a small harmonic bond lengths constraint was applied during the minimum conformer search in order to avoid dissociation. The conformer ensemble was efficiently refined in multiple steps at DFT level PBEh‐3c/DCOSMO‐RS(THF)^[15]^ to obtain the lowest lying conformer, facilitated by the ENSO code.^[16]^ After identification of the lowest lying conformer, each of the structures, was used to calculate Gibbs free energies (G) to investigate the thermodynamics of the catalytic cycle shown in Scheme 2. Herein, PW6B95‐D4/def2‐QZVP^[17]^ dispersion corrected, high‐level single‐point energies and COSMO‐RS^[18]^ solvation contributions were calculated on the PBEh‐3c+DCOSMO‐RS(THF) optimized geometries. Thermostatistical contributions are obtained at the PBEh‐3c+COSMO(THF) level using the modified rigid rotor harmonic oscillator (mRRHO) approach.^[19]^ The mRRHO ansatz is applied to reduce the error of the harmonic approximation for low lying vibrational frequencies and to reduce the effect of numerical noise in the DFT. The vibrational frequencies are scaled by 0.95 and the thermostatistical analysis is evaluated at 339.15 K. Free energies are calculated as the sum of electronic energy, solvation‐ and thermostatistical terms. All DFT calculations were performed with TURBOMOLE program package 7.4.1.^[20]^ The resolution of the identity approximation for the Coulomb integrals is generally applied in combination with the default auxiliary basis sets.^[21]^ The numerical integration of the exchange correlation contribution is performed with grids m4 for PBEh‐3c and grid m5 for PW6B95. Default convergence criteria are applied for all single‐point calculations (Econv 10^−7^ E_h_). For geometry optimizations Econv 2×10^−5^ Eh and Gconv 2×10^−3^ E_h_ Bohr^−1^ are used. The convergence criteria for the transition‐state optimizations were set to 5×10^−6^ E_h_ and 2×10^−3^ E_h_ Bohr^−1^. The solvation contributions were calculated with the conductor like screening model for real solvents (COSMO‐RS) using the BP_TZVP_C30_1601 parametrization and the COSMOtherm2016 package.^[22]^ The solvation contribution incorporates the volume work RTln(V_ideal_) (*T*=339.15 K) for an ideal gas at 1 bar to 1 mol L^−1^. Geometries and tabulated data are provided in the SI.

The free energy Δ*G*_339.15_ of the reaction **1 a** to **2 a** (−19.1 kcal mol^−1^ for *cis*‐**2 a** and −19.4 kcal mol^−1^ for *trans*‐**2 a**) is strongly negative. This is a manifestation of the loss of the epoxide's ring strain.^[5]^ The very similar values of Δ*G*_339.15_ for *cis*‐**2 a** and *trans*‐**2 a** also show that the observed highly diastereoselective acetal formation cannot be under thermodynamic control.

The other important question of the mechanism is the understanding of the high diastereoselectivity of acetal formation in the reductive elimination. To this end, we studied the preferred conformation of the benzylic radical **IIa** and the structures and energies of the transition‐states leading to *cis*‐ and *trans*‐**2 a**.

The oxidative addition of titanocene catalyst Cp_2_TiOMs to the epoxide that leads to radical formation is the thermodynamically critical step (Δ*G*_339.15_=+13.9 kcal mol^−1^ for Cp_2_TiOMs and Δ*G*_339.15_=+9.7 kcal mol^−1^ for Cp_2_TiCl). The more favorable reaction with Cp_2_TiCl is in line with an earlier study on the thermodynamics of epoxide opening.^[1a]^ Experimental studies on the use of electron deficient catalysts have demonstrated that they lead to substantially lower rate constants (e.g. Cp_2_TiOMs: k_293_=0.013 M^−1^ s^−1^) compared to Cp_2_TiCl (k_293_=1.1 M^−1^ s^−1^).^[10d]^ The HAT step is strongly exergonic in line with the difference in stability of tertiary and benzylic radicals. Here the conformational search for the lowest lying conformer established that the HAT reaction from **Ia** to **IIa** is accompanied by a conformational relaxation. This structural relaxation results in an energy gain of 3.2 kcal mol^−1^ compared to a HAT where no conformational search has been employed (see SI for further information). The identification of a lower lying conformation affects all following reactions in the catalytic cycle.

Finally, the reductive elimination that results in product formation and catalyst regeneration is thermodynamically favorable for both Cp_2_TiOMs (***trans***: Δ*G*_339.15_=−18.1 kcal mol^−1^) and Cp_2_TiCl (***trans***: Δ*G*_339.15_=−14.8 kcal mol^−1^). The results suggest that increased efficiency of Cp_2_TiOMs over Cp_2_TiCl in the reductive elimination is the reason for the superiority of the former complex as catalyst.

**Scheme 3:**
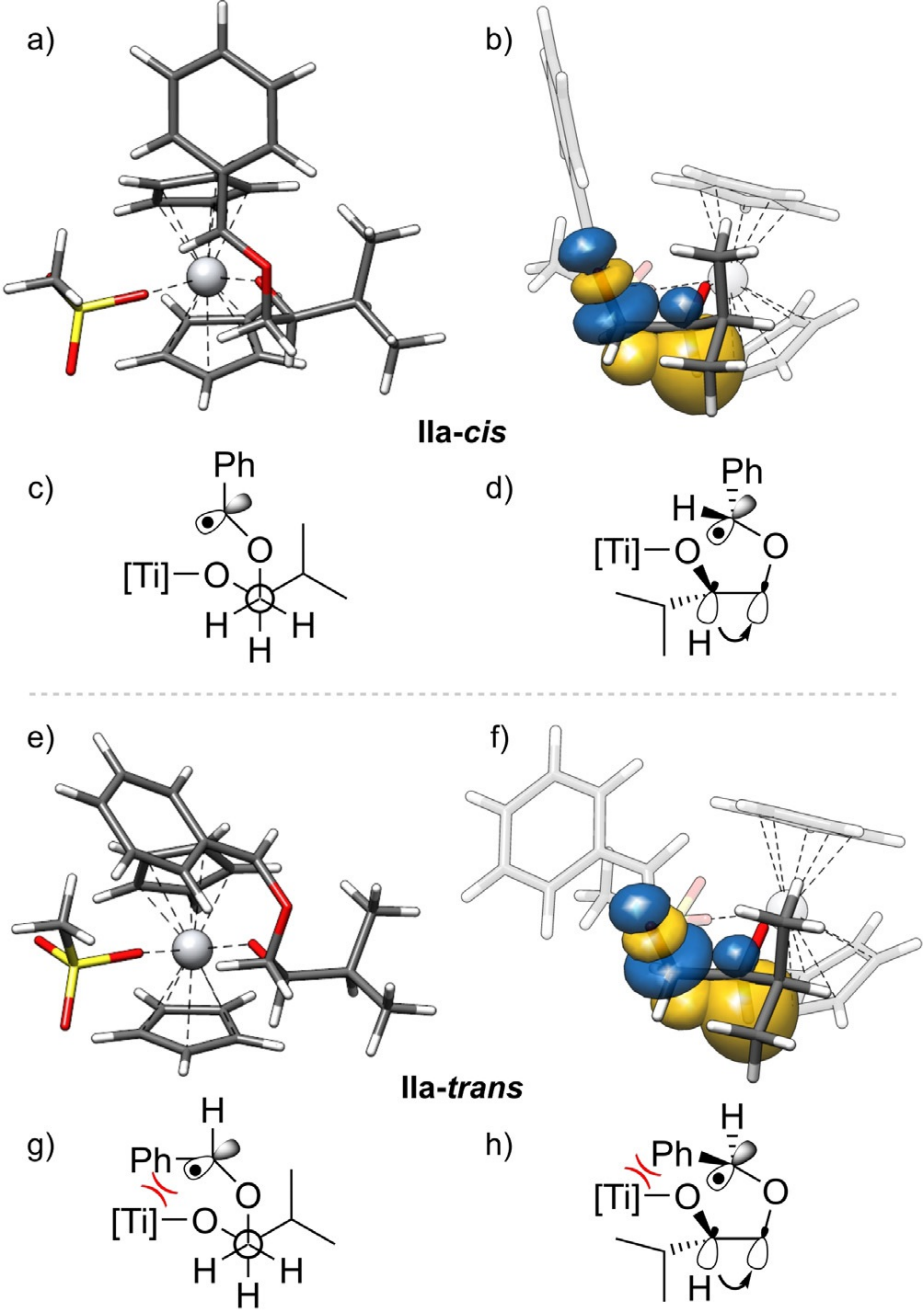
3D‐Structures of the radicals **IIa‐*cis*** (a,b) and **IIa‐*trans*** (e,f) and their stabilization by *gauche* interactions, with side view. The natural bond orbitals (NBO)^[23]^ shown are obtained from a PBEh‐3c+CPCM(THF) calculation. In addition Newman projections (c,g) and wedge and dash structures (d,h) of these radicals are given. They show the C−H bond orbital donating into the σ*‐orbital of the C−O bond, visualizing the gauche‐effect. Only one of two effective gauche interactions is shown. Isosurface value=0.05 e^−1/2^ bohr^−3/2^.

We found that **IIa‐*cis*** (leading to ***cis*** **‐2 a**, Scheme 3) is the most stable conformer of **IIa**. We attribute this stability to two hyperconjugative interactions between the σ‐orbitals of C−H bonds and σ*‐orbitals of C−O bonds (the ***C−O***−Ti bond and the ***C−O***−C^•^ bond). This results in the preferred *gauche*‐orientation of the two C−O bonds. Additionally, the “upper” Cp‐ligand and the ‐OMs ligand of the catalyst forces the phenyl ring in a position pointing away from “upper” Cp‐ligand and the H‐substituent of the benzylic radical towards the ‐OMs ligand. Natural bond orbitals (see Scheme 3) and second‐order perturbative estimates of donor‐acceptor interactions in the NBO basis were calculated using the PBEh‐3c/CPCM(THF) density obtained with the ORCA 4.2.1 program package^[24]^ and the NBO6 code.^[23]^ The second conformer **IIa‐*trans*** (leading to *trans*‐**2 a**, Scheme 3) that we were able to identify is 6.5 kcal mol^−1^ less favored than **IIa‐*cis*** at 339.15 K. It arises from **IIa‐*cis*** by a rotation around the O−C^•^ bond. The stabilization by the *gauche*‐interactions in **IIa‐*cis*** and **IIa‐*trans*** is essentially identical and, therefore, the difference in stability is due to increased repulsive steric interactions between the Ph‐group and the “upper” Cp‐ligand and the ‐OMs‐ligand. The barrier for the rotation that transforms **IIa‐*cis*** into **IIa‐*trans*** is estimated to be 7.1 kcal mol^−1^ (see SI).

From **IIa‐*cis*** and **IIa‐*trans*** we calculated the barriers for the transformation to *cis*‐**2 a** (16.7 kcal mol^−1^) and *trans*‐**2 a** (18.3 kcal mol^−1^), respectively.

**Scheme 4:**
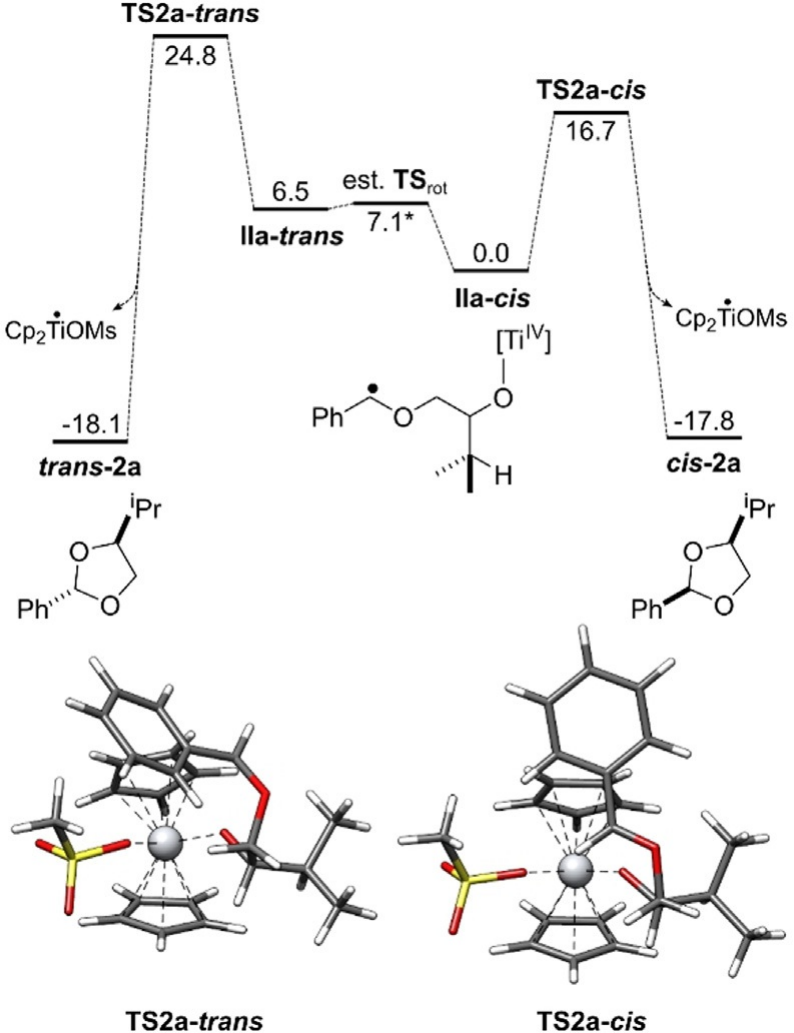
Energy profile of the conversion of **IIa‐*cis*** and **IIa‐*trans*** to *cis*‐**2 a** and *trans*‐**2 a**. All Gibbs free energies are shown in kcal mol^−1^. The transition‐state geometries were obtained with PBEh‐3c/COSMO(THF). The catalytic cycle has been investigated at PW6B95‐D4/def2‐QZVP + COSMO‐RS(THF)// PBEh‐3c/ COSMO(THF) level of theory.

The initial reaction paths were calculated with GSM/GFN2‐xTB(GBSA[THF])^[14]^ and subsequently refined at PBEh‐3c/COSMO(THF) level. The final transition‐state is optimized and verified for having only one imaginary mode. Free energies are calculated using the multilevel approach discussed before (PW6B95‐D4/def2‐QZVP + COSMO‐RS(THF)// PBEh‐3c/ COSMO(THF)). The energy profile of the conversion of the radicals **IIa** to products **2 a** is shown in Scheme 4. The selectivity of acetal formation is mainly due to the difference in stability of **IIa‐*cis*** and **IIa‐*trans***. Both transition‐states are “early” with long distances (**TS2 a**‐***trans***=285 pm, **TS2 a**‐***cis***=242 pm vs. 139 and 140 pm in *trans*‐**2 a** and *cis*‐**2 a**) of the forming C−O bonds and are, therefore, structurally similar to **IIa‐*cis*** and **IIa‐*trans***. Thus, the conformations of radicals **IIa‐*cis*** and **IIa‐*trans*** and also those of the transition‐states **TS2 a**‐***cis*** and **TS2 a**‐***trans*** are controlled by the same two factors. First, the dihedral angles in the **O**‐**C**H*i*Pr‐**C**H_2_‐**O** chain are determined by hyperconjugative interactions between the σ‐orbitals of C−H bonds and σ*‐orbitals of C−O bonds (*gauche*‐interactions). Second, the conformation at the benzylic radical center is determined by the steric interactions of the Ph‐ring and the H‐substituent with the “upper” Cp‐ligand and the OMs‐ligand.

This analysis suggests that acetals derived from benzylic ethers (Ar***CH***_***2***_O‐groups) should be formed with high diastereoselectivity. This is indeed the case as summarized in Table 2. Diastereoselectivities of >99:<1 imply that the other isomer could not be detected by ^1^H‐NMR spectroscopy.

**Table 2:** Substrate scope of the 1,3‐dioxolane synthesis by catalysis in single‐electron steps.

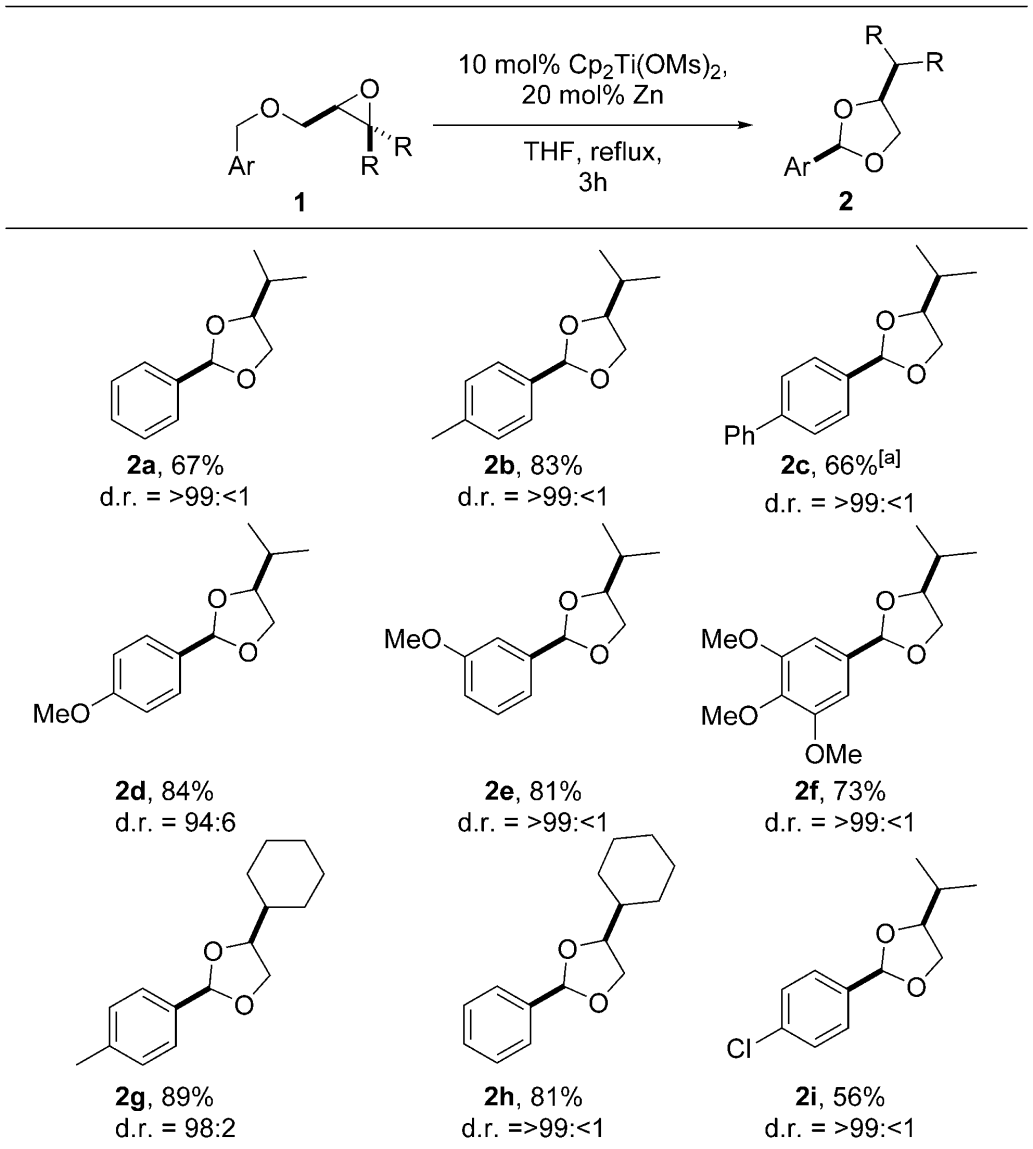

Yields are those of the isolated products. The d.r. values were determined by ^1^H NMR spectroscopy. [a] 19 mol % Cp_2_Ti(OMs)_2_ and 38 mol % Zn are used.

The *cis*‐2,4‐disubstituted 1,3‐dioxolanes **2 a**–**2 i** were smoothly obtained from the substituted benzyl ethers **1 a**–**1 i** in reasonable to high yield. Substituents at the *m*‐ and *p*‐positions are readily tolerated. Diastereoselectivity is high (94:6–99:1) for 4‐*i*propyl substituted dioxolanes. With a cyclohexyl substituent, the diastereoselectivity is equally high. For the details of the determination of relative configuration by NOESY‐spectroscopy and the determination of the diastereomeric ratios by ^1^H‐NMR‐spectroscopy see the SI.

**Scheme 5:**
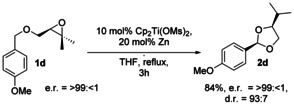
Preparation of enantiomerically pure **2 d** from **1 d**.

Our method can also be employed to prepare *cis*‐2,4‐disubstituted 1,3‐dioxolanes in enantiomerically pure form (Scheme 5). This experiment proves that no racemization is occurring and provides further support for the proposed mechanism (Scheme 2).

The transition‐state models for the formation of **2 a** provide an explanation for the experimentally observed diastereoselectivity with benzyl ethers. Our mechanistic analysis suggests that replacing one‐H atom at the benzylic center in **1** with bulkier alkyl groups should have a profound impact on the reaction. This is indeed the case as exemplified in the synthesis of ketals **4 a**–**4 e** (Table 3). In all cases, diastereoselectivity was either non‐existent or low (50:50–72:38). We note that when applicable the major diastereomer has a *trans*‐orientation of the aryl and the *i*Pr group. For details of the structural assignment, see SI. While the experiments were typically performed with 100 mg of the substrate, the reaction of **4 e** was performed on a 1 g scale indicating that a larger scale is not problematic.

**Table 3:** Substrate scope and diastereoselectivity of the 2,2‐disubstituted 1,3‐dioxolane synthesis by catalysis in single‐electron steps.

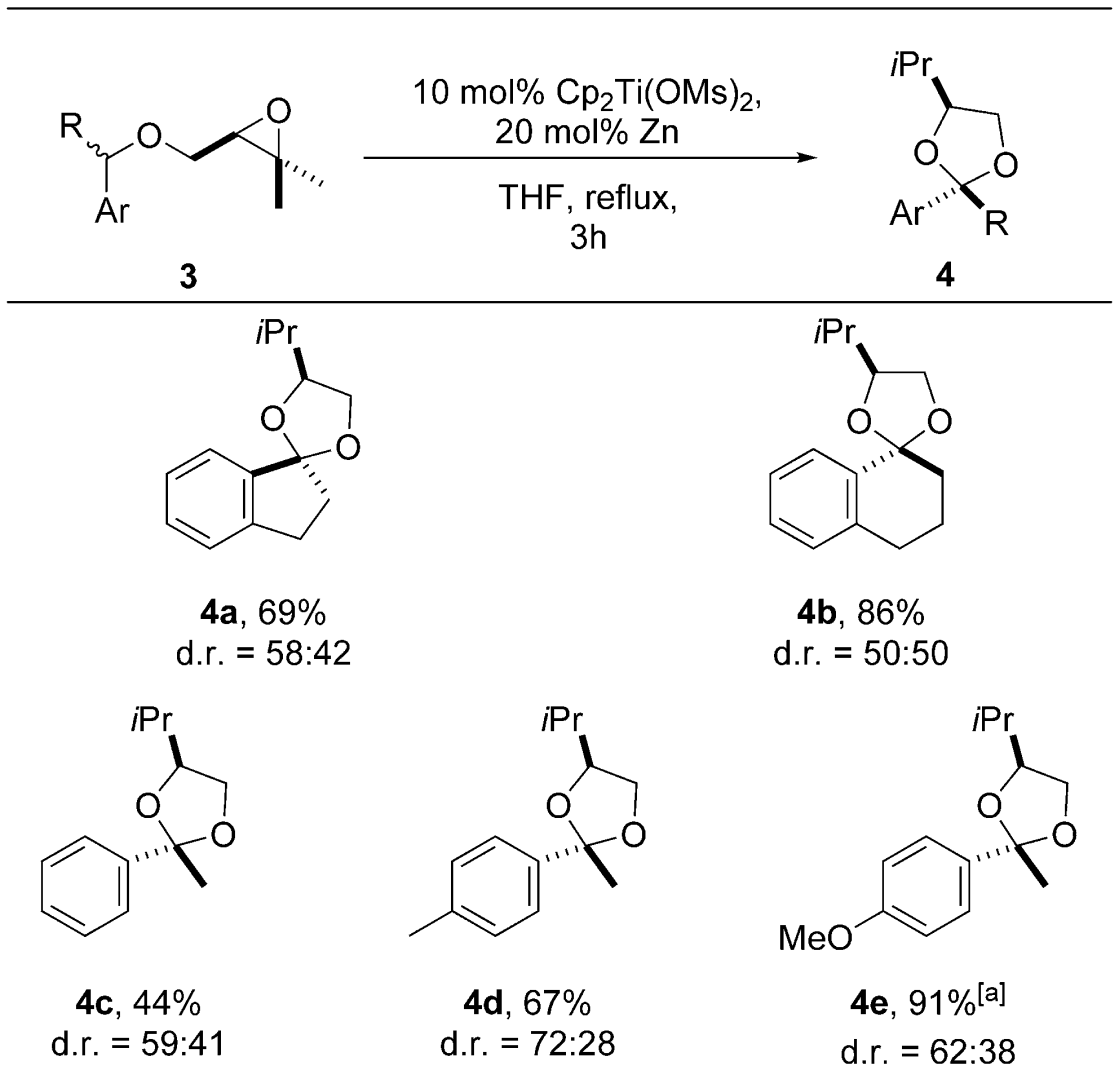

Yields are those of the isolated products. The d.r. values were determined by ^1^H NMR spectroscopy. [a] The experiment is performed on a 1 g scale.

**Scheme 6:**
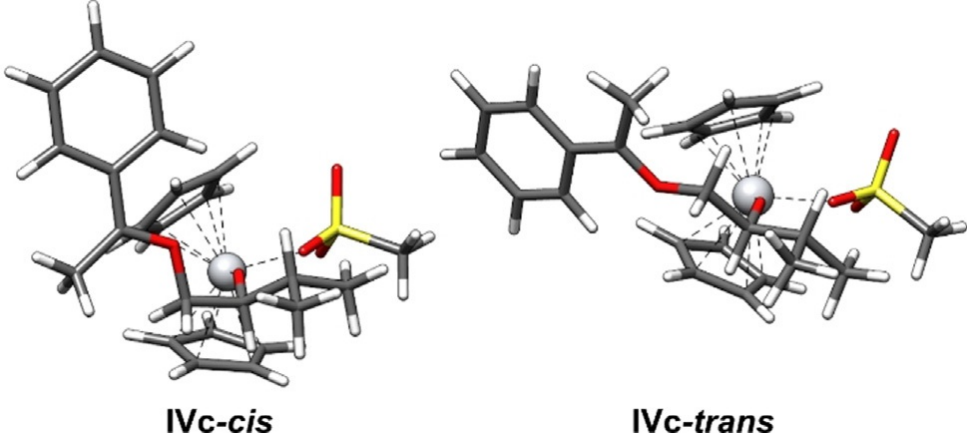
PBEh‐3c/DCOSMO‐RS(THF) structures of the radicals **IVc‐*cis*** and **IVc‐*trans***.

The computed structures and energies of radicals **IVc‐*cis*** and **IVc‐*trans*** highlight the reasons for the selectivity (Scheme 6). In contrast to the situation for radical **II** (Δ*G*_339.15_=6.5 kcal mol^−1^), the difference in the stability between the two conformers is low (Δ*G*_339.15_=−0.6 kcal mol^−1^) and **IVc‐*trans*** is more stable than **IVc‐*cis***. The small difference of −0.6 kcal mol^−1^ should therefore lead to a slight excess of *trans*‐**4 c** compared to *cis*‐**4 c**, which indeed is obtained for synthesis of ketals **4 a**–**4 e** (Table 3).

Finally, we investigated the synthesis of hemiaminals from suitably substituted benzylamines (Table 4). In this manner, it can be established if amines are tolerated under our conditions and how this change in the substitution pattern affects selectivity. Moreover, our method provides a convenient route to hemiaminals and eventually carbonyl compounds in one step from benzyl amines.

**Table 4:** Substrate scope and diastereoselectivity of the hemiaminal synthesis by catalysis in single‐electron steps.

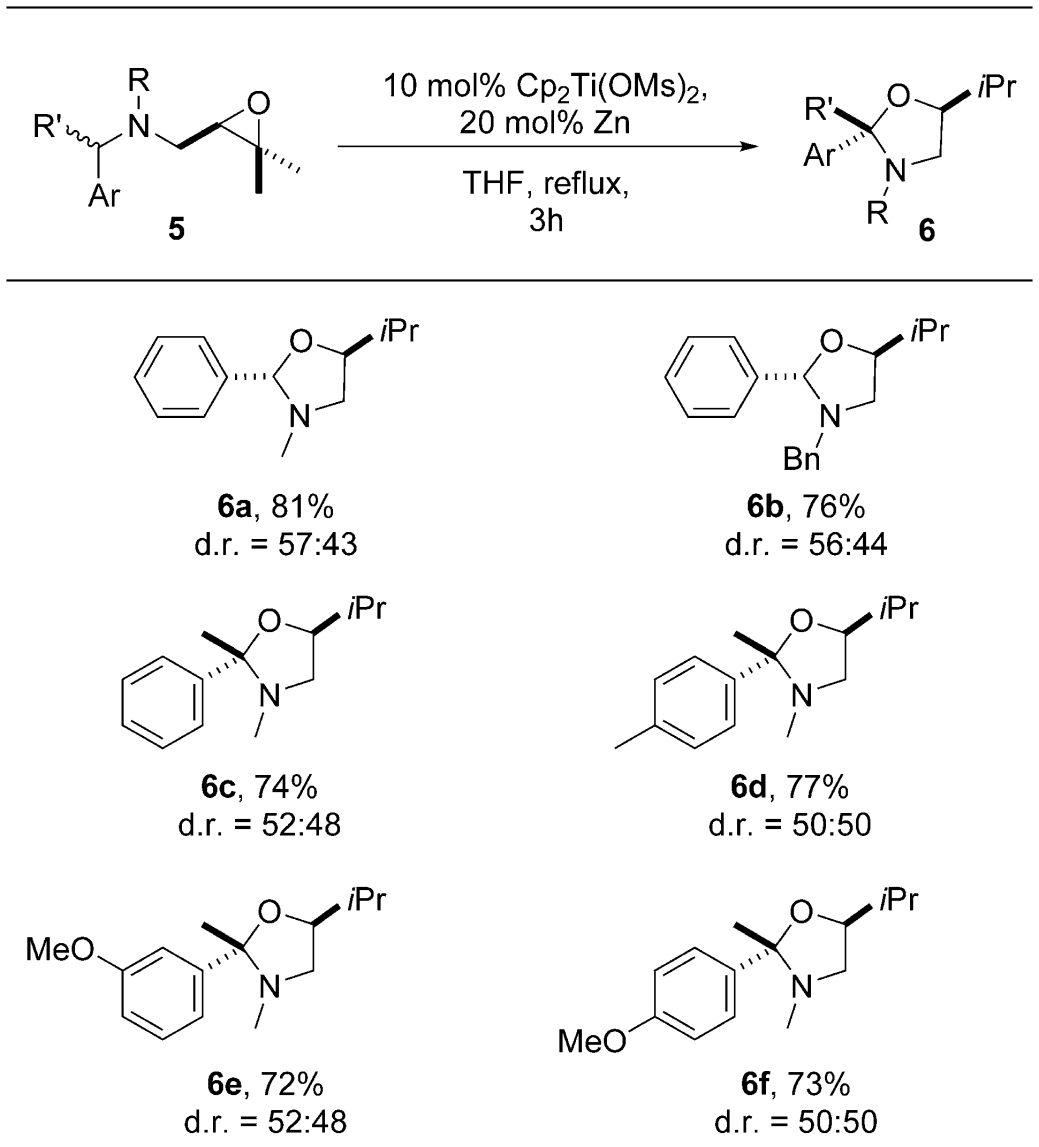

Yields are those of the isolated products. The d.r. values were determined by ^1^H NMR spectroscopy.

A practical difficulty associated with the hemiaminals obtained is their lability towards chromatography on SiO_2_. The aldehyde derived products **6 a** and **6 b** could be isolated in good yield. We found that for the synthesis of the ketone derived hemiaminals **6 c–6 f** dissolving the crude product in diethylether followed by a filtration through a plug of cotton wool was sufficient to isolate the desired products.

**Scheme 7:**
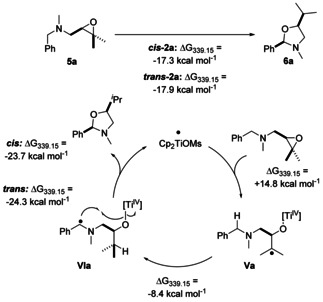
Atom‐economic transformation of benzylamines into hemiaminals by titanocene(III) catalysis in single electron steps. Calculated at PW6B95‐D4/def2‐QZVP + COSMO‐RS(THF)// PBEh‐3c/DCOSMO‐RS(THF) level of theory.

The thermodynamics of hemiaminal synthesis and of each step of the catalytic cycle (Scheme 7) show that hemiaminal formation is slightly less favorable than acetal formation. With the oxidative addition being most likely rate‐limiting, the calculations suggest that in agreement with the experiment the overall reaction should proceed without problems.

Radical translocation by H‐atom abstraction is distinctly less attractive (about 8 kcal mol^−1^) than in acetal formation. It seems that this is not due to differences in the BDE at the benzylic C−H bonds since the calculated BDE in benzylmethylether is only 0.2–0.9 kcal mol^−1^ lower than for benzyldimethylamine for 6 different theoretical methods (see SI for details). Additionally, the conformational investigation established that for the HAT from **Va** to **VIa** only a small structural relaxation occurs, which further explains the less attractive value for the HAT reaction compared to the acetal formation (see SI). The reductive heterocycle formation, is substantially more advantageous than for acetals.

Radical **VIa‐*trans*** is slightly more stable than radical **VIa‐*cis*** (0.9 kcal mol^−1^) and thus a low diastereoselectivity is predicted in agreement with the experiment. This is in line with our analysis of ketal formation. Replacing oxygen with the bulkier alkylated nitrogen results in similar steric interactions with the titanocene as in radicals **IV**.

**Scheme 8:**
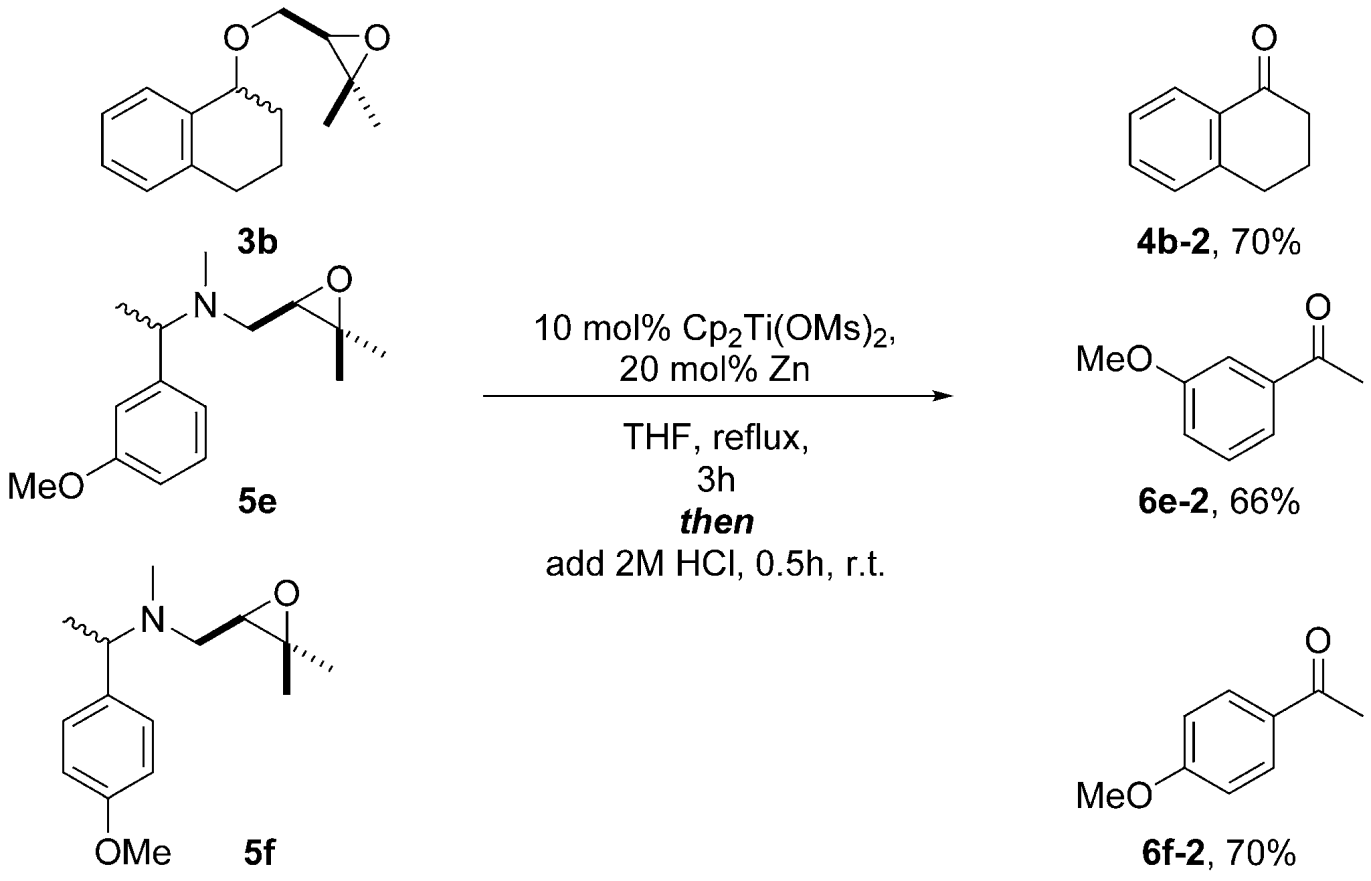
Acetal and hemiaminal synthesis with aqueous acidic work up for the synthesis of carbonyl compounds from benzylic ethers and amines.

An attractive feature of our method is that from the benzylamines substrates, the corresponding ketones can be prepared when an acidic aqueous work‐up is carried out with the crude product (Scheme 8).

## Conclusion

In summary, we have developed a novel titanocene catalyzed method for the synthesis of acetals and hemiaminals from benzylic ethers and benzylic amines with pending epoxides. The reaction features catalysis in single electron steps, an epoxide opening as oxidative addition, a H‐atom transfer to generate a benzylic radical as radical translocation step, and an organometallic oxygen rebound as reductive elimination. The thermochemistry of the reactions was studied at PW6B95‐D4/def2‐QZVP + COSMO‐RS(THF)// PBEh‐3c/ DCOSMO‐RS(THF) level of theory including full conformational search for all stationary points. Theory and experiment are in good qualitative agreement. The automatic exploration of conformational space by the CREST program was essential for identifying the lowest lying conformations and we recommend our computational approach in general for such mechanistic studies. The stereoselectivity can be deduced from the structure of the lowest lying benzylic radicals. Their conformations are controlled by hyperconjugative interactions and by steric interactions between the catalyst and the aryl groups. A particularly interesting aspect of our mechanism is that the oxidation of the benzylic center occurs under reducing conditions. No external oxidizing agent is required, making it a promising method for highly selective oxidations in natural product and API synthesis. Moreover, the corresponding carbonyl compounds can be directly obtained by an acidic aqueous work‐up of the reaction mixture.

## Conflict of interest

The authors declare no conflict of interest.

## Supporting information

As a service to our authors and readers, this journal provides supporting information supplied by the authors. Such materials are peer reviewed and may be re‐organized for online delivery, but are not copy‐edited or typeset. Technical support issues arising from supporting information (other than missing files) should be addressed to the authors.

Supplementary
