# Supplementary material for: Oxidation Under Reductive Conditions: From Benzylic Ethers to Acetals with Perfect Atom‐Economy by Titanocene(III) Catalysis
Source: Angew Chem Int Ed Engl. 2021 Jan 15;60(10):5482–8. doi: 10.1002/anie.202013561 (PMC7986230; doi:10.1002/anie.202013561)
Supplement: Supplementary file 1 — Supplementary [file ANIE-60-5482-s001.pdf]

Angewandte Chemie
Eine Zeitschrift der Gesellschaft Deutscher Chemiker

Supporting Information

# Oxidation Under Reductive Conditions: From Benzylic Ethers to Acetals with Perfect Atom-Economy by Titanocene(III) Catalysis

*Pierre Funk, Ruben B. Richrath, Fabian Bohle, Stefan Grimme, and Andreas Gansäuer**

anie_202013561_sm_miscellaneous_information.pdf

# Table of Contents


1. General Information ........ 5

2. General procedures ........ 6

2.1 GP I – Williamson ether synthesis with 1-bromo-3-methyl-2,3-epoxybutane ........ 6

2.2 GP II – $NaBH_4$-mediated reduction of aldehydes ........ 6

2.3 GP III – Williamson ether synthesis with 1-bromo-3-methyl-2-butene ........ 6

2.4 GP IV – Epoxidation of alkenes with *m*CPBA ........ 7

2.5 GP V – Reducitve amination to α-branched 1-phenylethylamines ........ 7

2.6 GP VI – Amidation of primary amines and reduction to *N*-methylated amines ........ 8

2.7 GP VII – Titanocene-catalyzed synthesis of acetals and hemiaminals ........ 8

2.8 GP VIII – Titanocene-catalyzed synthesis of ketone-derived hemiaminals ........ 9

2.9 GP IX – Titanocene-catalyzed synthesis of acetophenone derivatives ........ 9

3. Synthesis of substrates ........ 10

3.1 Benzyl-glycidyl ether synthesis **1a-1i** ........ 10

3.1.1 Synthesis of 1-bromo-3-methyl-2,3-epoxybutane **1a-p1** ........ 10

3.1.2 Synthesis of 3-[(benzyloxy)methyl]-2,2-dimethyloxirane **1a** ........ 11

3.1.3 Synthesis of 2,2-dimethyl-3-{[(4-methylphenyl)methoxy]methyl}oxirane **1b** ........ 12

3.1.4 Synthesis of 3-{[([1,1'-biphenyl]-4-yl)methoxy]methyl}-2,2-dimethyloxirane **1c**. 14

3.1.5 Synthesis of 3-{[(4-methoxyphenyl)methoxy]methyl}-2,2-dimethyloxirane **1d** ........ 18

3.1.6 Synthesis of 3-{[(3-methoxyphenyl)methoxy]methyl}-2,2-dimethyloxirane **1e** ........ 19

3.1.7 Synthesis of 2,2-dimethyl-3-{[(3,4,5-trimethoxyphenyl)methoxy]methyl}oxirane **1f** ........ 23

3.1.8 Synthesis of 2-(bromomethyl)-1-oxaspiro[2.5]octane **1g-p1** ........ 25

3.1.9 Synthesis of 2-{[(4-methylphenyl)methoxy]methyl}-1-oxaspiro[2.5]octane **1g** ........ 26

3.1.10 Synthesis of 2-[(benzyloxy)methyl]-1-oxaspiro[2.5]octane **1h** ........ 28

3.1.11 Synthesis of 2,2-dimethyl-3-{[(4-chlorophenyl)methoxy]methyl}oxirane **1i** ........ 30

3.2 α-Methylbenzyl-glycidyl ether synthesis **3a-3e** ........ 32

3.2.1 Synthesis of 3-{[(2,3-dihydro-1*H*-inden-1-yl)oxy]methyl}-2,2-dimethyloxirane **3a** ........ 32

3.2.2 Synthesis of 2,2-dimethyl-3-{[(1,2,3,4-tetrahydronaphthalen-1-yl)oxy]methyl}-oxirane **3b** ....................34

3.2.3 Synthesis of 2,2-dimethyl-3-[(1-phenylethoxy)methyl]oxirane **3c** ....................36

3.2.4 Synthesis of 2,2-dimethyl-3-{[1-(4-methylphenyl)ethoxy]methyl}oxirane **3d**.....39

3.2.5 Synthesis of 2,2-dimethyl-3-{[1-(4-methoxyphenyl)ethoxy]methyl}oxirane **3e** ..41

3.3 Benzyl amine synthesis **5a-5f**....................44

3.3.1 Synthesis of *N*-benzyl-1-(3,3-dimethyloxiran-2-yl)-*N*-methylmethanamine **5a**..44

3.3.2 Synthesis of *N*,*N*-dibenzyl-1-(3,3-dimethyloxiran-2-yl)methanamine **5b** ..........46

3.3.3 Synthesis of *N*-[(3,3-dimethyloxiran-2-yl)methyl]-*N*-methyl-1-phenylethan-1-amine **5c** ....................48

3.3.4 Synthesis of *N*-[(3,3-dimethyloxiran-2-yl)methyl]-*N*-methyl-1-(4-.. methylphenyl)-ethan-1-amine **5d**....................50

3.3.5 Synthesis of *N*-[(3,3-dimethyloxiran-2-yl)methyl]-1-(3-methoxyphenyl)-*N*-methylethan-1-amine **5e**....................53

3.3.6 Synthesis of *N*-[(3,3-dimethyloxiran-2-yl)methyl]-1-(4-methoxyphenyl)-*N*-methylethan-1-amine **5f** ....................55

3.4 Synthesis of $Cp_2Ti(OMs)_2$....................58

4. Titanocene catalyzed reactions ....................59

4.1 Titanocene catalyzed synthesis of acetals **2a-2i**....................59

4.1.1 Synthesis of 2-phenyl-4-(propan-2-yl)-1,3-dioxolane **2a** ....................59

4.1.2 Synthesis of 2-(4-methylphenyl)-4-(propan-2-yl)-1,3-dioxolane **2b**....................62

4.1.3 Synthesis of 2-([1,1'-biphenyl]-4-yl)-4-(propan-2-yl)-1,3-dioxolane **2c** .............64

4.1.4 Synthesis of 2-(4-methoxyphenyl)-4-(propan-2-yl)-1,3-dioxolane **2d**...............67

4.1.5 Synthesis of 2-(3-methoxyphenyl)-4-(propan-2-yl)-1,3-dioxolane **2e**...............72

4.1.6 Synthesis of 4-(propan-2-yl)-2-(3,4,5-trimethoxyphenyl)-1,3-dioxolane **2f** .......74

4.1.7 Synthesis of 4-cyclohexyl-2-(4-methylphenyl)-1,3-dioxolane **2g** ....................76

4.1.8 Synthesis of 4-cyclohexyl-2-phenyl-1,3-dioxolane **2h**....................78

4.1.9 Synthesis of 2-(4-chlorophenyl)-4-(propan-2-yl)-1,3-dioxolane **2i** ....................80

4.2 Titanocene catalyzed synthesis of ketone-dervied acetals **4a-4e** ....................83

4.2.1 Synthesis of 4-(propan-2-yl)-2',3'-dihydrospiro[[1,3]dioxolane-2,1'-indene] **4a** .83

4.2.2 Synthesis of 4-(propan-2-yl)-3',4'-dihydro-2'*H*-spiro[[1,3]dioxolane-2,1'-naphthalene] **4b** ........ 85

4.2.3 Synthesis of 2-methyl-2-phenyl-4-(propan-2-yl)-1,3-dioxolane **4c** ........ 87

4.2.4 Synthesis of 2-methyl-2-(4-methylphenyl)-4-(propan-2-yl)-1,3-dioxolane **4d** ... 89

4.2.5 Synthesis of 2-methyl-2-(4-methoxyphenyl)-4-(propan-2-yl)-1,3-dioxolane **4e** . 91

4.3 Titanocene catalyzed synthesis of hemiaminals **6a-6f** ........ 95

4.3.1 Synthesis of 3-methyl-2-phenyl-5-(propan-2-yl)-1,3-oxazolidine **6a** ........ 95

4.3.2 Synthesis of 3-benzyl-2-phenyl-5-(propan-2-yl)-1,3-oxazolidine **6b** ........ 98

4.3.3 Synthesis of 3-methyl-2-phenyl-5-(propan-2-yl)-1,3-oxazolidine **6c** ........ 100

4.3.4 Synthesis of 2,3-dimethyl-2-(4-methylphenyl)-5-(propan-2-yl)-1,3-oxazolidine **6d** ........ 102

4.3.5 Synthesis of 2,3-dimethyl-2-(3-methoxyphenyl)-5-(propan-2-yl)-1,3-oxazolidine **6e** ........ 104

4.3.6 Synthesis of 2,3-dimethyl-2-(4-methoxyphenyl)-5-(propan-2-yl)-1,3-oxazolidine **6f** ........ 106

4.4 Catalysis with subsequent acidic workup to carbonyls **4b-2, 6e-2, 6f-2** ........ 109

4.4.1. Synthesis of tetralon **4b-2** ........ 109

4.4.2 Synthesis of 3-methoxy acetophenone **6e-2** ........ 110

4.4.3 Synthesis of 4-methoxy acetophenone **6f-2** ........ 112

5. DFT Calculations ........ 114

6. References ........ 133

# 1. General Information

All reactions involving air- or moisture sensitive compounds were carried out in oven-dried glassware under argon using standard SCHLENK and vacuum line technique. All solvents were either dried and deoxygenated by distillation (THF over Na) before use or purified inside a *M-Braun MB-SPS-800* solvent purification system and used after degasification. Commercially available chemicals were used without further purification. $^{1}$H-NMR and $^{13}$C-NMR spectra were recorded on *Bruker* spectrometers. $^{1}$H-NMR chemical shifts were given in ppm and calibrated by using the residual and deuterated solvent as internal reference [$CHCl_3$ (7.26 ppm), $C_6HD_5$ (7.16 ppm)]. If a signal exhibits two similar coupling constants, it might result in a *pseudo* resolution. The signal is then signed with a *p*. $^{13}$C-NMR chemical shifts were given in ppm and calibrated by using the solvent peak as internal reference [$CHCl_3$ (77.16 ppm), $C_6D_6$ (128.06 ppm)]. The diastereomeric ratio is determined by comparing integrals of the benzylic hydrogen in $^{1}$H-NMR spectra. If there is no benzylic hydrogen, methyl groups of the isopropyl group are used to determine the ratio. NOESY spectra were only measured for some acetals and hemiaminals in order to prove the relative configuration. The relative configuration of the other acetals and hemiaminals was then determined by comparing the chemical shift pattern in $^{1}$H-spectra. For quantitative $^{1}$H-NMR spectrosocy dimethyl terephthalate (10 – 20 mg per sample) was used as internal standard. IR spectra were measured on an ATR-IR Spectrometer *NicoletTM 380* instrument as neat film. High resolution mass spectra analysis data were obtained on a *Thermoquest MAT 95 CL* instrument by ESI (+) measurement. Column chromatography was carried out on silica gel (230-400 mesh) supplied by *Merck* and *Macherey- Nagel* or on neutral alumina (Brockmann grade) supplied by *Merck*. TLC was performed on silica gel or aluminum plates and the compounds were detected with the *Seebach* staining mixture. Employed solvent mixtures consisted of ethyl acetate (EA) in cyclohexane (CH) or diethylether ($Et_2O$) and pentane. In some cases triethylamine (TEA) was added for column chromatography. $R_f$ values were measured on silica plates using UV light as visualizing agent (if applicable) and a solution of ammoniummolybdate tetrahydrate (25 g/l) and $Ce(SO_4)_2\cdot4H_2O$ (10 g/l) in 10% aqueous $H_2SO_4$ followed by heating as developing agents.

# 2. General procedures

## 2.1 GP I – Williamson ether synthesis with 1-bromo-3-methyl-2,3-epoxybutane

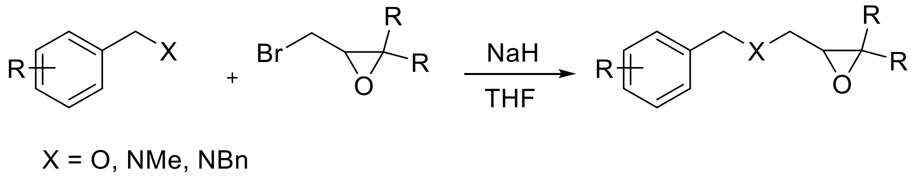


To a solution of a benzyl alcohol or amine in THF (0.2 mmol/ml), an epibromohydrin derivative is added. Then NaH (60% dispersion in mineral oil) is slowly added. The mixture is stirred at room temperature and the reaction progress is monitored by TLC. After full consumption of the substrate the reaction is quenched by addition of $H_2O$ (2 ml/mmol) at 0 °C and THF is evaporated under reduced pressure. Subsequently the aqueous phase is extracted three times with DCM, the combined organic extracts are washed with brine, dried over $MgSO_4$ and the solvent was removed under reduced pressure. The crude product was purified via column chromatography ($SiO_2$) to yield the product as a colourless oil.

## 2.2 GP II – $NaBH_4$-mediated reduction of aldehydes

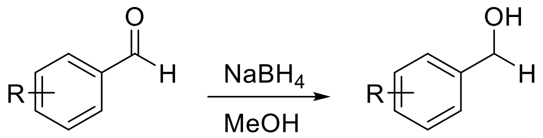


The reaction is performed according a literature procedure.[1] A benzaldehyde derivative is dissolved in MeOH (0.5 mmol/ml) and cooled to 0 °C. Then $NaBH_4$ is slowly added and the mixture is stirred for the given time while warming up to room temperature. The reaction is quenched with $H_2O$ (2 ml/mmol) and methanol is removed under reduced pressure. The aqueous phase is extracted three times with DCM or EA, the combined organic phases are washed with brine and dried over $MgSO_4$. The solution is concentrated under reduced pressure. The crude product is obtained as a colourless solid and used without further purification.

## 2.3 GP III – Williamson ether synthesis with 1-bromo-3-methyl-2-butene

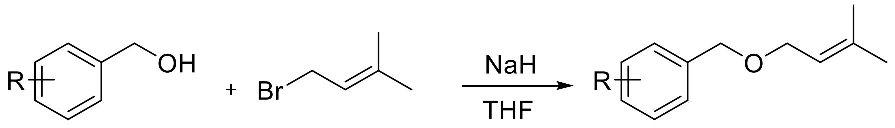

3,3-Dimethylallylbromide (1.0 eq.) and a benzyl alcohol derivative (1.1 eq.) are dissolved in THF (0.25 mmol/ml) and cooled to 0 °C. Then NaH (60% dispersion in mineral oil, 1.4 eq.) is added portionwise and the mixture is heated up to 60 °C. The reaction progress in monitored by TLC. After full consumption of the 3,3-dimethylallylbromide the reaction is cooled to room temperature and quenched with $H_2O$ (2 ml/mmol). The phases were separated and the aqueous layer is extracted three times with DCM. The combined organic extracts are washed three times with $H_2O$ and once with brine. After drying over $MgSO_4$ the solvent is removed under reduced pressure. The crude product is purified by column chromatography ($SiO_2$, eluent: CH:EA, 95:5) and isolated as a colourless oil.

## 2.4 GP IV – Epoxidation of alkenes with *m*CPBA

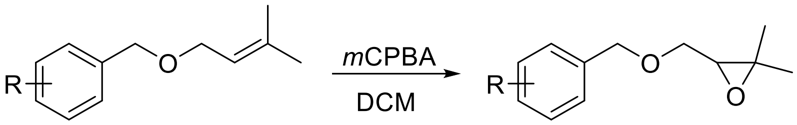


The reaction is performed according a procedure of *Ivasyshyn*.[2] The allylbenzylether derivative (1.0 eq.) is dissolved in DCM (0.2 mmol/ml) and cooled to 0 °C. Then *m*CPBA (77%, 1.5 eq.) is added portionwise and the mixture is stirred at room temperature. The reaction progress is monitored by TLC. After full consumption of the substrate, the mixture is washed several times with 2 M NaOH, once with brine and dried over $MgSO_4$. The solvent is removed under reduced pressure and the crude product is purified by column chromatography ($SiO_2$, eluent: CH:EA, 95:5) and isolated as a colourless oil.

## 2.5 GP V – Reducitve amination to α-branched 1-phenylethylamines

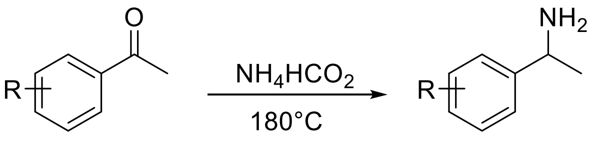


Substituted 1-phenylethanamines are synthesized by hydrogenative reductive amination of ketones.[3] In a flame dried three-neck flask, equipped with a Dean-Stark vessel and a reflux condenser, an acetophenone derivative (100 mmol, 1.0 eq.) and ammonium formate (15.8 g, 250 mmol, 2.5 eq.) is slowly heated to 155 °C until the two-phases become a homogeneous mixture. $H_2O$, acetophenone-derivative and ammonium carbonate are distilled off until the temperature reached to 185 °C for 20 h while generating $NH_3$ and $CO_2$. The mixture is allowed to cool down to room

temperature, washed with $H_2O$ two times to remove ammonium formate and ammonium carbonate. 20 ml concentrated HCl are added to the organic phase which is then refluxed for 1h. The acidic aqueous phase is basified with 2M NaOH to pH 12 and then extracted with DCM three times. The organic phase was dried over $NaOH_{(s)}$ and concentrated *in vacuo*. The residue was used without further purification. Different substituted α-branched amines are synthesized according to this procedure whose spectroscopic data matched those previously reported.

## 2.6 GP VI – Amidation of primary amines and reduction to *N*-methylated amines

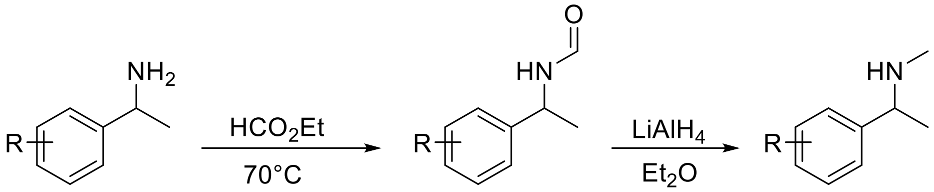


The reaction is performed according to a literature procedure.[4] A solution of amine in ethyl formate (0.3 mmol/ml) was heated at 70 °C for 2 h and then, the reaction was concentrated under reduced pressure to afford formamide product in quantitative yield. The crude material was used in the next step without purification.

The formamide is slowly added to a suspension of $LiAlH_4$ (x g 2.0 eq.) in $Et_2O$ at -78 °C. The flask is warmed to room temperature and the contents stirred for 12 h under argon. The reaction was quenched at 0 °C by the sequential addition of x ml $H_2O$, x ml 15% aqueous NaOH, (3*x ml) $H_2O$. The inorganics solids are removed by filtration, and the ether filtrate was dried over $MgSO_4$. Filtration and evaporation of the solvent yields product as a homogenous colourless oil. The crude product is used without further purification.

## 2.7 GP VII – Titanocene-catalyzed synthesis of acetals and hemiaminals

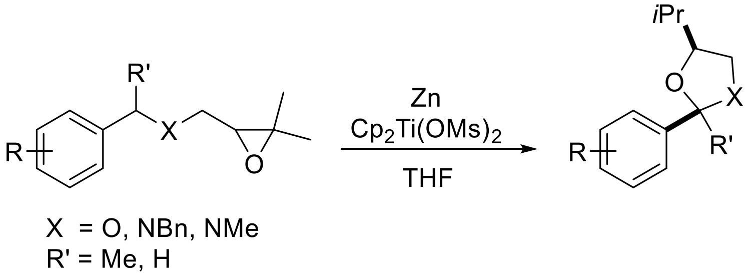


In a flame dried schlenk flask $Cp_2Ti(OMs)_2$ (0.1 eq.) and zinc dust (0.2 eq.) are placed under Ar. After successive addition of the substrate (1.0 eq.) and THF (0.1 mmol/ml), the mixture is refluxed for 3 h. Subsequently the reaction is exposed to air and allowed

to cool to room temperature. With EA the solution is flushed through a short filtration column ($SiO_2$) and the solvent is removed in vacuo. The crude product is purified by column chromatography ($SiO_2$, eluent: CH:EA).

## 2.8 GP VIII – Titanocene-catalyzed synthesis of ketone-derived hemiaminals

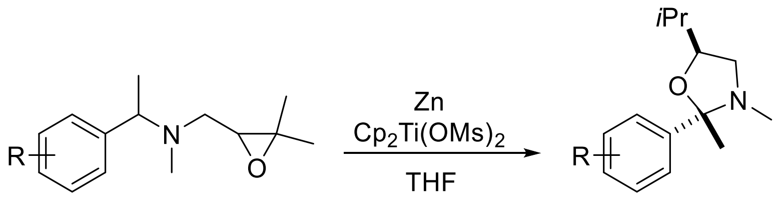


$Cp_2Ti(OMs)_2$ (0.1 eq.) and zinc dust (0.2 eq.) are placed in a flame dried schlenk flask under Ar. After addition of the substrate (1.0 eq.) and THF (0.1 mmol/ml), the mixture is refluxed for 3 h. Subsequently the reaction is exposed to air and allowed to cool to room temperature. The mixture is filtrated through a plug of cotton wool and the solvent is removed under reduced pressure. Finally the residue is dissolved in $Et_2O$ and filtrated again through a plug of cotton wool. The solvent is removed under reduced pressure and the crude product is obtained as a light yellow oil.

## 2.9 GP IX – Titanocene-catalyzed synthesis of acetophenone derivatives

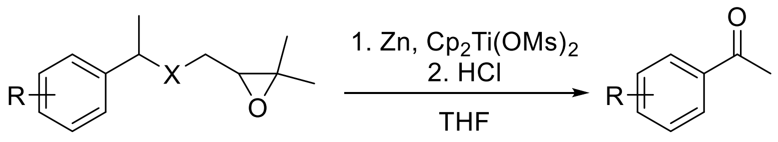


X = O, NMe

$Cp_2Ti(OMs)_2$ (0.1 eq.) and zinc dust (0.2 eq.) are placed in a flame dried schlenk flask under Ar. After addition of the substrate (1.0 eq.) and THF (0.1 mmol/ml), the mixture is refluxed for 3 h. Subsequently the reaction is exposed to air and allowed to cool to room temperature. Then 2 M HCl is added and stirring is continued for 30 min. The reaction solution is extracted three times with diethyl ether and washed with sat. $NaHCO_3$ solution and brine. The solvent is dried over $Na_2SO_4$ and removed under reduced pressure (100 mbar, 40 °C). The crude product is purified by column chromatography ($SiO_2$, eluent: $Et_2O$: pentane).

# 3. Synthesis of substrates

## 3.1 Benzyl-glycidyl ether synthesis **1a-1i**

### 3.1.1 Synthesis of 1-bromo-3-methyl-2,3-epoxybutane **1a-p1**

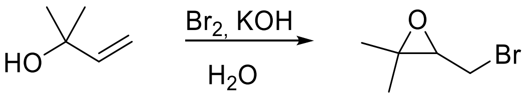


The reaction is performed in accordance to a literature procedure.[5] To a stirred solution of 189 g KOH (85%, 4.4 eq., 2.86 mol) in 500 ml $H_2O$ is slowly added 50 ml bromine (1.5 eq., 975 mmol) at 5 °C via a dropping funnel. The mixture is stirred for 10 min at the same temperature. Then, to this solution is added 68 ml 2-methyl-3-buten-2-ol (1.0 eq., 650 mmol) at 10 °C and stirred for 70 h at room temperature. The mixture is diluted with 300 ml CH, the aqueous layer is extracted once with 120 ml CH and the combined organic extracts are dried over $MgSO_4$. The solvent is removed under reduced pressure. The crude product (84.0 g, 509 mmol, 78%) is used without further purification.

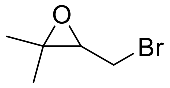


$C_5H_9BrO$
165,03 g/mol

**$^1$H-NMR (300 MHz, $C_6D_6$, RT): δ [ppm] =** 3.01 (dd, *J* = 9.7, 5.5 Hz, 1H), 2.76 (dd, *J* = 9.7, 7.3 Hz, 1H), 2.70 (dd, *J* = 7.3, 5.5 Hz, 1H), 0.95 (s, 3H), 0.89 (s, 3H).

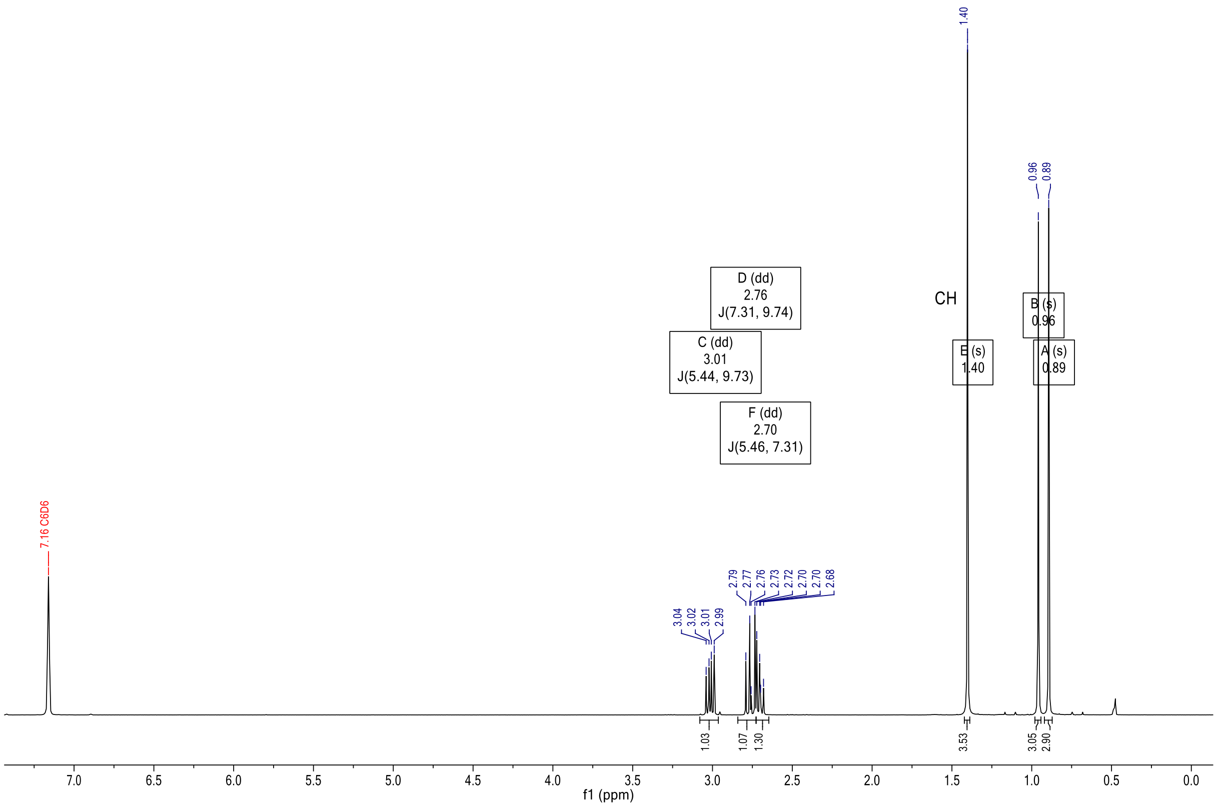

Analytical data in accordance with literature.[5]

### 3.1.2 Synthesis of 3-[(benzyloxy)methyl]-2,2-dimethyloxirane **1a**

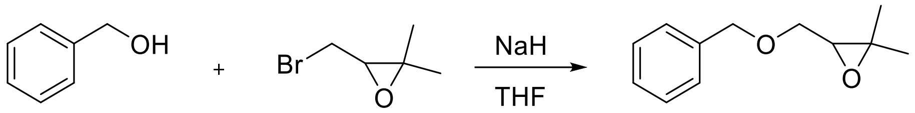


According to GP I 1.12 g benzyl alcohol (10.4 mmol, 1.1 eq.), 1.56 g 1-bromo-3-methyl-2,3-epoxybutane (9.45 mmol, 1.0 eq.) and 530 mg NaH (60% dispersion in mineral oil, 13.2 mmol, 1.4 eq.) are reacted in THF for 17 h. Column chromatography ($SiO_2$, eluent: CH:EA, 95:5) afforded 1.29 g **1a** (6.69 mmol, 71%) as a light yellow oil.

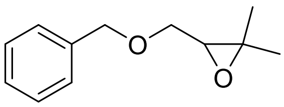


**1a**
$C_{12}H_{16}O_2$
192,26 g/mol

**$R_f$ =** 0.51 (20% EA, 80% CH). **$^1$H-NMR (400 MHz, $C_6D_6$, RT): δ [ppm] =** 7.31 – 7.26 (m, 2H), 7.20 – 7.13 (m, 2H), 7.12 – 7.06 (m, 1H), 4.42 (d, *J* = 12.1 Hz, 1H), 4.30 (d, *J* = 12.1 Hz, 1H), 3.45 (dd, *J* = 11.0, 5.0 Hz, 1H), 3.42 (dd, *J* = 11.0, 5.5 Hz, 1H), 2.90 (*p*t, *J* = 5.3 Hz, 1H), 1.06 (s, 3H), 1.00 (s, 3H). **$^{13}$C-NMR (100 MHz, $C_6D_6$): δ [ppm] =** 138.9, 128.6, 127.9, 127.8, 73.2, 69.4, 61.9, 56.8, 24.7, 18.9. **IR:** 2963, 2926 2859, 1454, 1379, 1087, 1028, 866, 812, 736, 697, 610, 480 cm$^{-1}$. **HRMS (ESI+):** *m/z* calculated for $C_{12}H_{16}O_2Na^+$: 215.1043 u, found: 215.1044 u.

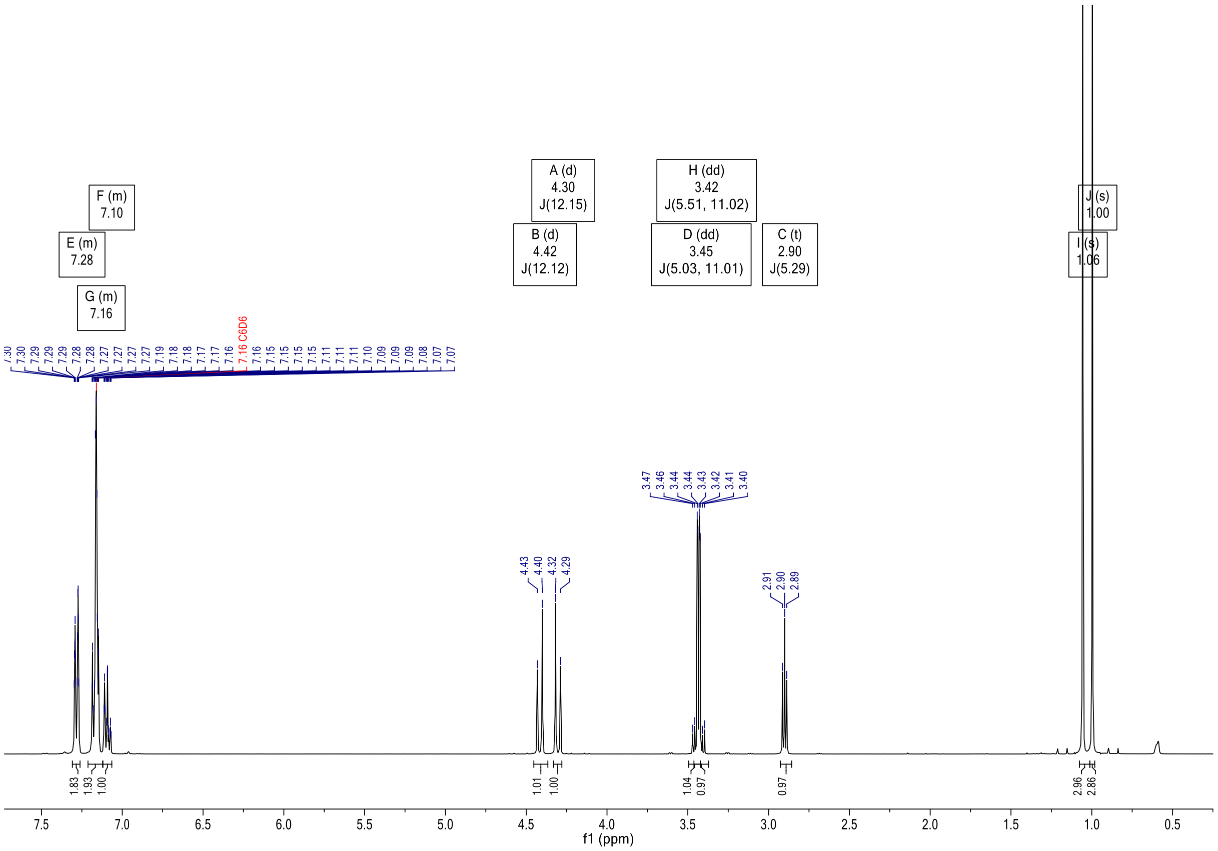

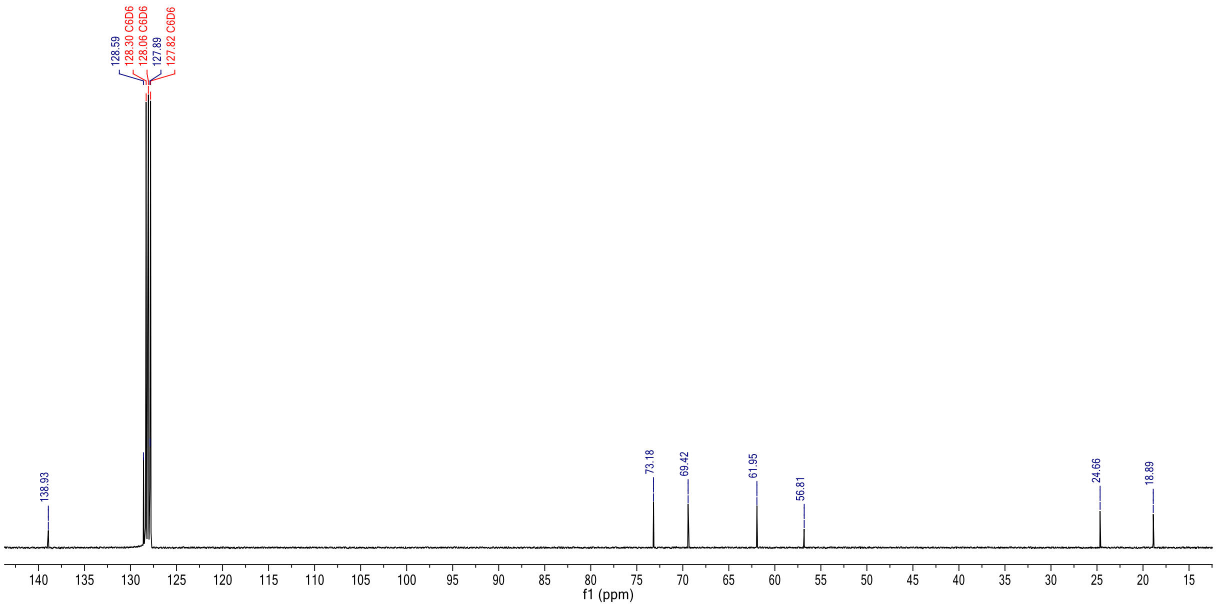


### 3.1.3 Synthesis of 2,2-dimethyl-3-{[(4-methylphenyl)methoxy]methyl}oxirane **1b**

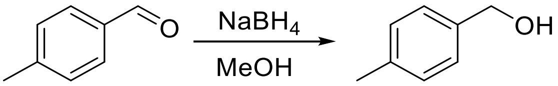


According to GP II 3.24 g 4-methylbenzaldehyde (27.0 mmol, 1.0 eq.) and 1.02 g $NaBH_4$ (27.0 mmol, 1.0 eq.) are reacted for 1.5 h in MeOH. DCM is used for extraction. Workup yielded 3.07 g **1b-p** (25.2 mmol, 93%) as a white solid, which is used without further purification.

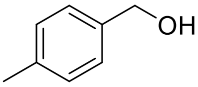


**1b-p**
$C_7H_{10}O$
122,17 g/mol

Analytical data is in accordance with literature.[1]

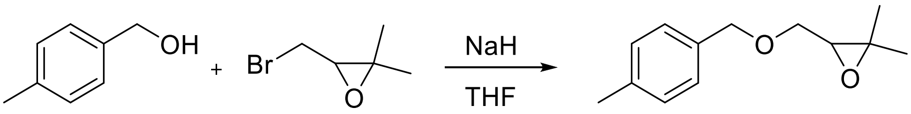


According to GP I 3.07 g 4-methylbenzyl alcohol **1b-p** (25.2 mmol, 1.1 eq.), 3.77 g 1-bromo-3-methyl-2,3-epoxybutane (22.9 mmol, 1.0 eq.) and 1.28 g NaH (60%

dispersion in mineral oil, 32.0 mmol, 1.4 eq.) are reacted for 18 h in THF. Column chromatography ($SiO_2$, eluent: CH:EA, 95:5) afforded 2.95 g **1b** (14.3 mmol, 63%) as a light yellow oil.

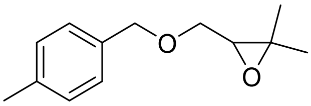

**1b**
$C_{13}H_{18}O_2$
206,29 g/mol

**$R_f$ = 0.41** (10% EA, 90% CH). **$^1$H-NMR (300 MHz, $C_6D_6$, RT): δ [ppm] =** 7.23 (d, *J* = 7.8 Hz, 2H), 7.01 (d, *J* = 7.8 Hz, 2H), 4.44 (d, *J* = 11.9 Hz, 1H), 4.33 (d, *J* = 11.9 Hz, 1H), 3.46 (d, *J* = 5.3 Hz, 2H), 2.92 (t, *J* = 5.3 Hz, 1H), 2.11 (s, 3H), 1.06 (s, 3H), 1.01 (s, 3H). **$^{13}$C-NMR (75 MHz, $C_6D_6$, RT): δ [ppm] =** 137.2, 136.0, 129.3, 128.1, 73.1, 69.3, 62.0, 56.8, 24.7, 21.2, 18.9. **IR:** 1455, 1379, 1083, 866, 802, 753, 487, 470, 405 cm$^{-1}$. **HRMS (ESI+):** *m/z* calculated for $C_{13}H_{18}O_2Na^+$: 229.1199 u, found: 229.1201 u.

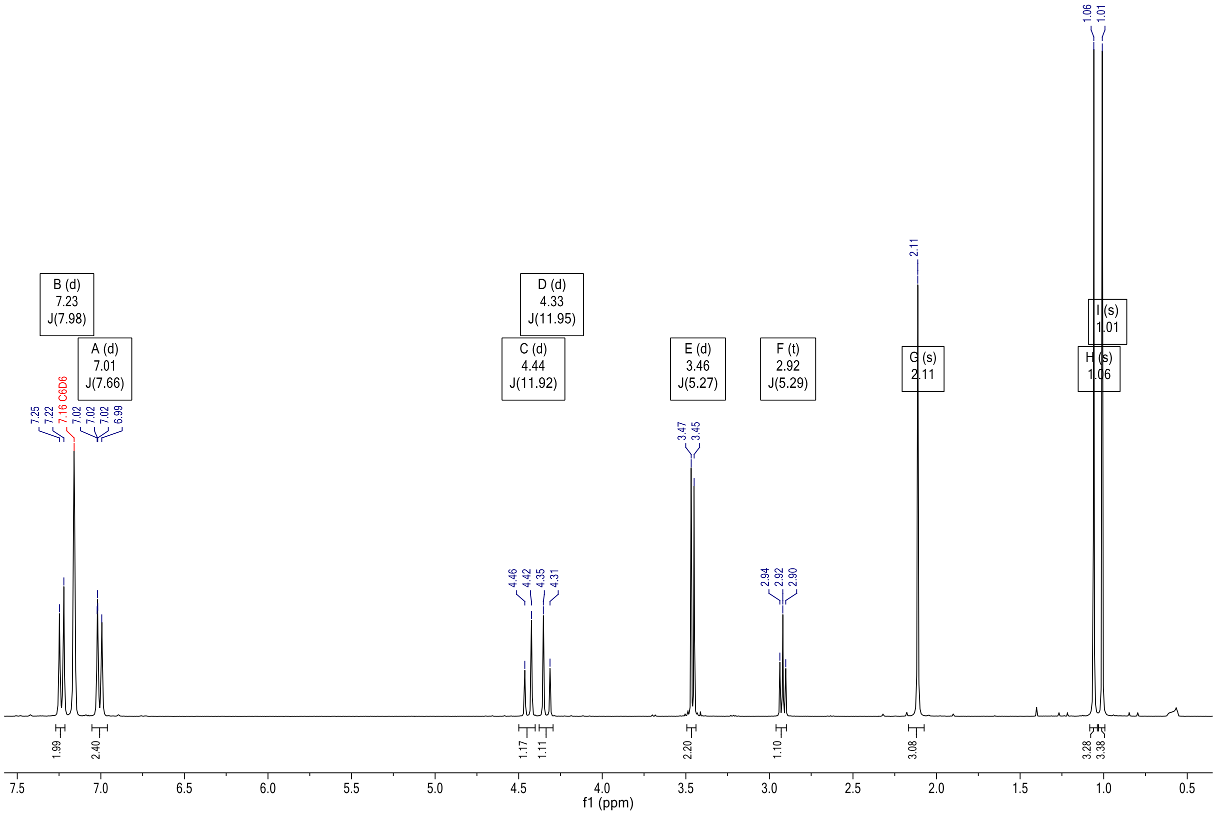

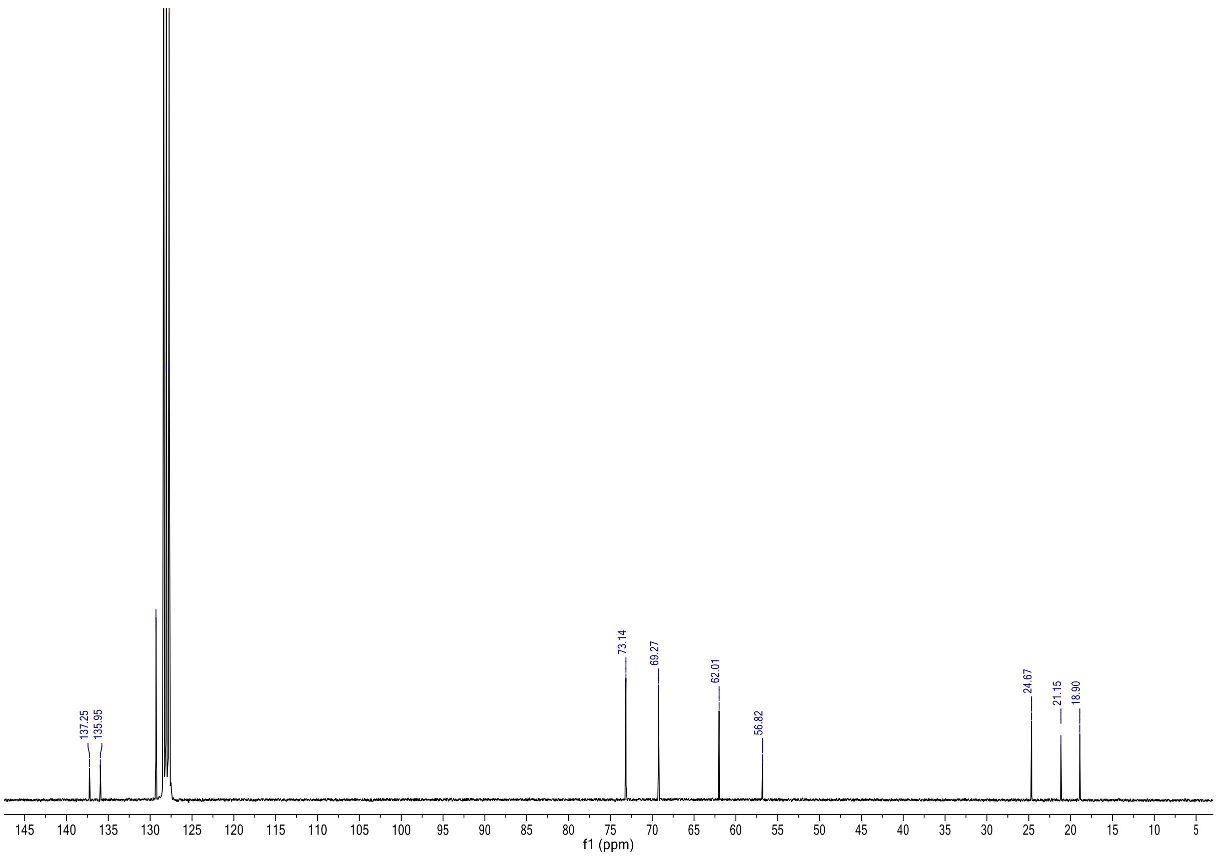

3.1.4 Synthesis of 3-{[([1,1'-biphenyl]-4-yl)methoxy]methyl}-2,2-dimethyloxirane **1c**

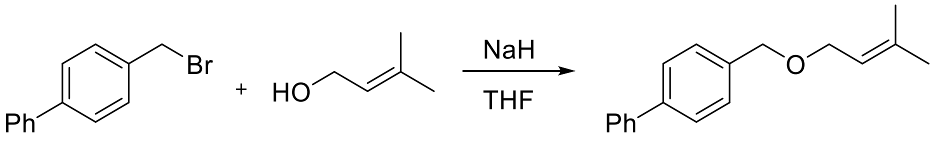


974 mg Prenol (11.3 mmol, 1.4 eq.) is dissolved in 25 ml THF and cooled to 0 °C. Subsequently 628 mg NaH (60% dispersion in mineral oil, 15.4 mmol, 1.9 eq.) is slowly added. The mixture is stirred for 15 min at room temperature. Then 1.99 g 4-bromomethyl biphenyl (8.09 mmol, 1.0 eq.) is added and the mixture is warmed up to 60 °C. The reaction progress is monitored by TLC. After full consumption of 4-bromomethyl biphenyl the mixture is quenched with $H_2O$. The layers are separated and the aqueous phase is extracted three times with CH. The combined organic extracts are washed wither $H_2O$ three times and once with brine. The solution is dried over $MgSO_4$ and the solvent is removed under reduced pressure. After purification by column chromatography ($SiO_2$, eluent: CH:EA, 95:5) 1.26 g (4.98 mmol, 61%) of the product **1c-p** is isolated as a colourless oil.

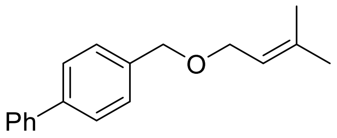


**1c-p**
$C_{18}H_{20}O$
252,36 g/mol

**R$_f$ =** 0.23 (5% EA, 95% CH). **$^1$H-NMR (400 MHz, $C_6D_6$, RT): δ [ppm] =** 7.52 – 7.45 (m, 4H), 7.42 – 7.36 (m, 2H), 7.26 – 7.18 (m, 2H), 7.15 – 7.09 (m, 1H), 5.54 (ddt, *J* = 8.1, 5.3, 1.4 Hz, 1H), 4.44 (s, 2H), 4.07 – 3.96 (m, 2H), 1.66 – 1.58 (m, 3H), 1.56 – 1.43 (m, 3H). **$^{13}$C-NMR (101 MHz, $C_6D_6$, RT): δ [ppm]** = 141.5, 140.8, 138.7, 135.9, 129.1, 127.5, 127.4, 122.5, 71.7, 67.0, 25.8, 18.0. **IR:** 2914, 2853, 1487, 1448, 1377, 1360, 1114, 1073, 1007, 933, 822, 758, 735, 696, 547, 494, 447 cm$^{-1}$. **HRMS (ESI+):** *m/z* calculated for $C_{18}H_{21}O^+$: 253.1587 u, found: 253.1572 u.

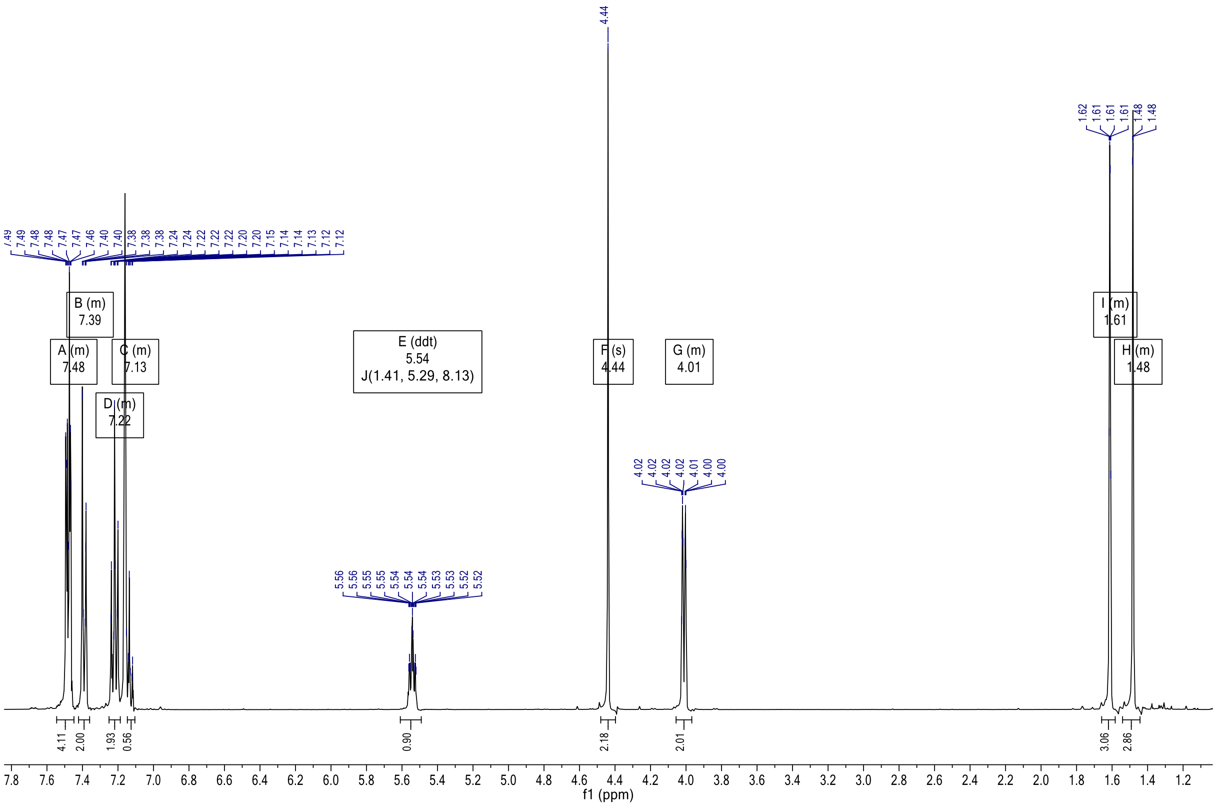

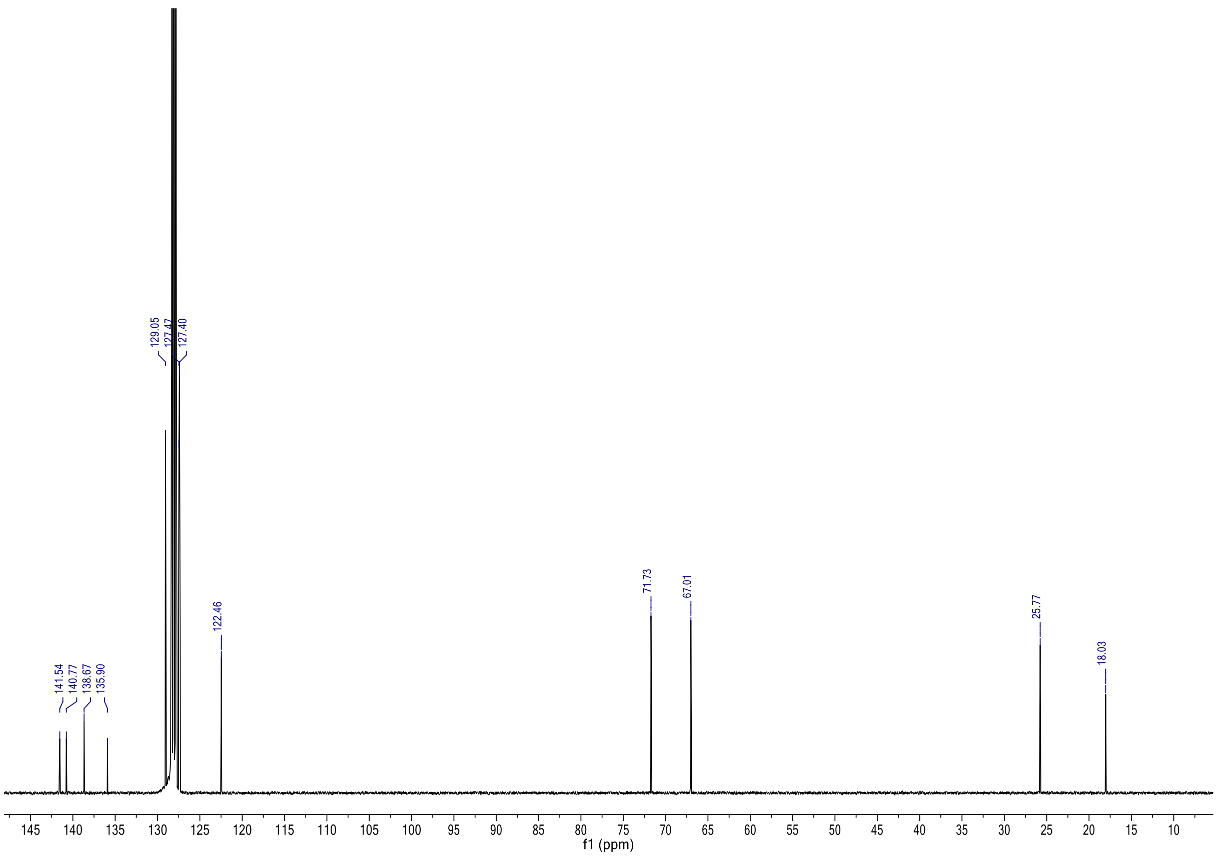

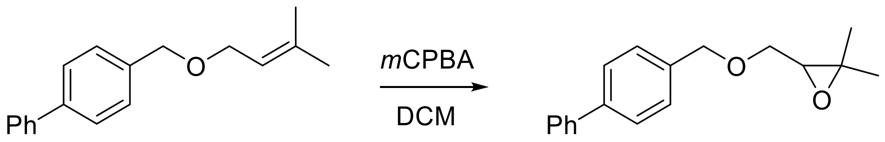


According to GP IV 1.17 g **1c-p** (4.63 mmol) are reacted with 3.43 g *m*CPBA (13.9 mmol). Column chromatography ($SiO_2$, eluent: CH:EA, 95:5) afforded 1.10 g **1c** (4.10 mmol, 88%) as a colourless oil.

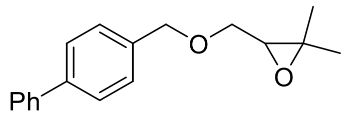


**1c**
$C_{18}H_{20}O_2$
268,36 g/mol

**$R_f$ =** 0.32 (10% EA, 90% CH). **$^1$H-NMR (300 MHz, $C_6D_6$, RT): δ [ppm] =** 7.52 – 7.42 (m, 4H), 7.36 – 7.31 (m, 2H), 7.26 – 7.18 (m, 2H), 7.15 – 7.10 (m, 1H), 4.48 (d, *J* = 12.2 Hz, 1H), 4.36 (d, *J* = 12.2 Hz, 1H), 3.49 (dd, *J* = 11.0, 4.9 Hz, 1H), 3.48 (d, *J* = 11.0, 5.6 Hz, 1H), 2.94 (*p*t, *J* = 5.3 Hz, 1H), 1.08 (s, 3H), 1.02 (s, 3H). **$^{13}$C-NMR (75 MHz, $C_6D_6$, RT): δ [ppm] =** 141.4, 141.0, 137.9, 129.1, 127.5, 127.5, 127.5, 127.4, 72.9, 69.5, 62.0, 56.8, 24.7, 18.9. **IR:** 2963, 2859, 1488, 1450, 1409, 1379, 1246, 1212, 1083, 1008, 909, 849, 823, 759, 697, 497 $cm^{-1}$. **HRMS (ESI+):** *m/z* calculated for $C_{18}H_{20}O_2Na^+$: 291.1356 u, found: 291.1351 u.

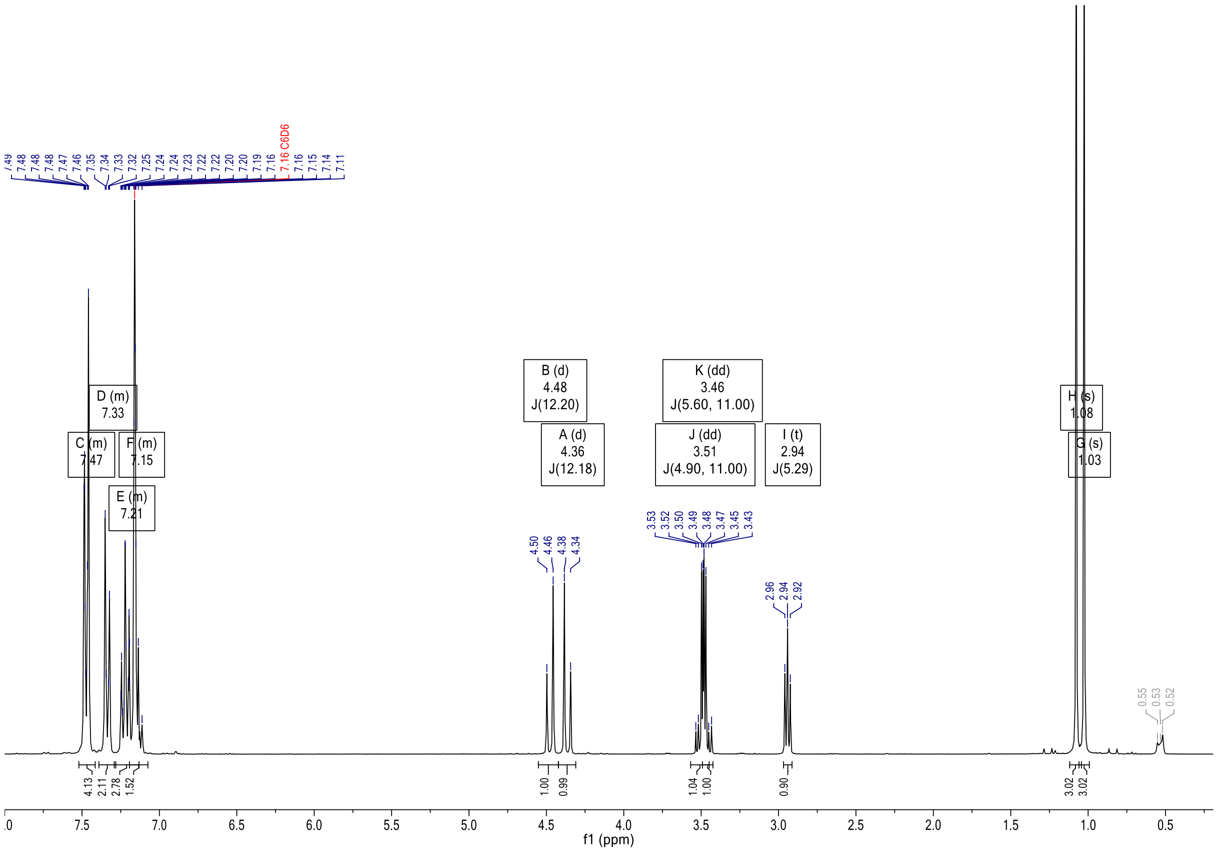
7.16 C6D6
C (m)
7.47
D (m)
7.33
E (m)
7.21
F (m)
7.15
B (d)
4.48
J(12.20)
A (d)
4.36
J(12.18)
K (dd)
3.46
J(5.60, 11.00)
J (dd)
3.51
J(4.90, 11.00)
I (t)
2.94
J(5.29)
H (s)
1.08
G (s)
1.03
4.50
4.46
4.38
4.34
3.53
3.52
3.50
3.49
3.48
3.47
3.45
3.43
2.96
2.94
2.92
4.13
2.11
2.78
1.52
1.00
0.99
1.04
1.00
0.90
3.02
3.02
f1 (ppm)

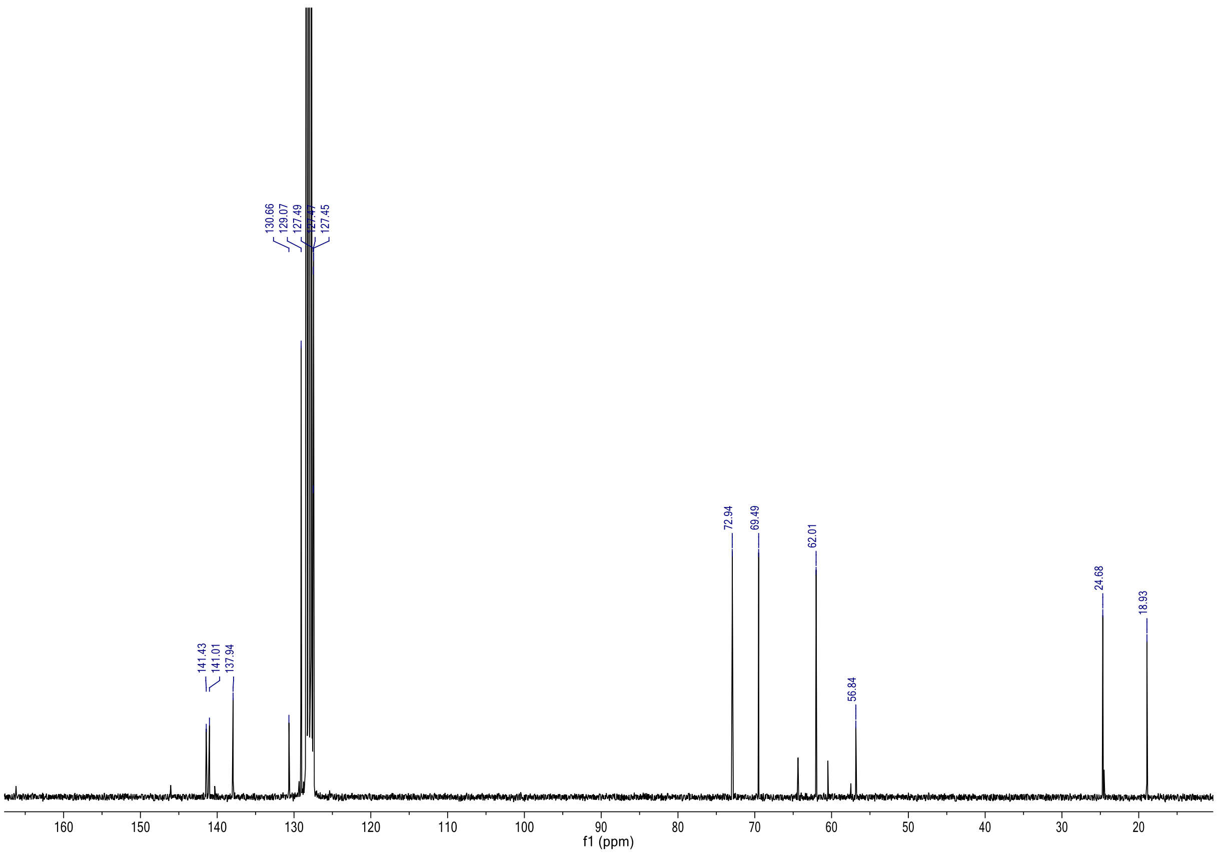
130.66
129.07
127.49
127.47
127.45
141.43
141.01
137.94
72.94
69.49
62.01
56.84
24.68
18.93
f1 (ppm)

### 3.1.5 Synthesis of 3-{[(4-methoxyphenyl)methoxy]methyl}-2,2-dimethyloxirane **1d**

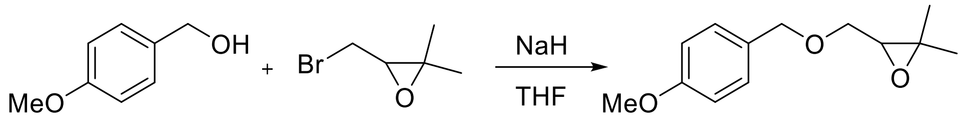


According to GP I 1.24 g 4-methoxybenzyl alcohol (9.00 mmol, 1.1 eq.), 1.35 g 1-bromo-3-methyl-2,3-epoxybutane (8.18 mmol, 1.0 eq.) and 460 mg NaH (60% dispersion in mineral oil, 11.5 mmol, 1.4 eq.) are reacted for 20 h in THF. Column chromatography ($SiO_2$, eluent: CH:EA, 95:5) afforded 1.24 g **1d** (5.60 mmol, 68%) as a colourless oil.

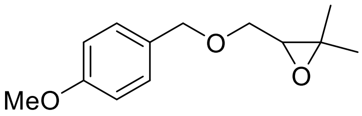


**1d**
$C_{13}H_{18}O_3$
222,28 g/mol

**$R_f$ =** 0.37 (20% EA, 80% CH). **$^1$H-NMR (300 MHz, $C_6D_6$, RT): δ [ppm] =** 7.22 (d, *J* = 8.7 Hz, 2H), 6.79 (d, *J* = 8.7 Hz, 2H), 4.43 (d, *J* = 11.7 Hz, 1H), 4.32 (d, *J* = 11.7 Hz, 1H), 3.47 (d, *J* = 5.3 Hz, 2H), 3.30 (s, 3H), 2.93 (t, *J* = 5.3 Hz, 1H), 1.07 (s, 3H), 1.02 (s, 3H). **$^{13}$C-NMR (75 MHz, $C_6D_6$, RT): δ [ppm] =** 159.8, 130.9, 129.6, 114.1, 72.9, 69.1, 62.0, 56.8, 54.8, 24.7, 18.9. **IR:** 2961, 2929, 1612, 1512, 1457, 1379, 1302, 1244, 1173, 1112, 1080, 1033, 848, 815, 758, 580 cm$^{-1}$. **HRMS (ESI+):** *m/z* calculated for $C_{13}H_{18}O_3Na^+$: 245.1148 u, found: 245.1140 u.

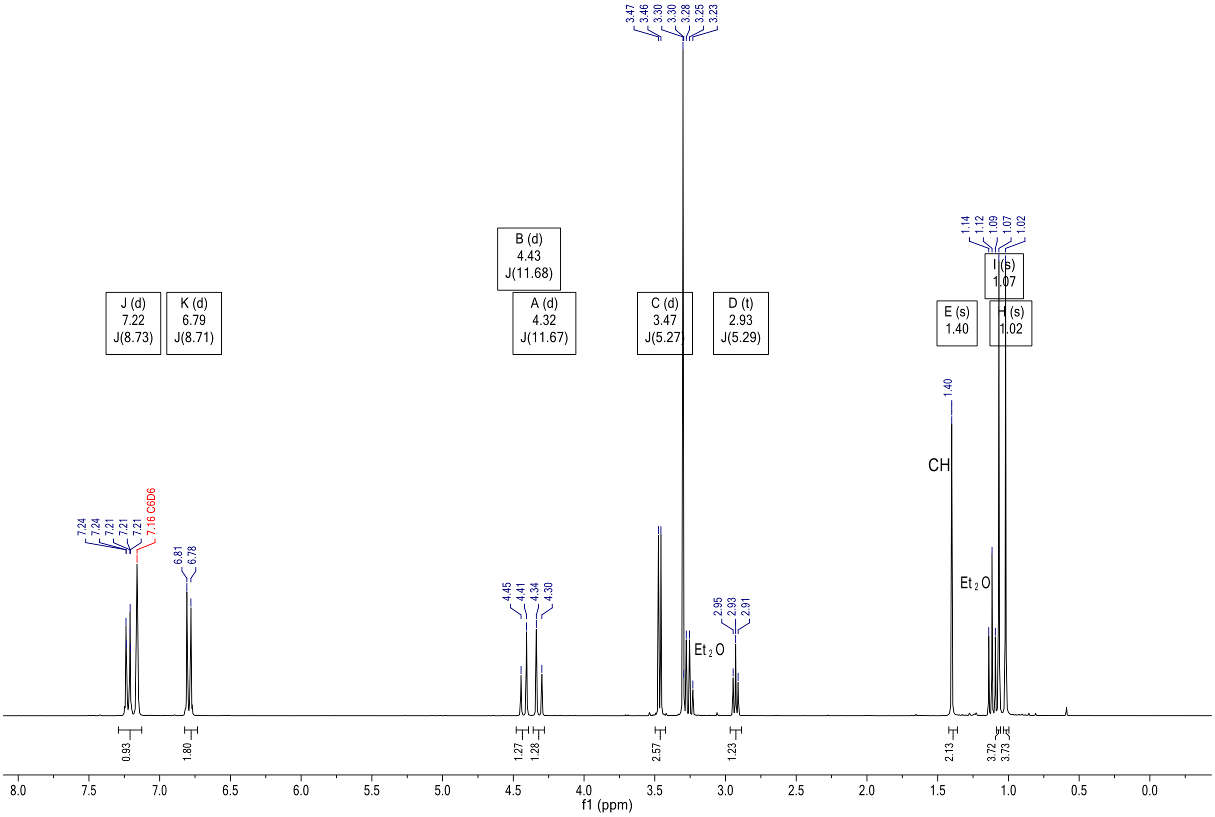

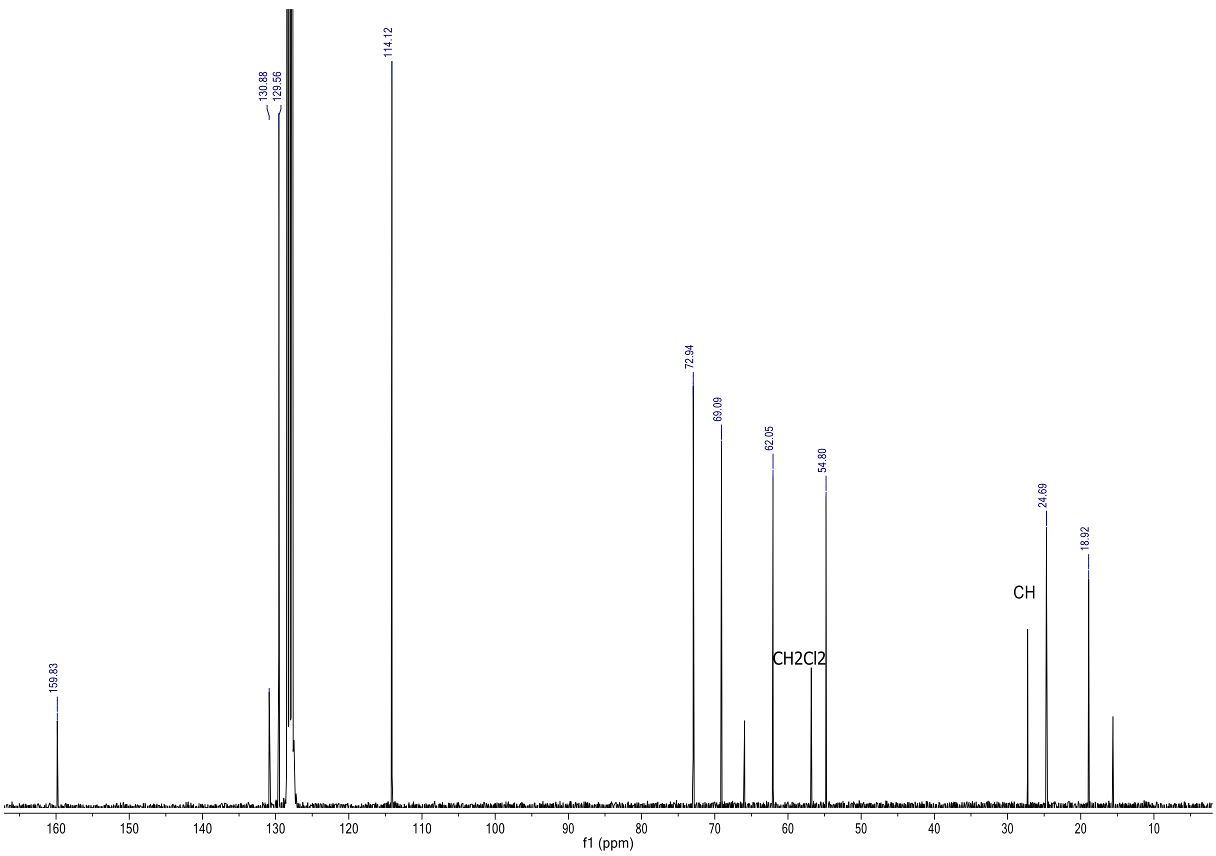


### 3.1.6 Synthesis of 3-{[(3-methoxyphenyl)methoxy]methyl}-2,2-dimethyloxirane **1e**

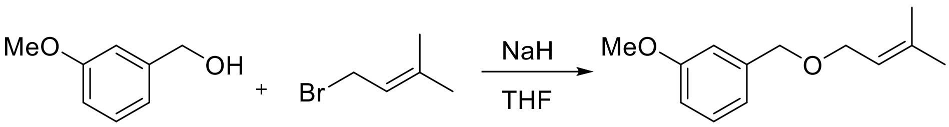


According to GP III 2.80 g 3-methoxybenzyl alcohol (20.3 mmol, 1.1 eq.), 2.75 g 3,3-dimethylallylbromide (18.4 mmol, 1.0 eq.) and 1.03 g NaH (60% dispersion in mineral oil, 25.8 mmol, 1.4 eq.) are reacted in THF for 21 h. Column chromatography ($SiO_2$, eluent: CH:EA, 95:5) afforded 2.54 g **1e-p** (12.3 mmol, 67%) as a colourless oil.

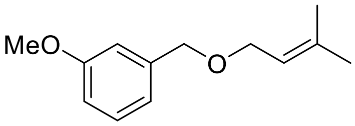


**1e-p**
$C_{13}H_{18}O_2$
206,29 g/mol

**$R_f$ =** 0.42 (5% EA, 95% CH). **$^1$H-NMR (400 MHz, $C_6D_6$, RT): δ [ppm] =** 7.13 (*p*t, *J* = 8.0, 7.6 Hz, 1H), 7.08 (dd, *J* = 2.6, 1.5 Hz, 1H), 6.98 (dd, *J* = 7.6, 1.5 Hz, 1H), 6.76 (dd, *J* = 8.0, 2.6 Hz, 1H), 5.54 – 5.47 (m, 1H), 4.41 (s, 2H), 3.98 (d, *J* = 6.5 Hz, 2H), 3.33 (s, 3H), 1.63 – 1.57 (m, 3H), 1.47 – 1.43 (m, 3H). **$^{13}$C-NMR (101 MHz, $C_6D_6$, RT): δ [ppm] =** 160.5, 141.3, 135.9, 129.6, 122.4, 120.0, 113.4, 113.2, 71.9, 66.9, 54.7, 25.7, 18.0. **IR:** 2913, 2854, 1598, 1586, 1489, 1454, 1436, 1263, 1153, 1070, 1051, 778, 743, 692 $cm^{-1}$. **HRMS (ESI+):** *m/z* calculated for $C_{13}H_{19}O_2^+$: 207.1380 u, found: 207.1379 u.

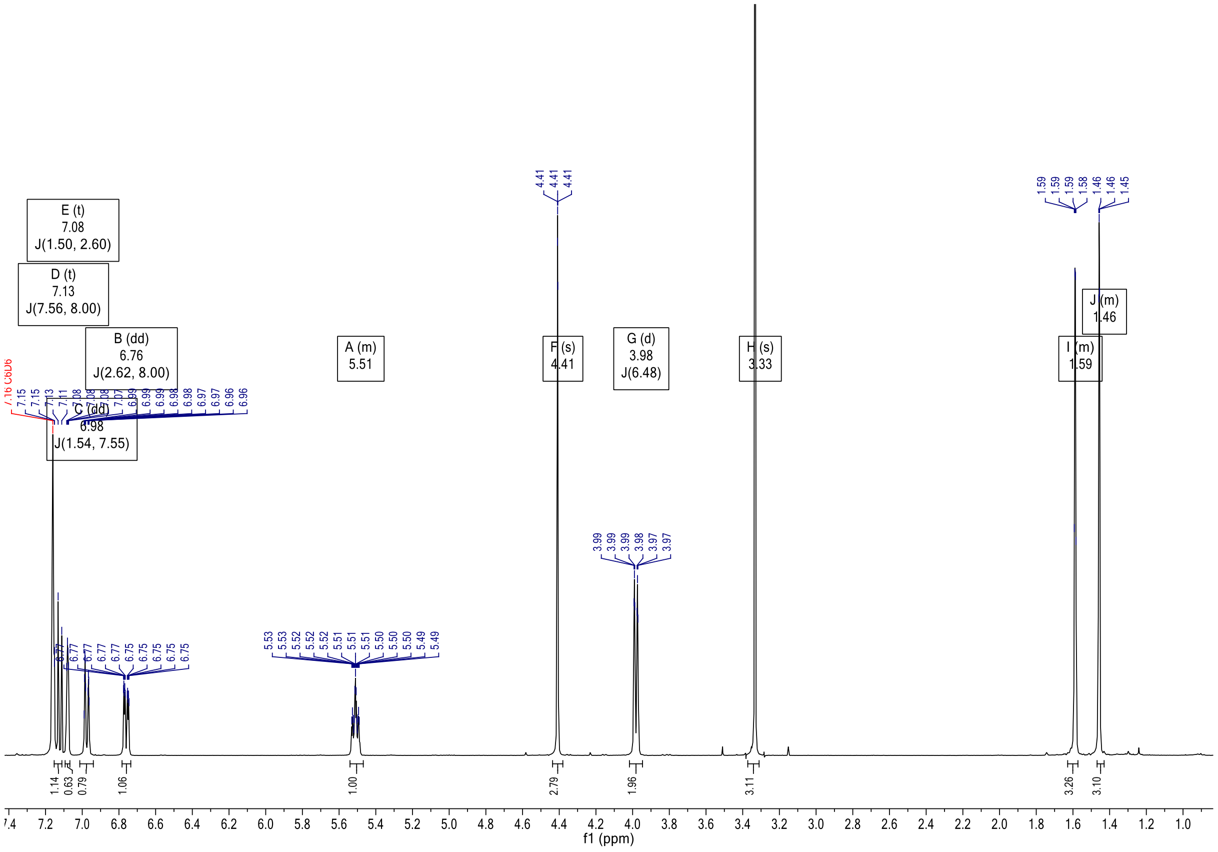
E (t)
7.08
J(1.50, 2.60)
D (t)
7.13
J(7.56, 8.00)
B (dd)
6.76
J(2.62, 8.00)
C (dd)
6.98
J(1.54, 7.55)
A (m)
5.51
F (s)
4.41
G (d)
3.98
J(6.48)
H (s)
3.33
I (m)
1.59
J (m)
1.46
f1 (ppm)

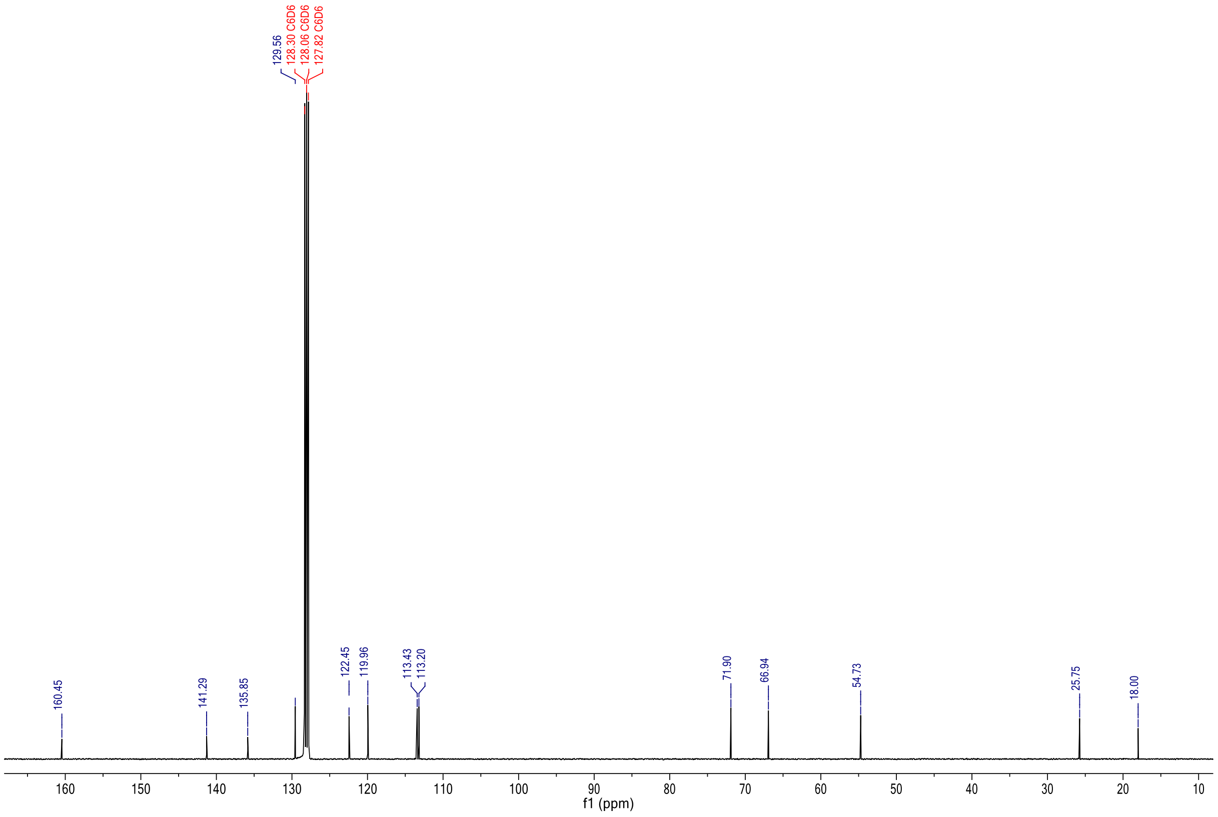
160.45
141.29
135.85
129.56
128.30 C6D6
128.06 C6D6
127.82 C6D6
122.45
119.96
113.43
113.20
71.90
66.94
54.73
25.75
18.00
f1 (ppm)

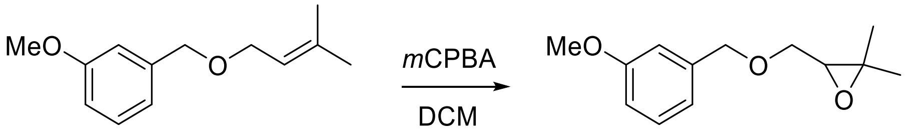


According to GP IV 2.40 g **1e-p** (11.6 mmol) are reacted with 4.30 g *m*CPBA (17.4 mmol). Column chromatography ($SiO_2$, eluent: CH:EA, 95:5) afforded 1.92 g **1e** (8.61 mmol, 74%) as a colourless oil.

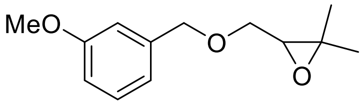


**1e**
$C_{13}H_{18}O_3$
222,28 g/mol

**$R_f$ =** 0.57 (30% EA, 70% CH). **$^1$H-NMR (300 MHz, $C_6D_6$, RT): δ [ppm] =** 7.15 – 7.09 (m, 1H), 7.06 – 6.99 (m, 1H), 6.98 – 6.88 (m, 1H), 6.75 (dd, *J* = 8.2, 2.7 Hz, 1H), 4.44 (d, *J* = 12.2 Hz, 1H), 4.33 (d, *J* = 12.2 Hz, 1H), 3.48 (dd, *J* = 11.0, 5.1 Hz, 1H), 3.43 (dd, *J* = 11.0, 5.5 Hz, 1H), 3.34 (s, 3H), 2.91 (*p*t, *J* = 5.3 Hz, 1H), 1.06 (s, 3H), 1.00 (s, 3H). **$^{13}$C-NMR (75 MHz, $C_6D_6$, RT): δ [ppm] =** 160.5, 140.6, 129.6, 120.0, 113.6, 113.4, 73.1, 69.4, 62.0, 56.8, 54.8, 24.7, 18.9. **IR:** 2961, 2837, 1598, 1586, 1489, 1455, 1435, 1379, 1265, 1154, 1080, 1049, 995, 865, 781, 692 $cm^{-1}$. **HRMS (ESI+):** *m/z* calculated for $C_{13}H_{19}O_3^+$: 223.1329 u, found: 223.1329 u.

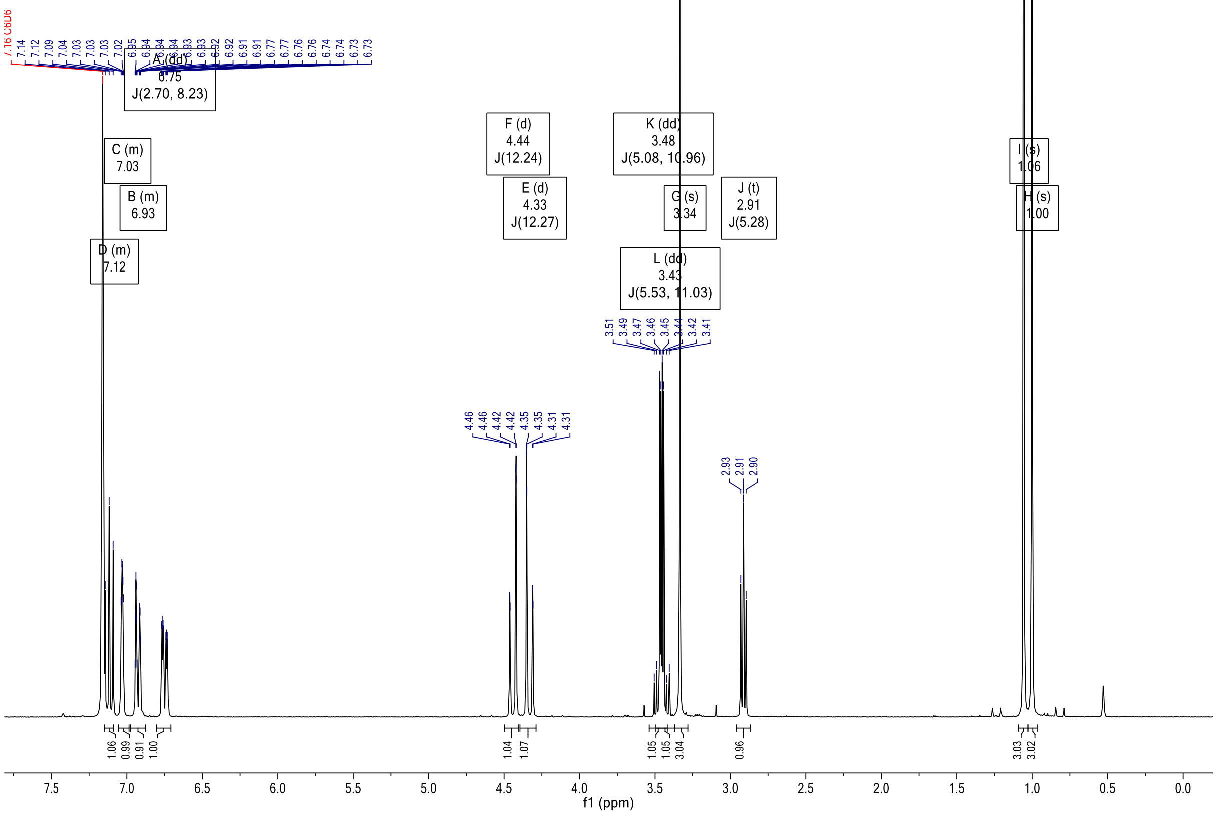
A (dd)
6.75
J(2.70, 8.23)
C (m)
7.03
B (m)
6.93
D (m)
7.12
F (d)
4.44
J(12.24)
E (d)
4.33
J(12.27)
K (dd)
3.48
J(5.08, 10.96)
G (s)
3.34
J (t)
2.91
J(5.28)
L (dd)
3.43
J(5.53, 11.03)
I (s)
1.06
H (s)
1.00
f1 (ppm)

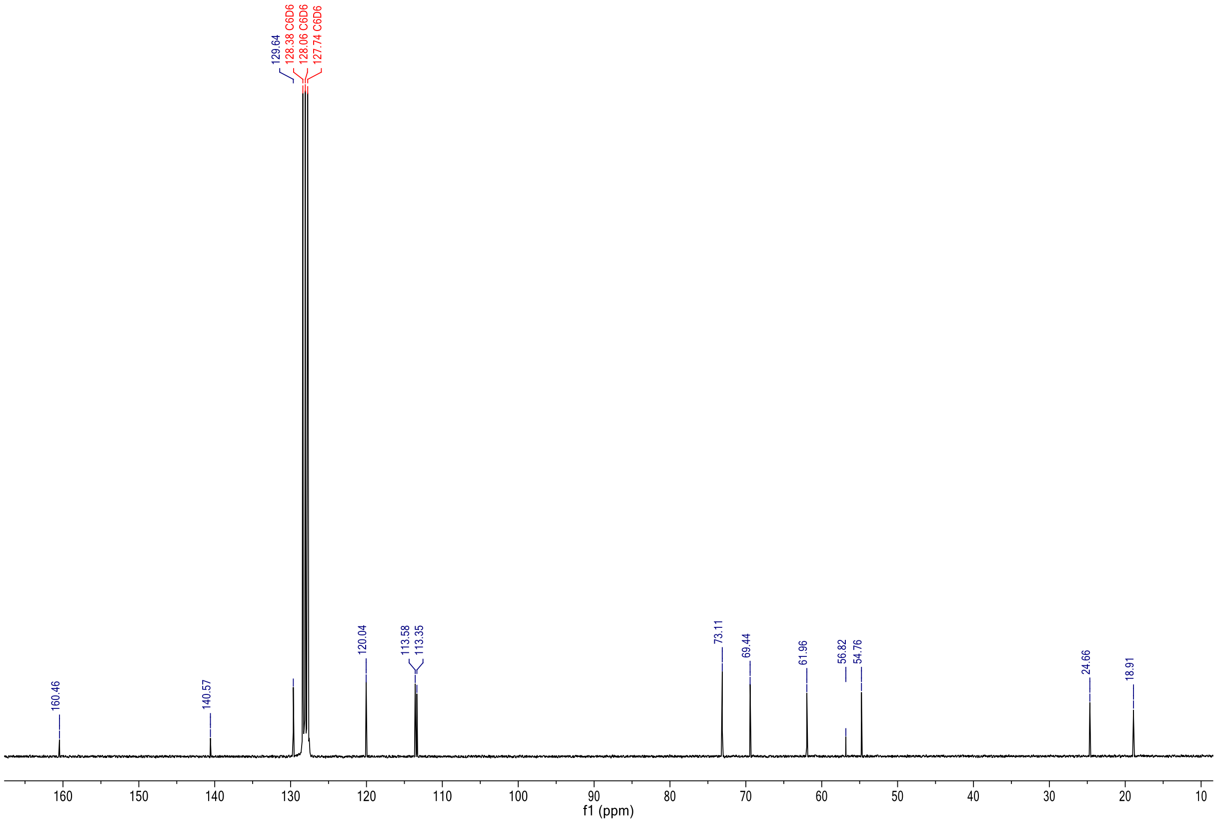
129.64
128.38 C6D6
128.06 C6D6
127.74 C6D6
160.46
140.57
120.04
113.58
113.35
73.11
69.44
61.96
56.82
54.76
24.66
18.91
f1 (ppm)

### 3.1.7 Synthesis of 2,2-dimethyl-3-{[(3,4,5-trimethoxyphenyl)methoxy]methyl}oxirane **1f**

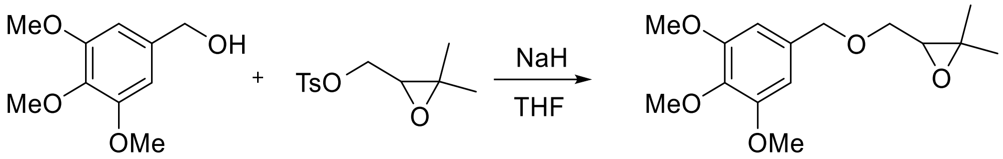


433 mg 3,4,5-Trimethoxybenzyl alcohol (2.18 mmol, 1.1 eq.) and 509 mg 2,2-Dimethyl-3-[(tosyloxy)methyl]oxirane (2.00 mmol, 1.0 eq.) are dissolved in 7 ml THF. 111 mg NaH (60% dispersion in mineral oil, 2.78 mmol, 1.4 eq.) is added slowly at 0 °C. The reaction mixture is stirred for 4 h at 60 °C. After cooling to room temperature, the mixture is quenched with $H_2O$. The phases are separated and the aqueous layer is extracted three times with DCM. The combined organic extracts are washed three times with $H_2O$ and once with brine. The solvent is dried over $MgSO_4$ and removed under reduced pressure. The crude product is purified by column chromatography ($SiO_2$, eluent: CH:EA, 80:20) and 242 mg **1f** (43%, 0.855 mmol) of the product is isolated as a colourless oil.

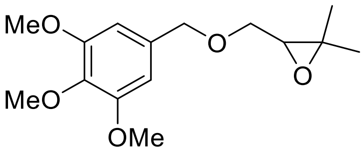


**1f**
$C_{15}H_{22}O_5$
282,34 g/mol

**$R_f$ =** 0.29 (30% EA, 70% CH). **$^1$H-NMR (300 MHz, $C_6D_6$, RT): δ [ppm] =** 6.59 (s, 2H), 4.48 (d, *J* = 12.0 Hz, 1H), 4.38 (d, *J* = 12.0 Hz, 1H), 3.85 (s, 3H), 3.57 (dd, *J* = 11.1, 4.5 Hz, 1H), 3.49 (dd, *J* = 11.1, 6.0 Hz, 1H), 3.43 (s, 6H), 2.97 (dd, *J* = 6.0, 4.5 Hz, 1H), 1.08 (s, 3H), 1.03 (s, 3H).**$^{13}$C-NMR (75 MHz, $C_6D_6$, RT): δ [ppm] =** 154.3, 139.1, 134.1, 105.6, 73.4, 69.4, 62.1, 60.5, 56.8, 55.9, 24.7, 19.0. **IR:** 2938, 1591, 1506, 1457, 1421, 1379, 1330, 1233, 1149, 1123, 1006, 864, 826, 780, 680 $cm^{-1}$. **HRMS (ESI+):** *m/z* calculated for $C_{15}H_{23}O_5^+$: 283.1540 u, found: 283.1538 u.

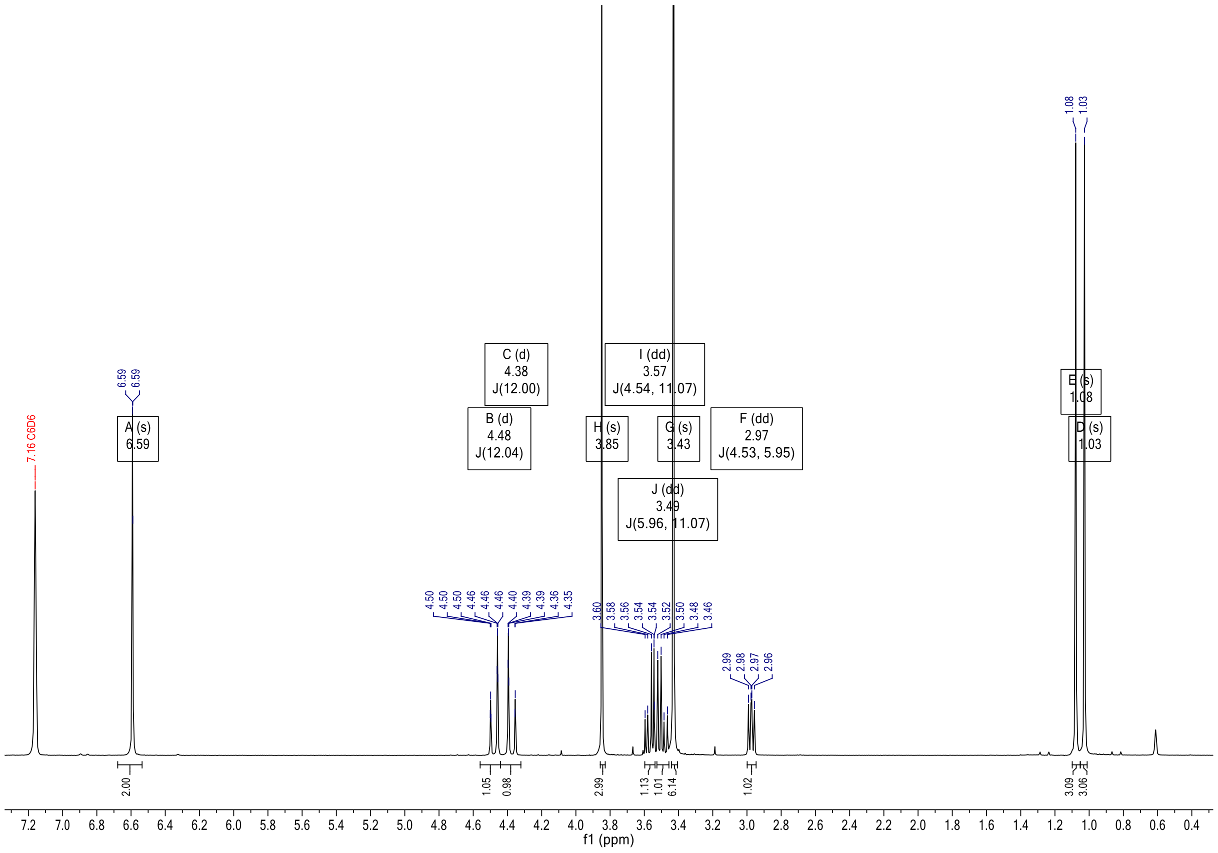
7.16 C6D6
6.59
6.59
A (s)
6.59
C (d)
4.38
J(12.00)
B (d)
4.48
J(12.04)
H (s)
3.85
I (dd)
3.57
J(4.54, 11.07)
G (s)
3.43
J (dd)
3.49
J(5.96, 11.07)
F (dd)
2.97
J(4.53, 5.95)
E (s)
1.08
D (s)
1.03
1.08
1.03
2.00
1.05
0.98
2.99
1.13
1.01
6.14
1.02
3.09
3.06
f1 (ppm)

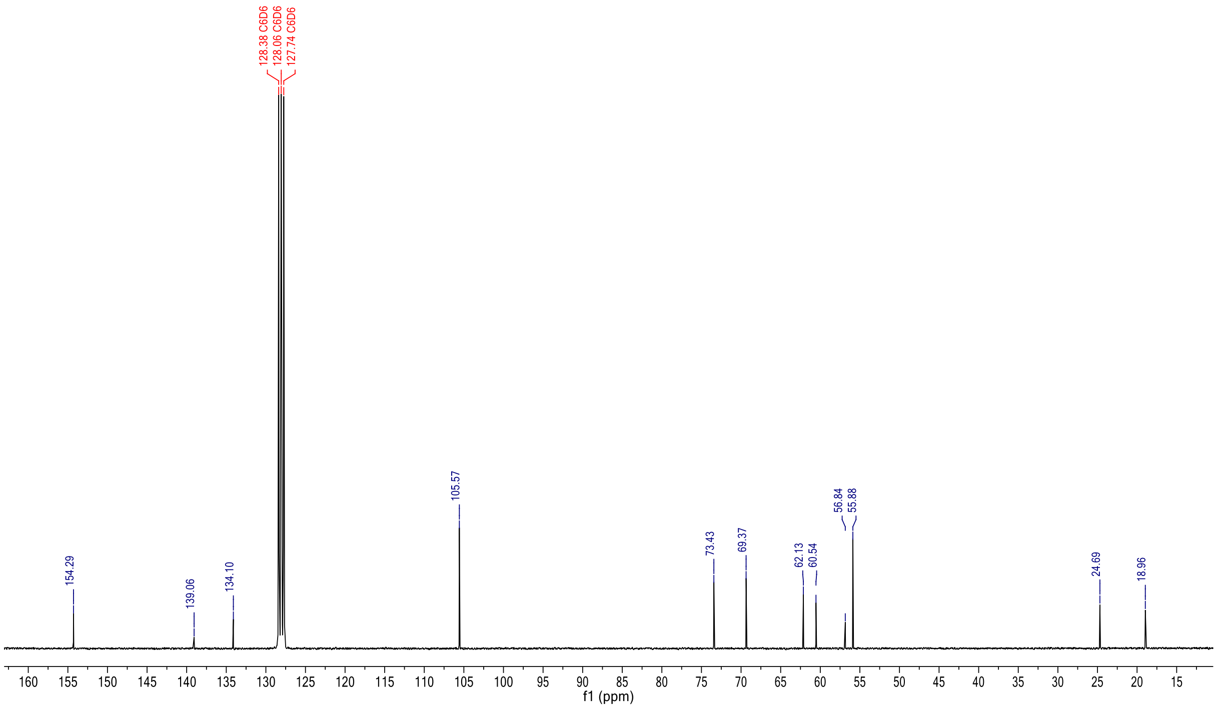
128.38 C6D6
128.06 C6D6
127.74 C6D6
154.29
139.06
134.10
105.57
73.43
69.37
62.13
60.54
56.84
55.88
24.69
18.96
f1 (ppm)

### 3.1.8 Synthesis of 2-(bromomethyl)-1-oxaspiro[2.5]octane **1g-p1**

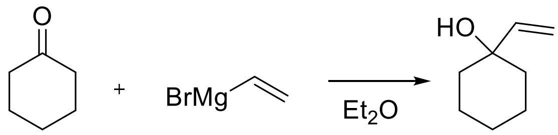


The reaction is performed in accordance with a literature procedure.[6] Note: this reaction was attempted with an “old” bottle of cyclohexanone, and significant amounts of byproducts/decomposition products were noted, therefore we recommend distillation of any “old” samples of cyclohexanone prior to conducting this procedure. 4.5 ml cyclohexanone (43.0 mmol, 1.0 eq.) is dissolved in $Et_2O$. 52 ml vinylmagnesium bromide (1 M in THF, 52 mmol, 1.2 eq.) is added dropwise at 0 °C. The mixture is stirred at 0 °C for 2 h and then at room temperature for 21 h and quenched with 40 ml saturated $NH_4Cl$ solution and diluted with 75 ml $Et_2O$. The phases are separated and aqueous layer is extracted with 75 ml $Et_2O$. The organic solvent is dried over $MgSO_4$ and removed under reduced pressure (110 mbar, 40 °C). The crude product is used without further purification.

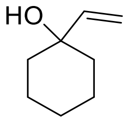


**1g-p2**
$C_8H_{14}O$
126,20 g/mol

The analytical data is in accordance with literature.[6]

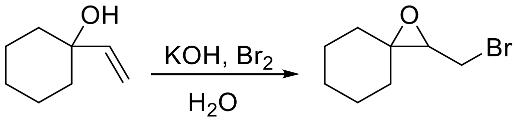


The procedure is in accordance with literature.[5] To a stirred solution of 12.6 g KOH (85%, 190 mmol, 4.5 eq.) in 36 ml $H_2O$ is slowly added 3.3 ml bromine (63.5 mmol, 1.5 eq.) at 5 °C via a dropping funnel. The mixture is stirred for 10 min at the same temperature, and then to this solution is added 6.5 ml **1g-p2** (42.3 mmol, 1.0 eq.) at 10 °C and stirred for 70 h at room temperature. The mixture is diluted with 60 ml $Et_2O$ and the organic layer is separated. The aqueous layer is extracted twice with CH and the combined organic extracts are washed with brine and dried over $MgSO_4$. The solvent is removed under reduced pressure (90 mbar, 40 °C). Column chromatography ($SiO_2$, eluent: pentane:$Et_2O$, 80:20) afforded 833 mg **1g-p1** (4.06 mmol, 10%) as a colourless oil.

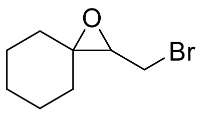

**1g-p1**
$C_8H_{13}BrO$
205,10 g/mol

Analytical data is in accordance with literature.[7]

3.1.9 Synthesis of 2-{[(4-methylphenyl)methoxy]methyl}-1-oxaspiro[2.5]octane **1g**

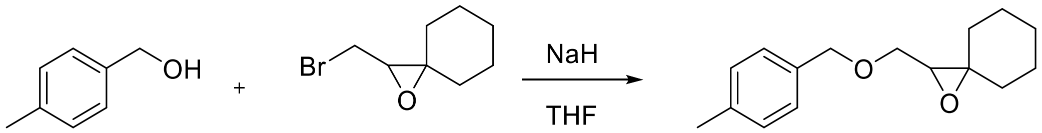


According to GP I 0.401 g 4-methylbenzyl alcohol **1b-p** (3.28 mmol, 1.1 eq.), 0.612 g 2-(bromomethyl)-1-oxaspiro[2.5]octane **1g-p1** (2.98 mmol, 1.0 eq.) and 100 mg NaH (60% dispersion in mineral oil, 4.18 mmol, 1.4 eq.) are reacted in THF for 24 h. Column chromatography ($SiO_2$, eluent: CH:EA, 95:5) afforded 311 mg **1g** (1.26 mmol, 42%) as a light yellow oil.

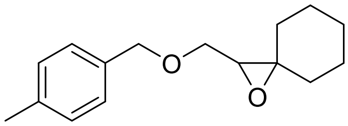

**1g**
$C_{16}H_{22}O_2$
246,35 g/mol

**R$_f$ =** 0.24 (5% EA, 95% CH). **$^1$H-NMR (500 MHz, $C_6D_6$, RT): δ [ppm] =** 7.24 (d, *J* = 7.9 Hz, 2H), 7.01 (d, *J* = 7.9 Hz, 2H), 4.46 (d, *J* = 11.9 Hz, 1H), 4.35 (d, *J* = 11.9 Hz, 1H), 3.53 (dd, *J* = 10.9, 5.0 Hz, 1H), 3.49 (dd, *J* = 10.9, 5.7 Hz, 1H), 2.96 (*p*t, *J* = 5.3 Hz, 1H), 2.11 (s, 3H), 1.64 – 1.52 (m, 2H), 1.42 – 1.30 (m, 6H), 1.28 – 1.21 (m, 2H). **$^{13}$C-NMR (125 MHz, $C_6D_6$, RT): δ [ppm] =** 137.2, 136.0, 129.3, 128.1, 73.2, 68.8, 62.3, 61.5, 35.7, 29.9, 26.0, 25.2, 25.2, 21.2. **IR:** 2930, 2856, 1447, 1098, 1021, 915, 896, 845, 801, 752, 675, 572, 550, 476, 450 $cm^{-1}$. **HRMS (APCI):** *m/z* calculated for $C_{16}H_{23}O_2^+$: 247.1693 u, found: 247.1693 u.

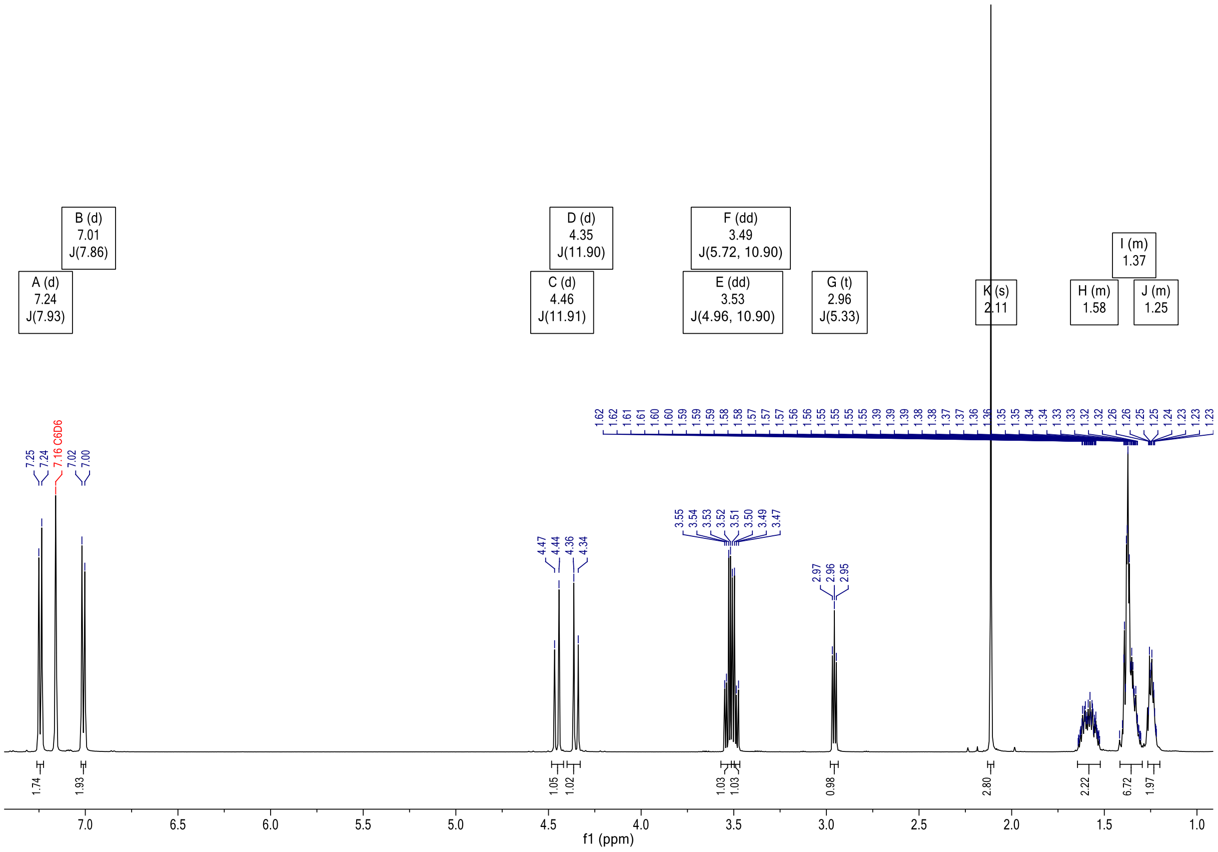

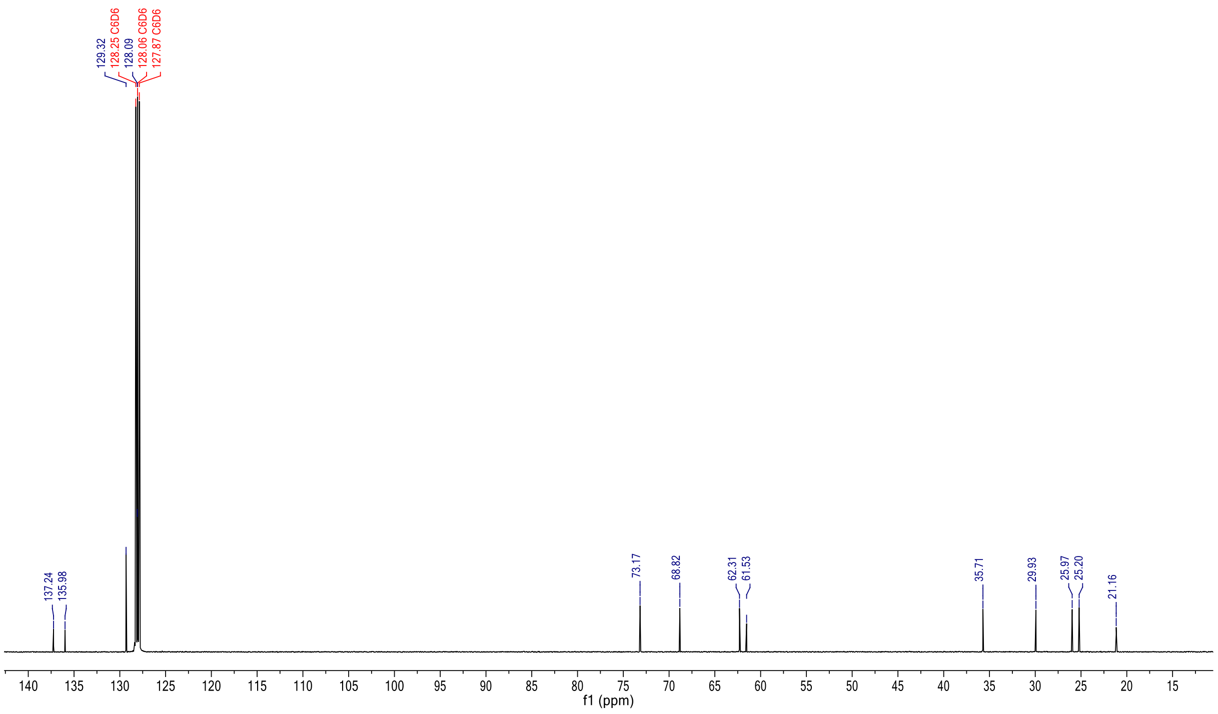

3.1.10 Synthesis of 2-[(benzyloxy)methyl]-1-oxaspiro[2.5]octane **1h**

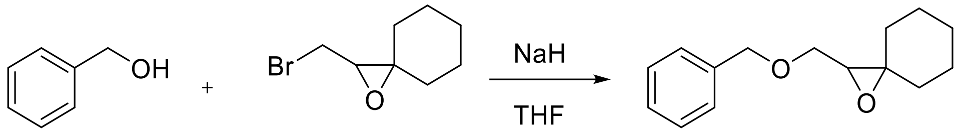


According to GP I 0. 460 g benzyl alcohol (4.17 mmol, 1.1 eq.), 0.777 g 2-(bromomethyl)-1-oxaspiro[2.5]octane (3.79 mmol, 1.0 eq.) and 368 mg NaH (60% dispersion in mineral oil, 9.20 mmol, 2.4 eq.) are reacted in THF for 22 h. Column chromatography ($SiO_2$, eluent: CH:EA, 95:5) afforded 741 mg **1h** (3.19 mmol, 84%) as a light yellow oil.

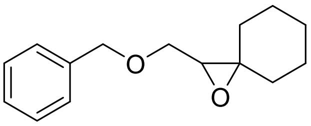


**1h**
$C_{15}H_{20}O_2$
232,32 g/mol

**$R_f$ =** 0.42 (10% EA, 90% CH). **$^1$H-NMR (500 MHz, $C_6D_6$, RT): δ [ppm] =** 7.32 – 7.27 (m, 2H), 7.20 – 7.14 (m, 2H), 7.12 – 7.07 (m, 1H), 4.43 (d, *J* = 12.1 Hz, 1H), 4.32 (d, *J* = 12.1 Hz, 1H), 3.51 (dd, *J* = 10.9, 4.9 Hz, 1H), 3.46 (dd, *J* = 10.9, 5.8 Hz, 1H), 2.94 (dd, *J* = 5.8, 4.9 Hz, 1H), 1.65 – 1.51 (m, 2H), 1.41 – 1.29 (m, 6H), 1.27 – 1.21 (m, 2H). **$^{13}$C-NMR (125 MHz, $C_6D_6$, RT): δ [ppm] =** 139.0, 128.6, 127.9, 127.8, 73.2, 69.0, 62.3, 61.5, 35.7, 29.9, 26.0, 25.2, 25.2. **IR:** 2930, 2855, 1452, 1361, 1191, 1093, 896, 851, 789, 735, 700, 675, 603, 551, 500 $cm^{-1}$. **HRMS (APCI):** *m/z* calculated for $C_{15}H_{21}O_2^+$: 233.1536 u, found: 233.1526 u.

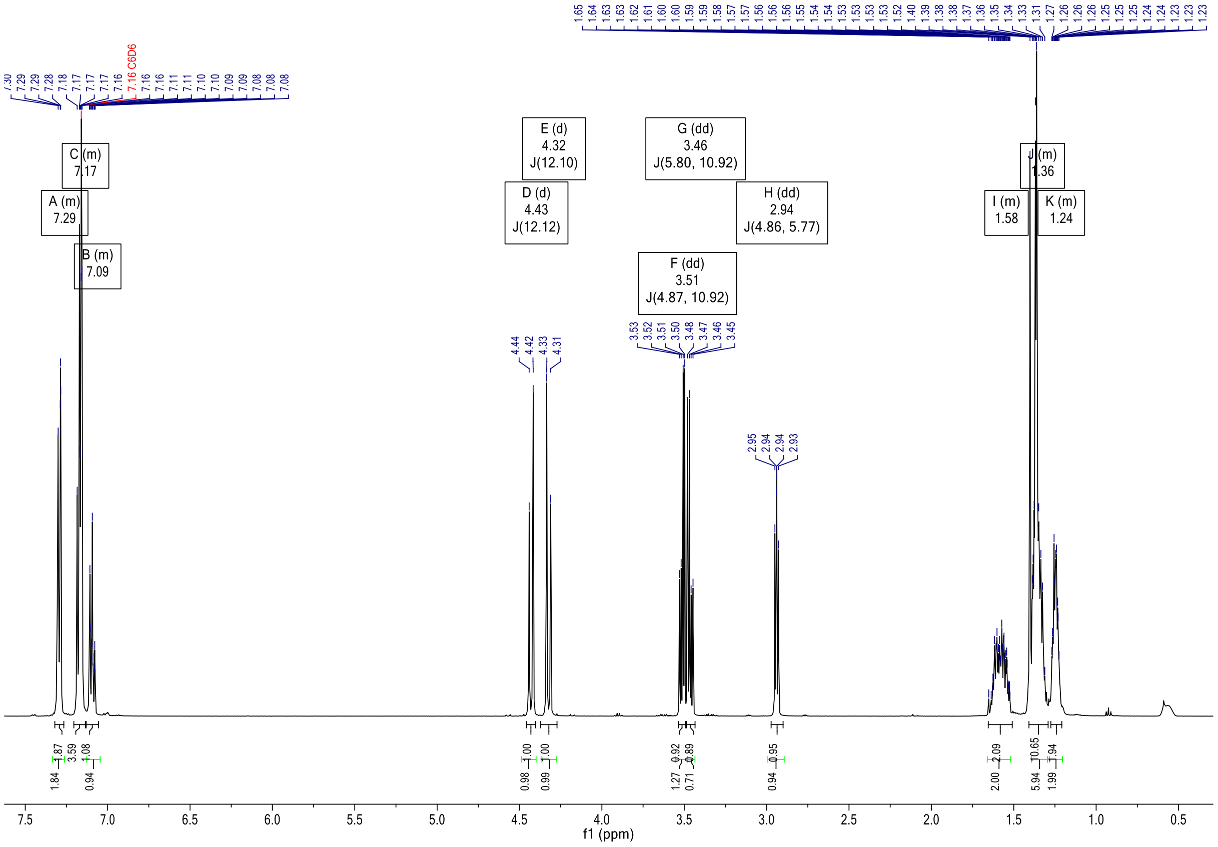
A (m) 7.29
B (m) 7.09
C (m) 7.17
D (d) 4.43 J(12.12)
E (d) 4.32 J(12.10)
F (dd) 3.51 J(4.87, 10.92)
G (dd) 3.46 J(5.80, 10.92)
H (dd) 2.94 J(4.86, 5.77)
I (m) 1.58
J (m) 1.36
K (m) 1.24
7.16 C6D6
f1 (ppm)

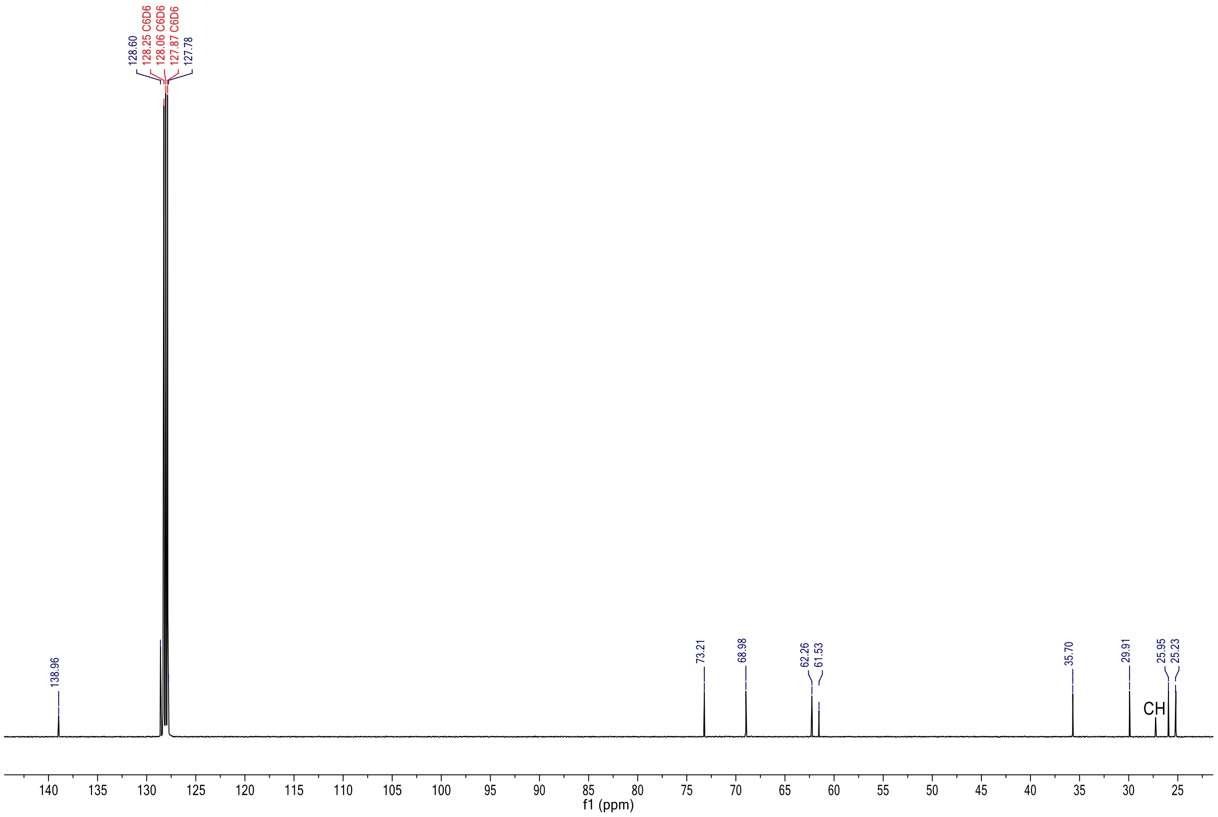
138.96
128.60
128.25 C6D6
128.06 C6D6
127.87 C6D6
127.78
73.21
68.98
62.26
61.53
35.70
29.91
25.95
25.23
CH
f1 (ppm)

3.1.11 Synthesis of 2,2-dimethyl-3-{[(4-chlorophenyl)methoxy]methyl}oxirane **1i**

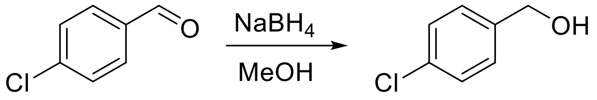


According to GP II 2.07 g 4-chlorobenzaldehyde (14.7 mmol, 1.00 eq.) and 0.560 g $NaBH_4$ (14.9 mmol, 1.01 eq.) are reacted for 2 h. EA is used for extraction. Workup yielded 2.70 g **1i-p** (14.7 mmol, 100%) as a white solid, which is used without further purification.

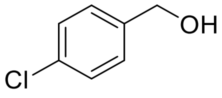


**1i-p**
$C_7H_7ClO$
142,58 g/mol

Analytical data is in accordance with literature.[8]

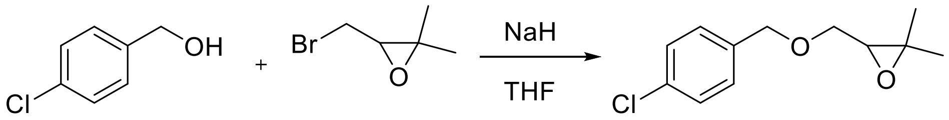


According to GP I 2.70 g 4-chlorobenzyl alcohol **1i-p** (18.9 mmol, 1.1 eq.), 2.84 g 1-bromo-3-methyl-2,3-epoxybutane (17.2 mmol, 1.0 eq.) and 960 mg NaH (60% dispersion in mineral oil, 24.1 mmol, 1.4 eq.) are reacted in THF for 3 d. Column chromatography ($SiO_2$, eluent: CH:EA, 95:5) afforded 1.40 g **1i** (6.16 mmol, 36%) as a light yellow oil.

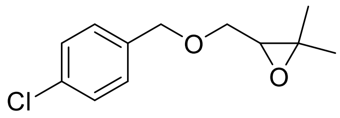


**1i**
$C_{12}H_{15}ClO_2$
226,70 g/mol

**$R_f$ =** 0.43 (20% EA, 80% CH). **$^1$H-NMR (300 MHz, $C_6D_6$, RT): δ [ppm] =** 7.11 (d, *J* = 8.4 Hz, 2H), 6.97 (d, *J* = 8.5 Hz, 2H), 4.23 (d, *J* = 12.3 Hz, 1H), 4.12 (d, *J* = 12.3 Hz, 1H), 3.39 (dd, *J* = 11.1, 4.6 Hz, 1H), 3.32 (dd, *J* = 11.1, 5.9 Hz, 1H), 2.85 (dd, *J* = 5.9, 4.6 Hz, 1H), 1.06 (s, 3H), 0.99 (s, 3H). **$^{13}$C-NMR (75 MHz, $C_6D_6$, RT): δ [ppm] =** 137.3, 133.6, 129.1, 128.7, 72.2, 69.5, 61.9, 56.8, 24.6, 18.9. **IR:** 2963, 1491, 1456, 1409, 1379, 1085, 1015, 866, 842, 800, 678, 487 $cm^{-1}$. **HRMS(ESI+):** *m/z* calculated for $C_{12}H_{15}ClO_2Na^+$: 249.0663 u, found: 249.0657 u.

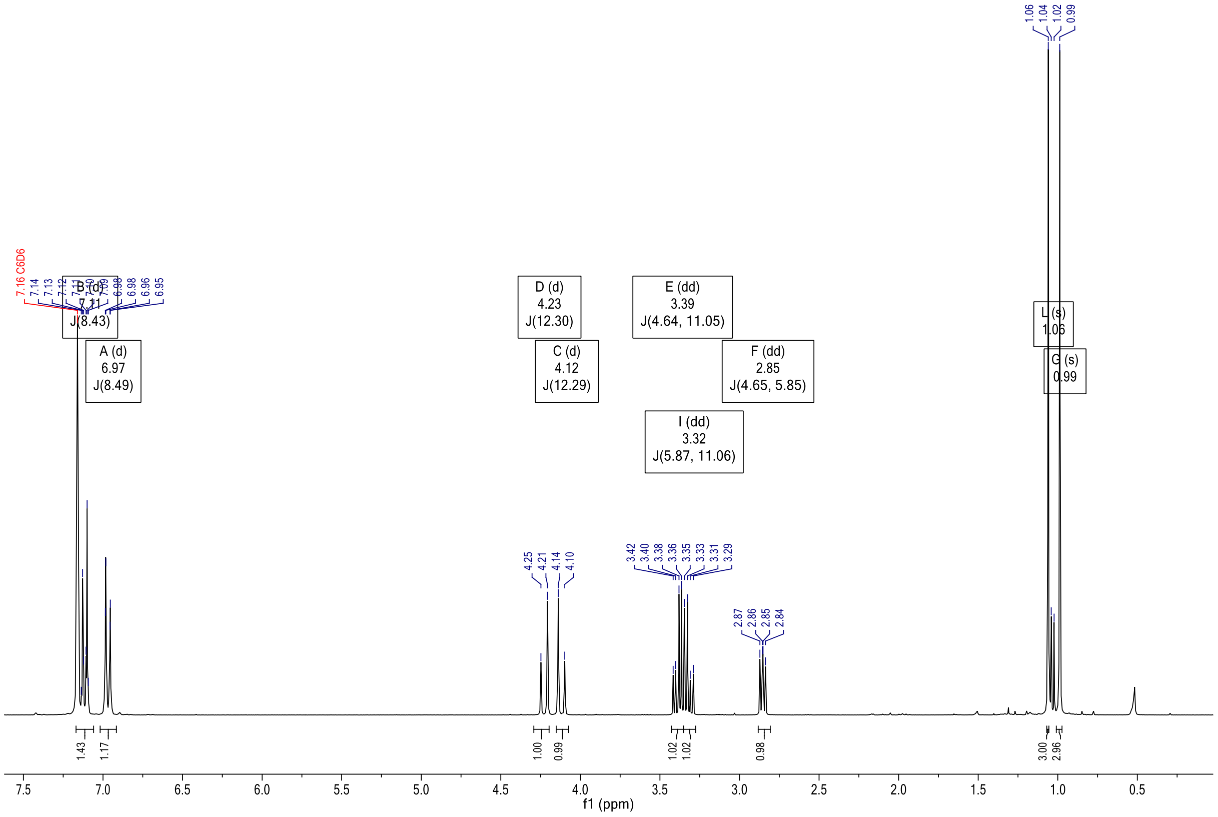
7.16 C6D6
B (d)
7.11
J(8.43)
A (d)
6.97
J(8.49)
D (d)
4.23
J(12.30)
C (d)
4.12
J(12.29)
E (dd)
3.39
J(4.64, 11.05)
I (dd)
3.32
J(5.87, 11.06)
F (dd)
2.85
J(4.65, 5.85)
L (s)
1.06
G (s)
0.99
f1 (ppm)

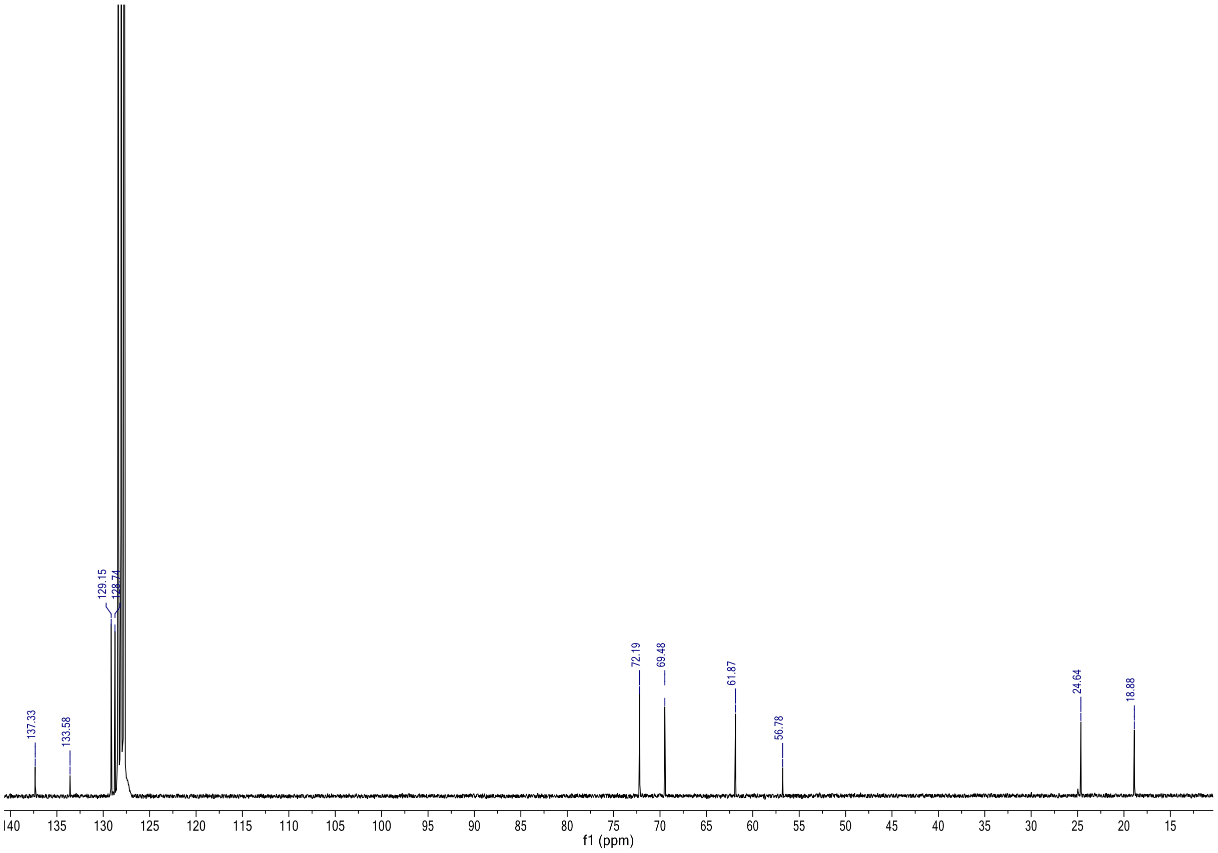
137.33
133.58
129.15
128.74
72.19
69.48
61.87
56.78
24.64
18.88
f1 (ppm)

## 3.2 α-Methylbenzyl-glycidyl ether synthesis **3a-3e**

### 3.2.1 Synthesis of 3-{[(2,3-dihydro-1*H*-inden-1-yl)oxy]methyl}-2,2-dimethyloxirane **3a**

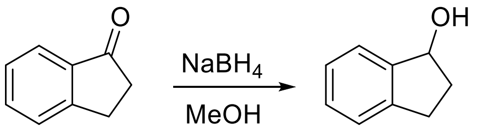


According to GP II 2.00 g 1-indanone (15.1 mmol, 1.00 eq.) and 687 mg $NaBH_4$ (18.2 mmol, 1.2 eq.) are reacted for 23 h in 75 ml MeOH (0.2 mmol/ml). EA is used for extraction. Work up yielded 1.94 g **3a-p** (14.4 mmol, 96%) as a colourless oil, which is used without further purification.

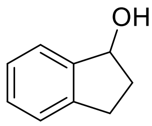


**3a-p**
$C_9H_{10}O$
134.18 g/mol

Analytical data is in accordance with literature.[9]

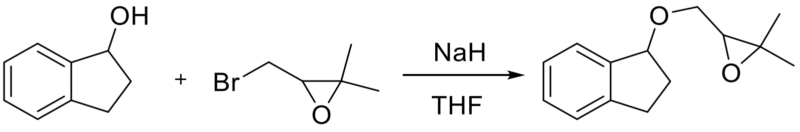


According to GP I 1.92 g 1-indanol **3a-p** (14.3 mmol, 1.0 eq.), 2.34 g 1-bromo-3-methyl-2,3-epoxybutane (14.3 mmol, 1.0 eq.) and 630 mg NaH (60% dispersion in mineral oil, 15.8 mmol, 1.1 eq.) are reacted for 12 h in THF. Column chromatography ($SiO_2$, eluent: CH:EA, 95:5) afforded 1.36 g **3a** (6.24 mmol, 44%) as a light yellow oil.

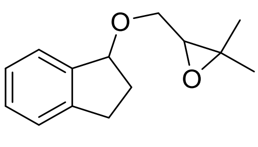


**3a**
$C_{14}H_{18}O_2$
218,30 g/mol

**$R_f$ =** 0.49 (20% EA, 80% CH). **Diastereomer a and b: $^1$H-NMR (300 MHz, $C_6D_6$, RT): δ [ppm] =** 7.47 – 7.39 (m, 1H), 7.14 – 7.05 (m, 3H), 4.79 (dd, *J* = 6.3, 4.9 Hz, 1H) and 4.75 (t, *J* = 5.2 Hz, 1H), 3.59 (dd, *J* = 10.7, 5.6 Hz, 1H) and 3.54 (d, *J* = 5.3 Hz, 2H) and 3.48 (dd, *J* = 10.6, 5.4 Hz, 1H), 3.03 – 2.78 (m, 2H), 2.60 – 2.47 (m, 1H), 2.10 – 1.86 (m, 2H), 1.08 (s, 3H), 1.07 (s, 3H) and 1.04 (s, 3H). **Diastereomer a: $^{13}$C-NMR (75 MHz, $C_6D_6$, RT): δ [ppm] =** 144.5, 143.2, 128.6, 126.4, 125.5, 125.0, 83.7, 67.7, 62.2, 57.0, 33.3, 30.5, 24.7, 18.9. **Diastereomer b: $^{13}$C-NMR (75 MHz, $C_6D_6$, RT): δ [ppm] =** 144.0, 143.4, 128.6, 126.6, 125.6, 125.2, 83.9, 67.9, 62.1, 57.1, 32.7, 30.4, 24.7, 18.9. **IR:** 2962, 1459, 1378, 1335, 1102, 1075, 1018, 866, 746, 678,

413 $cm^{-1}$. **HRMS (ESI+):** *m/z* calculated for $C_{14}H_{18}O_2Na^+$: 241.1199 u, found: 241.1198 u.

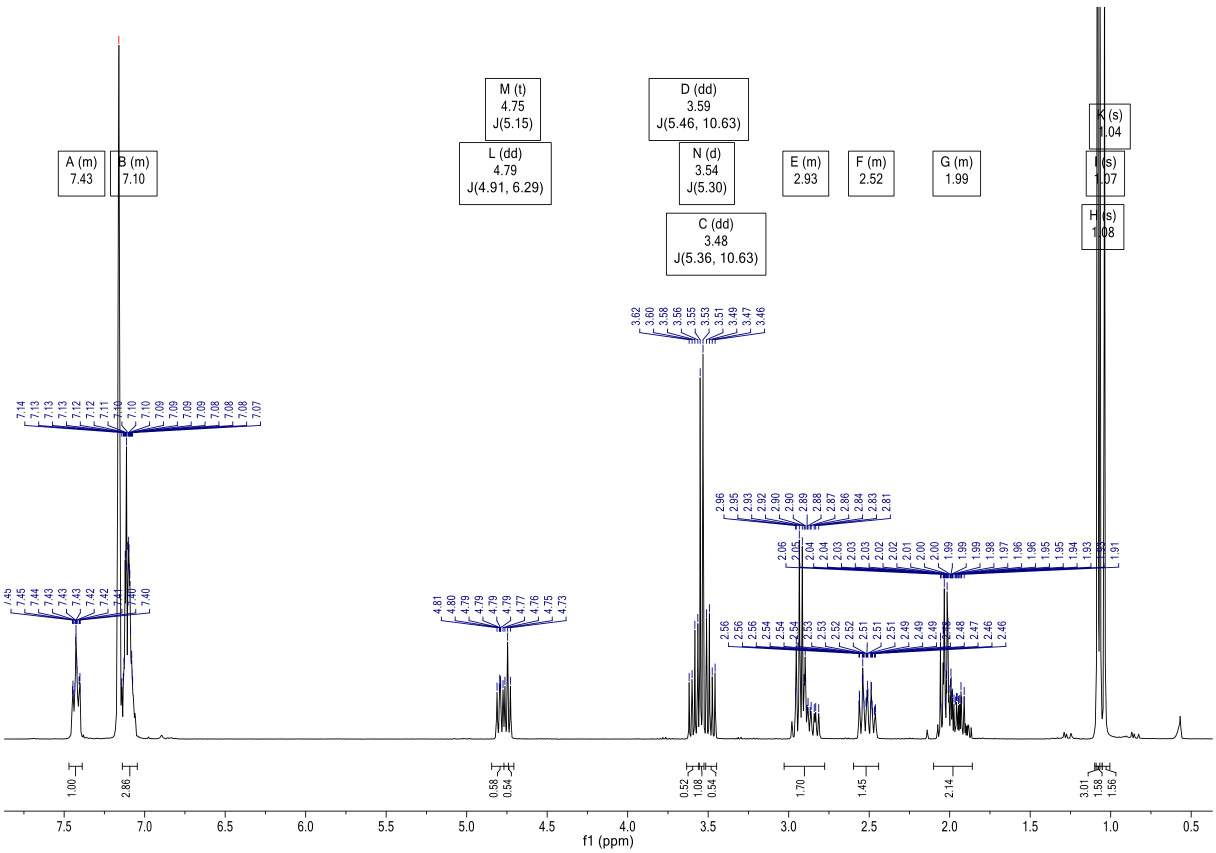

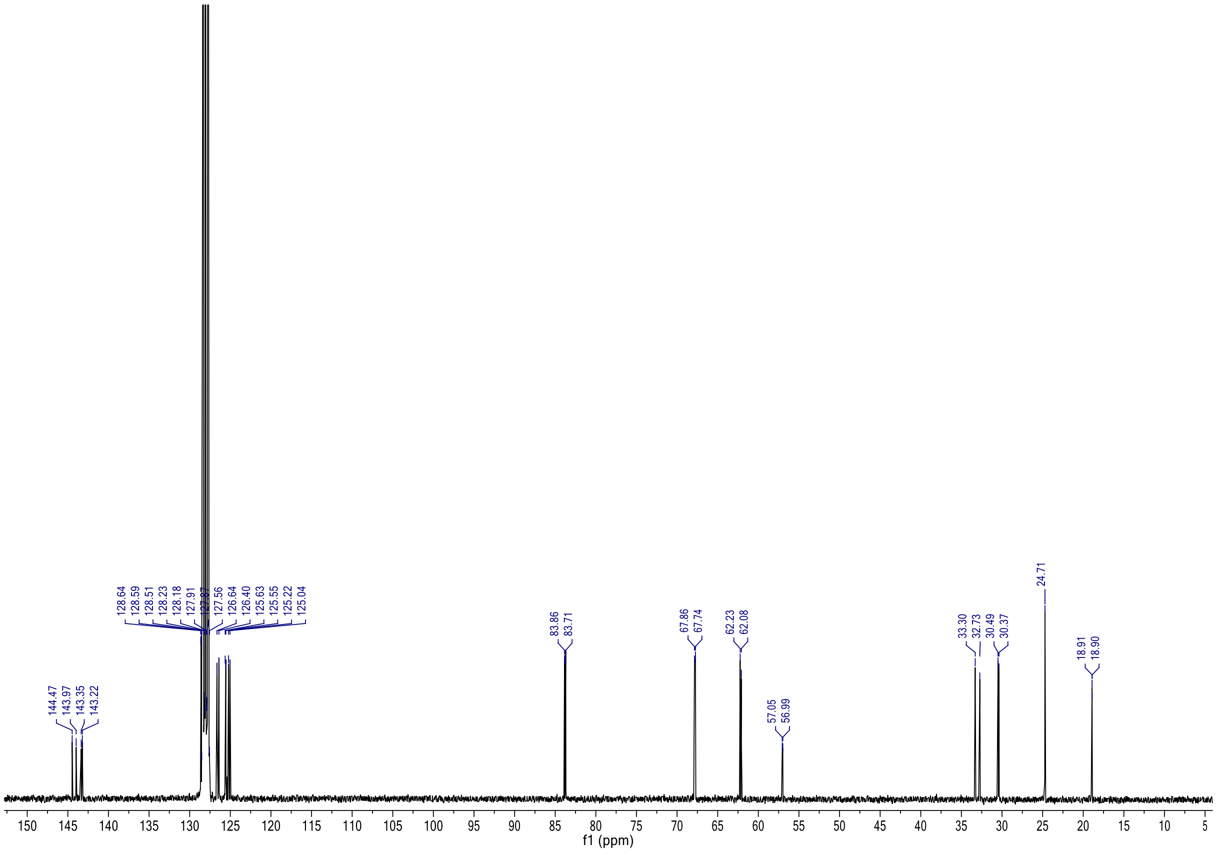

3.2.2 Synthesis of 2,2-dimethyl-3-{[(1,2,3,4-tetrahydronaphthalen-1-yl)oxy]methyl}-oxirane **3b**

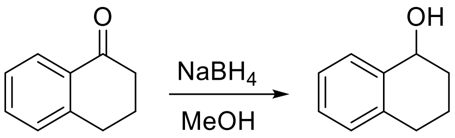


According to GP II 3.67 g 1-tetralon (25.0 mmol, 1.00 eq.) and 0.958 g $NaBH_4$ (25.3 mmol, 1.01 eq.) are reacted for 14 h in 30 ml MeOH (0.8 mmol/ml). EA is used for extraction. The mixture is washed with $H_2O$ once. Workup yielded 3.71 g 1-tetralol **3b-p** (25.0 mmol, 100%) as a light yellow oil, which is used without further purification.

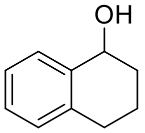


**3b-p**
$C_{10}H_{12}O$
148,21 g/mol

Analytical data is in accordance with literature.[10]

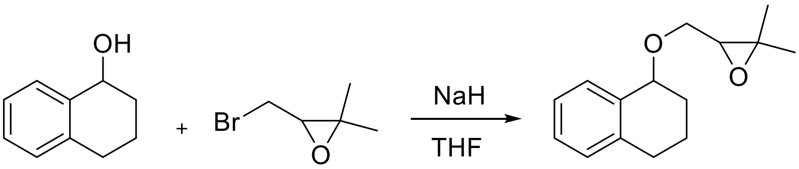


According to GP I 3.71 g 1-tetralol **3b-p** (25.1 mmol, 1.1 eq.), 3.76 g 1-bromo-3-methyl-2,3-epoxybutane (22.8 mmol, 1.0 eq.) and 1.28 g NaH (60% dispersion in mineral oil, 31.9 mmol, 1.4 eq.) are reacted for 17 h in THF. The mixture is washed with $H_2O$ during the workup. Column chromatography ($SiO_2$, eluent: CH:EA, 95:5) afforded 3.04 g **3b** (13.1 mmol, 52%) as a colourless oil.

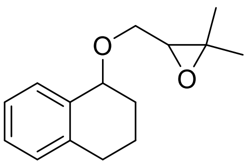


**3b**
$C_{15}H_{20}O_2$
232,32 g/mol

**$R_f$ =** 0.49 (20% EA, 80% CH). **Diastereomer a and b: $^1$H-NMR (500 MHz, $C_6D_6$, RT): δ [ppm]** = 7.52 – 7.46 (m, 1) and 7.44 – 7.36 (m, 1H), 7.12 – 7.04 (m, 2H), 6.97 – 6.92 (m, 1H), 4.34 – 4.31 (m, 1H) and 4.25 (*p*t, *J* = 4.7 Hz, 1H), 3.59 (dd, *J* = 10.8, 5.8 Hz, 1H) and 3.56 – 3.53 (m, 2H) and 3.49 (dd, *J* = 10.8, 4.9 Hz, 1H), 2.95 (*p*t, *J* = 5.4 Hz, 1H) and 2.93 (*p*t, *J* = 5.3 Hz, 1H), 2.70 – 2.55 (m, 1H), 2.52 – 2.36 (m, 1H), 2.04 – 1.74 (m, 2H), 1.67 – 1.56 (m, 1H), 1.53 – 1.37 (m, 1H), 1.09 (s, 3H), 1.08 (s, 3H) and 1.05 (s, 3H). **Diastereomer a: $^{13}$C-NMR (126 MHz, $C_6D_6$, RT): δ [ppm] =** 137.8, 137.6, 129.9, 129.1, 127.9, 125.8, 76.1, 67.8, 62.3, 57.0,

29.2, 28.8, 24.7, 19.3, 19.0. **Diastereomer b: $^{13}$C-NMR (101 MHz, $C_6D_6$, RT): δ [ppm] =** 137.3, 137.0, 129.8, 129.3, 127.7, 126.0, 76.1, 67.6, 62.3, 57.0, 29.4, 28.3, 24.7, 19.0, 18.9. **IR:** 2932, 2863, 1490, 1454, 1378, 1347, 1245, 1208, 1116, 1074, 901, 866, 769, 740, 680, 439, 420, 407 cm$^{-1}$. **HRMS (ESI+):** *m/z* calculated for $C_{15}H_{21}O_2^+$: 233.1536 u, found: 233.1534 u.

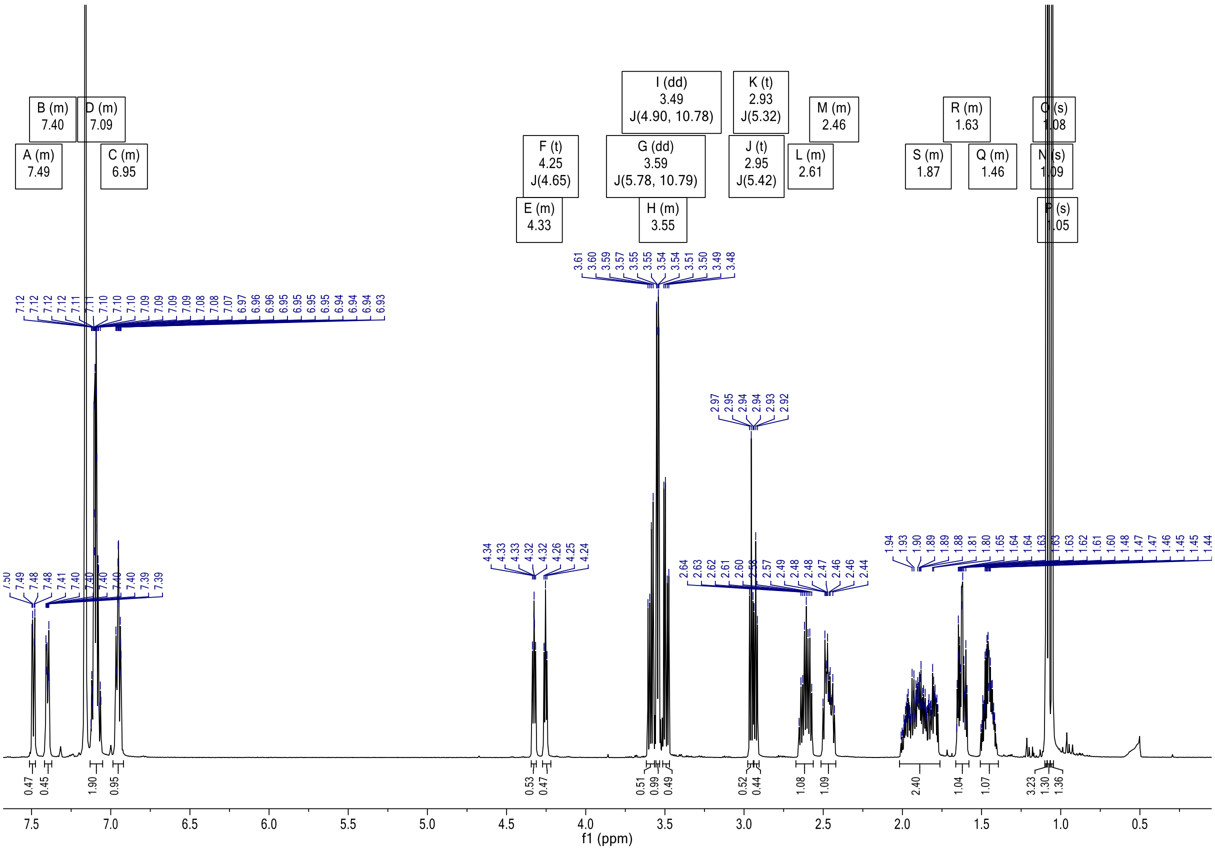

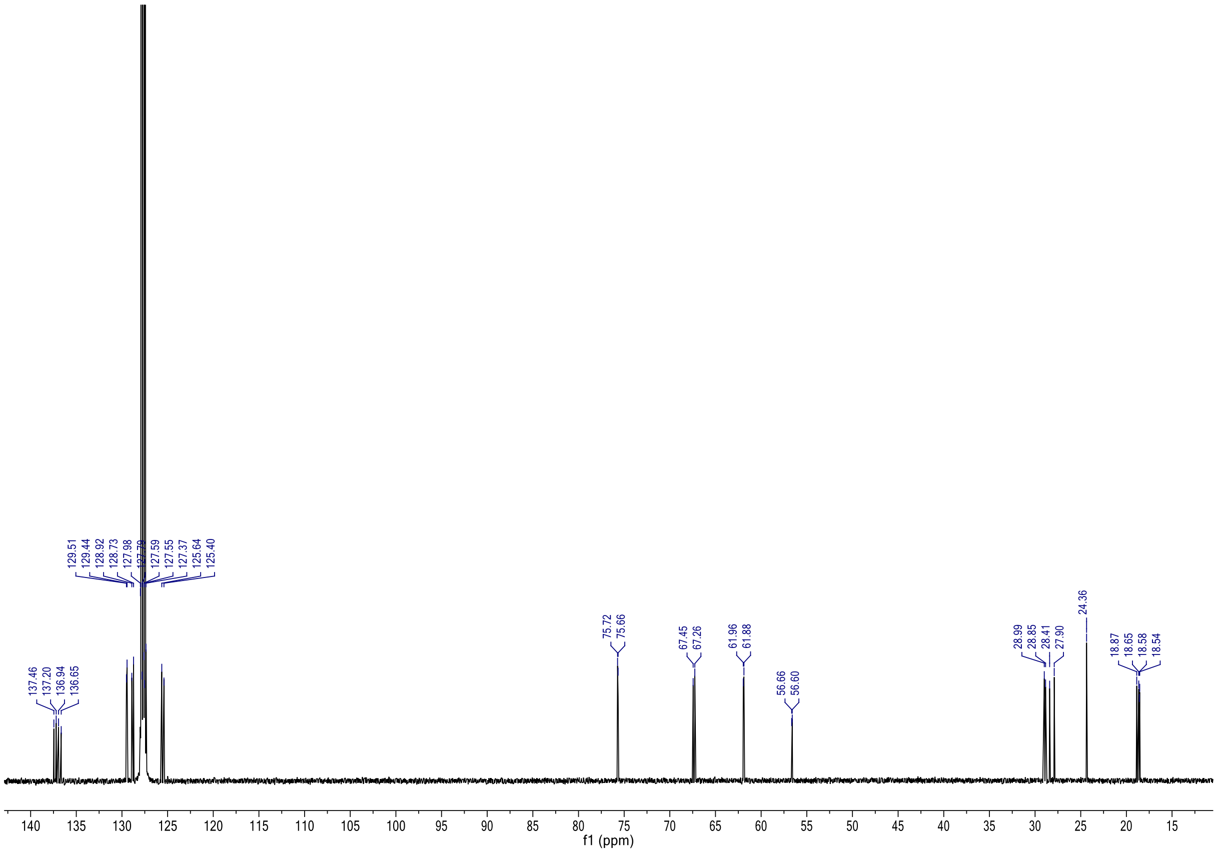


### 3.2.3 Synthesis of 2,2-dimethyl-3-[(1-phenylethoxy)methyl]oxirane **3c**

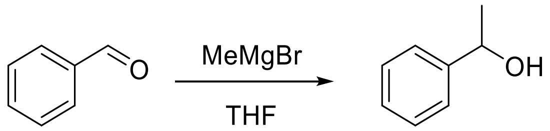


In a flame dried schlenk flask 15.5 ml Methylmagnesium bromide (3 M in $Et_2O$, 46.5 mmol, 1.2 eq.) is added dropwise to a solution of 3.98 g benzaldehyde (38.8 mmol, 1.0 eq.) in 95 ml anhydrous THF at 0 °C under argon. The resulting mixture is stirred 0.5 h at 0 °C and 4 h at room temperature and the reaction progress is monitored by TLC. After full consumption of the substrate the mixture is quenched with 20 ml saturated $NH_4Cl$ solution and the organic layer is separated. The aqueous layer was extracted three times with EA. The combined organic layers were washed with brine, dried over $MgSO_4$, and concentrated under reduced pressure (60 mbar, 40 °C). The 3.89 g crude product **3c-p** is isolated as a colourless oil and used without further purification.

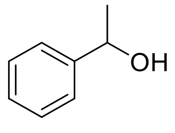

**3c-p**
$C_8H_{10}O$
122,17 g/mol

Analytical data is in accordance with literature.[1]

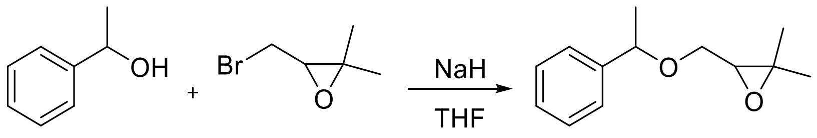

According to GP I 2.44 g α-methylbenzyl alcohol **3c-p** (20.0 mmol, 1.1 eq.), 3.00 g 1-bromo-3-methyl-2,3-epoxybutane (18.2 mmol, 1.0 eq.) and 1.02 g NaH (60% dispersion in mineral oil, 25.5 mmol, 1.4 eq.) are reacted for 23 h in THF. The reaction is quenched with 15 ml $H_2O$. Column chromatography ($SiO_2$, eluent: CH:EA, 95:5) afforded 1.56 g **3c** (7.56 mmol, 42%) as a light yellow oil.

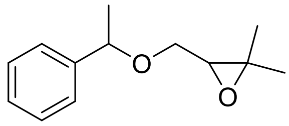

**3c**
$C_{13}H_{18}O_2$
206,29 g/mol

**$R_f$ =** 0.55 (20% EA, 80% CH). **Diastereomer a: $^1$H-NMR (300 MHz, $C_6D_6$, RT): δ [ppm] =** 7.29 – 7.22 (m, 2H), 7.20 – 7.13 (m, 2H), 7.12 – 7.04 (m, 1H), 4.33 – 4.19 (m, 1H), 3.46 – 3.33 (m, 2H), 2.93 (*p*t, *J* = 5.3 Hz, 1H), 1.40 (d, *J* = 6.3 Hz, 3H), 1.03 (s, 3H), 0.91 (s, 3H). **$^{13}$C-NMR (75 MHz, $C_6D_6$, RT): δ [ppm] =** 144.3, 128.7, 127.8, 126.6, 78.7, 68.0, 62.3, 56.8, 24.7, 24.6, 18.8. **Diastereomer b: $^1$H-NMR (300 MHz, $C_6D_6$, RT): δ [ppm] =** 7.29 – 7.22 (m, 2H), 7.20 – 7.13 (m, 2H), 7.12 – 7.04 (m, 1H), 4.33 – 4.19 (m, 1H), 3.47 (dd, *J* = 10.7, 5.6 Hz, 1H), 3.31 (dd, *J* = 10.7, 5.0 Hz, 1H), 2.90 (*p*t, *J* = 5.4 Hz, 1H), 1.38 (d, *J* = 6.3 Hz, 3H), 1.06 (s, 3H), 0.99 (s, 3H). **$^{13}$C-NMR (75 MHz, $C_6D_6$, RT): δ [ppm] =** 144.2, 128.8, 127.7 126.4, 78.6, 67.9, 61.9, 57.1, 24.7, 24.5, 18.9. **IR:** 2976, 2928, 2865, 1451, 1378, 1097, 1057, 1028913, 868, 809, 759, 700, 610, 562, 513, 413 $cm^{-1}$. **HRMS (ESI+):** *m/z* calculated for $C_{13}H_{19}O_2 + CH_3CN^+$: 248.1645 u, found: 248.1642 u.

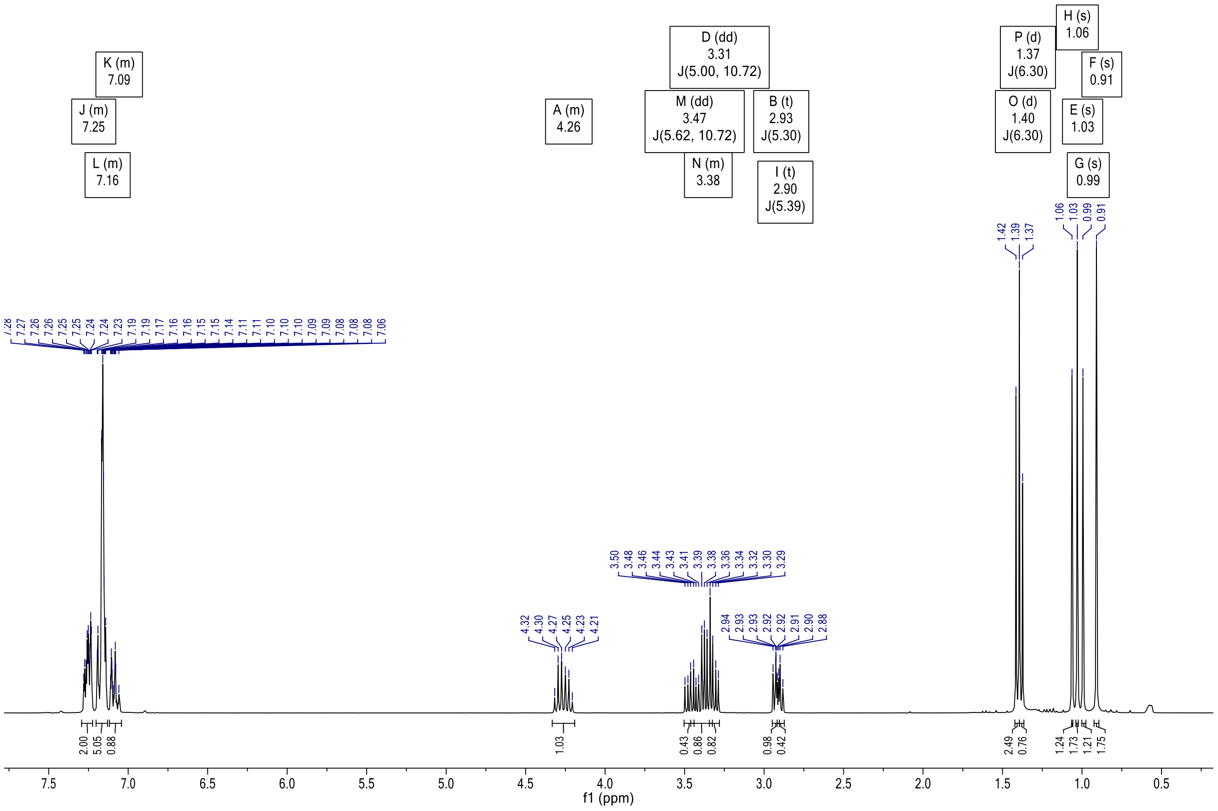
K (m)
7.09
J (m)
7.25
L (m)
7.16
A (m)
4.26
D (dd)
3.31
J(5.00, 10.72)
M (dd)
3.47
J(5.62, 10.72)
N (m)
3.38
B (t)
2.93
J(5.30)
I (t)
2.90
J(5.39)
P (d)
1.37
J(6.30)
O (d)
1.40
J(6.30)
H (s)
1.06
F (s)
0.91
E (s)
1.03
G (s)
0.99
f1 (ppm)

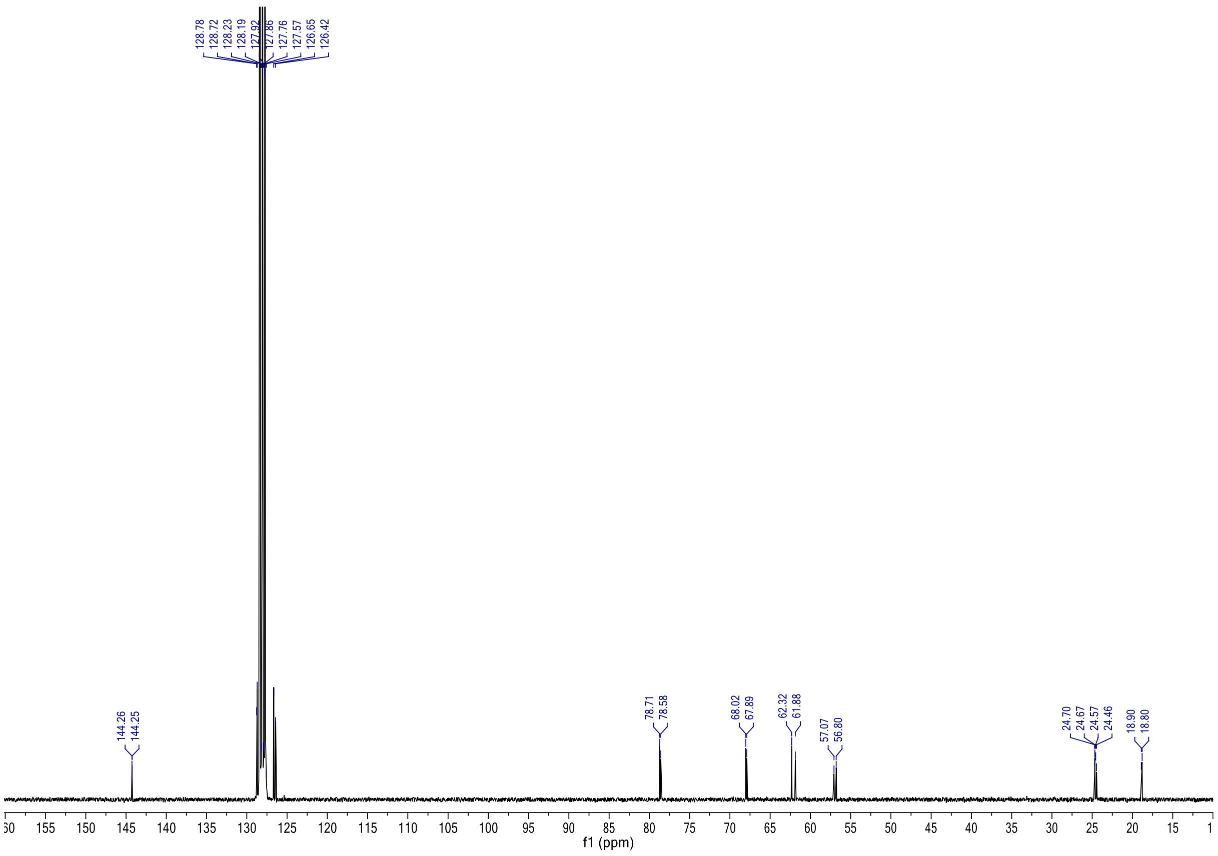
128.78
128.72
128.23
128.19
127.92
127.86
127.76
127.57
126.65
126.42
144.26
144.25
78.71
78.58
68.02
67.89
62.32
61.88
57.07
56.80
24.70
24.67
24.57
24.46
18.90
18.80
f1 (ppm)

3.2.4 Synthesis of 2,2-dimethyl-3-{[1-(4-methylphenyl)ethoxy]methyl}oxirane **3d**

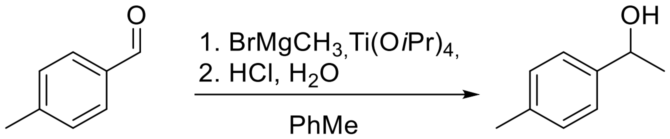


The reaction is performed in accordance to a literature procedure.[11] In a flame dried schlenk-flask 29.6 ml Ti(O$^i$Pr)$_4$ (100 mmol, 5.0 eq.) is dissolved in 160 ml toluene and cooled to -40 °C. 8.4 ml Methylmagnesium bromide (3 M in $Et_2O$, 25.3 mmol, 1.3 eq.) is added dropwise. After complete addition 2.40 g *p*-tolualdehyde (20.0 mmol, 1.0 eq.) is slowly added and the mixture is stirred for 4 h at the same temperature. The reaction is allowed to warm up to room temperature for 30 min and quenched with 100 ml 2 M HCl. The phases are separated, and the aqueous layer is extracted with EA three times. The combined organic extracts are washed with saturated $NaHCO_3$ solution and brine. The solvent is dried over $MgSO_4$ and removed under reduced pressure(50 mbar, 40 °C). 2.49 g Crude product is isolated as a colourless oil and used without further purification.

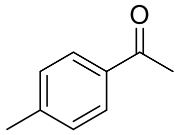


**3d-p**
$C_9H_{10}O$
134,18 g/mol

Analytical data is in accordance with literature.[11]

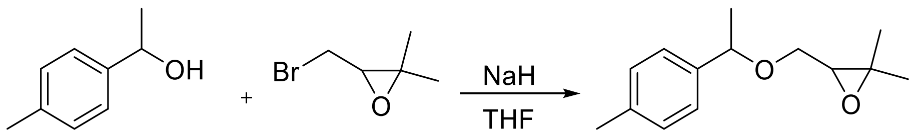


According to GP I 2.49 g 4-methyl-α-methylbenzyl alcohol **3d-p** (18.3 mmol, 1.1 eq.), 2.77 g 1-bromo-3-methyl-2,3-epoxybutane (16.8 mmol, 1.0 eq.) and 930 mg NaH (60% dispersion in mineral oil, 23.3 mmol, 1.4 eq.) are reacted for 23 h in THF. The reaction is quenched with 8 ml $H_2O$. Column chromatography ($SiO_2$, eluent: CH:EA, 90:10) afforded 2.21 g **3d** (10.0 mmol, 60%) as a light yellow oil.

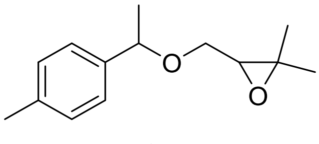


**3d**
$C_{14}H_{20}O_2$
220,31 g/mol

**$R_f$ =** 0.53 (20% EA, 80% CH). **Diastereomer a: $^1$H-NMR (300 MHz, $C_6D_6$, RT): δ [ppm]** = 7.19 (d, *J* = 7.8 Hz, 2H), 7.01 (d, *J* = 7.8 Hz, 2H), 4.32 (q, *J* = 6.5 Hz, 1H), 3.44 (dd, *J* = 10.9, 4.9 Hz, 1H), 3.36 (dd, *J* = 10.9, 5.8 Hz, 1H), 2.95 (*p*t, *J* =

5.3 Hz, 1H), 2.12 (s, 3H), 1.44 (d, *J* = 6.4 Hz, 3H), 1.04 (s, 3H), 0.93 (s, 3H). **$^{13}$C-NMR (75 MHz, $C_6D_6$, RT): δ [ppm] =** 141.3, 137.2, 129.4, 126.7, 78.6, 67.9, 62.4, 56.8, 24.7, 24.6, 21.1, 18.8. **Diastereomer b: $^{1}$H-NMR (300 MHz, $C_6D_6$, RT): δ [ppm] =** 7.19 (d, *J* = 7.8 Hz, 2H), 7.01 (d, *J* = 7.8 Hz, 2H), 4.28 – 4.20 (m, 1H), 3.51 (dd, *J* = 10.7, 5.6 Hz, 1H), 3.34 (dd, *J* = 10.7, 5.2 Hz, 1H), 2.97 – 2.88 (m, 1H), 2.12 (s, 3H), 1.43 (d, *J* = 6.3 Hz, 3H), 1.07 (s, 3H), 1.01 (s, 3H). **$^{13}$C-NMR (75 MHz, $C_6D_6$, RT): δ [ppm] =** 141.3, 137.1, 129.5, 126.5, 78.5, 67.8, 61.9, 57.1, 24.7, 24.5, 21.1, 18.9. **IR:** 2975, 2927, 2864, 1377, 1303, 1093, 1059, 868, 817, 761, 560, 516, 419, 408 cm$^{-1}$. **HRMS (ESI+):** *m/z* calculated for $C_{14}H_{20}O_2Na^+$: 243.1356 u, found: 243.1355 u.

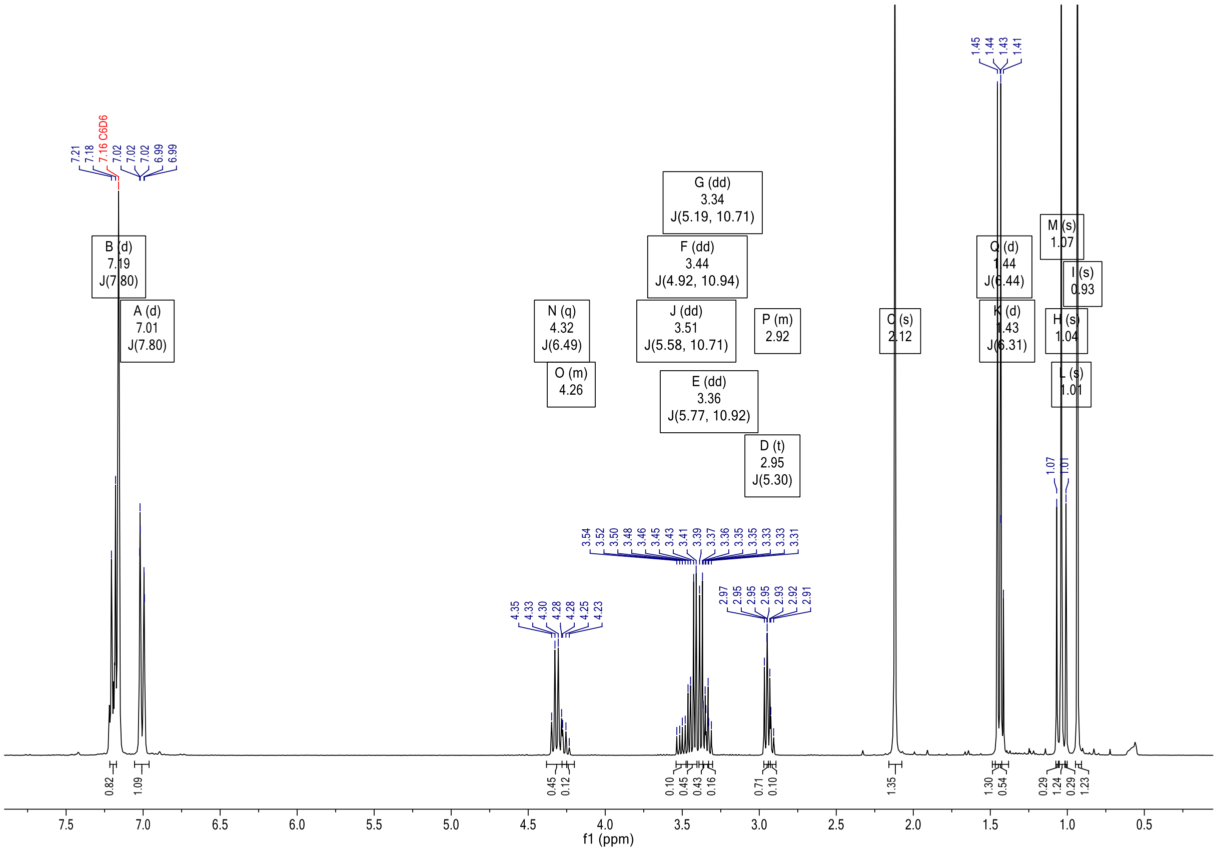

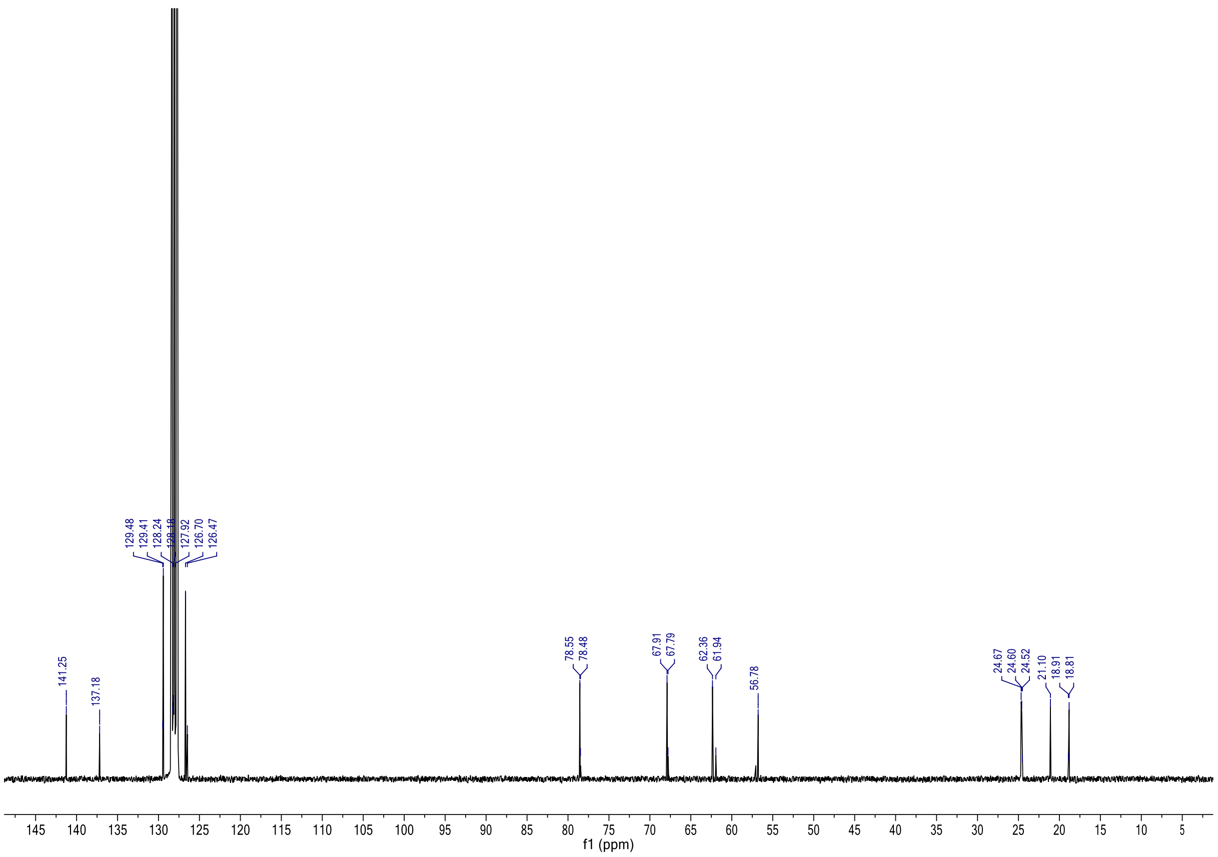


## 3.2.5 Synthesis of 2,2-dimethyl-3-{[1-(4-methoxyphenyl)ethoxy]methyl}oxirane **3e**

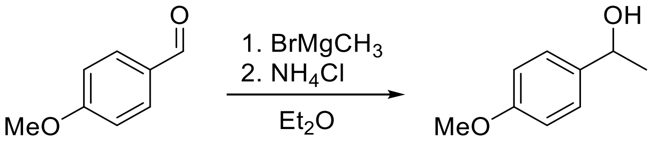


In a flame dried schlenk flask 5.6 ml Methylmagnesium bromide (3 M in $Et_2O$, 16.8 mmol, 1.2 eq.) is added dropwise to a solution of 1.90 g 4-methoxy benzaldehyde (14.0 mmol, 1.0 eq.) in 20 ml $Et_2O$ at 0 °C under argon. The resulting mixture is stirred for 20 h at room temperature. The mixture is quenched with 20 ml 4% aqueous $NH_4Cl$ solution at 0 °C and the organic layer is separated. The aqueous layer was extracted three times with EA. The combined organic layers are dried over $MgSO_4$ and concentrated under reduced pressure (50 mbar, 40 °C). 1.81 g crude product is isolated as a colourless oil and used without further purification.

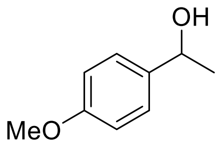


**3e-p**
$C_9H_{12}O_2$
152,19 g/mol

Analytical data is in accordance with literature.[12]

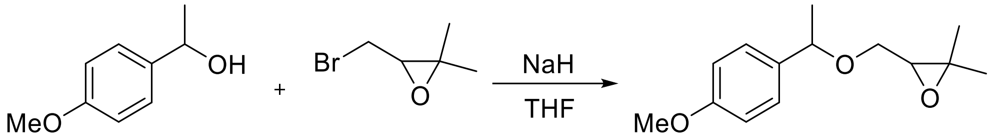


According to GP I 1.80 g 4-methoxy-α-methylbenzyl alcohol **3e-p** (11.8 mmol, 1.1 eq.), 1.77 g 1-bromo-3-methyl-2,3-epoxybutane (10.8 mmol, 1.0 eq.) and 602 mg NaH (60% dispersion in mineral oil, 15.1 mmol, 1.4 eq.) are reacted for 16 h in THF. The reaction is quenched with 8 ml $H_2O$. $H_2O$ is used for washing during the work up. Column chromatography ($SiO_2$, eluent: CH:EA, 85:15) afforded 1.58 g **3e** (6.69 mmol, 62%) as a colourless oil.

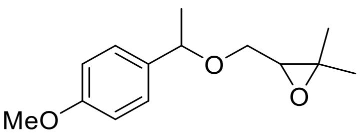


**3e**
$C_{14}H_{20}O_3$
236,31 g/mol

**$R_f$ =** 0.37 (20% EA, 80% CH). **Diastereomer a: $^{1}$H-NMR (300 MHz, $C_6D_6$, RT): δ [ppm] =** 7.19 (d, *J* = 8.6 Hz, 2H), 6.80 (dd, *J* = 8.6 Hz, 2H), 4.31 (q, *J* = 6.3 Hz, 1H), 3.51 (dd, *J* = 10.7, 5.6 Hz, 1H), 3.42 – 3.30 (m, 1H), 3.31 (s, 3H), 2.93 (*p*t, *J* = 5.3 Hz, 1H), 1.45 (d, *J* = 6.3 Hz, 3H), 1.05 (s, 3H), 0.95 (s, 3H). **$^{13}$C-NMR (75 MHz, $C_6D_6$, RT): δ [ppm] =** 159.8, 136.1, 127.9, 114.2, 78.2, 67.8, 62.4, 56.8, 54.8, 24.7, 24.6, 18.8. **Diastereomer b: $^{1}$H-NMR (300 MHz, $C_6D_6$, RT): δ [ppm] =** 7.20 (d, *J* = 8.6 Hz, 2H), 6.79 (dd, *J* = 8.6 Hz, 2H), 4.25 (q, *J* = 6.4 Hz, 1H), 3.43 (dd, *J* = 11.0, 4.9 Hz, 1H), 3.42 – 3.30 (m, 1H), 3.31 (s, 3H), 2.96 (dd, *J* = 5.8, 4.9 Hz, 1H), 1.43 (d, *J* = 6.4 Hz, 3H), 1.08 (s, 3H), 1.02 (s, 3H). **$^{13}$C-NMR (75 MHz, $C_6D_6$, RT): δ [ppm] =** 159.7, 136.1, 127.7, 114.3, 78.2, 67.7, 62.0, 57.1, 54.8, 24.7, 24.5, 18.9. **IR:** 2973, 2929, 1612, 1511, 1457, 1378, 1303, 1288, 1241, 1174, 1090, 1059, 1034, 831, 811, 760, 590, 564, 527, 411 $cm^{-1}$. **HRMS (ESI+):** *m/z* calculated for $C_{14}H_{21}O_3$ +$CH_3CN^+$: 278.1751 u, found: 278.1748 u.

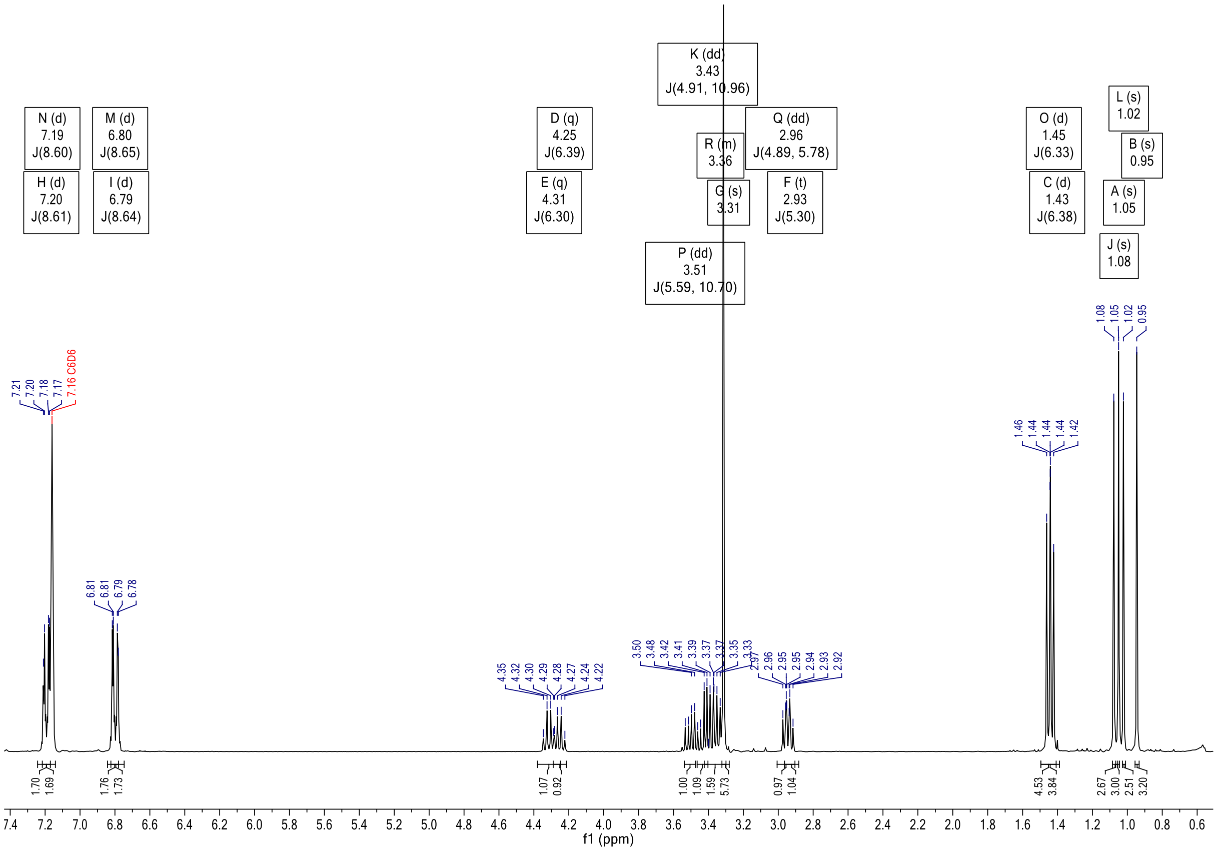
K (dd)
3.43
J(4.91, 10.96)
N (d)
7.19
J(8.60)
M (d)
6.80
J(8.65)
D (q)
4.25
J(6.39)
Q (dd)
2.96
J(4.89, 5.78)
O (d)
1.45
J(6.33)
L (s)
1.02
B (s)
0.95
H (d)
7.20
J(8.61)
I (d)
6.79
J(8.64)
E (q)
4.31
J(6.30)
R (m)
3.36
G (s)
3.31
F (t)
2.93
J(5.30)
C (d)
1.43
J(6.38)
A (s)
1.05
J (s)
1.08
P (dd)
3.51
J(5.59, 10.70)
7.16 C6D6
f1 (ppm)

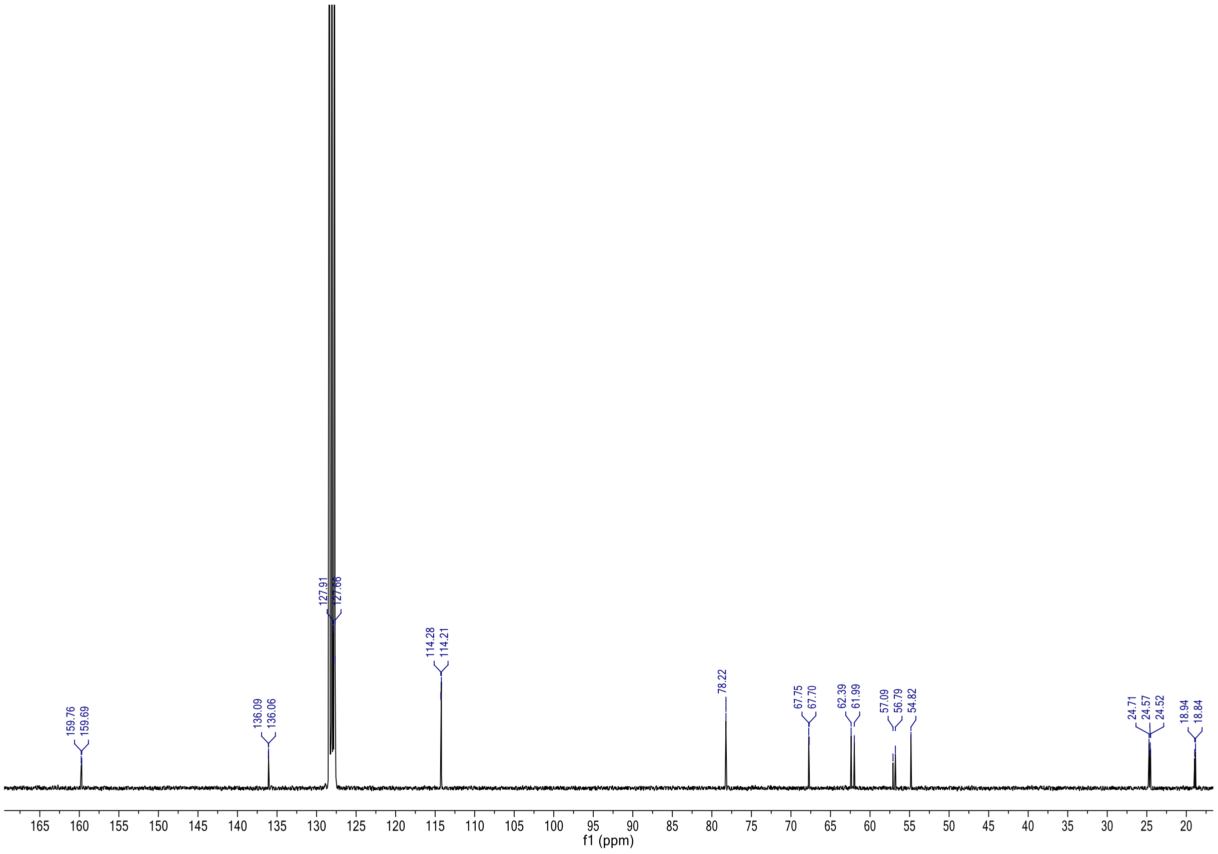
159.76
159.69
136.09
136.06
127.91
127.66
114.28
114.21
78.22
67.75
67.70
62.39
61.99
57.09
56.79
54.82
24.71
24.57
24.52
18.94
18.84
f1 (ppm)

## 3.3 Benzyl amine synthesis **5a-5f**

### 3.3.1 Synthesis of *N*-benzyl-1-(3,3-dimethyloxiran-2-yl)-*N*-methylmethanamine **5a**

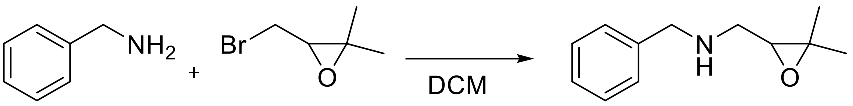

The reaction is performed in accordance to a literature procedure.[13] 4.62 g 3,3-Dimethylallylbromide (28.0 mmol, 1.0 eq.) is dissolved in 140 ml DCM and cooled to – 30 °C. Then 2.9 ml benzylamine (28.0 mmol, 1.0 eq.) is added dropwise. After complete addition the reaction is allowed to warm up to room temperature for 7 h. The mixture is washed with 2 M NaOH two times, then dried over $MgSO_4$ and the solvent is removed. The residue is purified by column chromatography ($SiO_2$, eluent: DCM:MeOH, 98:2) affording 1.06 g **5a-p** (5.55 mmol, 20%) as a colourless oil.

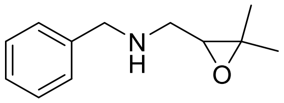

**5a-p**
$C_{12}H_{17}NO$
191.27 g/mol

Analytical data is in accordance with literature.[14]

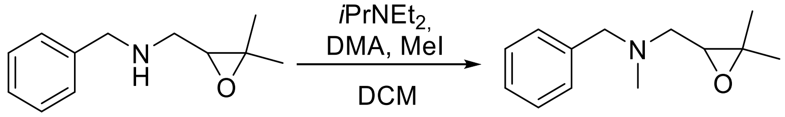

1.04 g Methyliodide (7.34 mmol, 1.46 eq.), 1.3 ml *i*$PrNEt_2$ (7.84 mmol, 1.56 eq.), 6.3 mg DMAP (0.0516 mmol, 0.01 eq.) are sequentially added to a stirred solution of the 960 mg amine **5a-p** (5.02 mmol, 1.0 eq.) in 20 ml DCM and the resultant solution was stirred at room temperature for 22 h. The mixture is washed with $H_2O$ and dried over $MgSO_4$. The solvent is removed under reduced pressure. Purification by column chromatography ($SiO_2$, eluent: CH:EA:TEA, 90:5:5) yielded 387 mg **5a** (1.88 mmol, 36%) product as a colourless oil.

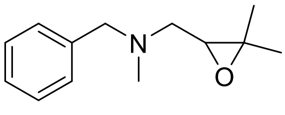

**5a**
$C_{13}H_{19}NO$
205,30 g/mol

**$R_f$ =** 0.41 (5% EA, 5% TEA, 90% CH). **$^1$H-NMR (300 MHz, $C_6D_6$, RT): δ [ppm] =** 7.42 – 7.29 (m, 2H), 7.24 – 7.17 (m, 2H) 7.14 – 7.07 (m, 1H), 3.51 (d, *J* = 13.2 Hz, 1H), 3.31 (d, *J* = 13.2 Hz, 1H), 2.86 (dd, *J* = 6.0, 4.4 Hz, 1H), 2.55 (dd, *J* = 13.1, 4.4 Hz, 1H), 2.36 (dd, *J* = 13.1, 6.0 Hz, 1H), 2.20 (s, 3H), 1.07 (s, 3H), 1.02 (s, 3H). **$^{13}$C-NMR (75 MHz, $C_6D_6$, RT): δ [ppm] =** 139.8, 129.2, 128.6, 127.3, 62.9,

62.6, 56.9, 56.5, 42.9, 24.8, 19.0. **IR:** 2960, 2786, 1453, 1377, 1123, 1025, 869, 737, 698, 680, 473 cm$^{-1}$. **HRMS (ESI+):** *m/z* calculated for $C_{13}H_{20}NO^{+}$: 206.1539 u, found: 206.1549 u.

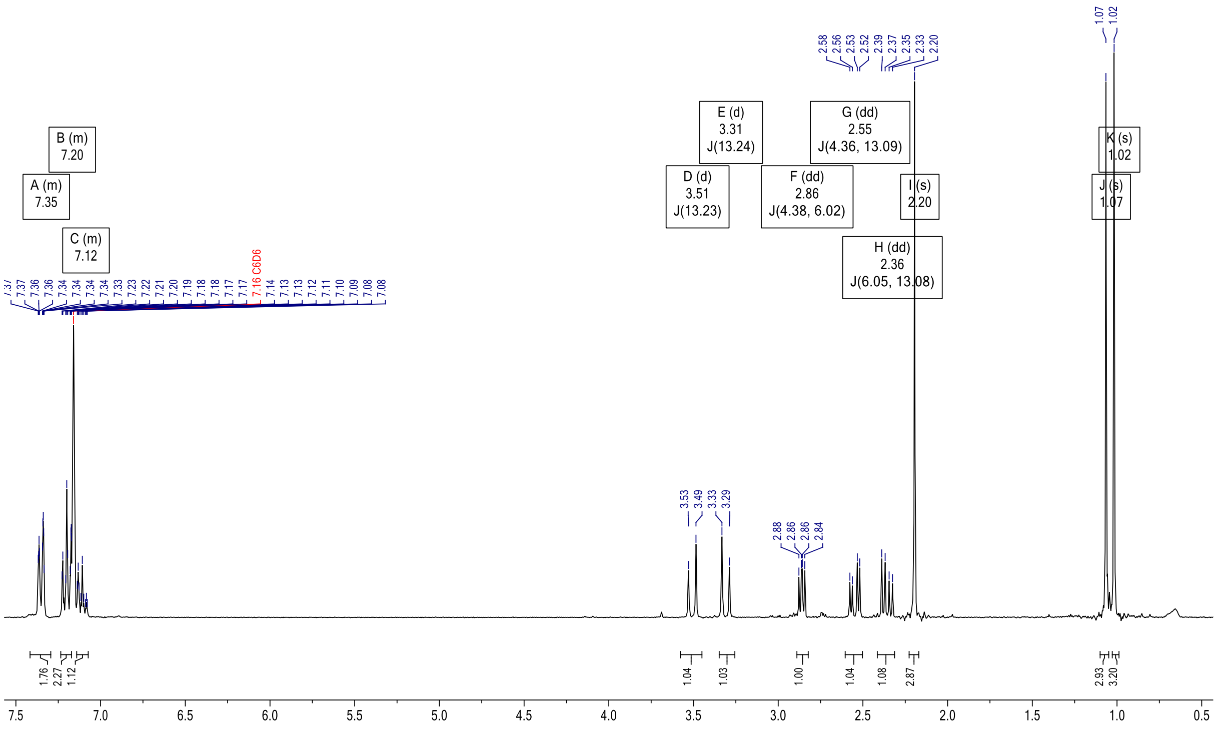

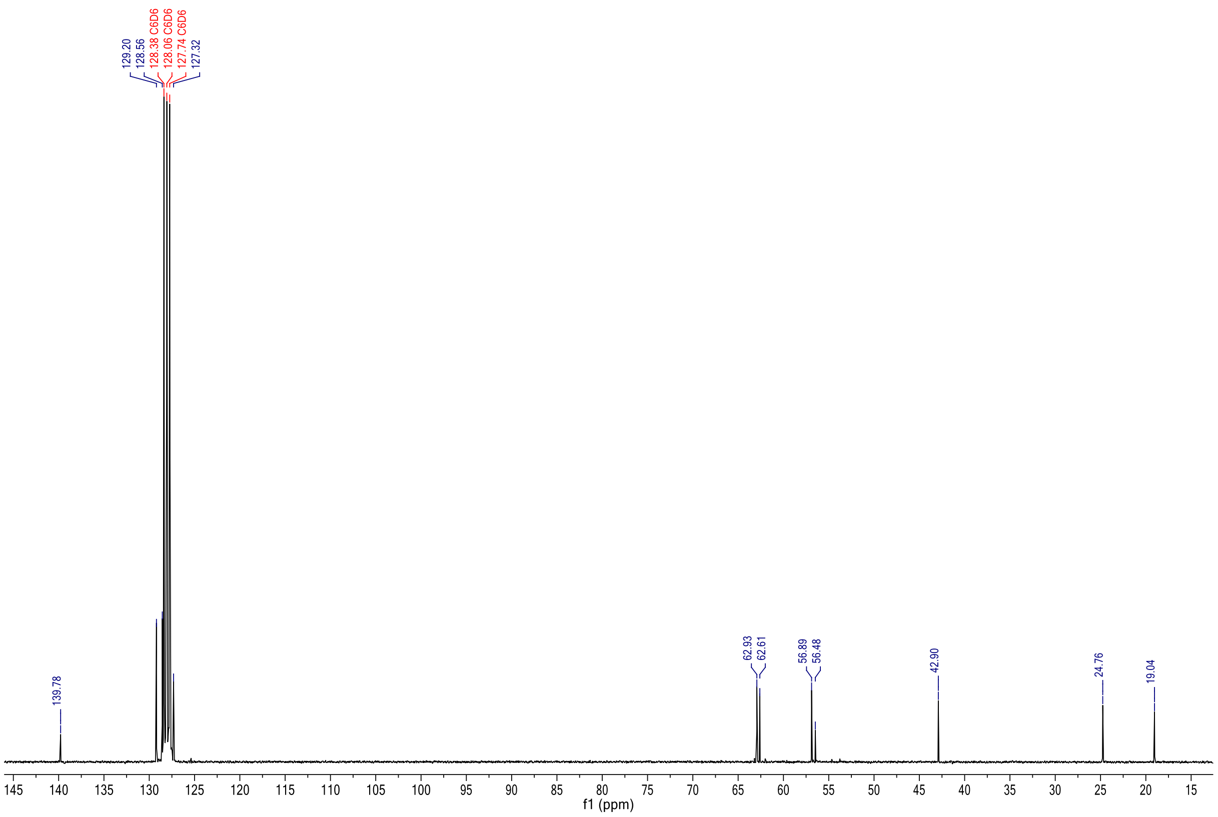


### 3.3.2 Synthesis of *N*,*N*-dibenzyl-1-(3,3-dimethyloxiran-2-yl)methanamine **5b**

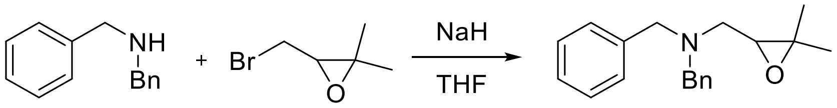


1.50 g Dibenzylamine (7.60 mmol, 1.0 eq.) and 1.53 g 1-bromo-3-methyl-2,3-epoxybutane (9.12 mmol, 1.2 eq.) are dissolved in 65 ml THF. Then 426 mg NaH (60% dispersion in mineral oil, 10.6 mmol, 1.4 eq.) are added and the mixture is stirred for 21 h at room temperature. The reaction is quenched with 10 ml $H_2O$ and diluted with 40 ml CH. The mixture is washed three times with $H_2O$, once with brine, dried over $MgSO_4$ and the solvent is removed under reduced pressure. Column chromatography ($SiO_2$, eluent: CH:EA, 97:3) yielded 527 mg **5b** (1.87 mmol, 25%) of a yellow oil.

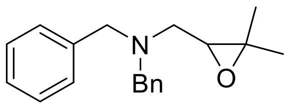


**5b**
$C_{19}H_{23}NO$
281,40 g/mol

**$R_f$ =** 0.51 (30% EA, 70% CH). **$^1$H-NMR (300 MHz, $C_6D_6$, RT): δ [ppm] =** 7.43 – 7.36 (m, 4H), 7.24 – 7.17 (m, 4H), 7.13 – 7.07 (m, 2H), 3.79 (d, *J* = 13.7 Hz, 2H), 3.40 (d, *J* = 13.7 Hz, 2H), 2.87 (dd, *J* = 6.3, 4.1 Hz, 1H), 2.71 (dd, *J* = 13.6, 4.1 Hz, 1H), 2.47 (dd, *J* = 13.6, 6.3 Hz, 1H), 0.99 (s, 3H), 0.93 (s, 3H). **$^{13}$C-NMR (75 MHz, $C_6D_6$, RT): δ [ppm] =** 139.9, 129.2, 128.6, 127.3, 62.6, 59.2, 56.5, 53.2, 24.7, 19.0. **IR:** 2961, 2925, 2796, 1494, 1453, 1376, 1245, 1118, 1118, 1072,

1028, 971, 905, 814, 735, 697, 493, 464 cm$^{-1}$. **HRMS (ESI+):** *m/z* calculated for $C_{19}H_{24}NO^{+}$: 282.1852 u, found: 282.1849 u.

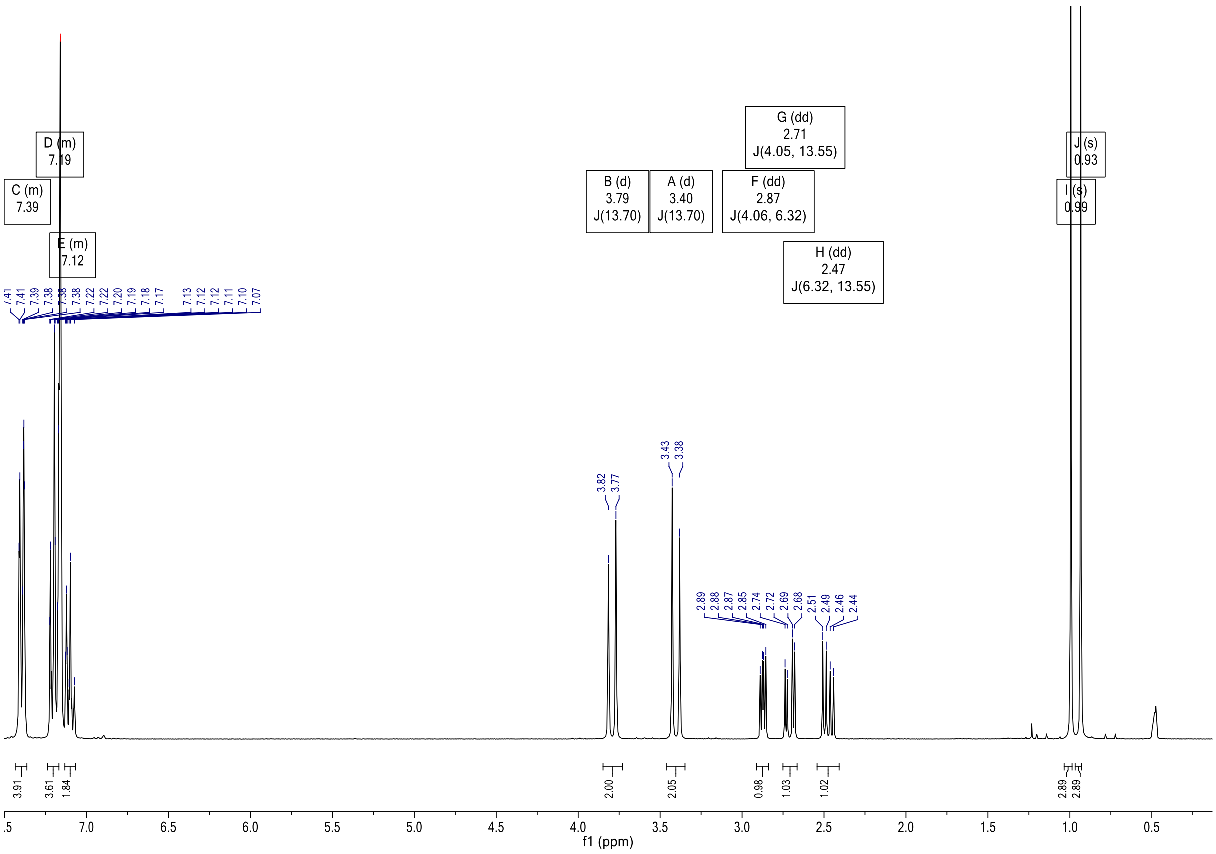

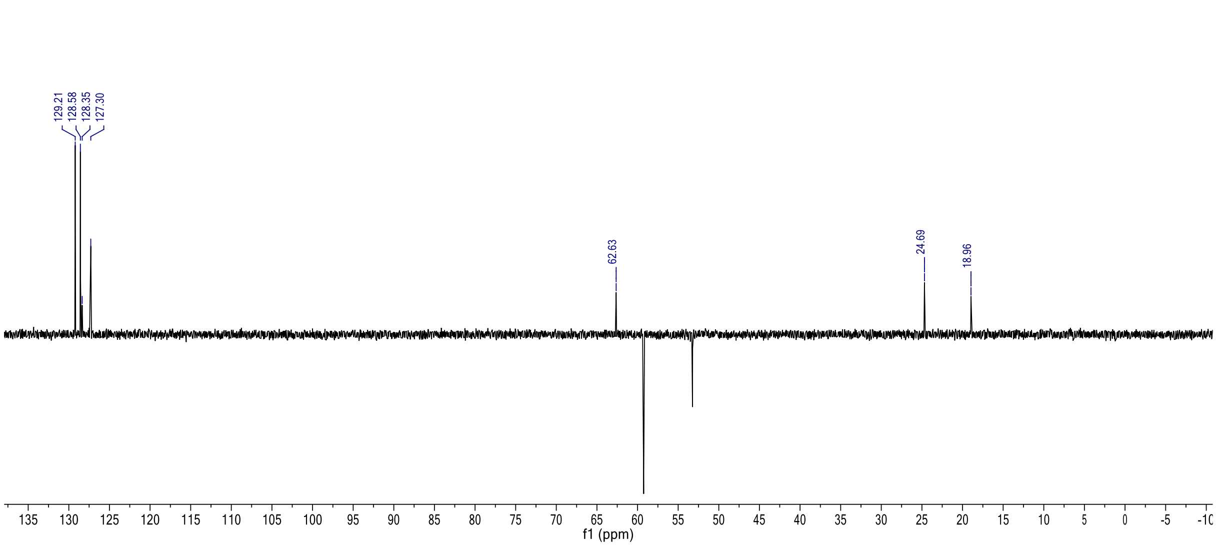

### 3.3.3 Synthesis of *N*-[(3,3-dimethyloxiran-2-yl)methyl]-*N*-methyl-1-phenylethan-1-amine **5c**

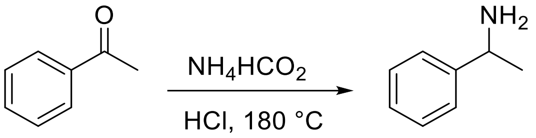


According to GP V, 12.0 g acetophenone (100 mmol, 1.0 eq.) is converted into the corresponding α-methylbenzyl amine. Workup yielded 3.64 g **5c-p1** as a light yellow oil.

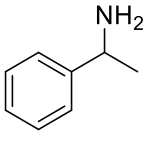


**5c-p1**
$C_8H_{11}N$
121,18 g/mol

Analytical data is in accordance with literature.[15]

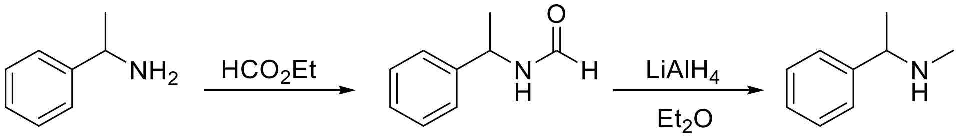


According to GP VI 3.64 g α-methylbenzyl amine **5c-p1** is refluxed in 80 ml $HCO_2Et$ and the corresponding formamide is reduced by 2.20 g $LiAlH_4$ (57.9 mmol). Workup yielded 3.35 g **5c-p2** (24.8 mmol, 25% via 3 steps) as a colourless oil.

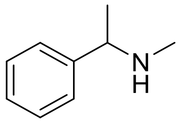


**5c-p2**
$C_9H_{13}N$
135,21 g/mol

Analytical data is in accordance with literature.[16]

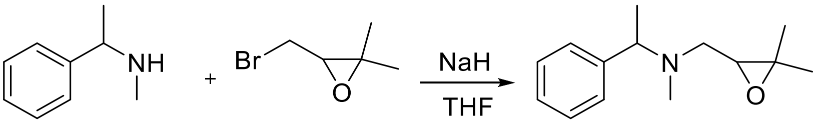


According to GP I 2.00 g **5c-p2** (14.8 mmol, 1.00 eq.), 2.57 g 1-bromo-3-methyl-2,3-epoxybutane (15.5 mmol, 1.05 eq.) and 649 mg NaH (60% dispersion in mineral oil, 16.2 mmol, 1.10 eq.) are reacted in THF for 22 h. Column chromatography ($SiO_2$, eluent: CH:EA:TEA, 93:2:5) afforded 1.32 g **5c** (6.02 mmol, 41%) as a yellow oil.

**$R_f$ =** 0.32 (5% EA, 5% TEA, 90% CH). **Diastereomer a: $^{1}$H-NMR (300 MHz, $C_6D_6$, RT): δ [ppm] =** 7.37 – 7.31 (m, 2H), 7.25 – 7.17 (m, 2H), 7.14 – 7.06 (m, 1H), 3.41 (q, *J* = 6.7 Hz, 1H), 2.87 (dd, *J* = 6.1, 4.1 Hz, 1H), 2.63 (dd, *J* = 13.3, 4.1 Hz, 1H), 2.34 (dd, *J* = 13.3, 6.1 Hz, 1H), 2.27 (s, 3H), 1.23 (d, *J* = 6.7 Hz, 3H), 1.05 (s, 3H), 0.96 (s, 3H). **$^{13}$C-NMR (76 MHz, $C_6D_6$, RT): δ [ppm] =** 144.8, 128.6, 128.0, 127.2, 64.3, 63.2, 56.5, 54.6, 39.3, 24.8, 19.7, 19.0. **Diastereomer b: $^{1}$H-NMR (300 MHz, $C_6D_6$, RT): δ [ppm] =** 7.37 – 7.31 (m, 2H), 7.25 – 7.17 (m, 2H), 7.14 – 7.06 (m, 1H), 3.51 (q, *J* = 6.7 Hz, 1H), 2.86 (t, *J* = 5.3 Hz, 1H), 2.53 (d, *J* = 5.3 Hz, 2H), 2.21 (s, 3H), 1.23 (d, *J* = 6.7 Hz, 3H), 1.09 (s, 3H), 1.01 (s, 3H). **$^{13}$C-NMR (76 MHz, $C_6D_6$, RT): δ [ppm]** = 144.7, 128.5, 127.9, 127.1, 63.8, 62.7, 56.7, 53.4, 39.5, 24.8, 19.0, 17.9. **IR:** 2972, 1451, 1377, 1125, 1073, 1032, 1014, 942, 860, 759, 735, 700, 655 $cm^{-1}$. **HRMS (ESI+):** *m/z* calculated for $C_{14}H_{22}NO^{+}$: 220.1698 u, found: 220.1698 u.

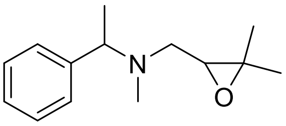


**5c**
$C_{14}H_{21}NO$
219,33 g/mol

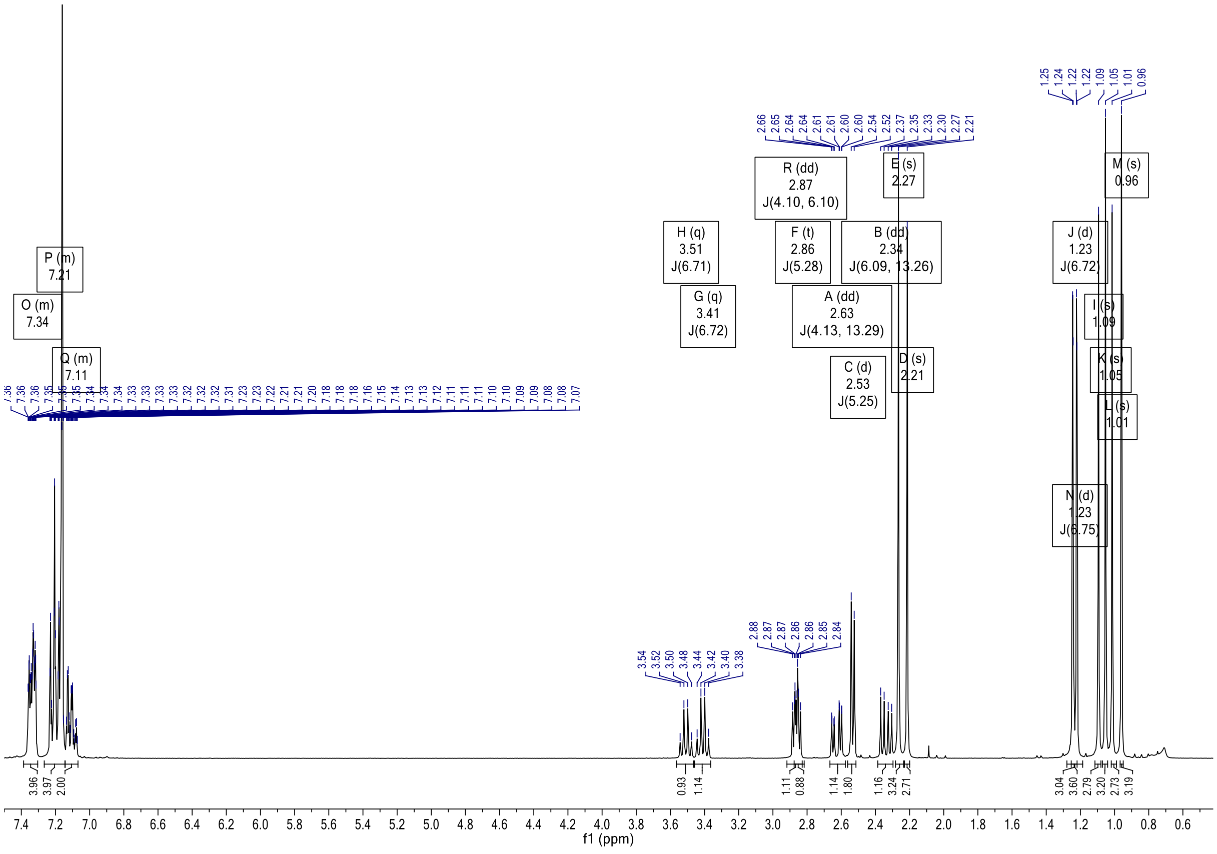

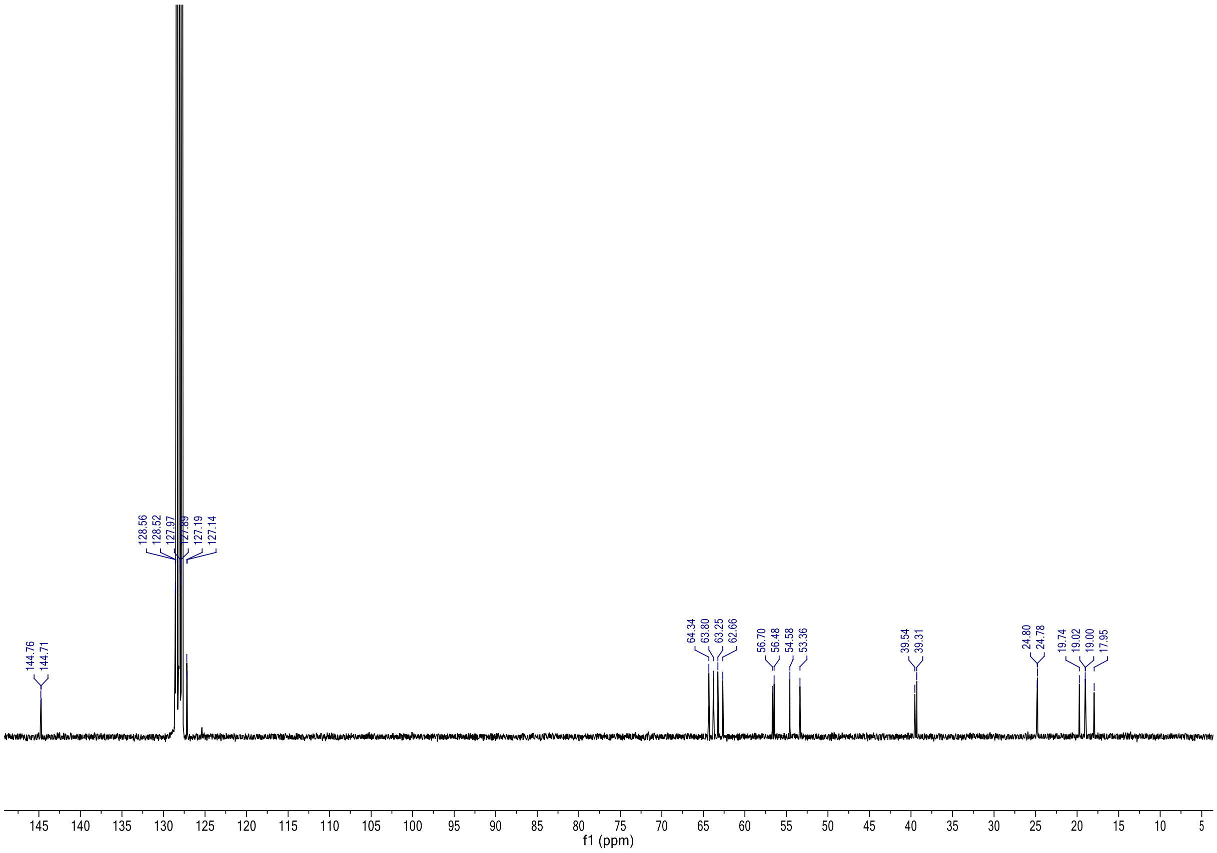


### 3.3.4 Synthesis of *N*-[(3,3-dimethyloxiran-2-yl)methyl]-*N*-methyl-1-(4-methylphenyl)-ethan-1-amine **5d**

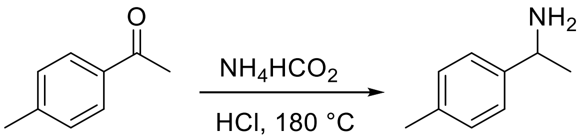


According to GP V, 13.3 g 4-methyl-acetophenone (100 mmol, 1.0 eq.) are reacted with 18.9 g ammonium formate (300 mmol, 3.0 eq.). Workup yielded 2.31 g 4-methyl-α-methylbenzyl amine **5d-p1** as a colourless oil, which is used without further purification.

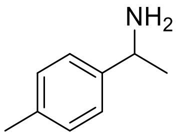


**5d-p1**
$C_9H_{13}N$
135,21 g/mol

Analytical data is in accordance with literature.[3]

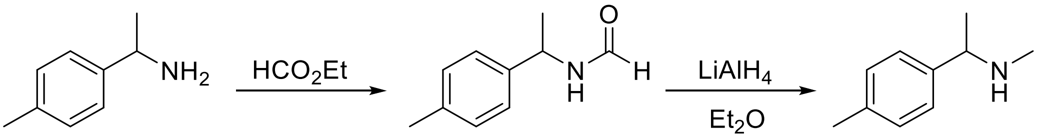

According to GP VI 2.31 g 4-methyl-α-methylbenzyl amine **5d-p1** is refluxed in 58 ml $HCO_2Et$ and the corresponding formamide is reduced by 1.27 g $LiAlH_4$ (33.4 mmol). Workup yielded 2.10 g **5d-p2** (14.1 mmol, 14% via 3 steps) as a light yellow oil.

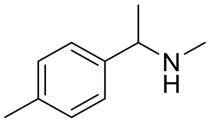

**5d-p2**
$C_{10}H_{15}N$
149,24 g/mol

Analytical data is in accordance with literature.[17]

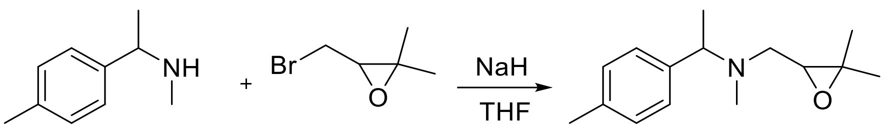

According to GP I 2.10 g **5d-p2** (14.1 mmol, 1.00 eq.), 2.44 g 1-bromo-3-methyl-2,3-epoxybutane (14.8 mmol, 1.05 eq.) and 620 mg NaH (60% dispersion in mineral oil, 15.5 mmol, 1.10 eq.) are reacted in THF for 22 h. Column chromatography ($SiO_2$, eluent: CH:EA:TEA, 93:2:5) afforded 1.66 g **5d** (7.09 mmol, 50%) as a yellow oil.

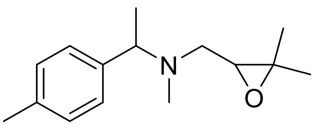

**5d**
$C_{15}H_{23}NO$
233,36 g/mol

**$R_f$ =** 0.21 (97.5% DCM, 0.5% MeOH, 2% TEA). **Diastereomer a: $^1$H-NMR (500 MHz, $C_6D_6$, RT): δ [ppm] =** 7.28 (d, *J* = 8.0 Hz, 2H), 7.07 – 7.02 (m, 2H), 3.44 (q, *J* = 6.7 Hz, 1H), 2.89 (dd, *J* = 6.0, 4.2 Hz, 1H), 2.66 (dd, *J* = 13.2, 4.2 Hz, 1H), 2.36 (dd, *J* = 13.2, 6.1 Hz, 1H), 2.29 (s, 3H), 2.15 (s, 3H), 1.27 (d, *J* = 6.7 Hz, 3H), 1.06 (s, 3H), 0.98 (s, 3H). **$^{13}$C-NMR (126 MHz, $C_6D_6$, RT): δ [ppm] =** 141.7, 136.5, 129.3, 128.0, 64.1, 63.3, 56.5, 54.6, 39.3, 24.8, 21.1, 19.8, 19.1. **Diastereomer b: $^1$H-NMR (500 MHz, $C_6D_6$, RT): δ [ppm] =** 7.27 (d, *J* = 7.7 Hz, 2H), 7.07 – 7.02 (m, 2H), 3.53 (q, *J* = 6.7 Hz, 1H), 2.88 (*p*t, *J* = 4.7 Hz, 1H), 2.58 (dd, *J* = 12.6, 4.9 Hz, 1H), 2.54 (dd, *J* = 12.6, 4.4 Hz, 1H), 2.25 (s, 3H), 2.16 (s, 3H), 1.27 (d, *J* = 6.7 Hz, 3H), 1.10 (s, 3H), 1.03 (s, 3H). **$^{13}$C-NMR (126 MHz, $C_6D_6$, RT): δ [ppm] =** 141.6, 136.4, 129.2, 127.9, 63.6, 62.8, 56.7, 53.4, 39.5, 24.8, 21.1, 19.0, 18.0. **IR:** 2972, 1455, 1377, 1122, 1074, 1045, 1021, 943, 860, 820, 681, 551 $cm^{-1}$. **HRMS (ESI+):** *m/z* calculated for $C_{15}H_{24}NO^+$: 234.1852 u, found: 234.1856 u.

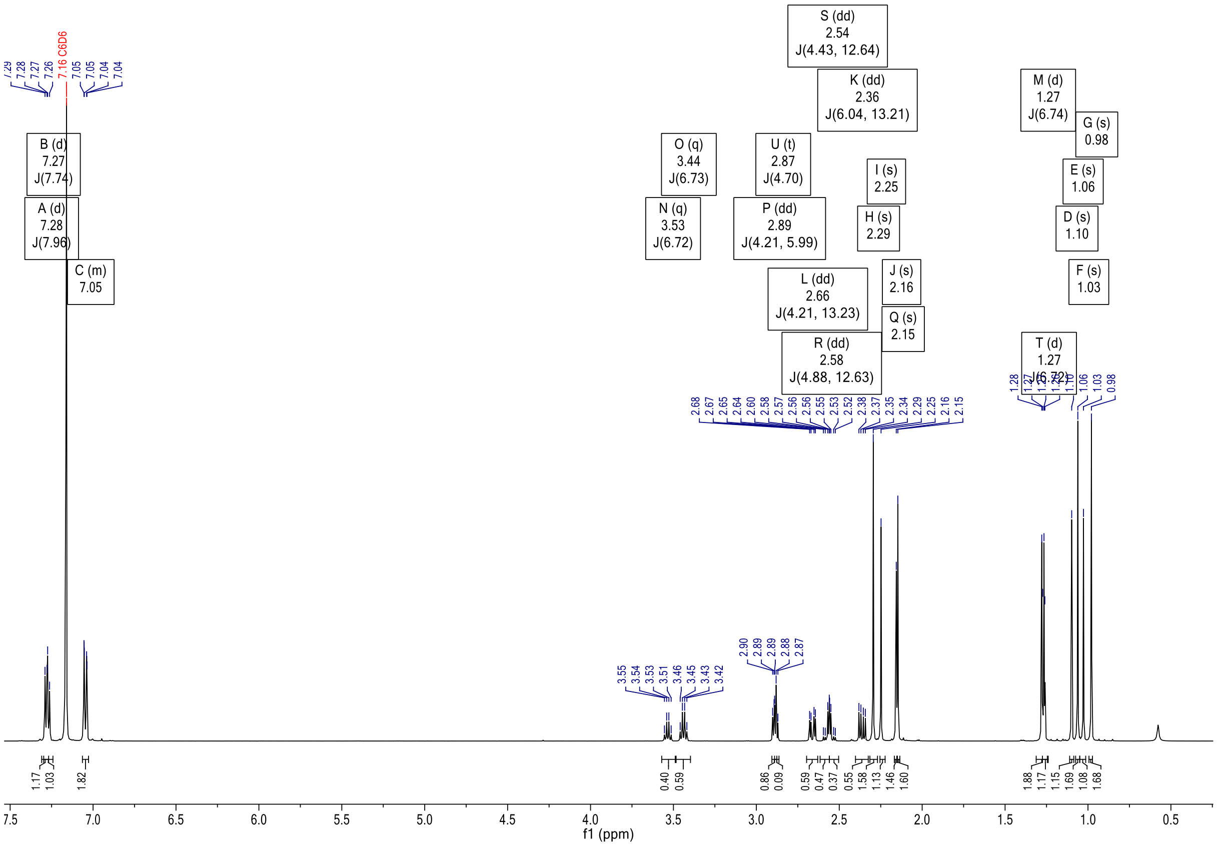

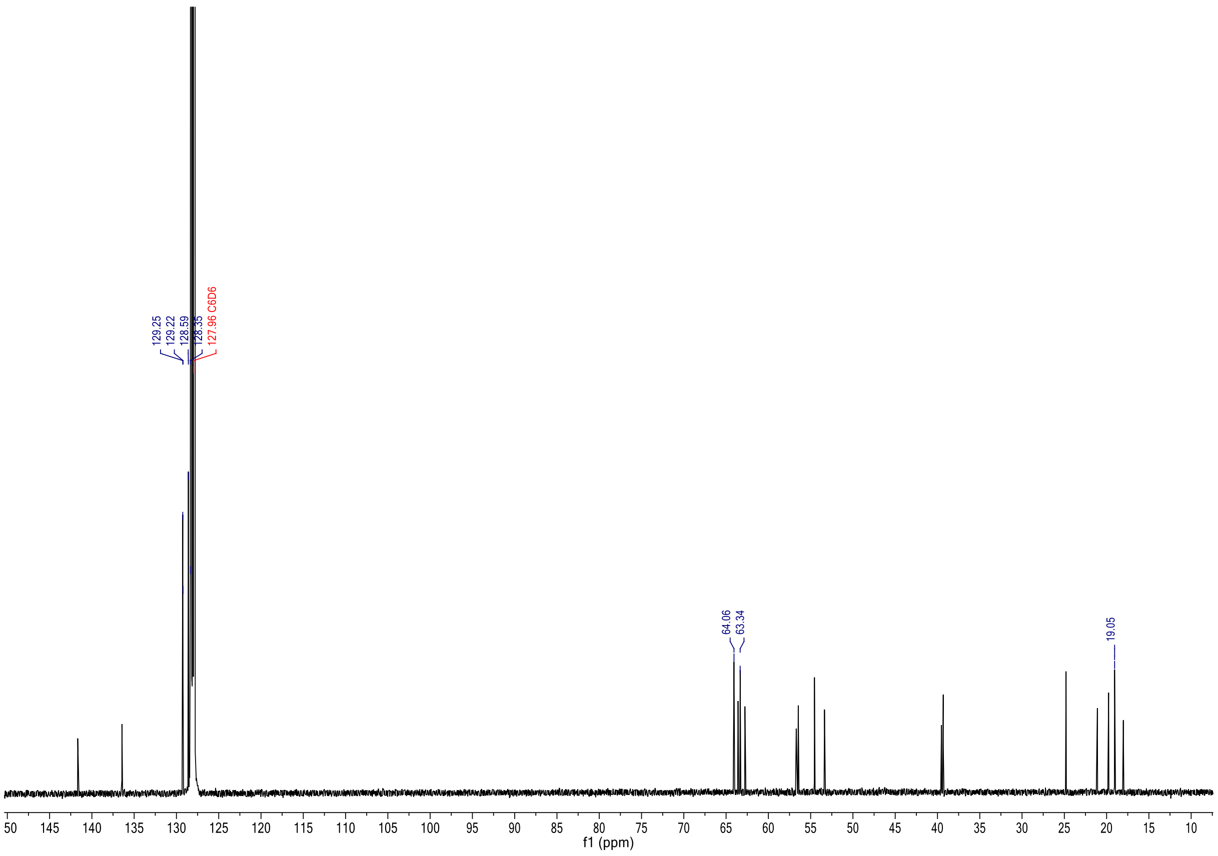

### 3.3.5 Synthesis of *N*-[(3,3-dimethyloxiran-2-yl)methyl]-1-(3-methoxyphenyl)-*N*-methylethan-1-amine **5e**

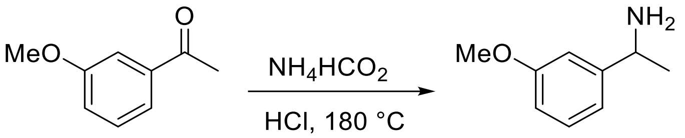


According to GP V, 15.1 g (100 mmol, 1.0 eq.) 3-methoxy-acetophenone is reacted with 16.0 g ammonium formate (250 mmol, 2.5 eq.). Workup yielded 8.56 g 3-methoxy-α-methylbenzyl amine **5e-p1** as a yellow oil, which is used without further purification.

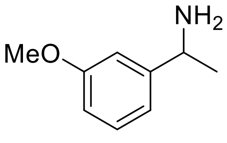


**5e-p1**
$C_9H_{13}NO$
151,21 g/mol

Analytical data is in accordance with literature.[3]

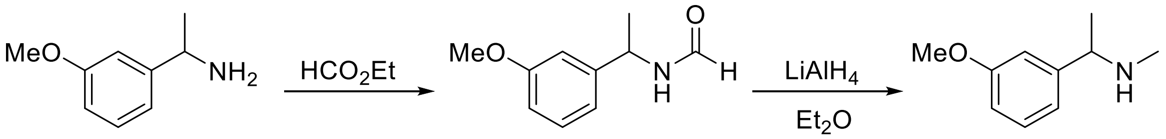


According to GP VI 8.56 g 3-methoxy-α-methylbenzyl amine **5e-p1** is refluxed in 100 ml $HCO_2Et$ and the corresponding formamide is reduced by 4.14 g $LiAlH_4$ (108 mmol). Workup yielded 8.19 g **5e-p2** (49.6 mmol, 50% via 3 steps) as a colourless oil.

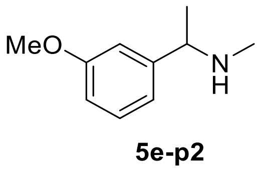


**5e-p2**
$C_{10}H_{15}NO$
165,24 g/mol

Analytical data is in accordance with literature.[16]

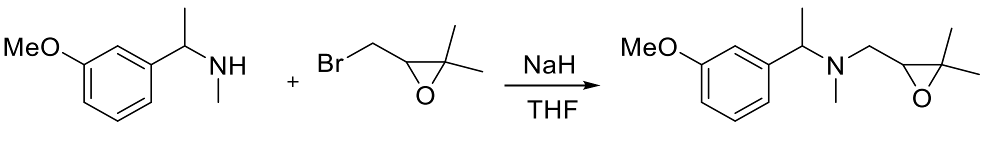


According to GP I 8.17 g **5e-p2** (49.4 mmol, 1.00 eq.), 11.02 g 1-bromo-3-methyl-2,3-epoxybutane (66.8 mmol, 1.35 eq.) and 2.67 g NaH (60% dispersion in mineral oil, 66.8 mmol, 1.35 eq.) are reacted in THF for 21 h. Column chromatography ($SiO_2$, eluent: CH:EA:TEA, 93:2:5) afforded 3.53 g **5e** (14.2 mmol, 29%) as a yellow oil.

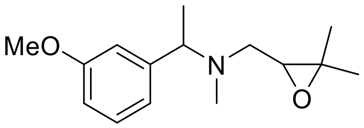


**5e**
$C_{15}H_{23}NO_2$
249,35 g/mol

**R$_f$ =** 0.11 (97% DCM, 1% MeOH, 2% TEA). **Diastereomer a: $^1$H-NMR (500 MHz, $C_6D_6$, RT): δ [ppm] =** 7.16 – 7.12 (m, 2H), 7.00 – 6.96 (m, 1H), 6.75 – 6.70 (m, 1H), 3.40 (q, *J* = 6.8 Hz, 1H), 3.39 (s, 3H), 2.88 (dd, *J* = 6.2, 4.1 Hz, 1H), 2.67 (dd, *J* = 13.3, 4.1 Hz, 1H), 2.35 (dd, *J* = 13.3, 6.2 Hz, 1H), 2.29 (s, 3H), 1.26 (d, *J* = 6.7 Hz, 3H), 1.05 (s, 3H), 0.96 (s, 3H). **$^{13}$C-NMR (126 MHz, $C_6D_6$, RT): δ [ppm] =** 160.5, 146.7, 129.5, 120.2, 113.9, 112.5, 64.5, 63.3, 56.5, 54.8, 54.7, 39.4, 24.8, 20.0, 19.0. **Diastereomer b: $^1$H-NMR (500 MHz, $C_6D_6$, RT): δ [ppm] =** 7.16 – 7.12 (m, 2H), 7.00 – 6.96 (m, 1H), 6.75 – 6.70 (m, 1H), 3.49 (q, *J* = 6.7 Hz, 1H), 3.39 (s, 3H), 2.87 (*p*t, *J* = 5.3 Hz, 1H), 2.57 (dd, *J* = 13.2, 5.5 Hz, 1H), 2.54 (dd, *J* = 13.2, 5.0 Hz, 1H), 2.24 (s, 3H), 1.25 (d, *J* = 6.7 Hz, 3H), 1.09 (s, 3H), 1.02 (s, 3H). **$^{13}$C-NMR (126 MHz, $C_6D_6$, RT): δ [ppm] =** 160.5, 146.7, 129.5, 120.2, 113.8, 112.5, 64.0, 62.6, 56.7, 54.8, 53.5, 39.7, 24.8, 19.0, 18.4. **IR:** 2973, 2836, 1599, 1584, 1486, 1454, 1434, 1377, 1282, 1252, 1150, 1043, 1020, 884, 858, 781, 702, 681 cm$^{-1}$. **HRMS (ESI+):** *m/z* calculated for $C_{15}H_{24}NO_2^+$: 250.1802 u, found: 250.1800 u.

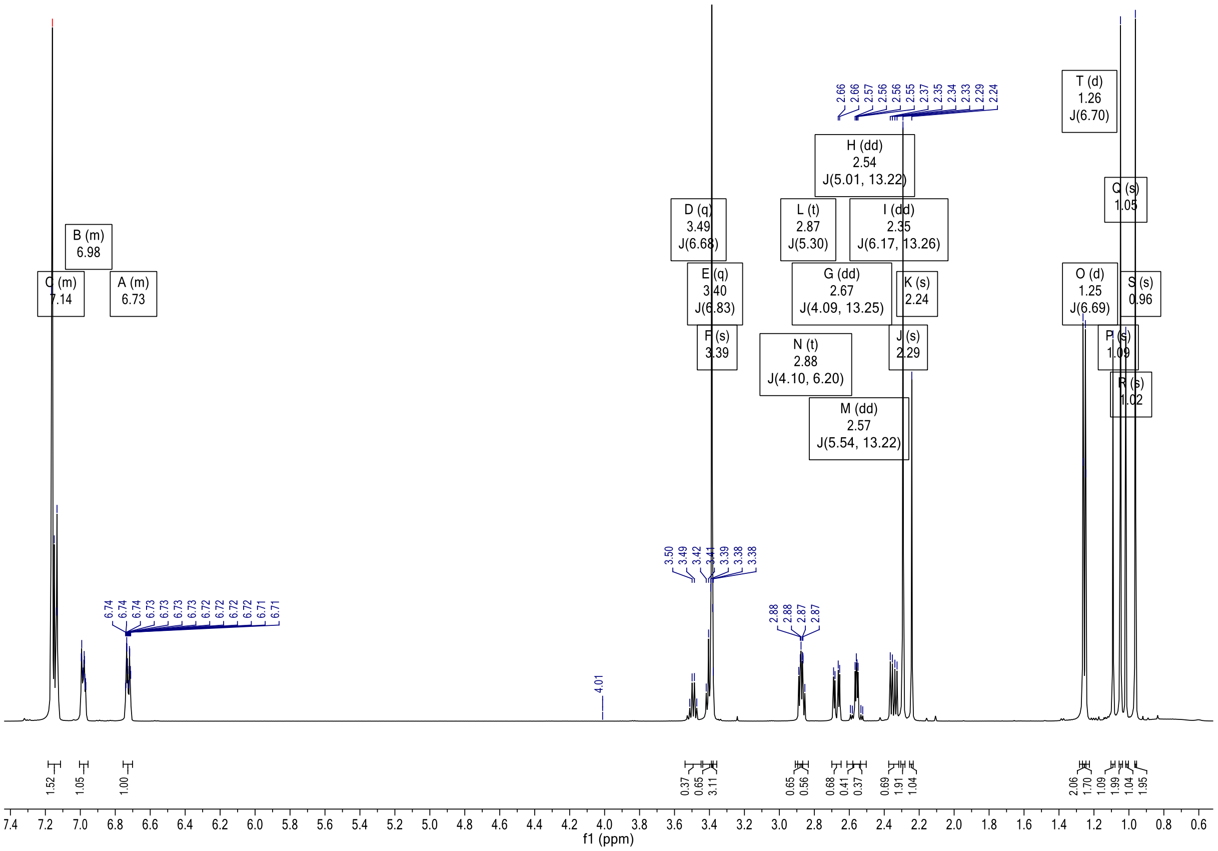

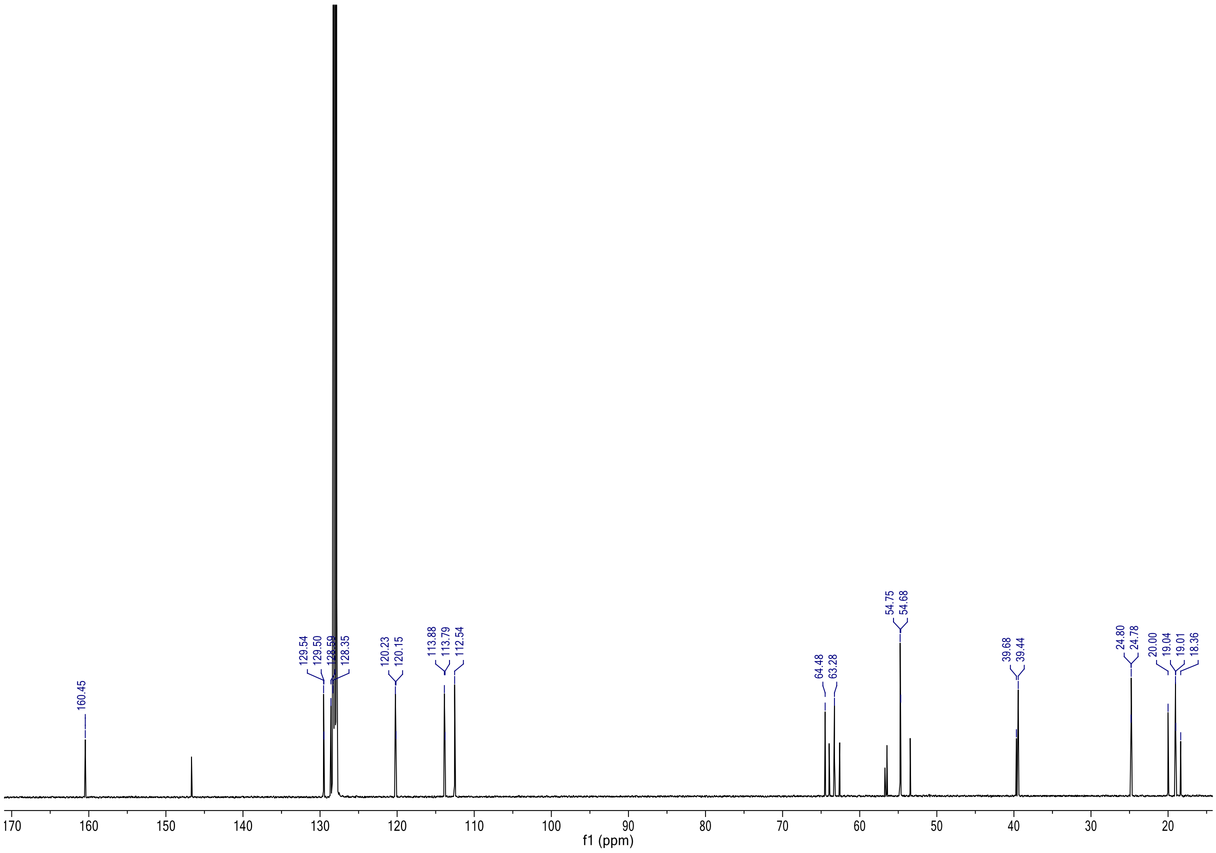


3.3.6 Synthesis of *N*-[(3,3-dimethyloxiran-2-yl)methyl]-1-(4-methoxyphenyl)-*N*-methylethan-1-amine **5f**

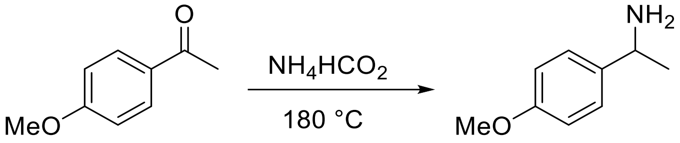


According to GP V, 12.9 g 4-methoxy-acetophenone (83.2 mmol, 1.0 eq.) are reacted with 13.3 g ammonium formate (211 mmol, 2.5 eq.). Workup yielded 2.38 g 4-methoxy-α-methylbenzyl amine **5f-p1** as a colourless oil, which is used without further purification.

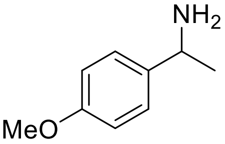


**5f-p1**
$C_9H_{13}NO$
151,21 g/mol

Analytical data is in accordance with literature.[3]

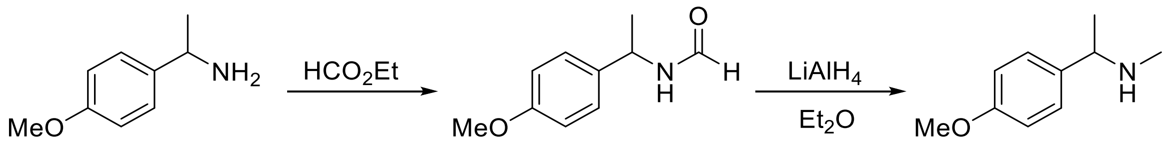


According to GP VI 2.38 g 4-methoxy-α-methylbenzyl amine **5f-p1** is refluxed in 50 ml $HCO_2Et$ and the corresponding formamide is reduced by 1.19 g $LiAlH_4$ (31.5 mmol). Workup yielded 2.43 g **5f-p2** (14.7 mmol, 18% via 3 steps) as a colourless oil.

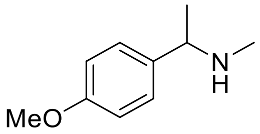


**5f-p2**
$C_{10}H_{15}NO$
165,24 g/mol

Analytical data is in accordance with literature.[16]

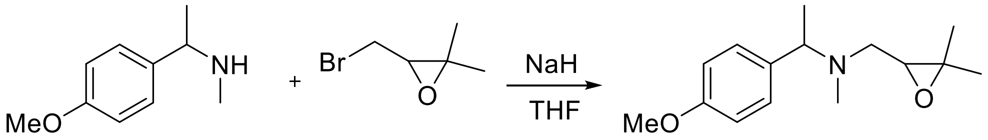


According to GP I 2.40 g **5f-p2** (14.7 mmol, 1.00 eq.), 2.55 g 1-bromo-3-methyl-2,3-epoxybutane (15.4 mmol, 1.05 eq.) and 646 mg NaH (60% dispersion in mineral oil, 16.1 mmol, 1.10 eq.) are reacted in THF for 23 h. Column chromatography ($SiO_2$, eluent: CH:EA:TEA, 93:2:5) afforded 1.29 g **5f** (5.17 mmol, 35%) as a yellow oil.

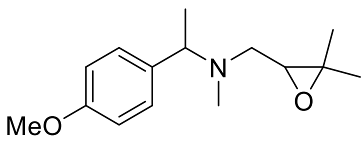


**5f**
$C_{15}H_{23}NO_2$
249,35 g/mol

**$R_f$ =** 0.23 (2% EA, 5% TEA, 93% CH). **Diastereomer a: $^1$H-NMR (500 MHz, $C_6D_6$, RT): δ [ppm] =** 7.25 (d, *J* = 8.6 Hz, 2H), 6.83 (d, *J* = 8.6 Hz, 2H), 3.53 (q, *J* = 6.7 Hz, 1H), 3.35 (s, 3H), 2.88 (*p*t, *J* = 5.3 Hz, 1H), 2.58 (dd, *J* = 13.3, 5.4 Hz, 1H), 2.54 (dd, *J* = 13.3, 5.1 Hz, 1H), 2.25 (s, 3H), 1.27 (d, *J* = 6.7 Hz, 3H), 1.11 (s, 3H), 1.04 (s, 3H). **$^{13}$C-NMR (126 MHz, $C_6D_6$, RT): δ [ppm] =** 159.2, 136.5, 128.9, 114.0, 63.2, 62.8, 56.7, 54.8, 53.3, 39.4, 24.8, 19.0, 17.9. **Diastereomer b: $^1$H-NMR (300 MHz, $C_6D_6$, RT): δ [ppm] =** 7.27 (d, *J* = 8.6 Hz, 2H), 6.84 (d, *J* = 8.6 Hz, 2H), 3.45 (q, *J* = 6.7 Hz, 1H), 3.34 (s, 3H), 2.92 – 2.87 (m, 1H), 2.65 (dd, *J* = 13.2, 4.2 Hz, 1H), 2.37 (dd, *J* = 13.2, 6.0 Hz, 1H), 2.29 (s, 3H), 1.27 (d, *J* = 6.7 Hz, 3H), 1.08 (s, 3H), 1.00 (s, 3H). **$^{13}$C-NMR (126 MHz, $C_6D_6$, RT): δ [ppm] =** 159.2, 136.4, 129.0, 114.0, 63.5, 63.3, 56.5, 54.8, 54.4, 39.2, 24.8, 19.6, 19.1. **IR:** 2970, 1610, 1510, 1456, 1377, 1242, 1176, 1035, 1115, 941, 832, 680, 556, 520 $cm^{-1}$. **HRMS (ESI+):** *m/z* calculated for $C_{15}H_{24}NO_2^+$: 250.1802 u, found: 250.1800 u.

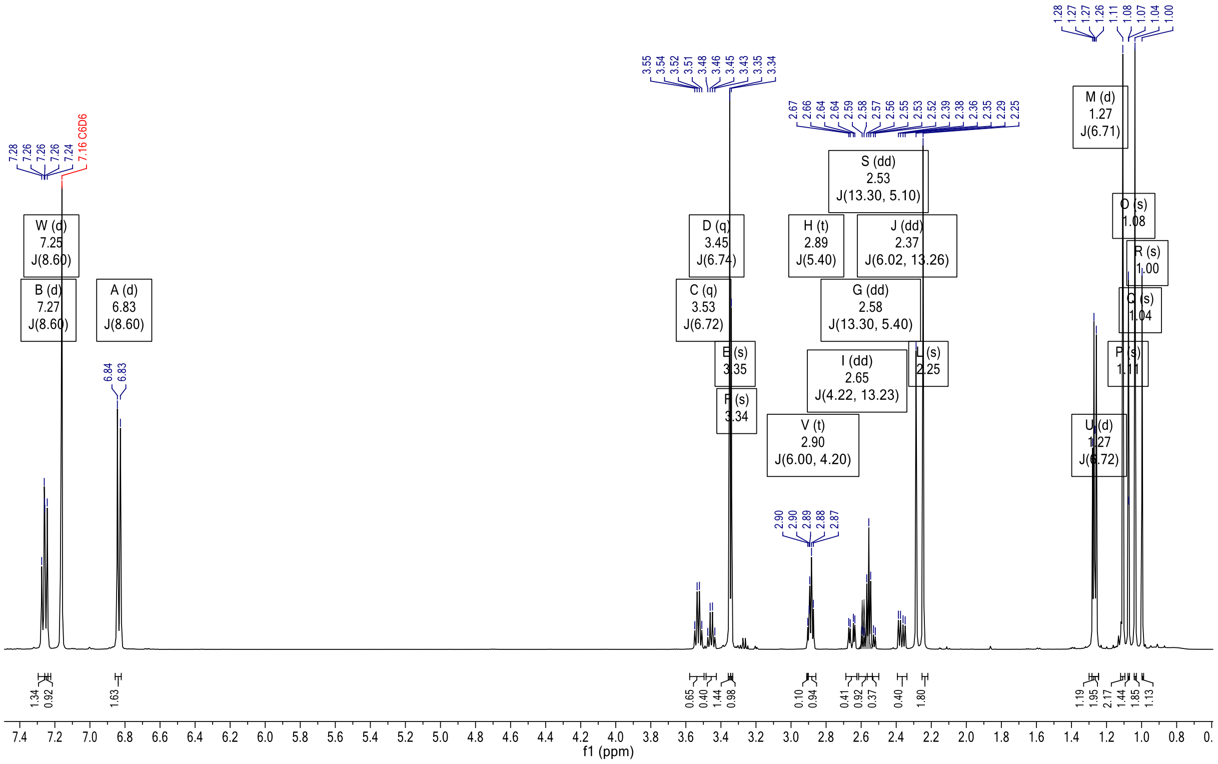
7.16 C6D6
W (d) 7.25 J(8.60)
B (d) 7.27 J(8.60)
A (d) 6.83 J(8.60)
D (q) 3.45 J(6.74)
C (q) 3.53 J(6.72)
E (s) 3.35
F (s) 3.34
S (dd) 2.53 J(13.30, 5.10)
H (t) 2.89 J(5.40)
J (dd) 2.37 J(6.02, 13.26)
G (dd) 2.58 J(13.30, 5.40)
I (dd) 2.65 J(4.22, 13.23)
V (t) 2.90 J(6.00, 4.20)
L (s) 2.25
M (d) 1.27 J(6.71)
O (s) 1.08
R (s) 1.00
Q (s) 1.04
P (s) 1.11
U (d) 1.27 J(6.72)
f1 (ppm)

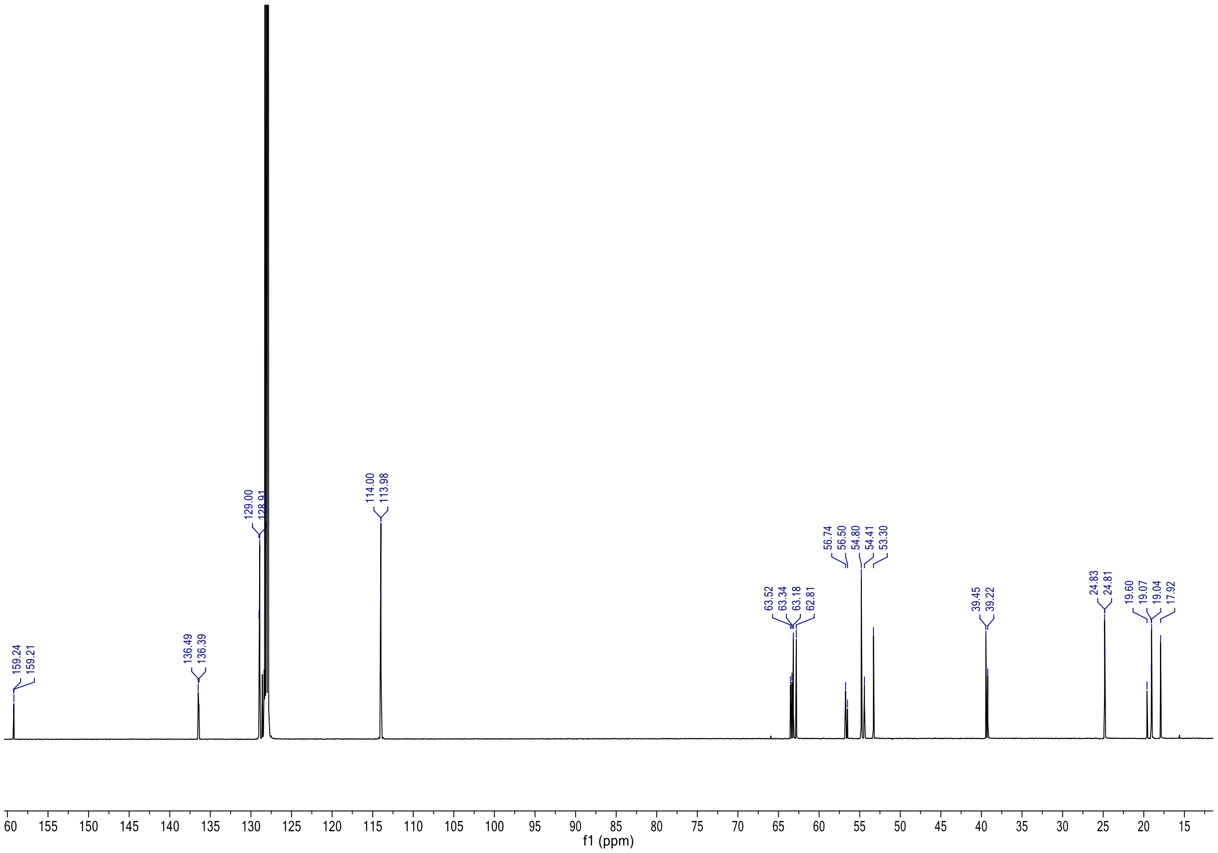
159.24
159.21
136.49
136.39
129.00
128.91
114.00
113.98
63.52
63.34
63.18
62.81
56.74
56.50
54.80
54.41
53.30
39.45
39.22
24.83
24.81
19.60
19.07
19.04
17.92
f1 (ppm)

## 3.4 Synthesis of $Cp_2Ti(OMs)_2$

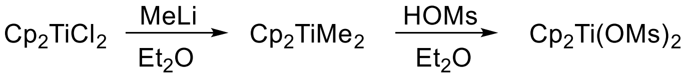


The synthesis is based on a procedure by *Luinstra*.[18] In a flame dried schlenk flask 4.98 g $Cp_2TiCl_2$ (20 mmol, 1.0 eq.) are dissolved 150 ml anhydrous $Et_2O$. Over a course of 10 min 31.3 ml MeLi (1.6 M in $Et_2O$, 50 mmol, 2.5 eq.) are added dropwise at 0 °C. The reaction is stirred for 0.5 h at this temperature and is then quenched by the careful addition of 120 ml 6% aqueous $NH_4Cl$ solution. The organic phase is separated, washed with $H_2O$ and brine to obtain a yellow stock solution which is used directly without further workup.

The stock solution is cooled to 0 °C and 4.04 g HOMs (42 mmol, 2.1 eq.) is added dropwise. The resulting mixture is stirred for 0.5 h at 0 °C and for 0.5 h at room temperature. The solution is filtrated and the precipitate is washed three times with toluene and twice with pentane. The solvent is removed under reduced pressure to obtain 5.05 g $Cp_2Ti(OMs)_2$ (13.7 mmol, 69%) as red needles. The catalyst is stored under Argon but weighted under air and securated again afterwards.

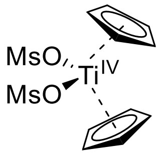


$C_{12}H_{16}O_6S_2Ti$
368,24 g/mol

**$^1$H-NMR (300 MHz, $C_6D_6$, RT): δ [ppm] =** 6.82 (s, 10H), 2.97 (s, 6H).
Analytical data is in accordance with literature.[19]

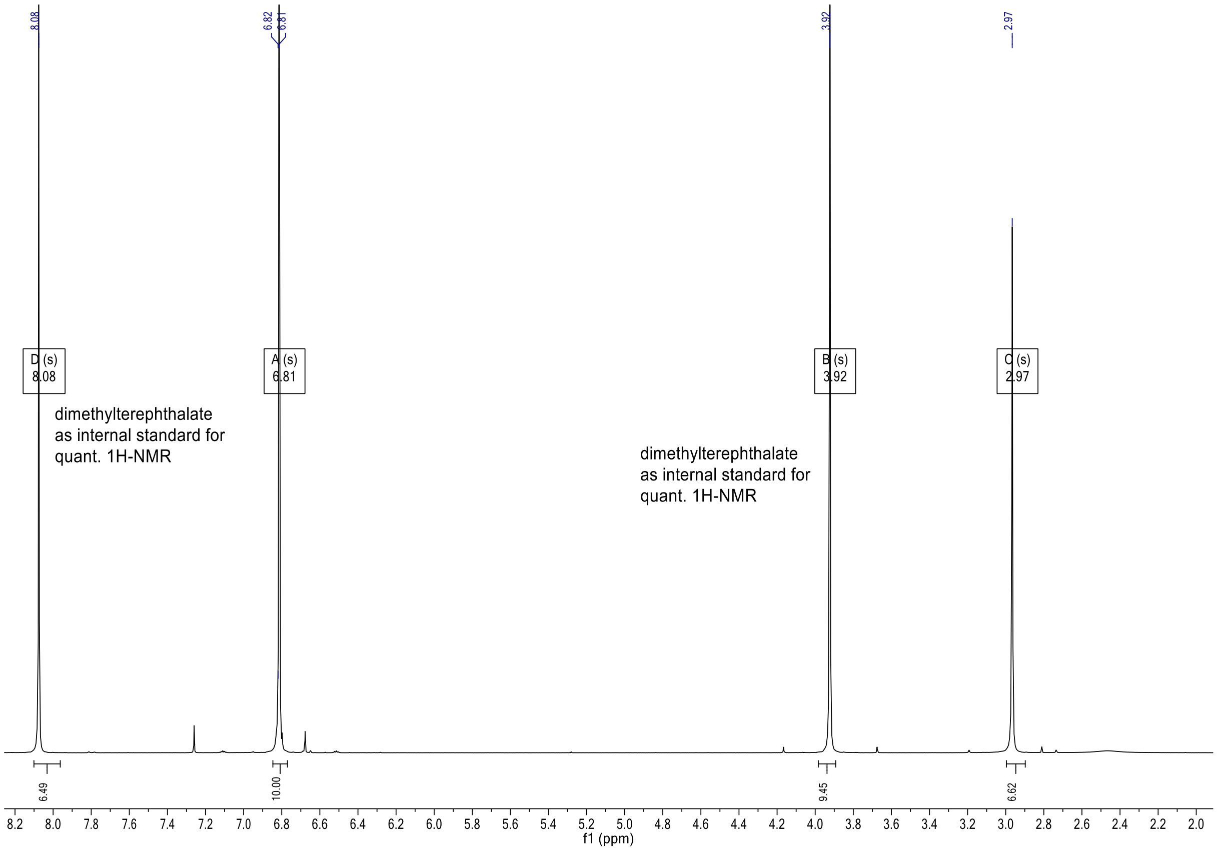


# 4. Titanocene catalyzed reactions

## 4.1 Titanocene catalyzed synthesis of acetals **2a-2i**

### 4.1.1 Synthesis of 2-phenyl-4-(propan-2-yl)-1,3-dioxolane **2a**

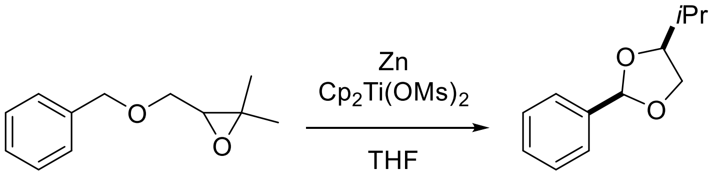


According to GP VII 21.3 mg $Cp_2Ti(OMs)_2$ (0.0578 mmol, 0.11 eq.), 6.7 mg zinc dust (0.102 mmol, 0.20 eq.) and 99.8 mg substrate **1a** (0.519 mmol, 1.00 eq.) are refluxed. Column chromatography ($SiO_2$, eluent: CH:EA, 97:3) afforded 66.2 mg **2a** (d.r. = 99:1, 0.346 mmol, 67%) as a colourless oil.

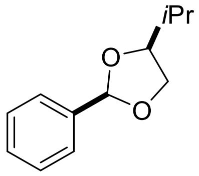

**2a**
$C_{12}H_{16}O_2$
192,26 g/mol

**R$_f$ =** 0.47 (5% EA, 95% CH). **$^1$H-NMR (400 MHz, $C_6D_6$, RT): δ [ppm] =** 7.62 – 7.58 (m, 2H, *o*-Ph), 7.20 – 7.15 (m, 2H, *m*-Ph), 7.14 – 7.09 (m, 1H, *p*-Ph), 5.79 (s, 1H, Ph-CH), 3.70 (dd, *J* = 7.0, 6.4 Hz, 1H, $CH_2$), 3.58 (*p*q, *J* = 6.9 Hz, 1H, *i*Pr-CH), 3.52 (*p*t, *J* = 6.9 Hz, 1H, $CH_2$), 1.62 (dqq, *J* = 7.2, 6.8, 6.8 Hz, 1H, $(CH_3)_2$-CH), 0.94 (d, *J* = 6.8 Hz, 3H, $CH_3$), 0.67 (d, *J* = 6.8 Hz, 3H, $CH_3$). **$^{13}$C-NMR (101 MHz, $C_6D_6$, RT): δ [ppm] =** 139.1, 129.2, 128.4, 127.2, 104.3, 82.6, 68.5, 31.9, 19.0, 18.4. **IR:** 2960, 2874, 1458, 1387, 1090, 1067, 1027, 1005, 977, 757, 696 $cm^{-1}$. **HRMS (ESI+):** *m/z* calculated for $C_{12}H_{17}O_2^+$: 193.1223 u, found: 193.1220 u.

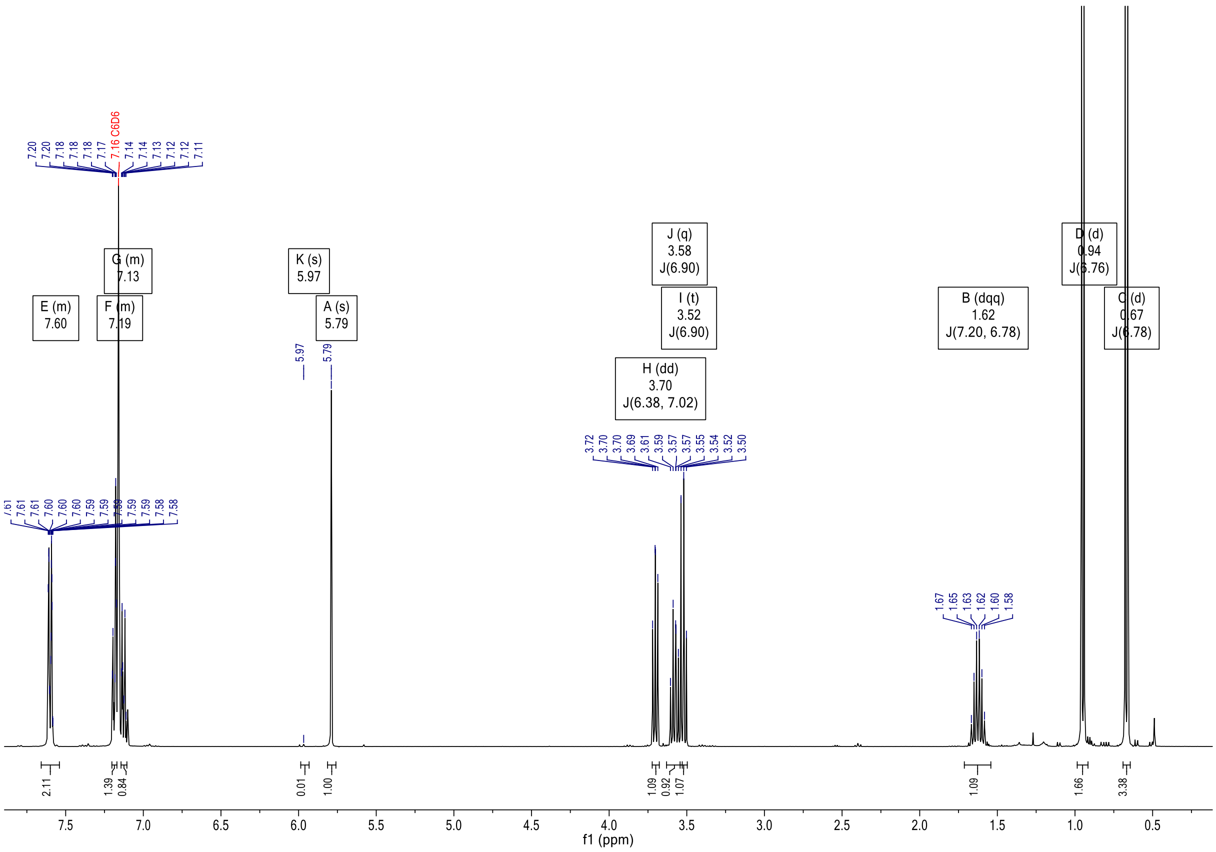

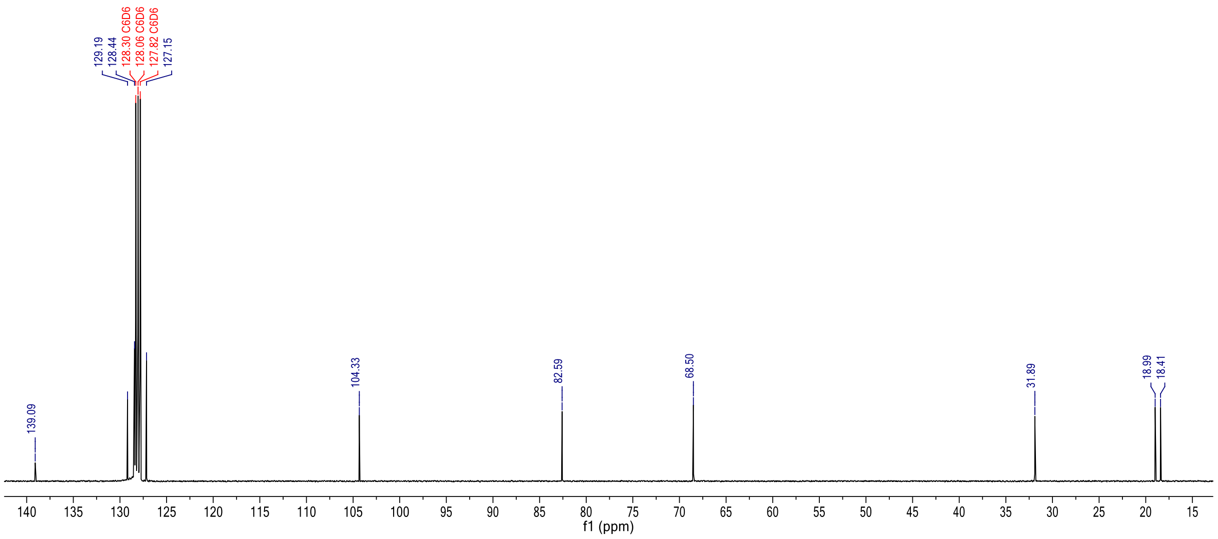


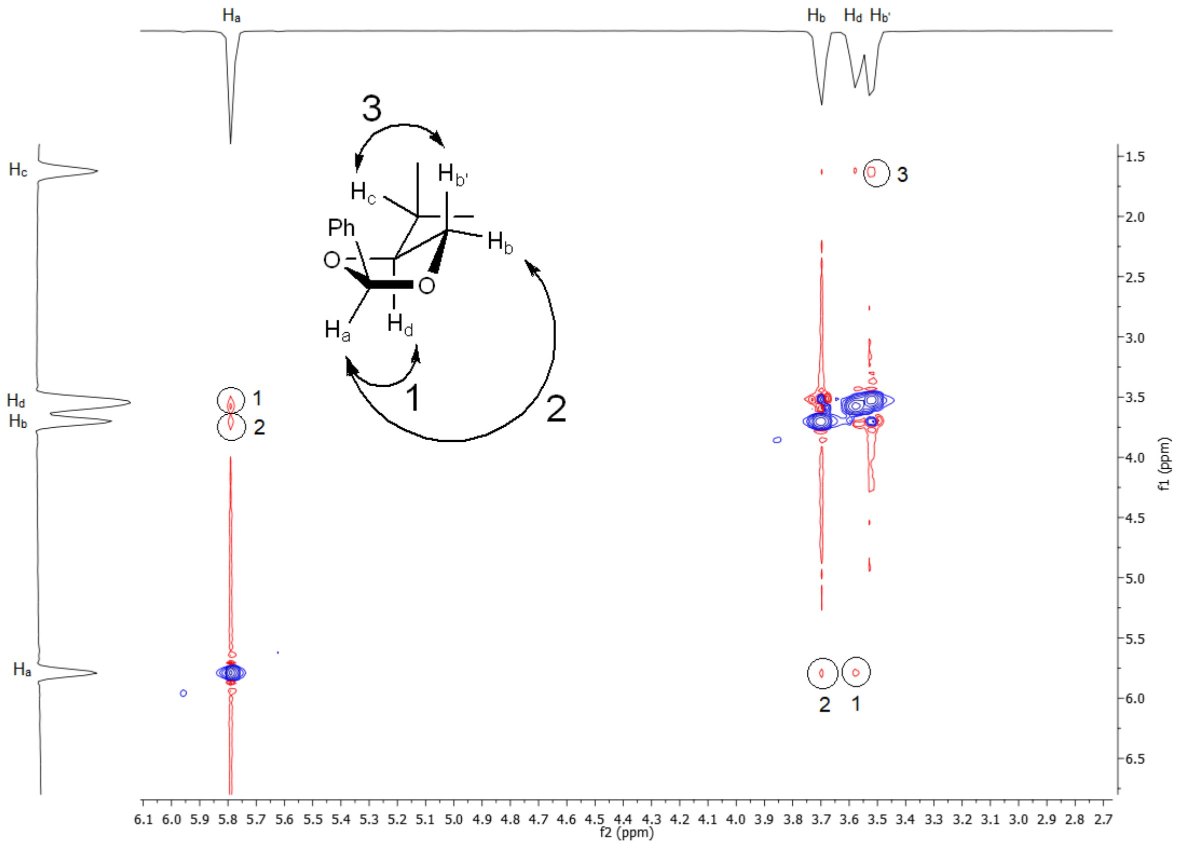


Coupling **1** indicates a *cis*–configuration of the aryl group and the isopropyl group. Furthermore, $H_b$ is *cis*-configurated to $H_d$ (see coupling constant in $^1$H-spectra). For

this reason, coupling 2 between $H_a$ and $H_b$ proves the relative configuration as depicted.

4.1.2 Synthesis of 2-(4-methylphenyl)-4-(propan-2-yl)-1,3-dioxolane **2b**

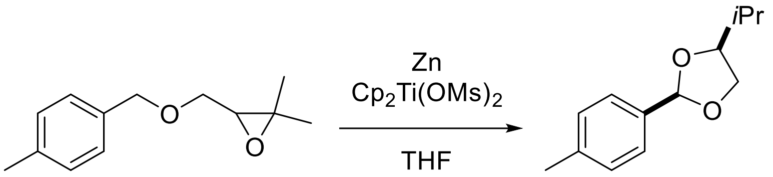


According to GP VII 35.8 mg $Cp_2Ti(OMs)_2$ (0.0972 mmol, 0.10 eq.), 12.7 mg zinc dust (0.194 mmol, 0.20 eq.) and 201.8 mg substrate **1b** (0.9782 mmol, 1.00 eq.) are refluxed. Column chromatography ($SiO_2$, eluent: CH:EA, 97:3) afforded 160.4 mg **2b** (d.r. = 99:1, 0.816 mmol, 83%) as a colourless oil.

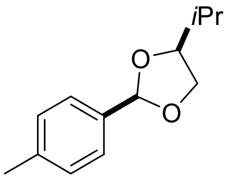


**2b**
$C_{13}H_{18}O_2$
206,29 g/mol

**$R_f$ =** 0.71 (20% EA, 80% CH). **$^1$H-NMR (500 MHz, $C_6D_6$, RT): δ [ppm] =** 7.55 (d, *J* = 8.0 Hz, 2H), 7.02 (d, *J* = 8.0 Hz, 2H), 5.82 (s, 1H), 3.72 (dd, *J* = 7.0, 6.4 Hz, 1H), 3.60 (*p*q, *J* = 6.9 Hz, 1H), 3.55 (*p*t, *J* = 7.0 Hz, 1H), 2.08 (s, 3H), 1.65 (dqq, *J* = 7.1, 6.8, 6.8 Hz, 1H), 0.97 (d, *J* = 6.8 Hz, 3H), 0.69 (d, *J* = 6.8 Hz, 3H). **$^{13}$C-NMR (126 MHz, $C_6D_6$, RT): δ [ppm] =** 138.8, 136.3, 129.1, 127.2, 104.4, 82.5, 68.5, 31.9, 21.2, 19.0, 18.4. **IR:** 2959, 2873, 1469, 1386, 1369, 1223, 1178, 1079, 1021, 1004, 978, 940, 811 $cm^{-1}$. **HRMS (ESI+) :** *m/z* calculated for $C_{13}H_{19}O_2^+$: 207.1380 u, found: 207.1384 u.

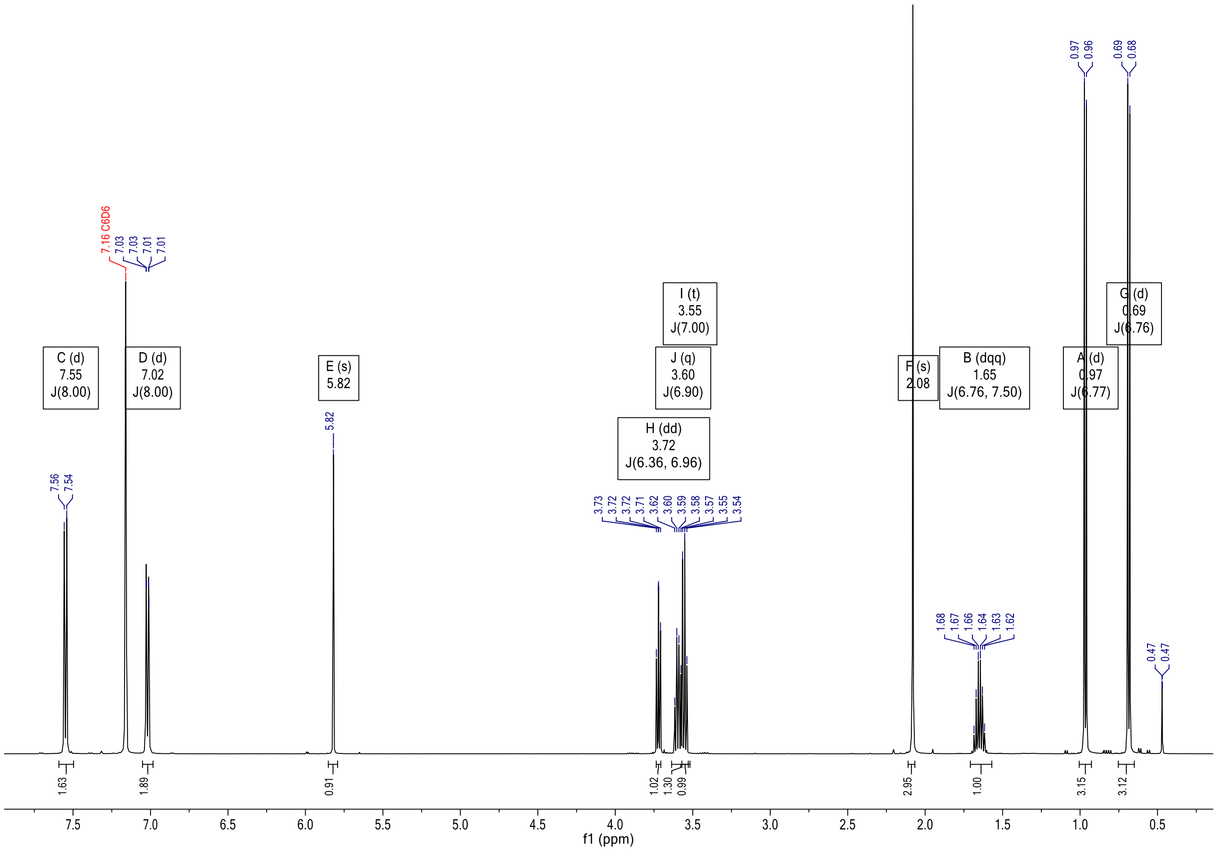
7.16 C6D6
7.03
7.03
7.01
7.01
C (d) 7.55 J(8.00)
D (d) 7.02 J(8.00)
E (s) 5.82
I (t) 3.55 J(7.00)
J (q) 3.60 J(6.90)
H (dd) 3.72 J(6.36, 6.96)
F (s) 2.08
B (dqq) 1.65 J(6.76, 7.50)
A (d) 0.97 J(6.77)
G (d) 0.69 J(6.76)
0.97
0.96
0.69
0.68
7.56
7.54
5.82
3.73
3.72
3.72
3.71
3.62
3.60
3.59
3.58
3.57
3.55
3.54
1.68
1.67
1.66
1.64
1.63
1.62
0.47
0.47
1.63
1.89
0.91
1.02
1.30
0.99
2.95
1.00
3.15
3.12
f1 (ppm)

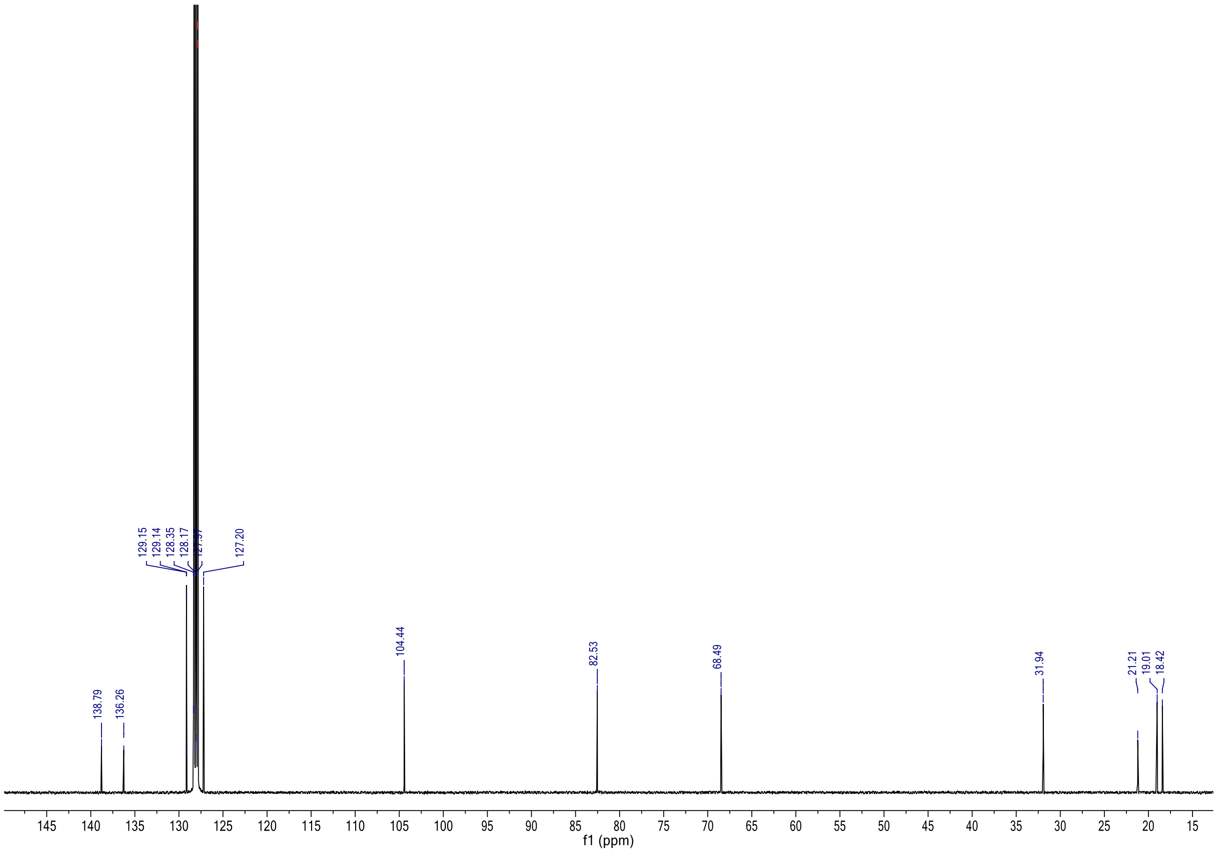
129.15
129.14
128.35
128.17
127.97
127.20
104.44
82.53
68.49
31.94
21.21
19.01
18.42
138.79
136.26
f1 (ppm)

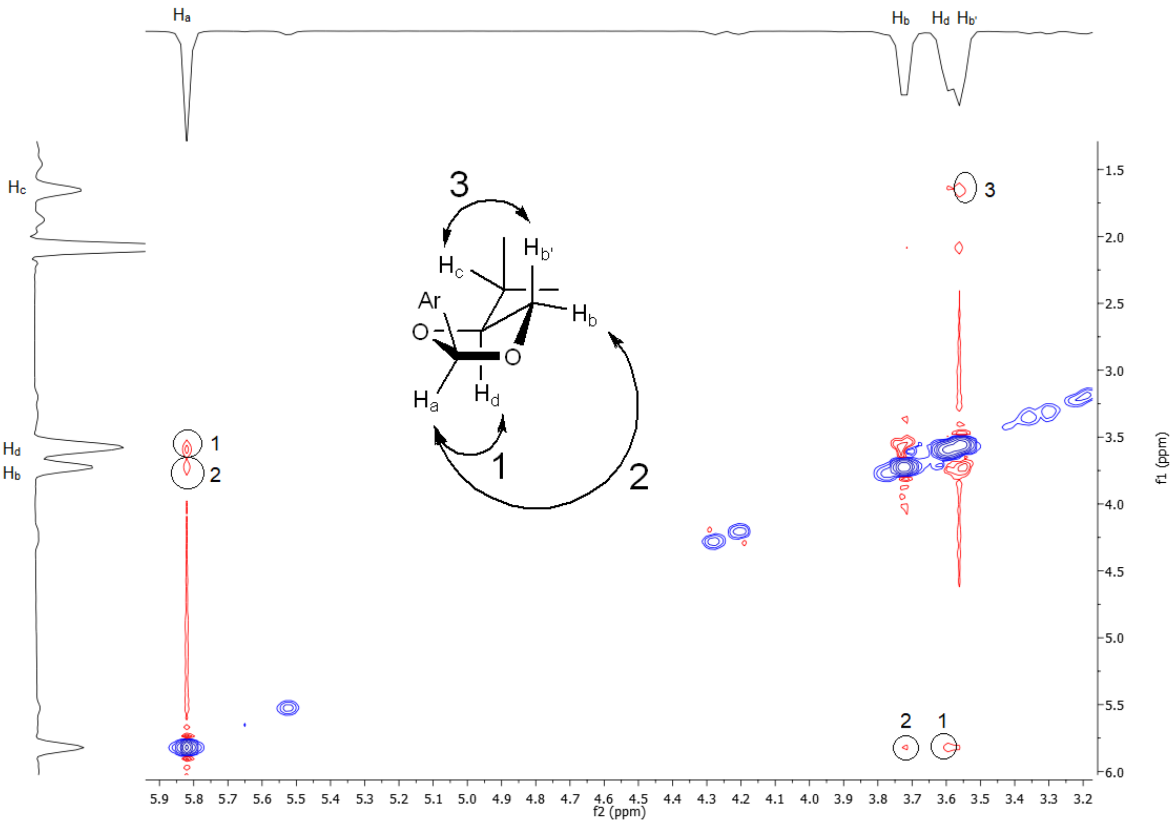


Coupling **1** indicates a *cis*–configuration of the aryl group and the isopropyl group. Furthermore, Hb is *cis*-configurated to Hd (see coupling constant in $^1$H-spectra). For this reason coupling **2** between Ha and Hb proves the relative configuration as depicted.

### 4.1.3 Synthesis of 2-([1,1'-biphenyl]-4-yl)-4-(propan-2-yl)-1,3-dioxolane **2c**

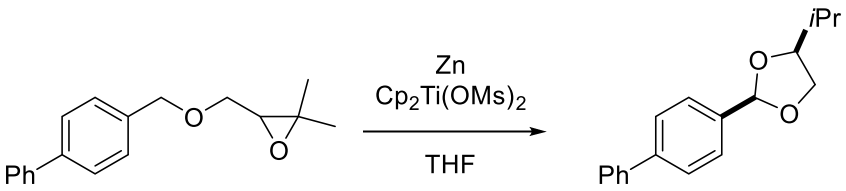


According to GP VII 27.6 mg $Cp_2Ti(OMs)_2$ (0.0750 mmol, 0.19 eq.), 9.8 mg zinc dust (0.150 mmol, 0.38 eq.) and 105 mg substrate **1c** (0.391 mmol, 1.00 eq.) are reacted. Column chromatography ($SiO_2$, eluent: CH:EA, 97:3) afforded 69.6 mg **2c** (d.r. = 99:1, 0.259 mmol, 66%) as a colourless oil.

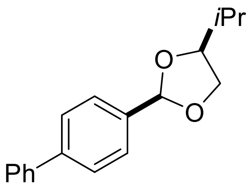

**2c**
$C_{18}H_{20}O_2$
268,36 g/mol

**$R_f$ =** 0.73 (30% EA, 70% CH). **$^{1}$H-NMR (500 MHz, $C_6D_6$, RT): δ [ppm] =** 7.70 – 7.62 (m, 2H), 7.52 – 7.46 (m, 2H), 7.46 – 7.41 (m, 2H), 7.26 – 7.14 (m, 2H), 7.18 – 7.07 (m, 1H), 5.85 (s, 1H), 3.74 (dd, *J* = 7.2, 6.5 Hz, 1H), 3.62 (*p*q, *J* = 6.9 Hz, 1H), 3.57 (*p*t, *J* = 7.0 Hz, 1H), 1.68 (dqq, *J* = 7.1, 6.8, 6.8 Hz, 1H), 0.99 (d, *J* = 6.8 Hz, 3H), 0.77 (d, *J* = 6.8 Hz, 3H). **$^{13}$C-NMR (126 MHz, $C_6D_6$, RT): δ [ppm] =** 142.4, 141.4, 138.0, 129.0, 127.7, 127.6, 127.7, 127.4, 104.2, 82.7, 68.6, 32.0, 19.0, 18.4. **IR:** 2956, 2910, 2867, 1598, 1487, 1470, 1448, 1417, 1384, 1364, 1310, 1116, 1084, 1038, 1007, 967, 948, 915, 865, 832, 763, 743, 721, 695, 628, 563, 522, 489 $cm^{-1}$. **HRMS (ESI+):** *m/z* calculated for $C_{18}H_{21}O_2^+$: 269.1536 u, found: 269.1542 u.

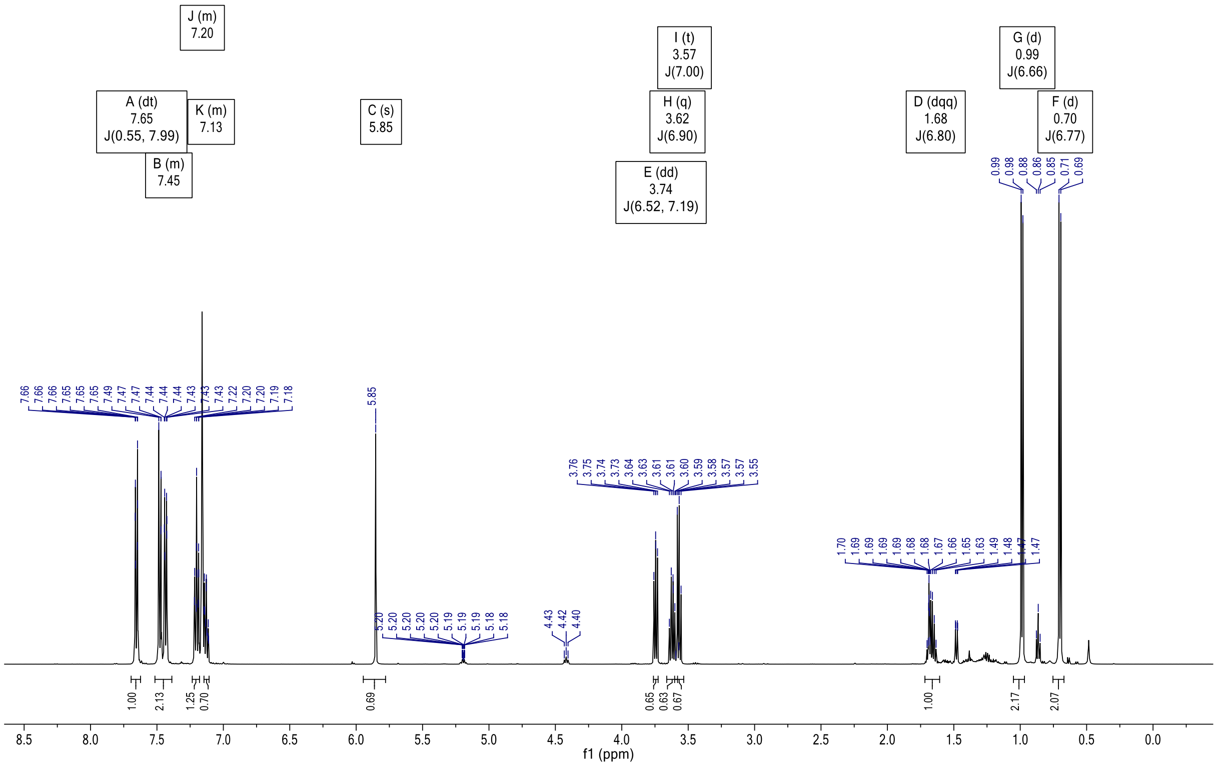
J (m)
7.20
A (dt)
7.65
J(0.55, 7.99)
K (m)
7.13
B (m)
7.45
C (s)
5.85
I (t)
3.57
J(7.00)
H (q)
3.62
J(6.90)
E (dd)
3.74
J(6.52, 7.19)
D (dqq)
1.68
J(6.80)
G (d)
0.99
J(6.66)
F (d)
0.70
J(6.77)
1.00
2.13
1.25
0.70
0.69
0.65
0.63
0.67
1.00
2.17
2.07
f1 (ppm)

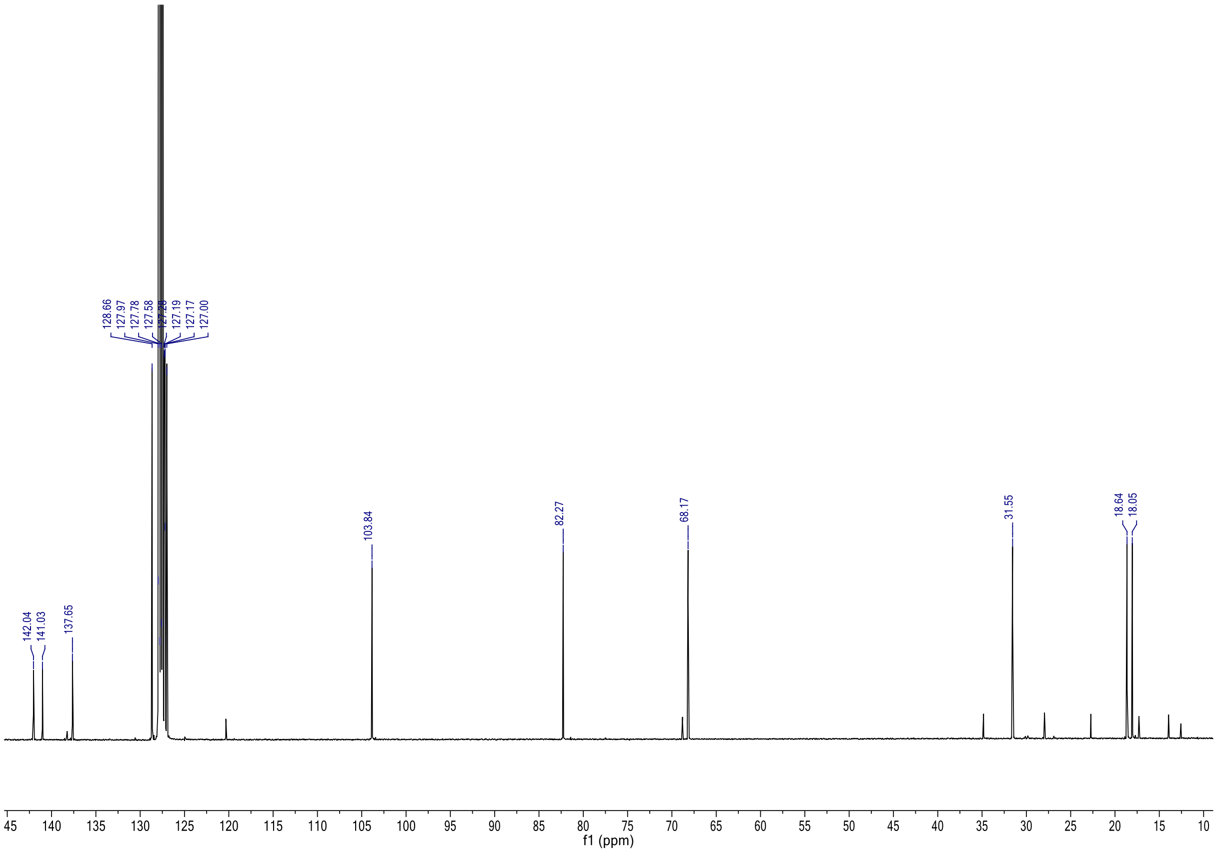
142.04
141.03
137.65
128.66
127.97
127.78
127.58
127.28
127.19
127.17
127.00
103.84
82.27
68.17
31.55
18.64
18.05
f1 (ppm)

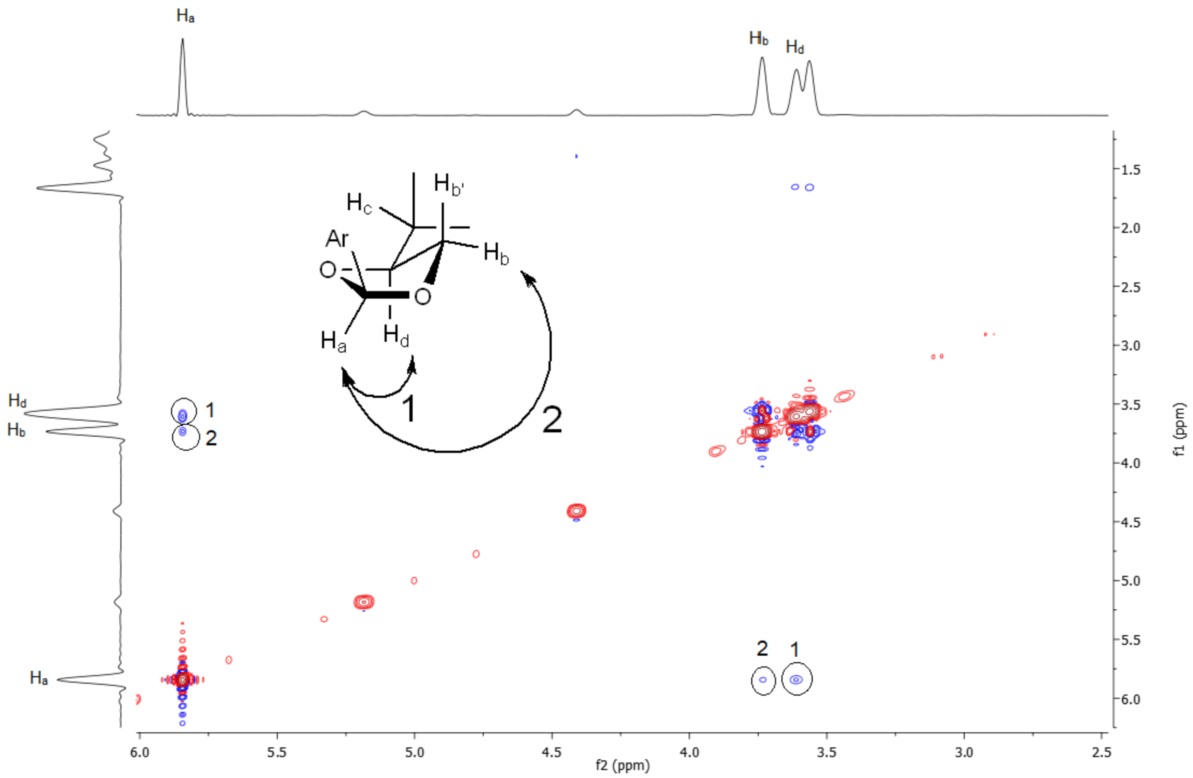


Coupling **1** indicates a *cis*–configuration of the aryl group and the isopropyl group. Furthermore, $H_b$ is *cis*-configurated to $H_d$ (see coupling constant in $^{1}$H-spectra). For this reason coupling **2** between $H_a$ and $H_b$ proves the relative configuration as depicted.

### 4.1.4 Synthesis of 2-(4-methoxyphenyl)-4-(propan-2-yl)-1,3-dioxolane **2d**

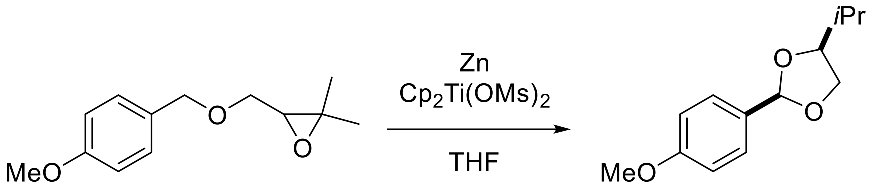


According to GP VII 16.5 mg $Cp_2Ti(OMs)_2$ (0.0448 mmol, 0.10 eq.), 6.3 mg zinc dust (0.0964 mmol, 0.21 eq.) and 101.5 mg substrate **1d** (0.456 mmol, 1.00 eq.) are refluxed. Column chromatography ($SiO_2$, eluent: CH:EA, 97:3) afforded 85.3 mg **2d** (d.r. = 94:6, 0.384 mmol, 84%) as a colourless oil.

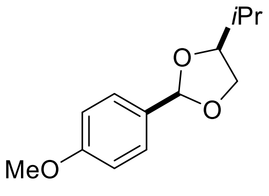


**2d**
$C_{13}H_{18}O_3$
222,28 g/mol

**$R_f$** = 0.53 (30% EA, 70% CH). ***cis*-Diastereomer (major): $^{1}$H-NMR (500 MHz, $C_6D_6$, RT): δ [ppm] =** 7.54 (d, *J* = 8.6 Hz, 2H), 6.80 (d, *J* = 8.6 Hz, 2H), 5.80 (s, 1H), 3.73 (*p*t, *J* = 6.7 Hz, 1H), 3.61 (*p*q, *J* = 6.9 Hz, 1H), 3.57 (*p*t, *J* = 6.9 Hz, 1H), 3.26 (s, 3H), 1.72 – 1.62 (m, 1H), 0.98 (d, *J* = 6.7 Hz, 3H), 0.70 (d, *J* = 6.8 Hz,

3H). **$^{13}$C-NMR (125 MHz, $C_6D_6$, RT): δ [ppm] =** 160.8, 131.2, 128.6, 114.0, 104.4, 82.5, 68.5. 54.8, 32.0, 19.0, 18.4. ***trans*-Diastereomer (minor): $^1$H-NMR (500 MHz, $C_6D_6$, RT): δ [ppm] =** 7.54 (d, *J* = 8.6 Hz, 2H), 6.80 (d, *J* = 8.6 Hz, 2H), 5.97 (s, 1H), 3.93 (dd, *J* = 8.1, 6.2 Hz, 1H), 3.67 (*p*td *J* = 7.8, 6.2 Hz, 1H), 3.43 (*p*t, *J* = 7.8 Hz, 1H), 3.27 (s, 3H), 1.67-1.56 (m, 1H), 0.99 (d, *J* = 6.7 Hz, 3H), 0.63 (d, *J* = 6.8 Hz, 3H). **$^{13}$C-NMR (125 MHz, $C_6D_6$, RT): δ [ppm] =** 160.7, 132.0, 128.6, 114.0, 103.9, 81.7, 69.3, 54.8, 32.0, 19.3, 18.0. **IR:** 2959, 2874, 1614, 1516, 1464, 1386, 1302, 1246, 1170, 1112, 1077, 1032, 1003, 977, 827, 591 $cm^{-1}$. **HRMS (ESI+):** *m/z* calculated for $C_{13}H_{19}O_3^+$: 223.1329 u, found: 223.1323 u.

.

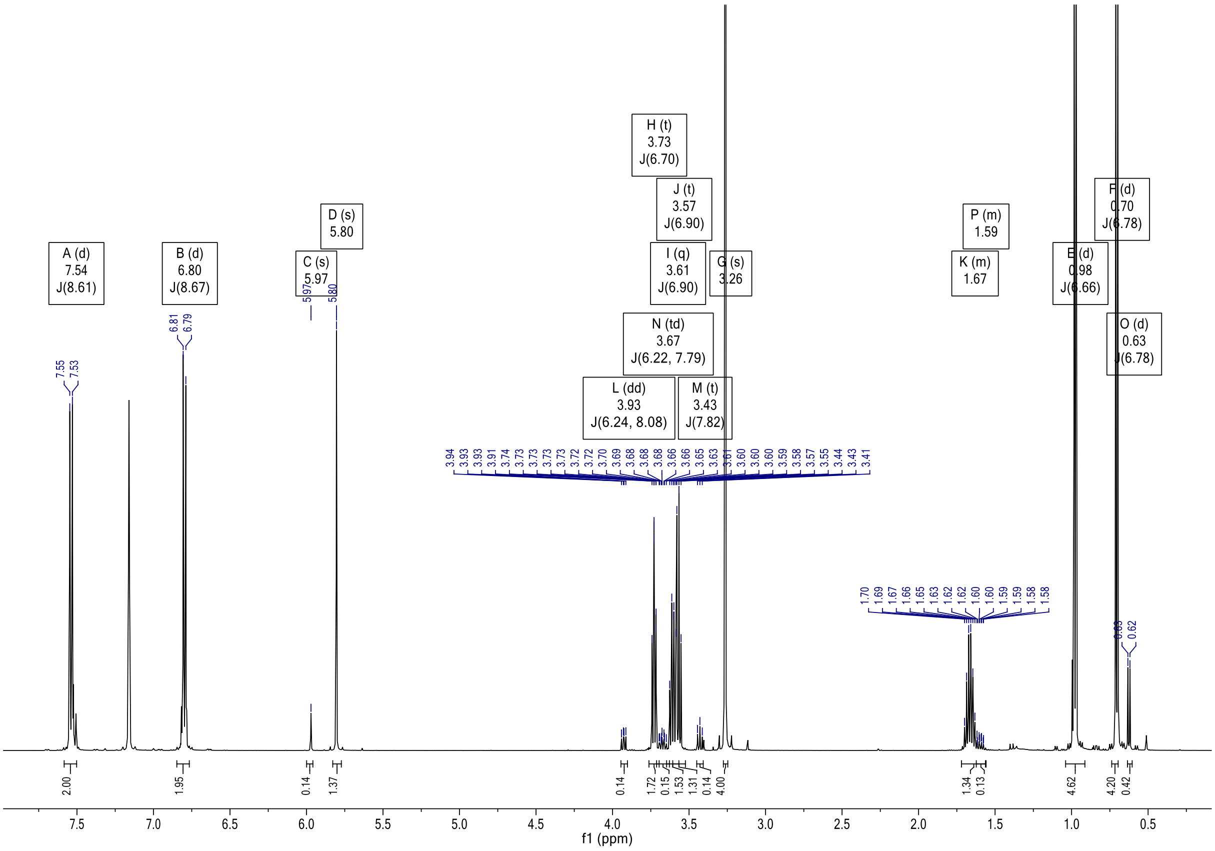

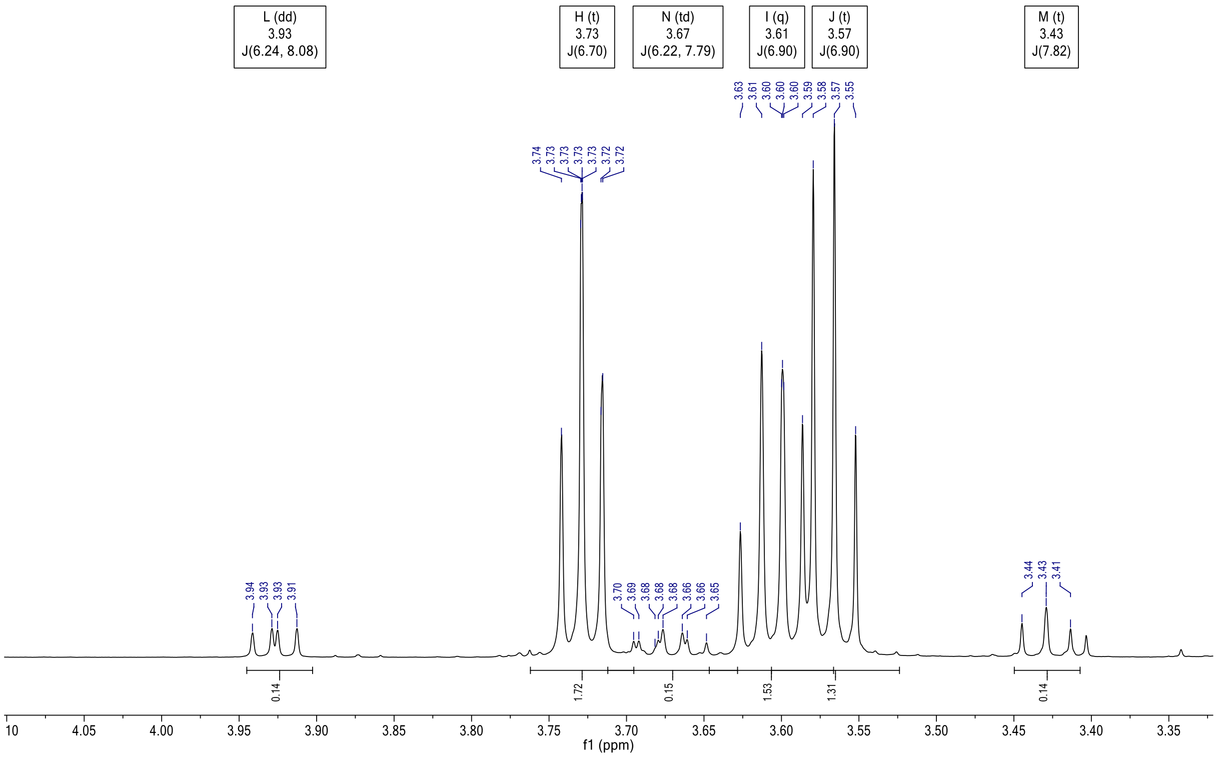
L (dd)
3.93
J(6.24, 8.08)
H (t)
3.73
J(6.70)
N (td)
3.67
J(6.22, 7.79)
I (q)
3.61
J(6.90)
J (t)
3.57
J(6.90)
M (t)
3.43
J(7.82)
0.14
1.72
0.15
1.53
1.31
0.14
f1 (ppm)

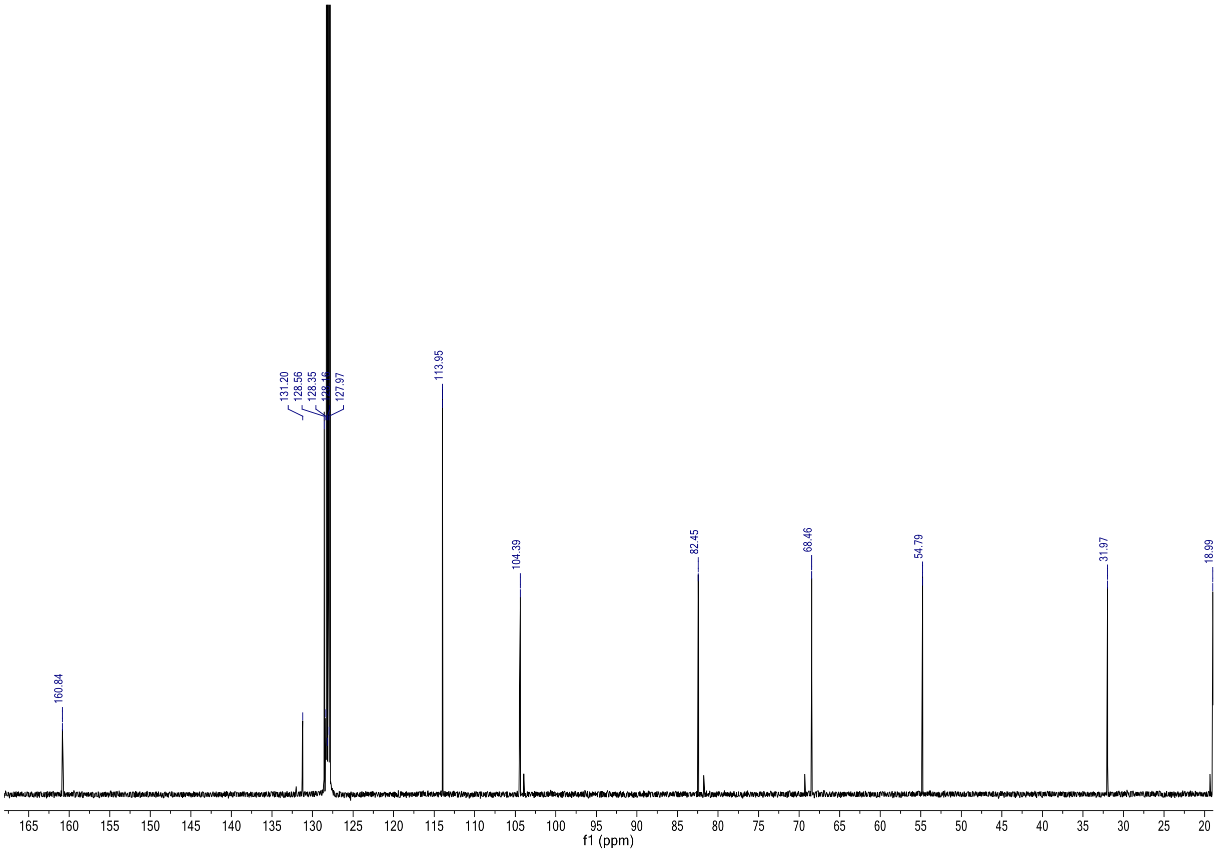
160.84
131.20
128.56
128.35
128.16
127.97
113.95
104.39
82.45
68.46
54.79
31.97
18.99
f1 (ppm)

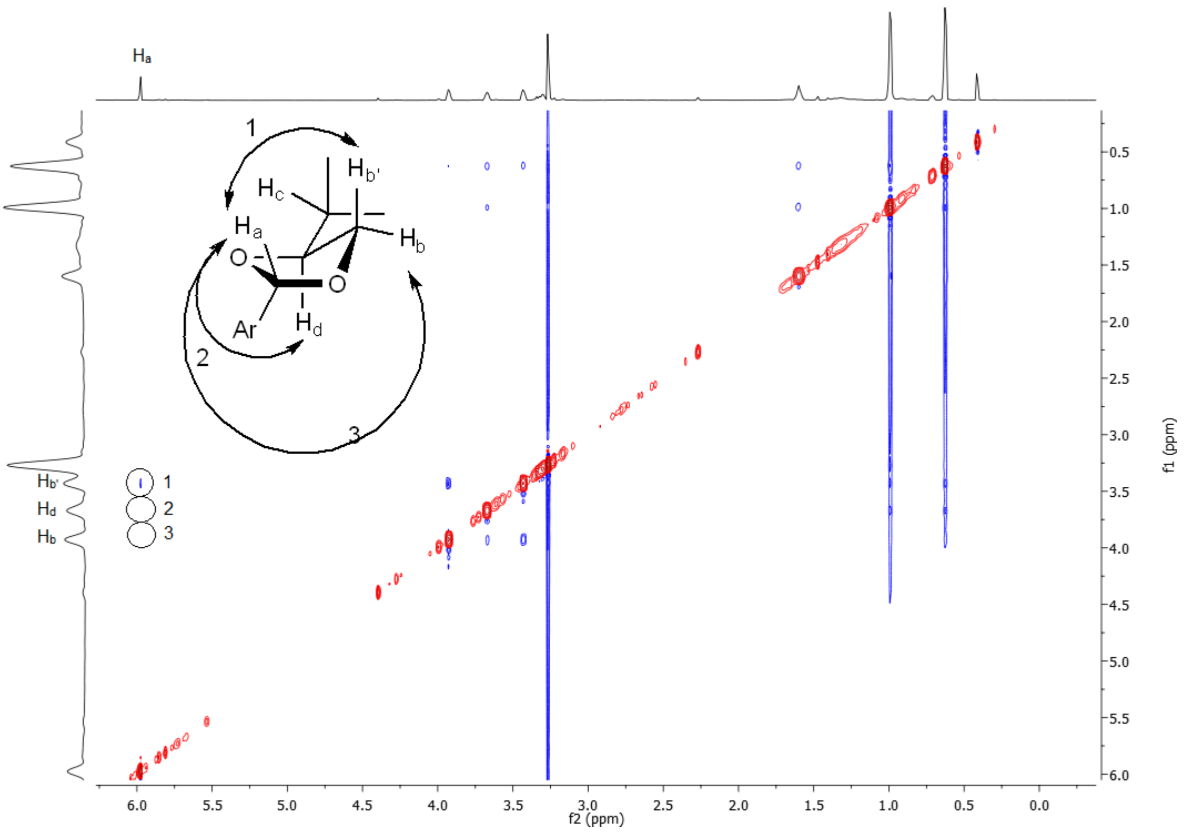


Coupling **1** indicates a 2,4-*trans*-configuration since $H_{b'}$ is *trans*-configurated to $H_d$ (see coupling constant $^{1}$H-spectra). The missing couplings **2** and **3** support this relative configuration.

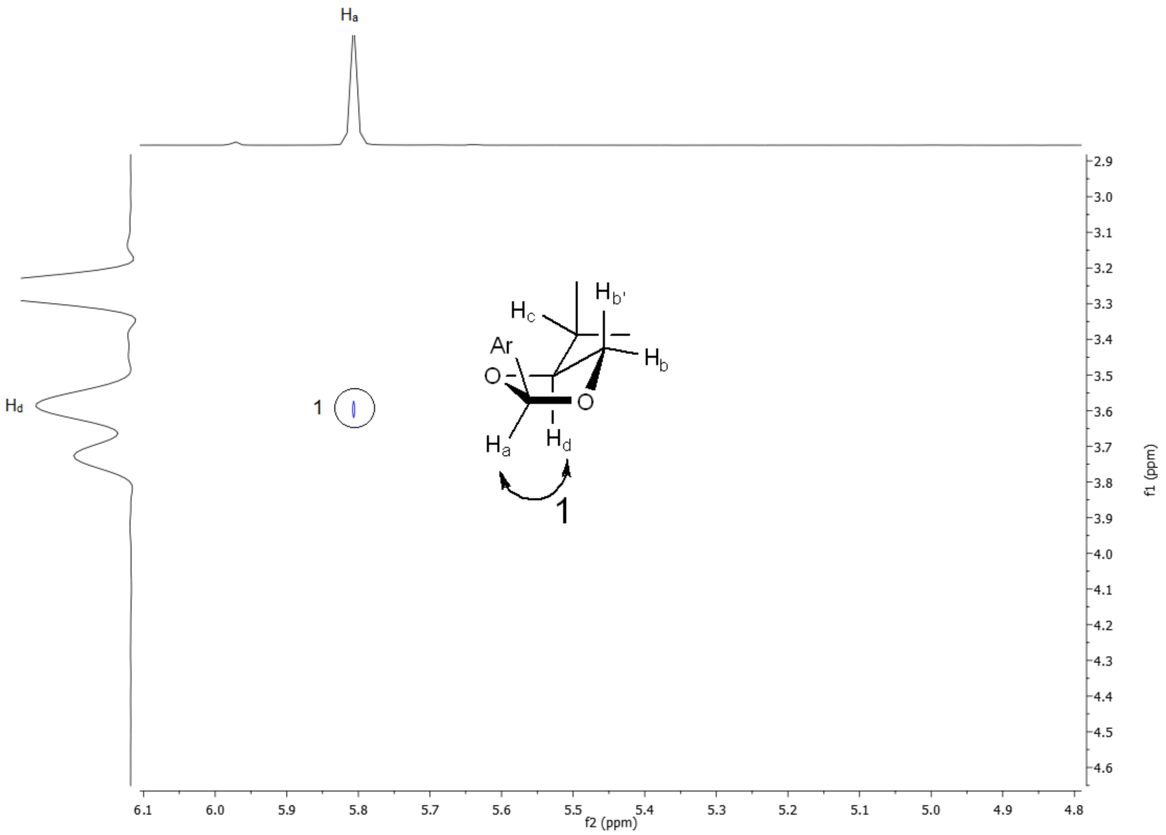

Coupling **1** between $H_a$ and $H_d$ proves the relative configuration of both stereocenters.

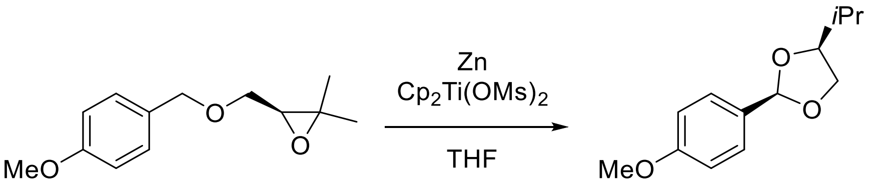


According to GP VII 15.9 mg $Cp_2Ti(OMs)_2$ (0.0432 mmol, 0.10 eq.), 5.4 mg zinc dust (0.0826 mmol, 0.20 eq.) and 93.7 mg substrate (e.r. = 99:1, 0.422 mmol, 1.00 eq.) are refluxed. Column chromatography ($SiO_2$, eluent: CH:EA, 97:3) afforded 79.1 mg **2d** (d.r. = 93:7, e.r. = 99:1, 0.315 mmol, 84%) as a colourless oil. The e.r. of the substrate and the product is determined by HPLC. $^1$H-NMR and $^{13}$C-NMR data is in accordance with that of the racemate.

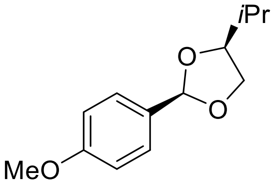


**2d**
$C_{13}H_{18}O_3$
222,28 g/mol

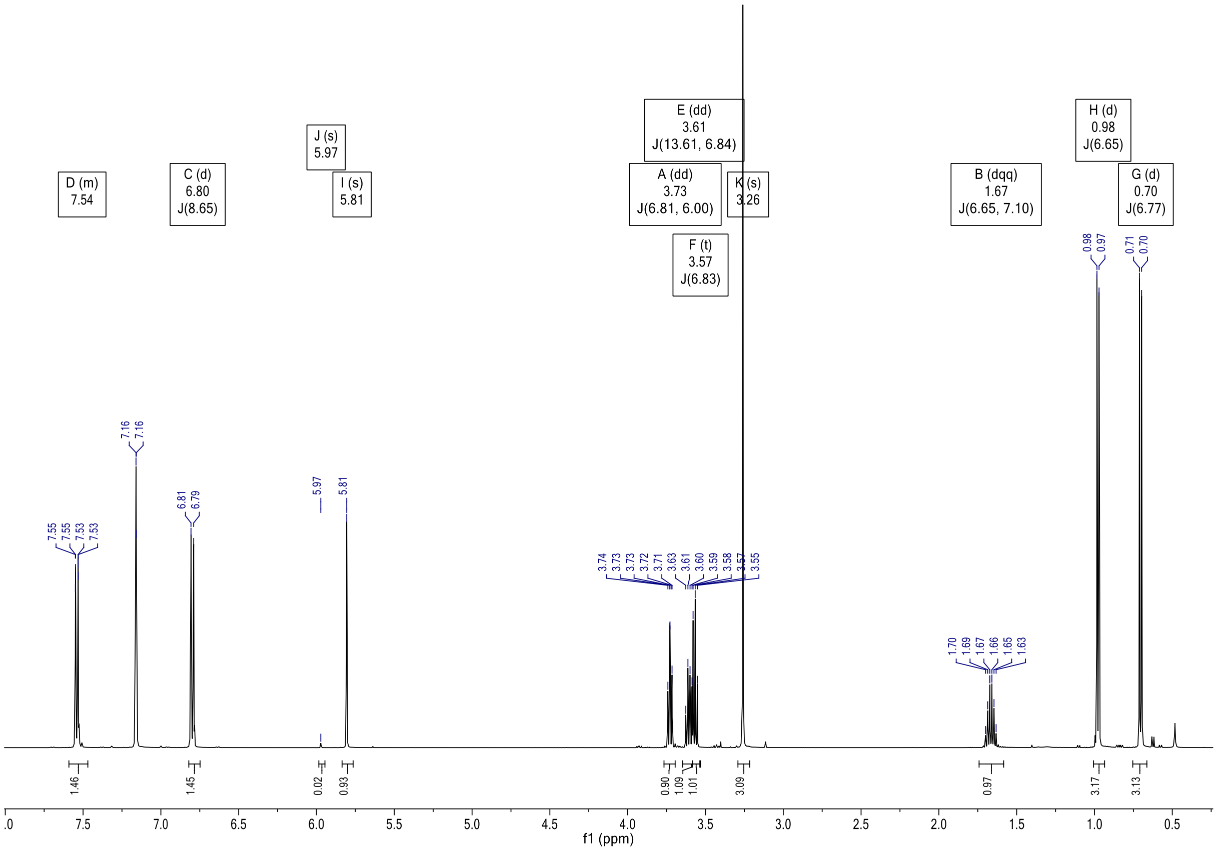

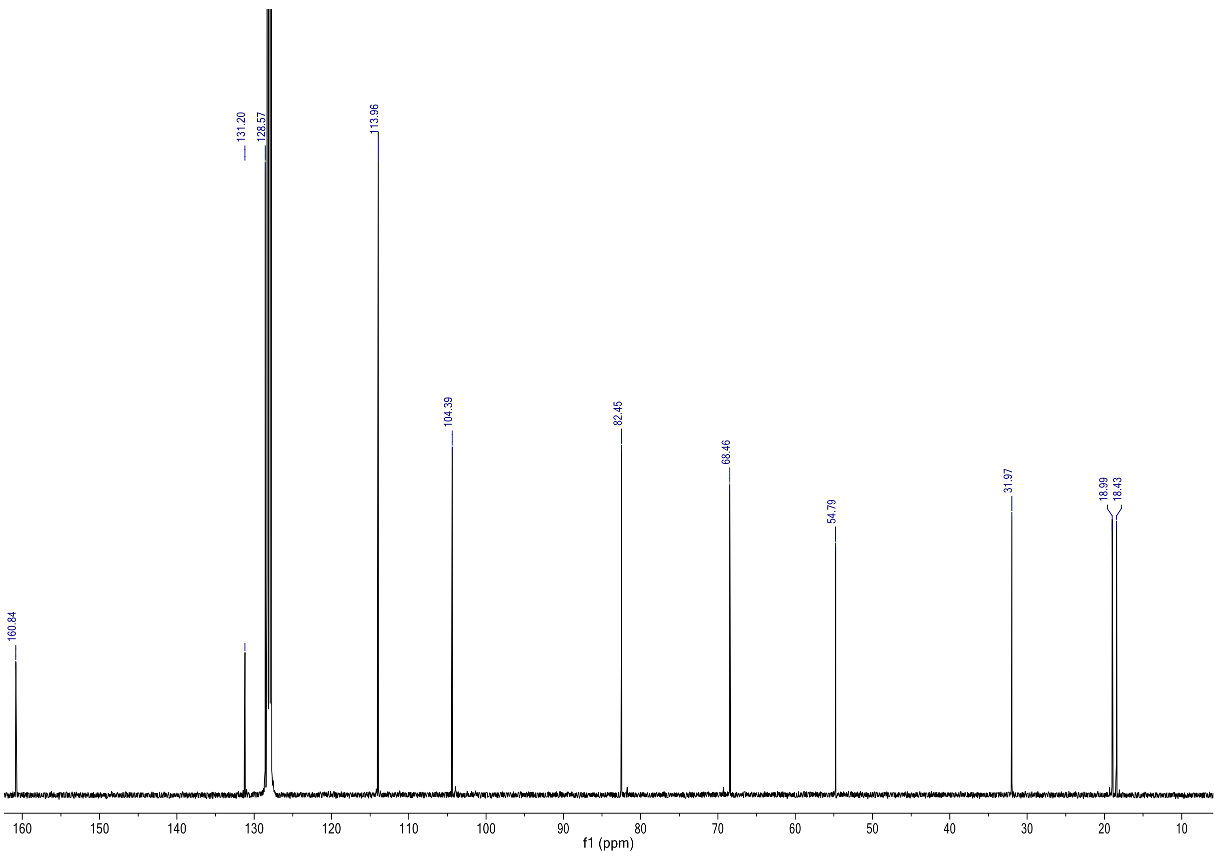


4.1.5 Synthesis of 2-(3-methoxyphenyl)-4-(propan-2-yl)-1,3-dioxolane **2e**

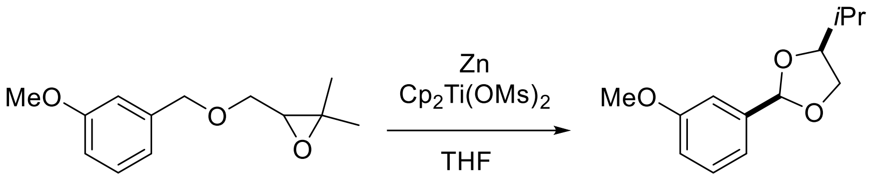


According to GP VII 16.8 mg $Cp_2Ti(OMs)_2$ (0.0456 mmol, 0.10 eq.), 5.9 mg zinc dust (0.0902 mmol, 0.20 eq.) and 99.7 mg substrate **1e** (0.449 mmol, 1.00 eq.) are refluxed. Column chromatography ($SiO_2$, eluent: CH:EA, 97:3) afforded 80.4 mg **2e** (d.r. = 99:1, 0.362 mmol, 81%) as a colourless oil.

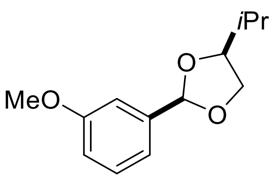


**2e**
$C_{13}H_{18}O_3$
222,28 g/mol

**$R_f$ =** 0.64 (30% EA, 70% CH). **$^1$H-NMR (300 MHz, $C_6D_6$, RT): δ [ppm] =** 7.31 (dd, *J* = 2.7, 1.5 Hz, 1H), 7.26 (d*p*t, *J* = 7.6, 1.4, 1.0 Hz, 1H), 7.12 (*p*t, *J* = 8.2, 7.6 Hz, 1H), 6.79 (ddd, *J* = 8.2, 2.6, 1.1 Hz, 1H), 5.81 (s, 1H), 3.74 – 3.68 (m, 1H), 3.64 – 3.47 (m, 2H), 3.31 (s, 3H), 1.63 (dqq, *J* = 7.1, 6.7, 6.7 Hz, 1H), 0.96 (d, *J* = 6.7 Hz, 3H), 0.67 (d, *J* = 6.7 Hz, 3H). **$^{13}$C-NMR (75 MHz, $C_6D_6$, RT): δ [ppm] =** 160.3, 140.7, 129.6, 119.4, 115.1, 112.6, 104.2, 82.6, 68.5, 54.7, 31.9, 19.0, 18.4. **IR:** 2959, 2874, 1604, 1589, 1491, 1460, 1386, 1369, 1284, 1263,

1171, 1160, 1096, 1070, 1043, 1004, 979, 861, 770, 723, 693 $cm^{-1}$. **HRMS (ESI+):** *m/z* calculated for $C_{13}H_{19}O_3^+$: 223.1329 u, found: 223.1323 u.

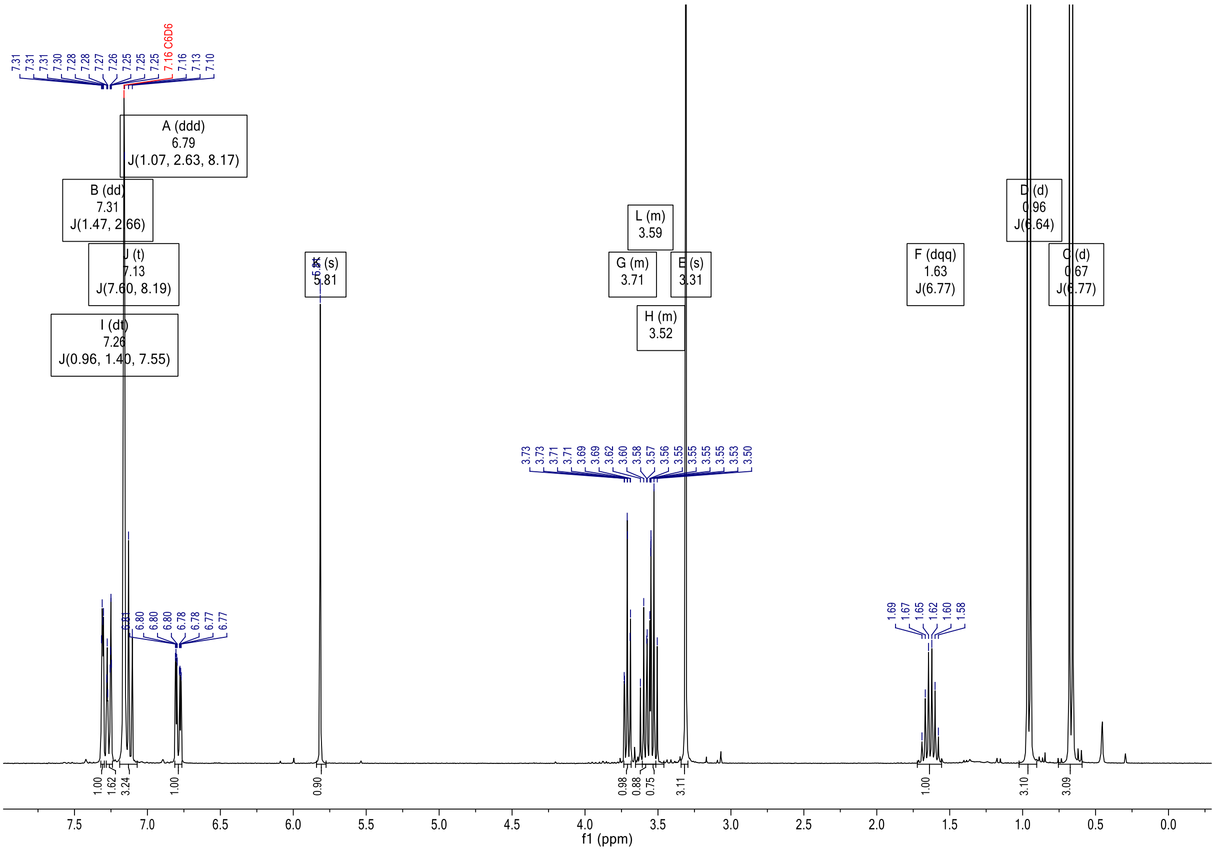

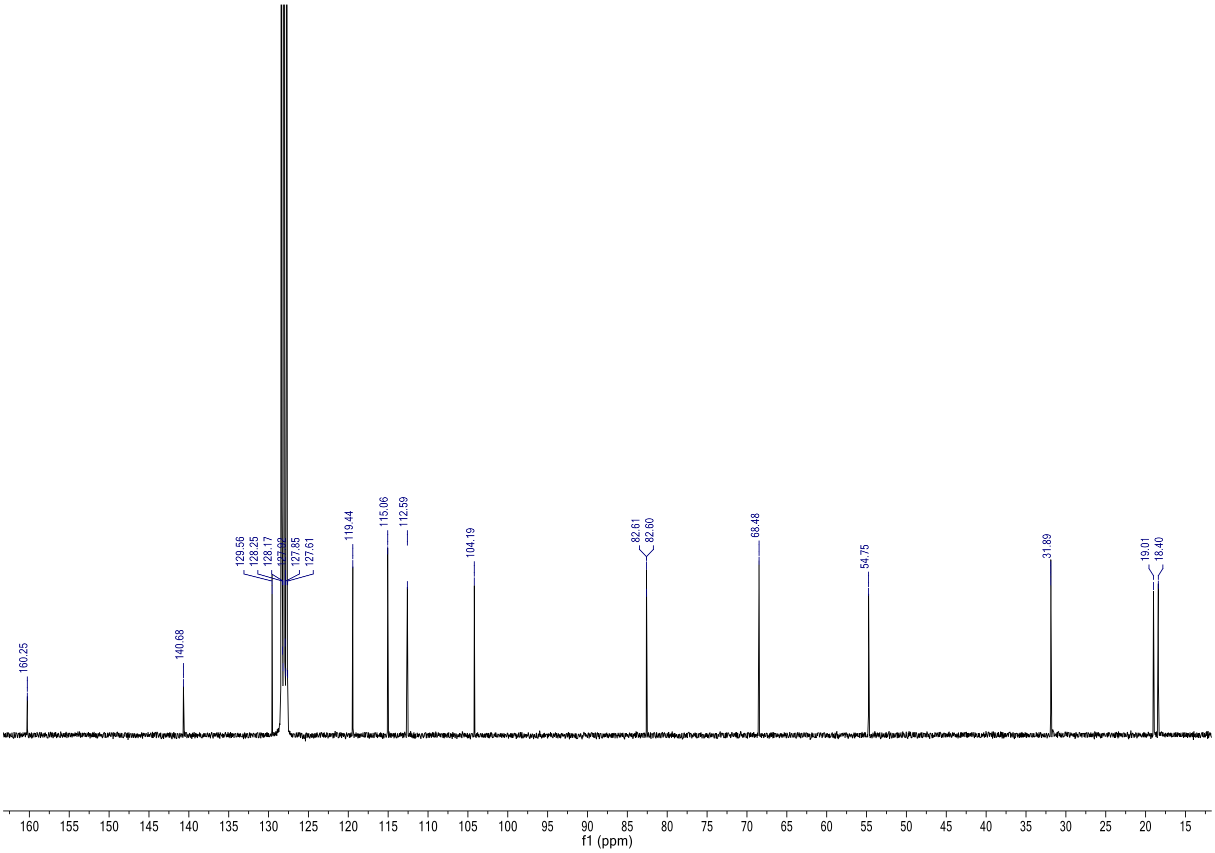

4.1.6 Synthesis of 4-(propan-2-yl)-2-(3,4,5-trimethoxyphenyl)-1,3-dioxolane **2f**

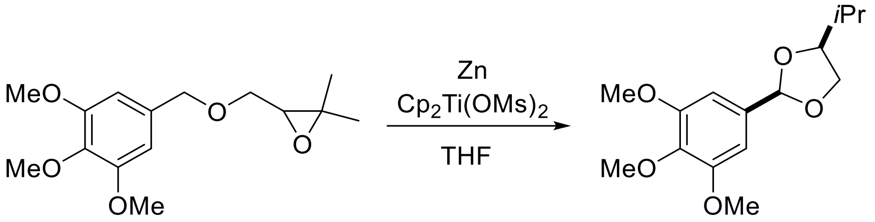

According to GP VII 13.2 mg $Cp_2Ti(OMs)_2$ (0.0358 mmol, 0.10 eq.), 4.7 mg zinc dust (0.0719 mmol, 0.20 eq.) and 99.5 mg substrate **1f** (0.352 mmol, 1.00 eq.) are refluxed. Column chromatography ($SiO_2$, eluent: CH:EA, 97:3) afforded 75.4 mg **2f** (d.r. = 99:1, 0.267 mmol, 73%) as a colourless oil.

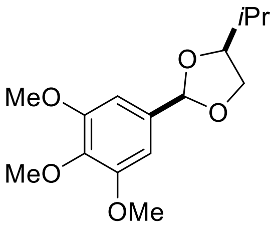

**2f**

$C_{15}H_{22}O_5$

282,34 g/mol

**$R_f$ =** 0.47 (30% EA, 70% CH). **$^1$H-NMR (300 MHz, $C_6D_6$, RT): δ [ppm] =** 6.90 (s, 2H), 5.82 (s, 1H), 3.83 (s, 3H), 3.80 – 3.74 (m, 1H), 3.69 – 3.56 (m, 2H), 3.41 (s, 6H), 1.70 (dqq, *J* = 7.1, 6.7, 6.7 Hz, 1H), 1.00 (d, *J* = 6.7 Hz, 3H), 0.72 (d, *J* = 6.7 Hz, 3H). **$^{13}$C-NMR (75 MHz, $C_6D_6$, RT): δ [ppm] =** 154.2, 140.1, 134.1, 104.8, 104.4, 82.6, 68.5, 60.5, 55.8, 32.0, 19.0, 18.4. **IR:** 2958, 2875, 1594, 1507, 1461, 1423, 1398, 1385, 1370, 13330, 1231, 1158, 1123, 1090, 1037, 1004, 834, 713 $cm^{-1}$. **HRMS (APCI):** *m/z* calculated for $C_{15}H_{23}O_5^+$: 283.1540 u, found: 283.1543 u.

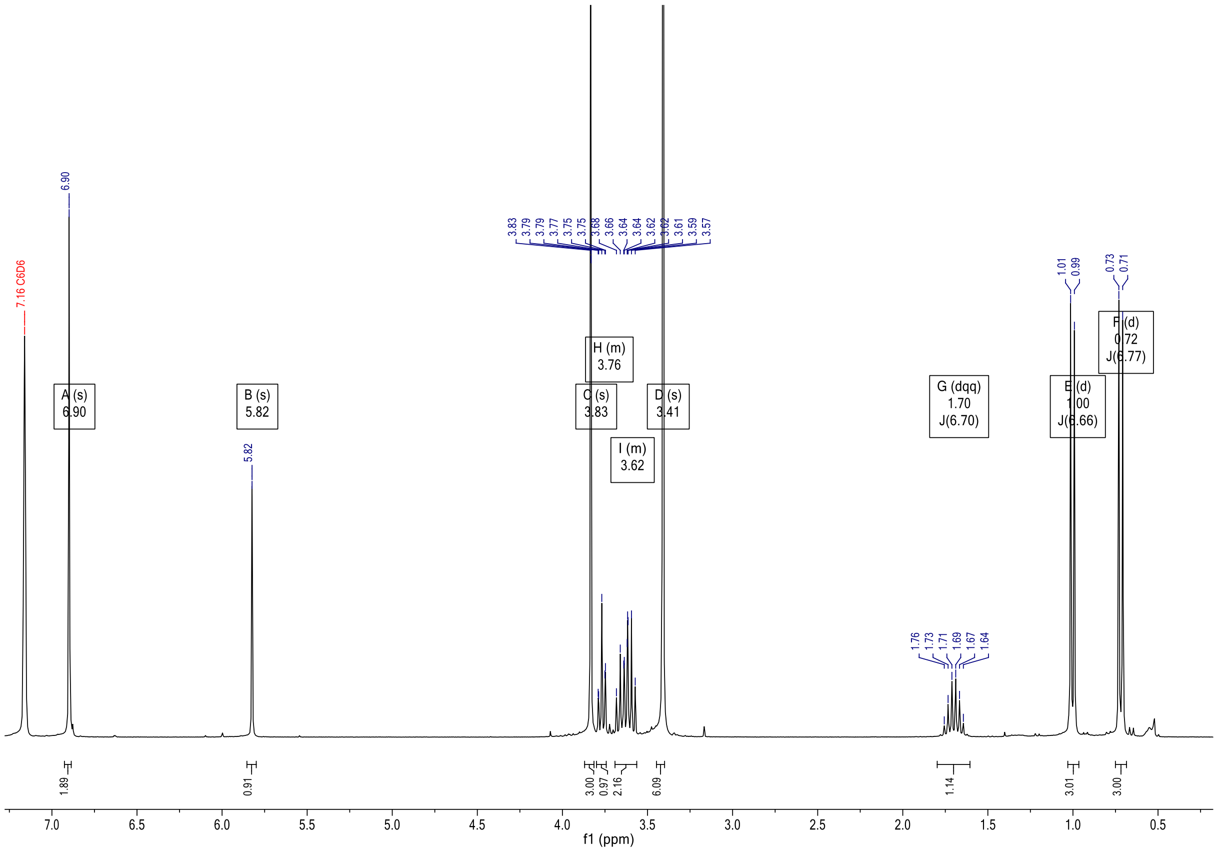
7.16 C6D6
6.90
5.82
A (s) 6.90
B (s) 5.82
C (s) 3.83
D (s) 3.41
H (m) 3.76
I (m) 3.62
G (dqq) 1.70 J(6.70)
E (d) 1.00 J(6.66)
F (d) 0.72 J(6.77)
1.89
0.91
3.00
0.97
2.16
6.09
1.14
3.01
3.00
f1 (ppm)

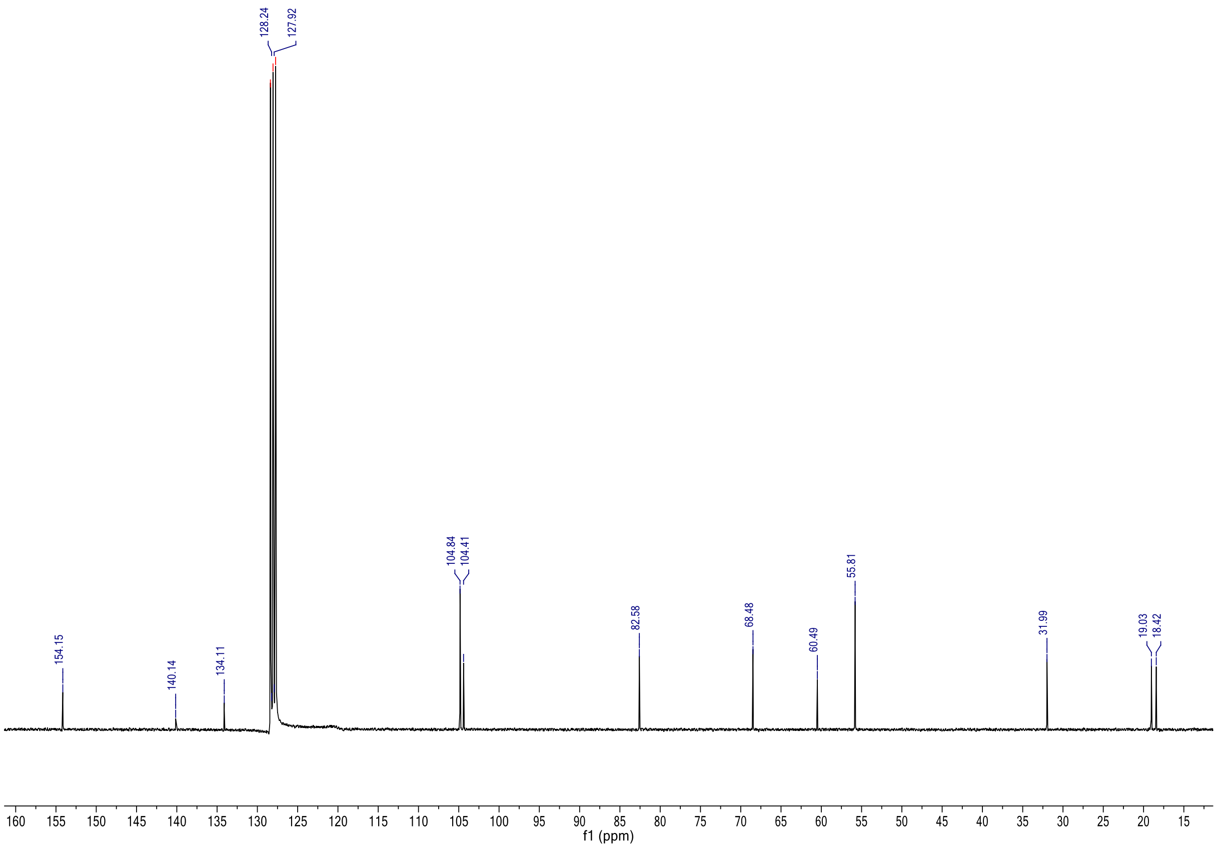
154.15
140.14
134.11
128.24
127.92
104.84
104.41
82.58
68.48
60.49
55.81
31.99
19.03
18.42
f1 (ppm)

### 4.1.7 Synthesis of 4-cyclohexyl-2-(4-methylphenyl)-1,3-dioxolane **2g**

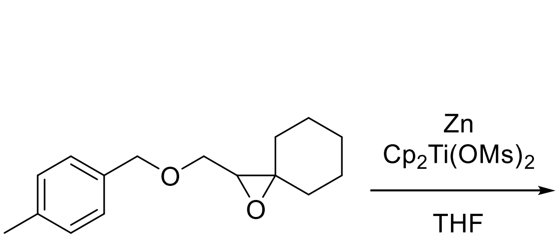


According to GP VII 15.5 mg $Cp_2Ti(OMs)_2$ (0.0421 mmol, 0.10 eq.), 5.7 mg zinc dust (0.0872 mmol, 0.22 eq.) and 99.6 mg substrate **1g** (0.404 mmol, 1.00 eq.) are refluxed. Column chromatography ($SiO_2$, eluent: CH:EA, 97:3) afforded 88.9 mg **2g** (d.r. = 98:2, 0.361 mmol, 89%) as a colourless oil.

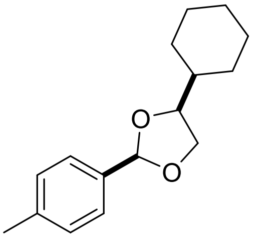

**2g**
$C_{16}H_{22}O_2$
246,35 g/mol

**$R_f$ =** 0.58 (10% EA, 90% CH). **$^1$H-NMR (500 MHz, $C_6D_6$, RT): δ [ppm] =** 7.56 (d, *J* = 7.9 Hz, 2H), 7.03 (d, *J* = 7.9 Hz, 2H), 5.84 (s, 1H), 3.76 (*p*t, *J* = 7.1 Hz, 1H), 3.66 (*p*q, *J* = 7.1 Hz, 1H), 3.59 (*p*t, *J* = 7.2 Hz, 1H), 2.08 (s, 3H), 2.08 – 2.01 (m, 1H), 1.67 – 1.60 (m, 1H), 1.60 – 1.51 (m, 2H), 1.45 – 1.36 (m, 1H), 1.36 – 1.30 (m, 1H), 1.16 – 0.96 (m, 4H), 0.91 – 0.82 (m, 1H). **$^{13}$C-NMR (125 MHz, $C_6D_6$, RT): δ [ppm] =** 138.8, 136.4, 129.1, 127.2, 104.3, 81.7, 68.5, 41.6, 29.6, 29.1, 26.8, 26.2, 26.1, 21.2. **IR:** 2923, 2852, 1449, 1424, 1378, 1307, 1223, 1209, 1177, 1089, 1020, 1006, 980, 939, 889, 811, 775, 728, 530 $cm^{-1}$. **HRMS (ESI+):** *m/z* calculated for $C_{16}H_{23}O_2^+$: 247.1693 u, found: 247.1692 u.

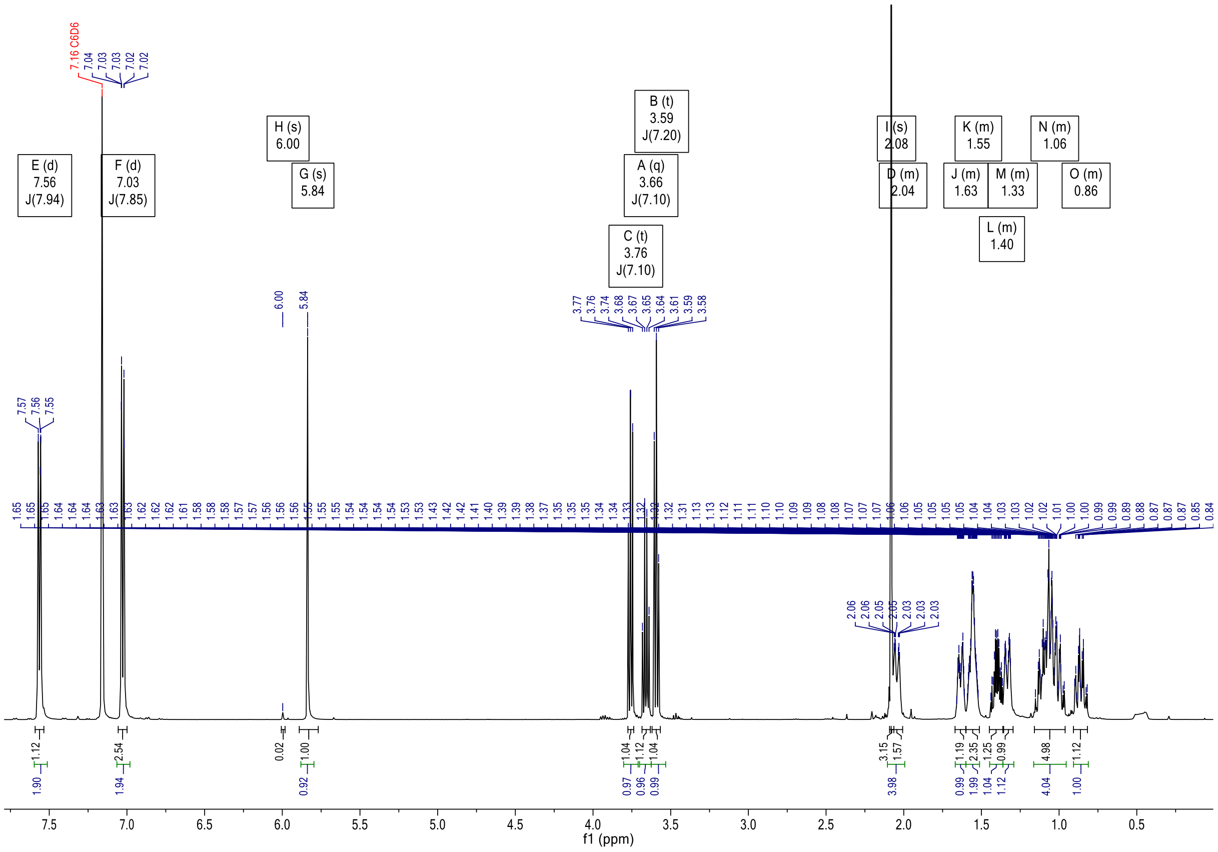

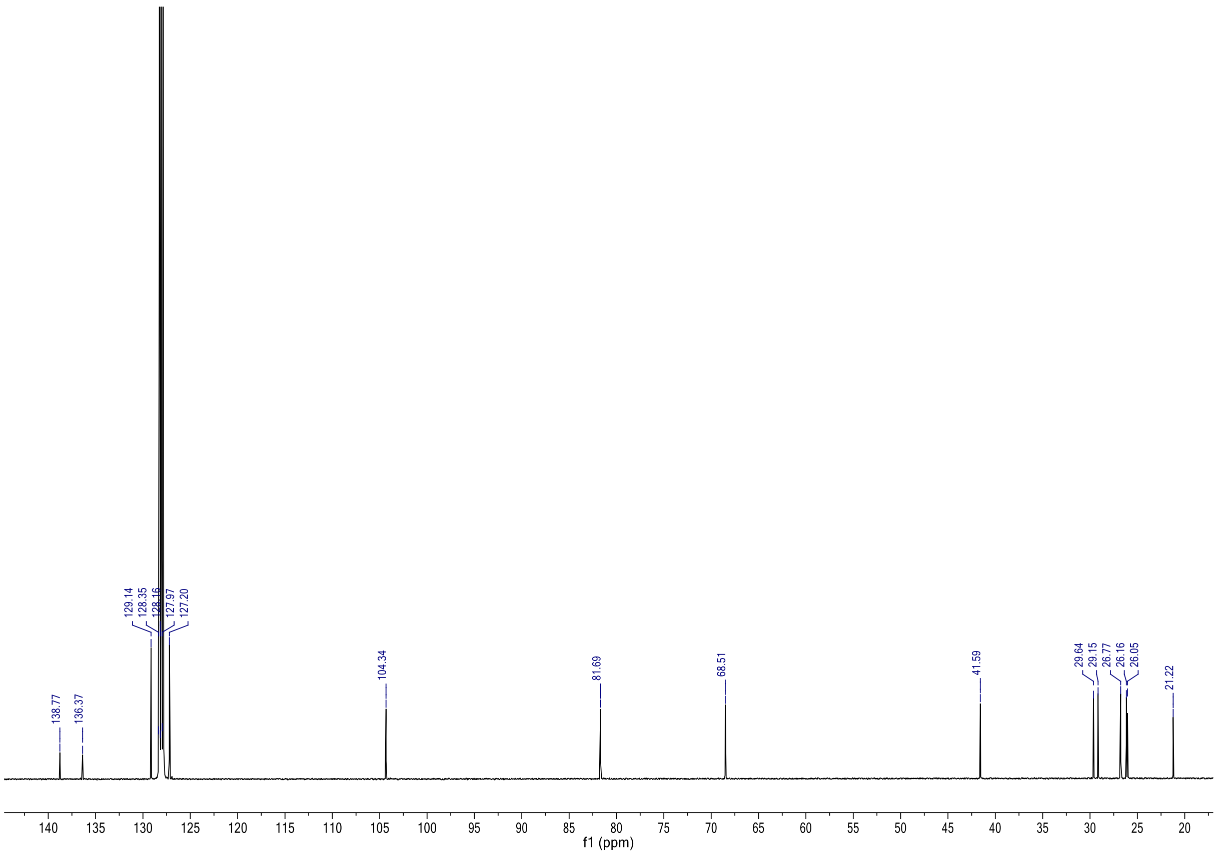

### 4.1.8 Synthesis of 4-cyclohexyl-2-phenyl-1,3-dioxolane **2h**

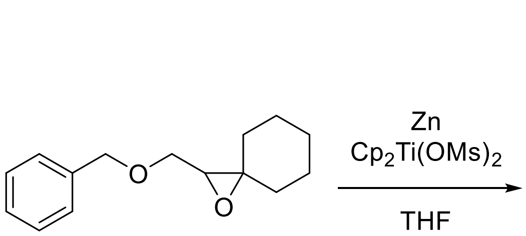


According to GP VII 16.0 mg $Cp_2Ti(OMs)_2$ (0.0432 mmol, 0.10 eq.), 5.5 mg zinc dust (0.0841 mmol, 0.19 eq.) and 100.4 mg substrate **1h** (0.432 mmol, 1.00 eq.) are refluxed. Column chromatography ($SiO_2$, eluent: CH:EA, 97:3) afforded 80.8 mg **2h** (d.r. = 99:1, 0.348 mmol, 81%) as a colourless oil.

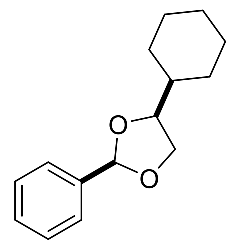


**2h**
$C_{15}H_{20}O_2$
232,32 g/mol

**$R_f$ =** 0.35 (10% EA, 90% CH). **$^1$H-NMR (500 MHz, $C_6D_6$, RT): δ [ppm] =** 7.64 – 7.59 (m, 2H), 7.21 – 7.16 (m, 2H), 7.15 – 7.09 (m, 1H), 5.81 (s, 1H), 3.74 (*p*t, *J* = 7.1 Hz, 1H), 3.65 (*p*q, *J* = 7.1 Hz, 1H), 3.56 (*p*t, *J* = 7.2 Hz, 1H), 2.08 – 1.98 (m, 1H), 1.67 – 1.60 (m, 1H), 1.58 – 1.52 (m, 2H), 1.44 – 1.33 (m, 1H), 1.34 – 1.28 (m, 1H), 1.16 – 0.95 (m, 4H), 0.89 – 0.80 (m, 1H). **$^{13}$C-NMR (125 MHz, $C_6D_6$, RT): δ [ppm] =** 139.2, 129.2, 128.4, 127.2, 104.2, 81.7, 68.5, 41.5, 29.6, 29.1, 26.7, 26.1, 26.0. **IR:** 2923, 2852, 1450, 1379, 1219, 1090, 1066, 1027, 1007, 979, 913, 889, 757, 697, 640 cm$^{-1}$. **HRMS (APCI):** *m/z* calculated for $C_{15}H_{21}O_2^+$: 233.1536 u, found: 233.1531 u.

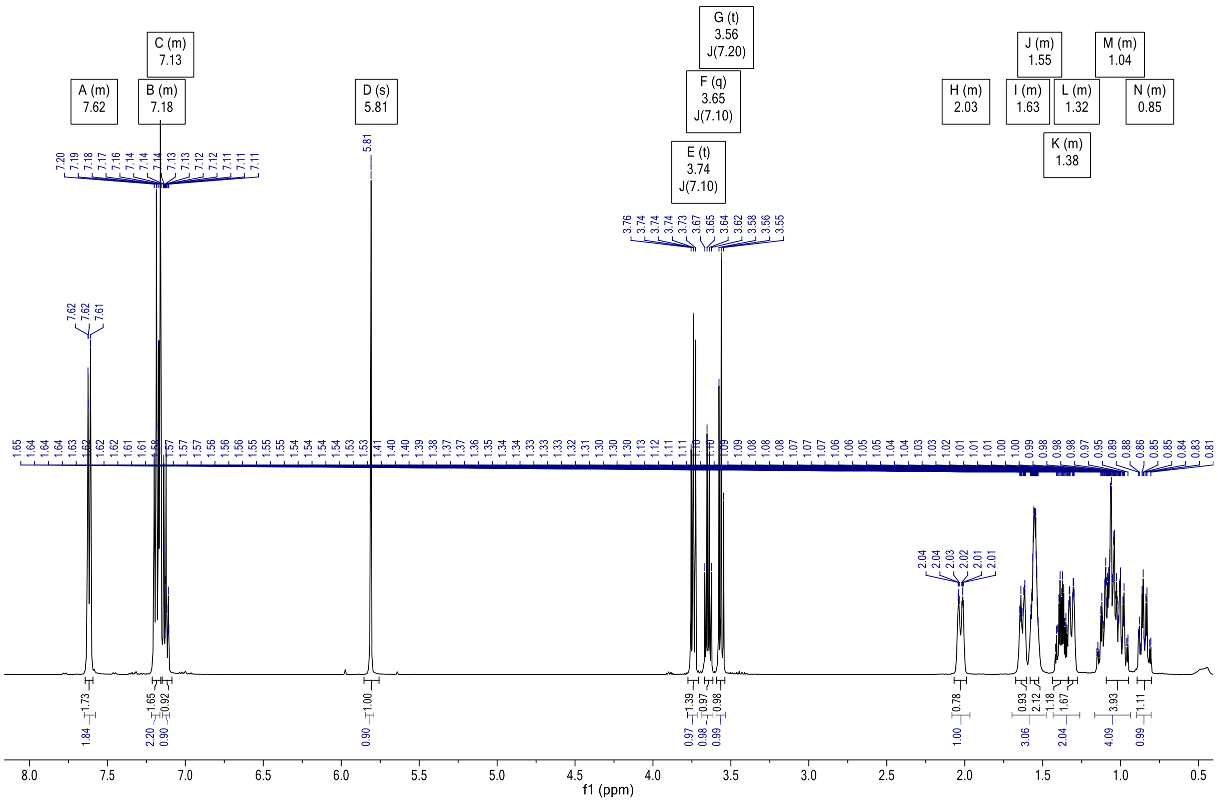

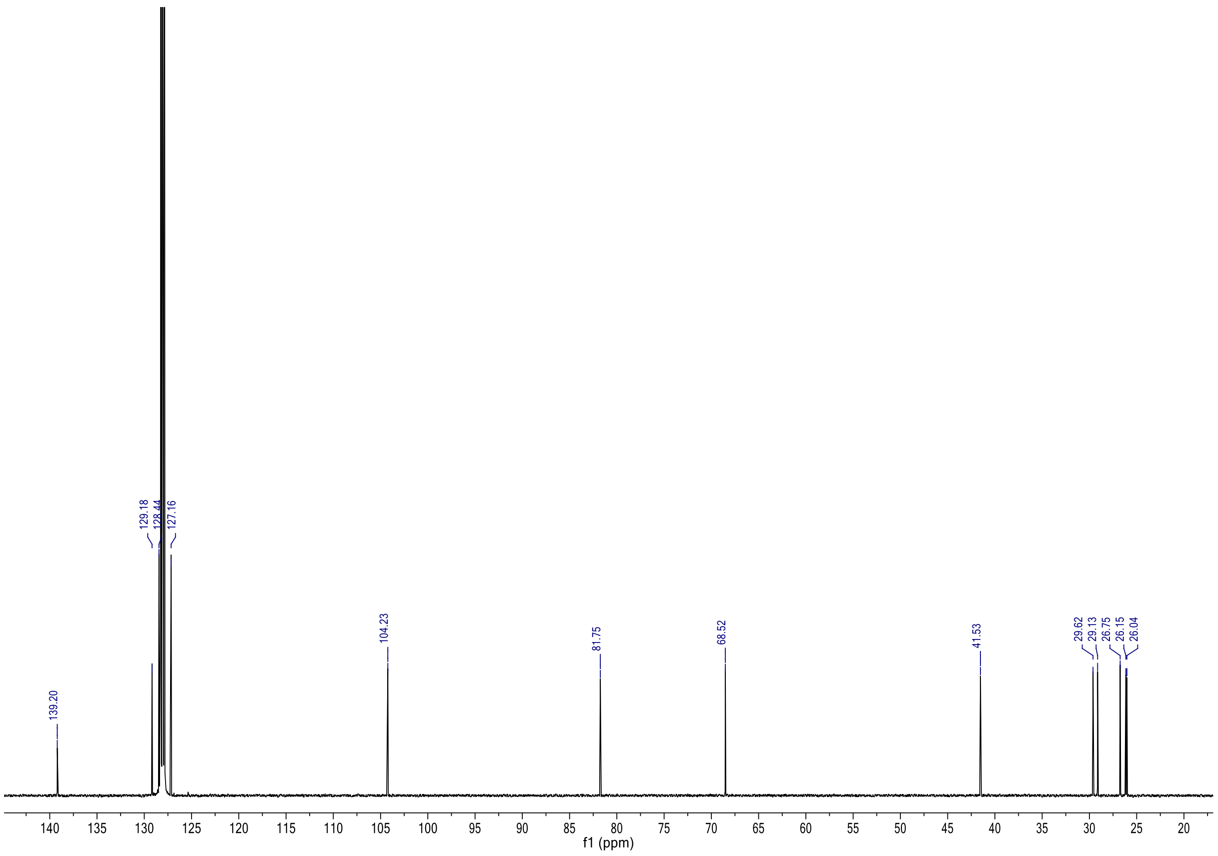

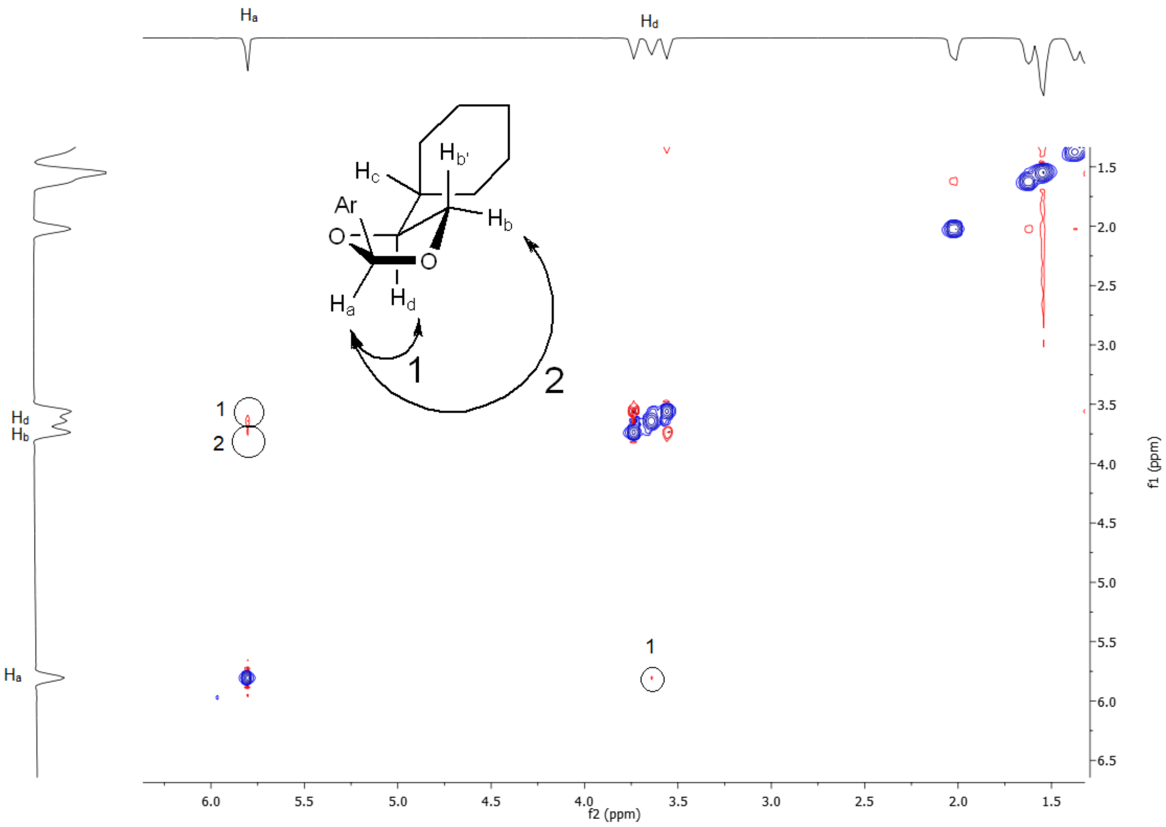


Coupling **1** indicates a *cis*–configuration of the aryl group and the cyclohexyl group. Furthermore, $H_b$ is *cis*-configurated to $H_d$ (see coupling constant in $^1$H-spectra). For this reason coupling **2** between $H_a$ and $H_b$ proves the relative configuration as depicted.

### 4.1.9 Synthesis of 2-(4-chlorophenyl)-4-(propan-2-yl)-1,3-dioxolane **2i**

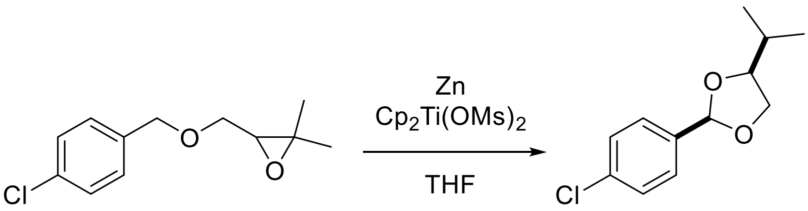


According to GP VII 32.4 mg $Cp_2Ti(OMs)_2$ (0.0880 mmol, 0.10 eq.), 11.6 mg zinc dust (0.177 mmol, 0.20 eq.) and 199.0 mg substrate **1i** (0.878 mmol, 1.00 eq.) are refluxed. Column chromatography ($SiO_2$, eluent: CH:EA, 97:3) afforded 111.7 mg **2i** (d.r. = 99:1, 0.493 mmol, 56%) as a colourless oil.

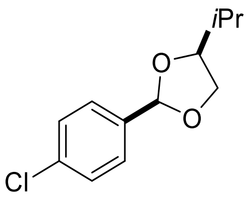


**2i**
$C_{12}H_{15}ClO_2$
226,70 g/mol

**R$_f$ =** 0.63 (30% EA, 70% CH). **$^1$H-NMR (500 MHz, $C_6D_6$, RT): δ [ppm] =** 7.30 (d, *J* = 8.4 Hz, 2H), 7.12 (d, *J* = 8.4 Hz, 2H), 5.62 (s, 1H), 3.65 (dd, *J* = 7.4, 6.7 Hz, 1H), 3.53 (*p*q, *J* = 7.0 Hz, 1H), 3.44 (*p*t, *J* = 7.1 Hz, 1H), 1.56 (dqq, *J* = 7.0, 6.8, 6.8 Hz, 1H), 0.92 (d, *J* = 6.8 Hz, 3H), 0.64 (d, *J* = 6.8 Hz, 3H). **$^{13}$C-NMR (125 MHz, $C_6D_6$, RT): δ [ppm] =** 137.5, 135.1, 128.7, 128.5, 103.5, 82.7, 68.5, 31.8, 18.9, 18.3. **IR:** 2973, 2872, 1492, 1466, 1423, 1385, 1367, 1217, 1107, 1082, 1011, 975, 949, 862, 821, 730, 720, 534, 511 cm$^{-1}$. **HRMS (ESI+):** *m/z* calculated for $C_{12}H_{14}O_2Cl^+$: 225.0682 u, found: 225.0684 u.

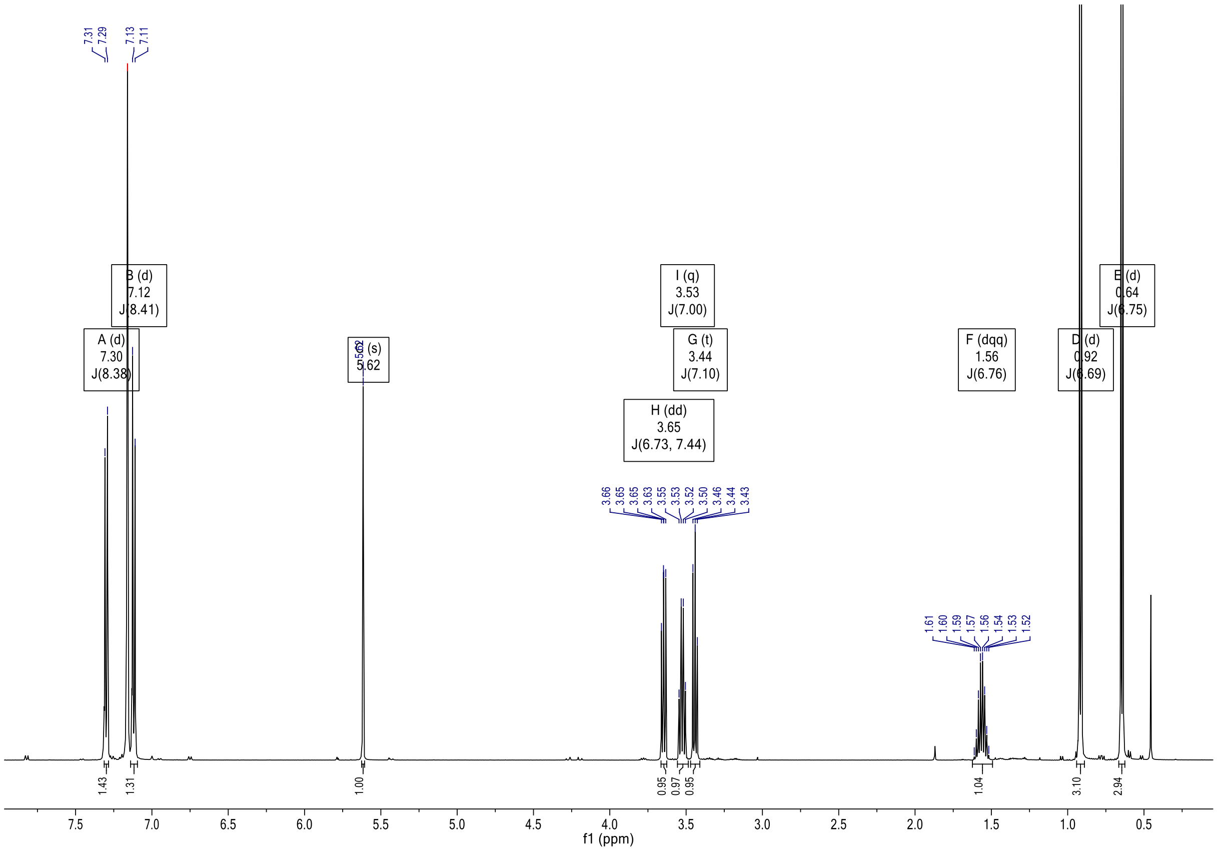

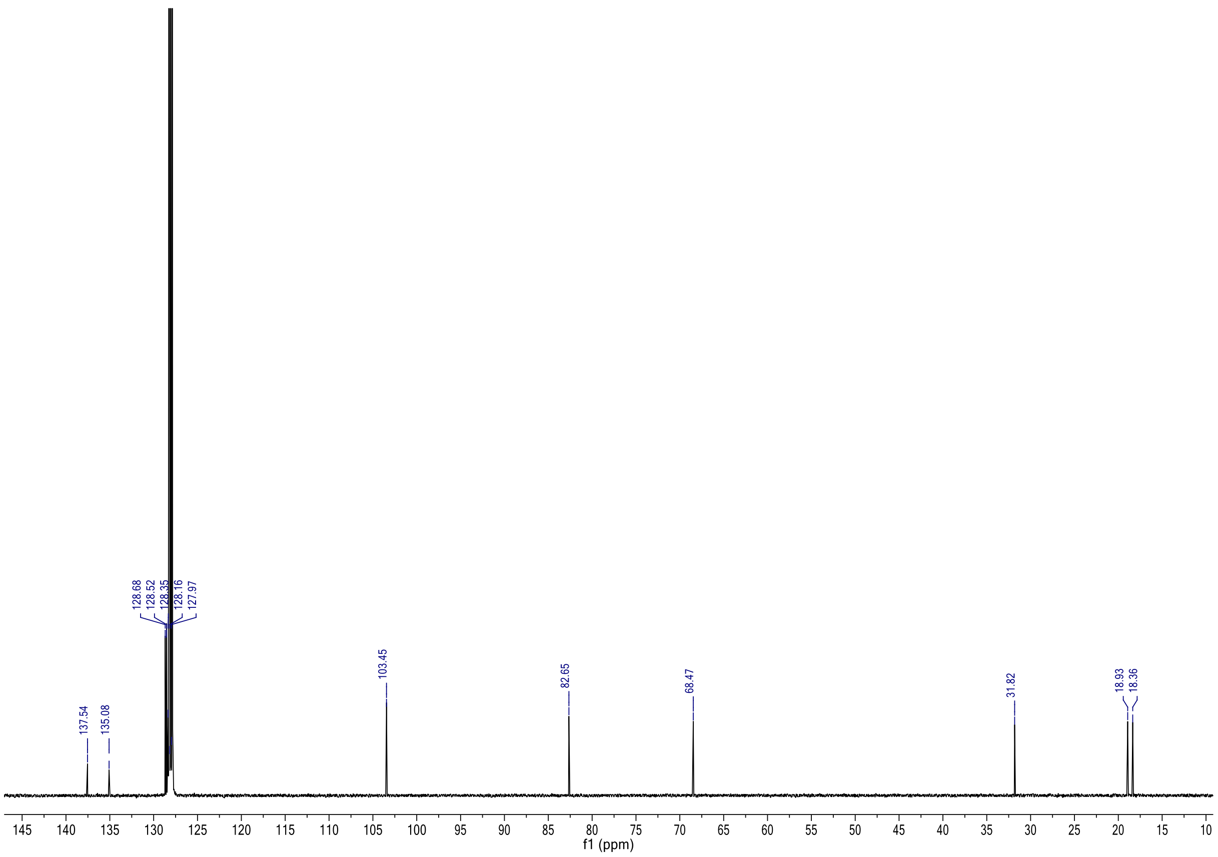


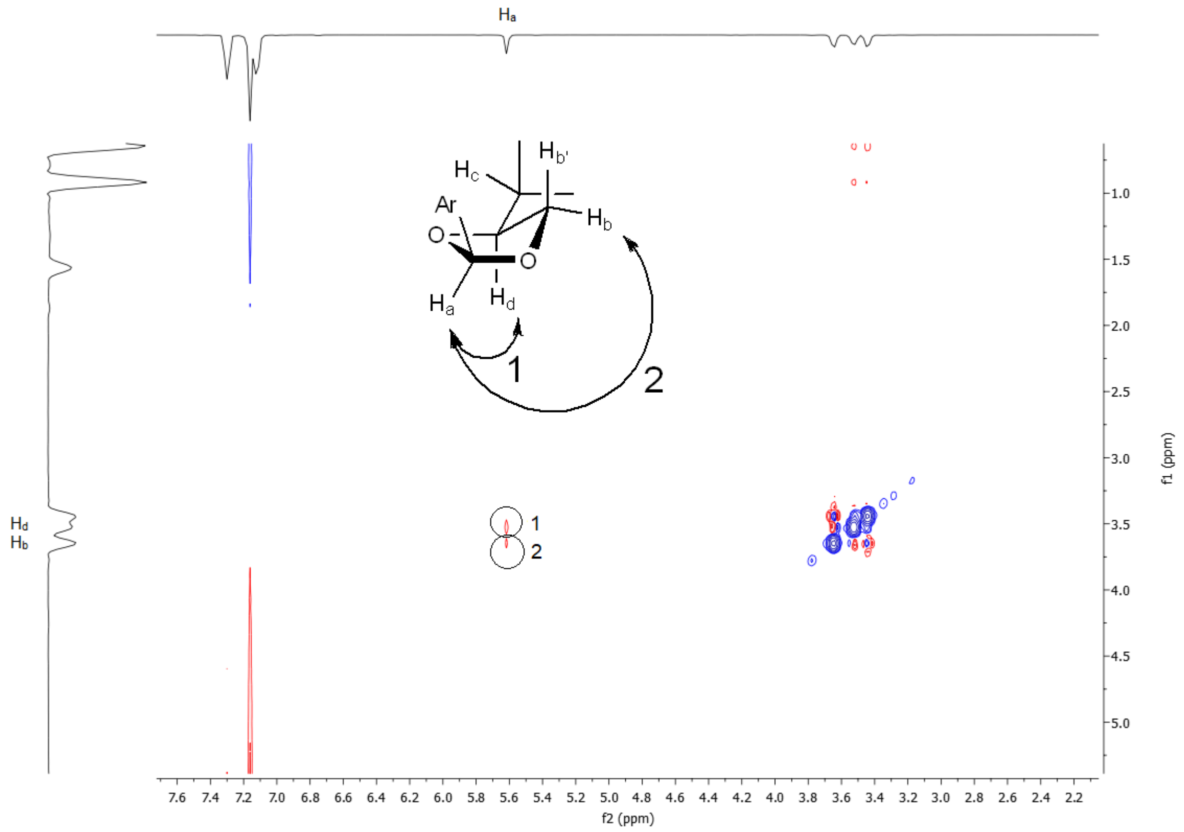


Coupling **1** indicates a *cis*–configuration of the aryl group and the isopropyl group. Furthermore, $H_b$ is *cis*-configurated to $H_d$ (see coupling constant in $^1$H-spectra). For this reason coupling **2** between $H_a$ and $H_b$ proves the relative configuration as depicted.

## 4.2 Titanocene catalyzed synthesis of ketone-dervied acetals **4a-4e**

### 4.2.1 Synthesis of 4-(propan-2-yl)-2',3'-dihydrospiro[[1,3]dioxolane-2,1'-indene] **4a**

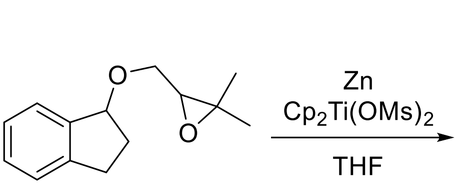


According to GP VII 17.0 mg $Cp_2Ti(OMs)_2$ (0.0462 mmol, 0.10 eq.), 6.4 mg zinc dust (0.0979 mmol, 0.22 eq.) and 98.6 mg substrate **3a** (0.452 mmol, 1.00 eq.) are refluxed. Column chromatography ($SiO_2$, eluent: CH:EA, 95:5) afforded 67.6 mg **4a** (d.r. = 58:42, 0.310 mmol, 69%) as a colourless oil.

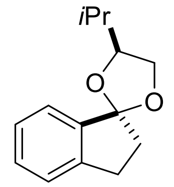


**4a**
$C_{14}H_{18}O_2$
218,30 g/mol

**$R_f$ =** 0.36 (5% EA, 95% CH). ***cis*-Diastereomer (major): $^1$H-NMR (300 MHz, $C_6D_6$, RT): δ [ppm] =** 7.59 – 7.54 (m, 1H), 7.20 – 7.10 (m, 2H), 7.07 – 6.98 (m, 1H), 3.88 – 3.78 (m, 1H), 3.71 – 3.59 (m, 2H), 2.83 – 2.63 (m, 2H), 2.36 – 2.21 (m, 2H), 1.79 – 1.65 (m, 1H), 0.99 (d, *J* = 6.6 Hz, 3H), 0.71 (d, *J* = 6.8 Hz, 3H). **$^{13}$C-NMR (75 MHz, $C_6D_6$, RT): δ [ppm] =** 144.1, 143.6, 129.6, 127.0, 125.2, 124.1, 117.9, 81.9, 68.6, 37.8, 32.0, 28.8, 19.3, 18.4. ***trans*-Diastereomer (minor): $^1$H-NMR (300 MHz, $C_6D_6$, RT): δ [ppm] =** 7.49 – 7.44 (m, 1H), 7.20 – 7.10 (m, 2H), 7.07 – 6.98 (m, 1H), 3.99 (dd, *J* = 7.7, 6.0 Hz, 1H), 3.96 – 3.82 (m, 1H), 3.51 (*p*t, *J* = 7.6 Hz, 1H), 2.83 – 2.63 (m, 2H), 2.36 – 2.21 (m, 2H), 1.72 – 1.54 (m, 1H), 0.98 (d, *J* = 6.7 Hz, 3H), 0.69 (d, *J* = 6.8 Hz, 3H). **$^{13}$C-NMR (75 MHz, $C_6D_6$, RT): δ [ppm] =** 144.0, 143.1, 129.3, 126.9, 125.4, 123.7, 118.1, 82.3, 68.6, 38.4, 32.3, 28.7, 19.3, 18.3. **IR:** 2958, 2873, 1462, 1307, 1273, 1124, 1082, 1044, 1019, 925, 757, 723, 577 $cm^{-1}$. **HRMS (ESI+):** *m/z* calculated for $C_{14}H_{19}O_2^+$: 219.1380 u, found: 219.1378 u.

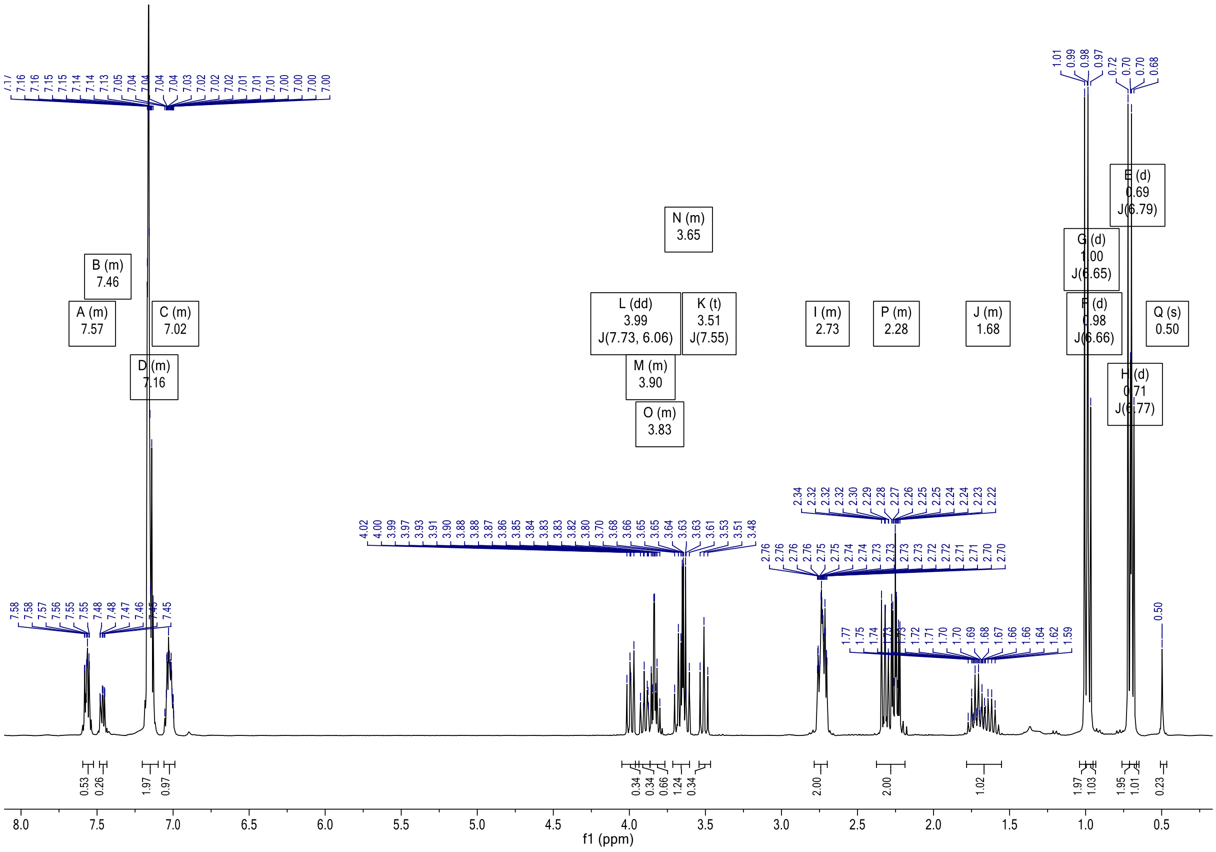

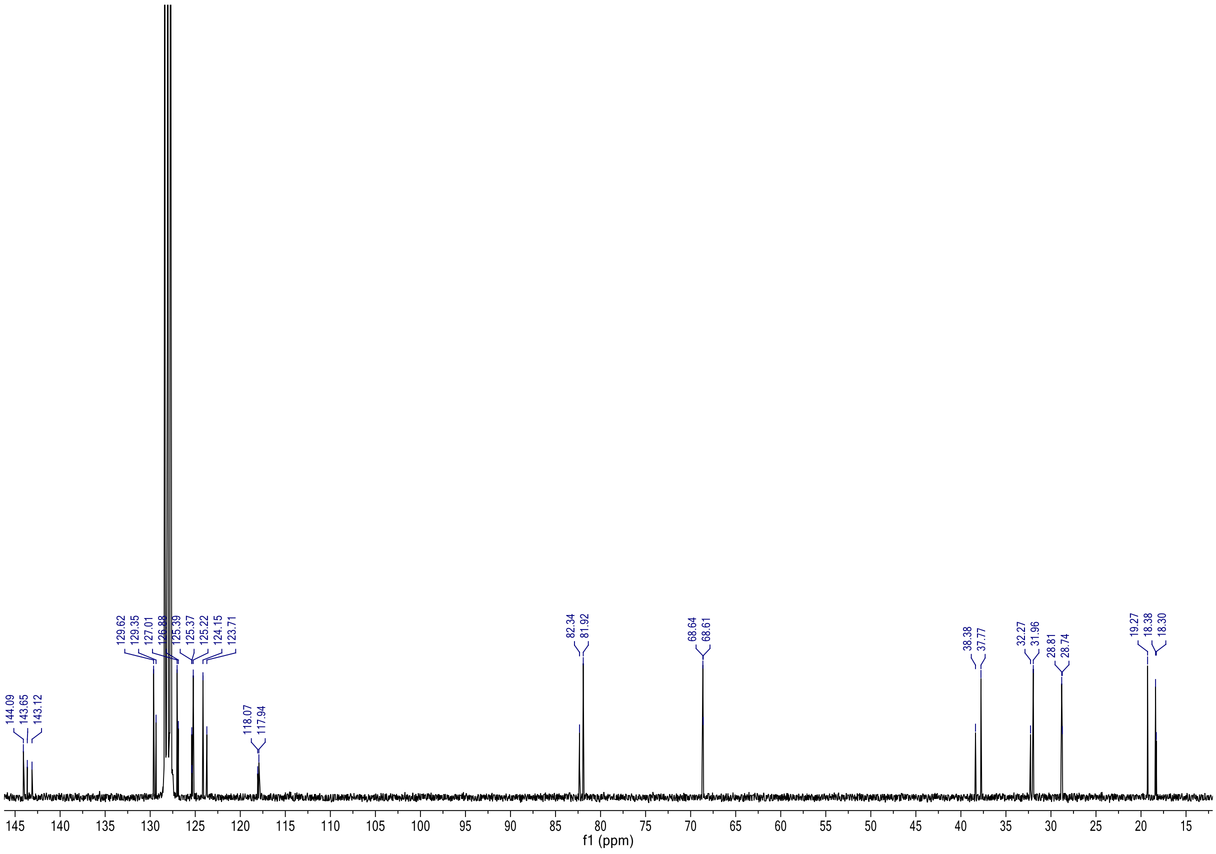

### 4.2.2 Synthesis of 4-(propan-2-yl)-3',4'-dihydro-2'*H*-spiro[[1,3]dioxolane-2,1'-naphthalene] **4b**

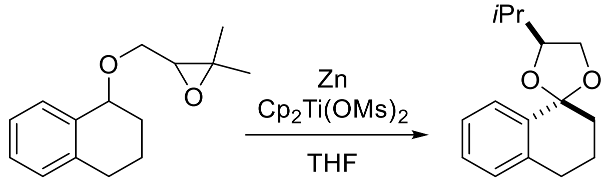


According to GP VII 16.0 mg $Cp_2Ti(OMs)_2$ (0.0434 mmol, 0.10 eq.), 5.8 mg zinc dust (0.0887 mmol, 0.20 eq.) and 100.8 mg substrate **3b** (0.434 mmol, 1.00 eq.) are refluxed. Column chromatography ($SiO_2$, eluent: CH:EA, 95:5) afforded 87.1 mg **4b** (d.r. = 50:50, 0.375 mmol, 86%) as a colourless oil.

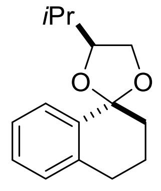


**4b**
$C_{15}H_{20}O_2$
232,32 g/mol

**$R_f$ =** 0.65 (20% EA, 80% CH). ***trans*-Diastereomer: $^1$H-NMR (700 MHz, $C_6D_6$, RT): δ [ppm] =** 7.70 (dd, *J* = 7.8, 1.4 Hz, 1H), 7.21 – 7.17 (m, 1H), 7.14 – 7.08 (m, 1H), 6.95 – 6.90 (m, 1H), 3.97 (dd, *J* = 8.1, 5.9 Hz, 1H), 3.83 (*p*td, *J* = 8.1, 5.9 Hz, 1H), 3.49 (*p*t, *J* = 8.2 Hz, 1H), 2.57 – 2.51 (m, 2H), 1.99 – 1.93 (m, 2H), 1.91 – 1.80 (m, 2H), 1.71 (dqq, *J* = 7.8, 6.8, 6.5 Hz, 1H), 0.99 (d, *J* = 6.5 Hz, 3H), 0.70 (d, *J* = 6.8 Hz, 3H). **$^{13}$C-NMR (176 MHz, $C_6D_6$, RT): δ [ppm] =** 138.7, 138.4, 128.7, 128.3, 126.9, 126.3, 108.0, 82.9, 68.7, 34.2, 32.0, 29.4, 21.1, 19.6, 18.3. ***cis*-Diastereomer: $^1$H-NMR (500 MHz, $C_6D_6$, RT): δ [ppm] =** 7.82 (dd, *J* = 7.9, 1.4 Hz, 1H), 7.21 – 7.17 (m, 1H), 7.14 – 7.08 (m, 1H), 6.95 – 6.90 (m, 1H), 3.87 (dd, *J* = 7.5, 6.3 Hz, 1H), 3.71 (*p*td, *J* = 7.8, 6.3 Hz, 1H), 3.63 (*p*t, *J* = 7.7 Hz, 1H), 2.57 – 2.51 (m, 2H), 1.99 – 1.93 (m, 2H), 1.91 – 1.80 (m, 2H), 1.57 (dqq, *J* = 7.6, 6.8, 6.5 Hz, 1H), 0.97 (d, *J* = 6.5 Hz, 3H), 0.65 (d, *J* = 6.8 Hz, 3H). **$^{13}$C-NMR (126 MHz, $C_6D_6$, RT): δ [ppm] =** 139.5, 138.6, 128.7, 128.5, 127.4, 126.1, 107.8, 81.4, 68.8, 35.8, 32.7, 29.2, 21.1, 19.4, 18.5. **IR:** 2938, 2870, 1454, 1440, 1344, 1309, 1296, 1232, 1168, 1134, 1116, 1062, 1034, 956, 933, 909, 754, 723, 553 cm$^{-1}$. **HRMS (ESI+):** *m/z* calculated for $C_{15}H_{21}O_2^+$: 233.1536 u, found: 233.1536 u.

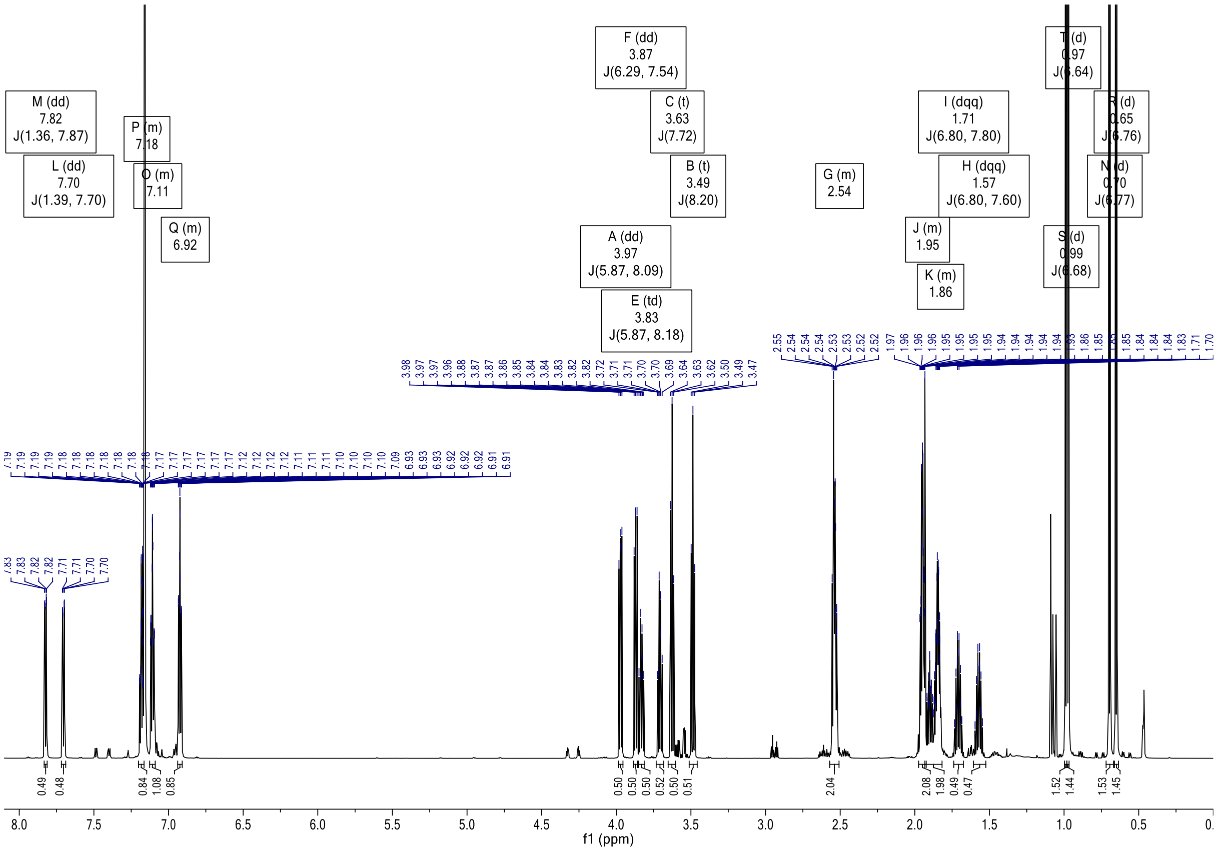

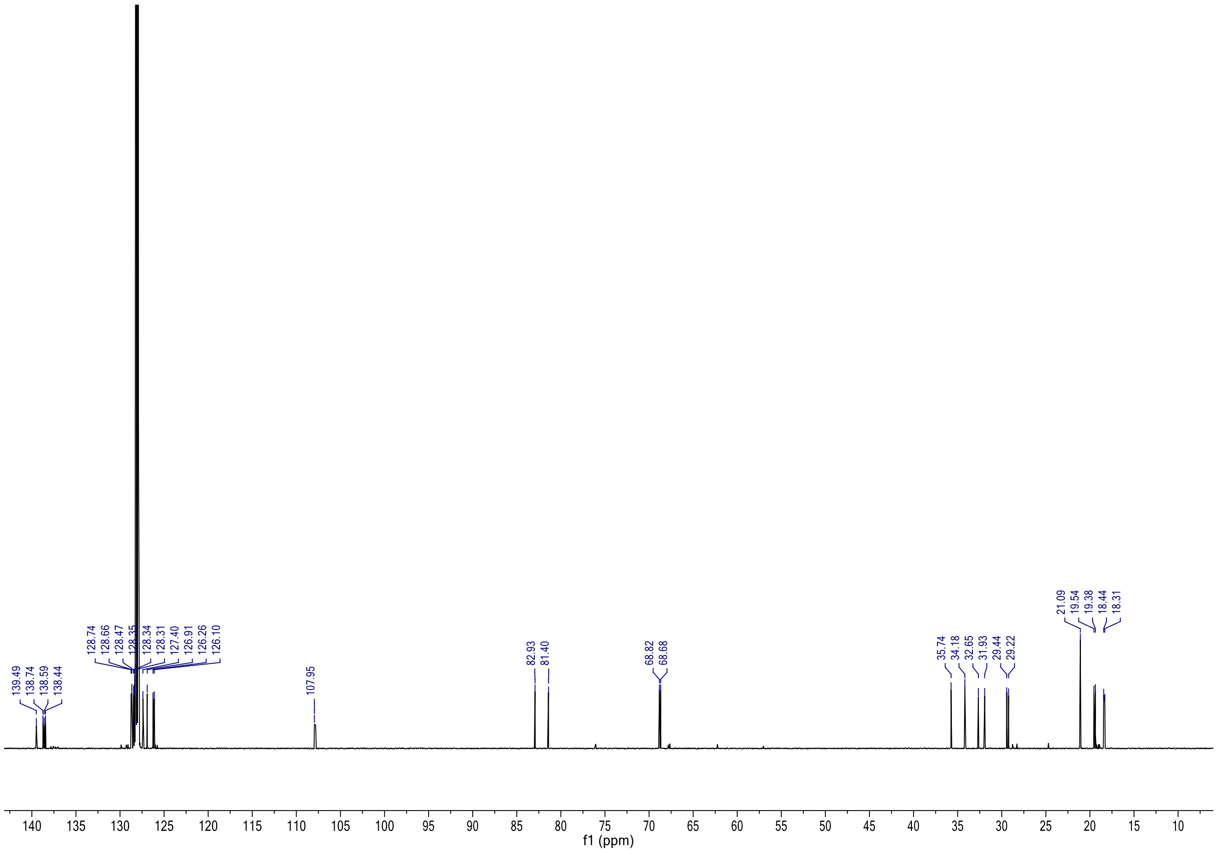

4.2.3 Synthesis of 2-methyl-2-phenyl-4-(propan-2-yl)-1,3-dioxolane **4c**

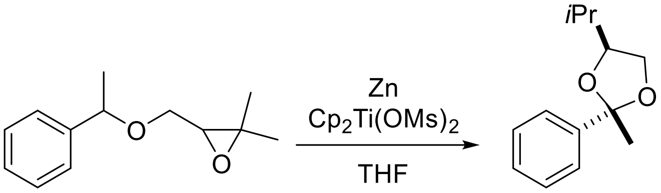

According to GP VII 18.2 mg $Cp_2Ti(OMs)_2$ (0.494 mmol, 0.10 eq.), 6.4 mg zinc dust (0.0979 mmol, 0.20 eq.) and 100.0 mg substrate **3c** (0.485 mmol, 1.00 eq.) are refluxed. Column chromatography ($SiO_2$, eluent: CH:EA, 95:5) afforded 44.4 mg **4c** (d.r. = 59:41, 0.215 mmol, 44%) as a colourless oil.

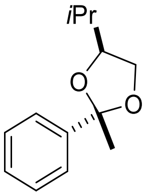

**4c**
$C_{13}H_{18}O_2$
206,29 g/mol

**$R_f$ =** 0.68 (20% EA, 80% CH). ***trans*–Diastereomer (major): $^1$H-NMR (700 MHz, $C_6D_6$, RT): δ [ppm] =** 7.68 (d, *J* = 8.2, 1.4 Hz, 2H), 7.23 – 7.18 (m, 2H), 7.13 – 7.09 (m, 1H), 3.84 (dd, *J* = 8.1, 6.0 Hz, 1H), 3.65 (*p*td, *J* = 8.6, 6.0 Hz, 1H), 3.26 (*p*t, *J* = 8.3 Hz, 1H), 1.69 (s, 3H), 1.37 (dqq, *J* = 8.5, 6.8, 6.6 Hz, 1H), 1.00 (d, *J* = 6.6 Hz, 3H), 0.43 (d, *J* = 6.8 Hz, 3H). **$^{13}$C-NMR (150 MHz, $C_6D_6$, RT): δ [ppm] =** 145.8, 128.3, 127.8, 125.7, 109.4, 83.1, 69.0, 32.3, 28.9, 19.9, 18.2. ***cis*-Diastereomer (minor): $^1$H-NMR (700 MHz, $C_6D_6$, RT): δ [ppm] =** 7.64 (d, *J* = 8.2, 1.4 Hz, 2H), 7.23 – 7.18 (m, 2H), 7.13 – 7.09 (m, 1H), 3.58 (dd, *J* = 7.5, 6.8 Hz, 1H), 3.50 (dd, *J* = 7.5, 6.5 Hz, 1H), 3.46 (*p*q, *J* = 6.8 Hz, 1H) 1.72 (s, 3H), 1.63 (dqq, *J* = 8.5, 6.8, 6.7 Hz, 1H), 0.93 (d, *J* = 6.7 Hz, 3H), 0.64 (d, *J* = 6.8 Hz, 3H). **$^{13}$C-NMR (176 MHz, $C_6D_6$, RT): δ [ppm] =** 144.9, 128.5, 127.9, 125.8, 109.3, 81.2, 67.8, 32.0, 28.7, 18.8, 18.1. **IR:** 2959, 2873, 1370, 1245, 1198, 1117, 1068, 1044, 1025, 921, 877, 762, 700, 596 $cm^{-1}$. **HRMS (ESI+):** *m/z* calculated for $C_{14}H_{21}O_2^+$: 207.1380 u, found: 207.1379 u.

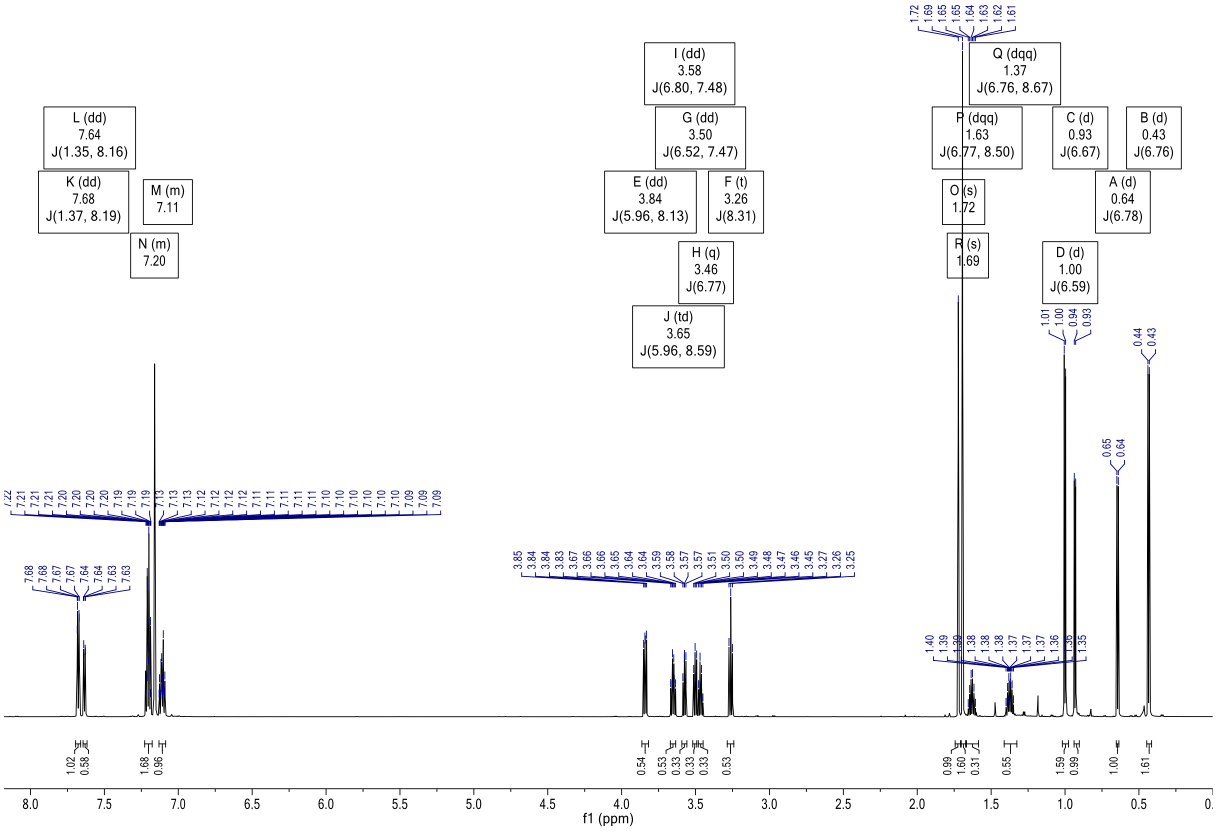
L (dd) 7.64 J(1.35, 8.16)
K (dd) 7.68 J(1.37, 8.19)
M (m) 7.11
N (m) 7.20
I (dd) 3.58 J(6.80, 7.48)
G (dd) 3.50 J(6.52, 7.47)
E (dd) 3.84 J(5.96, 8.13)
F (t) 3.26 J(8.31)
H (q) 3.46 J(6.77)
J (td) 3.65 J(5.96, 8.59)
Q (dqq) 1.37 J(6.76, 8.67)
P (dqq) 1.63 J(6.77, 8.50)
C (d) 0.93 J(6.67)
B (d) 0.43 J(6.76)
O (s) 1.72
A (d) 0.64 J(6.78)
R (s) 1.69
D (d) 1.00 J(6.59)
f1 (ppm)

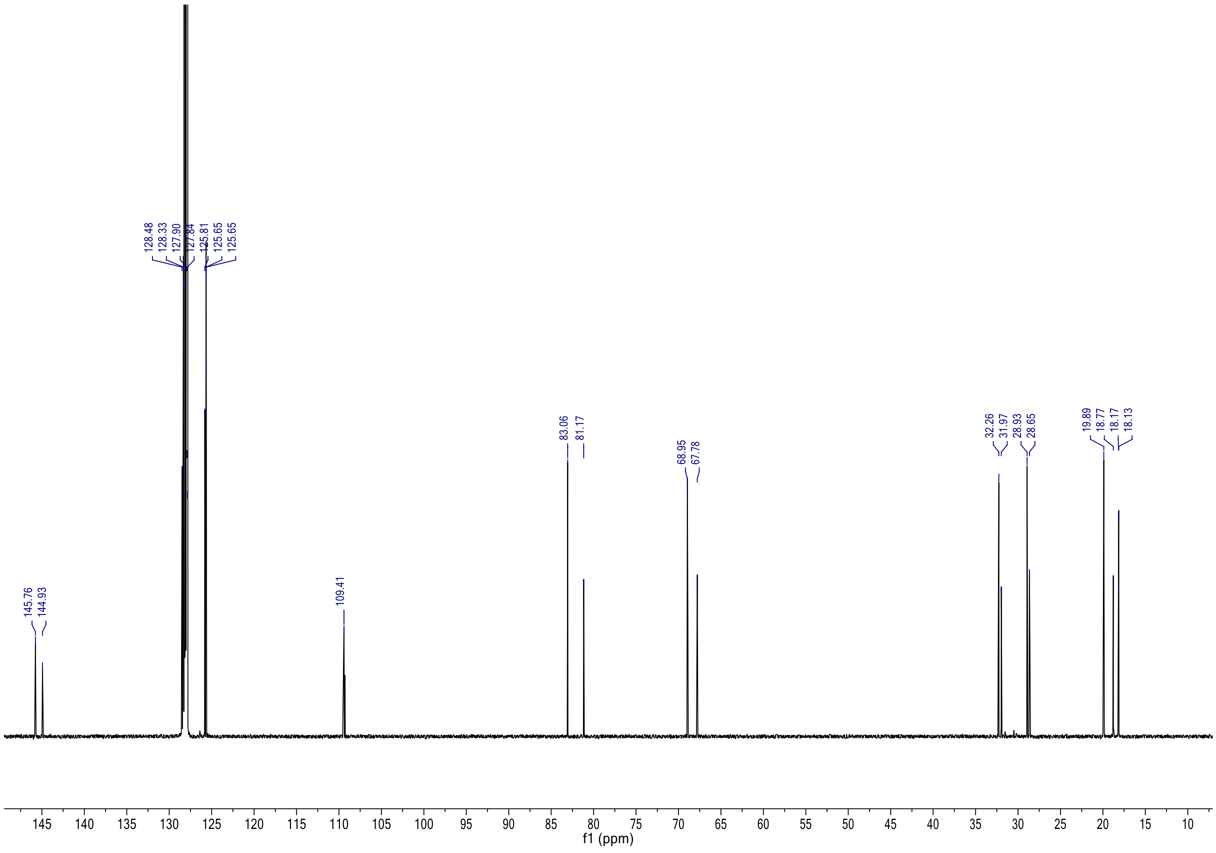
145.76
144.93
128.48
128.33
127.90
127.84
125.81
125.65
125.65
109.41
83.06
81.17
68.95
67.78
32.26
31.97
28.93
28.65
19.89
18.77
18.17
18.13
f1 (ppm)

4.2.4 Synthesis of 2-methyl-2-(4-methylphenyl)-4-(propan-2-yl)-1,3-dioxolane **4d**

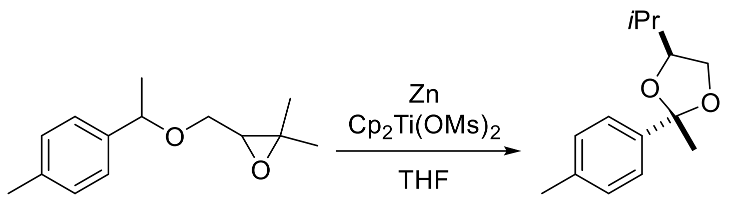


According to GP VII 17.0 mg $Cp_2Ti(OMs)_2$ (0.0462 mmol, 0.10 eq.), 5.9 mg zinc dust (0.0902 mmol, 0.20 eq.) and 100.0 mg substrate **3d** (0.454 mmol, 1.00 eq.) are refluxed. Column chromatography ($SiO_2$, eluent: CH:EA, 95:5) afforded 67.1 mg **4d** (d.r. = 72:28, 0.304 mmol, 67%) as a colourless oil.

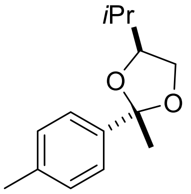


**4d**
$C_{14}H_{20}O_2$
220,31 g/mol

**$R_f$ =** 0.38 (20% EA, 80% CH). ***trans*–Diastereomer (major): $^{1}$H-NMR (300 MHz, $C_6D_6$, RT): δ [ppm] =** 7.61 (d, *J* = 8.1 Hz, 2H), 7.06 – 7.02 (m, 2H), 3.87 (dd, *J* = 8.1, 6.0 Hz, 1H), 3.67 (*p*td, *J* = 8.5, 6.0 Hz, 1H), 3.32 (*p*t, *J* = 8.3 Hz, 1H), 2.12 (s, 3H), 1.73 (s, 3H), 1.42 (dqq, *J* = 8.6, 6.8, 6.6 Hz, 1H), 1.02 (d, *J* = 6.6 Hz, 3H), 0.46 (d, *J* = 6.8 Hz, 3H). **$^{13}$C-NMR (75 MHz, $C_6D_6$, RT): δ [ppm] =** 142.9, 137.2, 129.0, 125.7, 109.5, 83.0, 69.0, 32.3, 29.0, 21.1, 19.3, 18.2. ***cis*–Diastereomer (minor): $^{1}$H-NMR (300 MHz, $C_6D_6$, RT): δ [ppm]** = 7.57 (d, *J* = 8.1 Hz, 2H), 7.06 – 7.02 (m, 2H), 3.65 – 3.61 (m, 1H), 3.55 – 3.50 (m, 2H), 2.13 (s, 3H), 1.76 (s, 3H), 1.68 – 1.62 (m, 1H), 0.95 (d, *J* = 6.7 Hz, 3H), 0.66 (d, *J* = 6.8 Hz, 3H). **$^{13}$C-NMR (75 MHz, $C_6D_6$, RT): δ [ppm] =** 142.0, 137.3, 129.2, 125.8, 109.4, 81.2, 67.8, 32.0, 28.7, 21.1, 18.8, 18.2. **IR:** 2958, 2871, 1468, 1369, 1255, 1197, 1179, 1101, 1079, 1044, 1020, 877, 818, 571 $cm^{-1}$. **HRMS (ESI+):** *m/z* calculated for $C_{14}H_{21}O_2^{+}$: 221.1536 u, found: 221.1535 u.

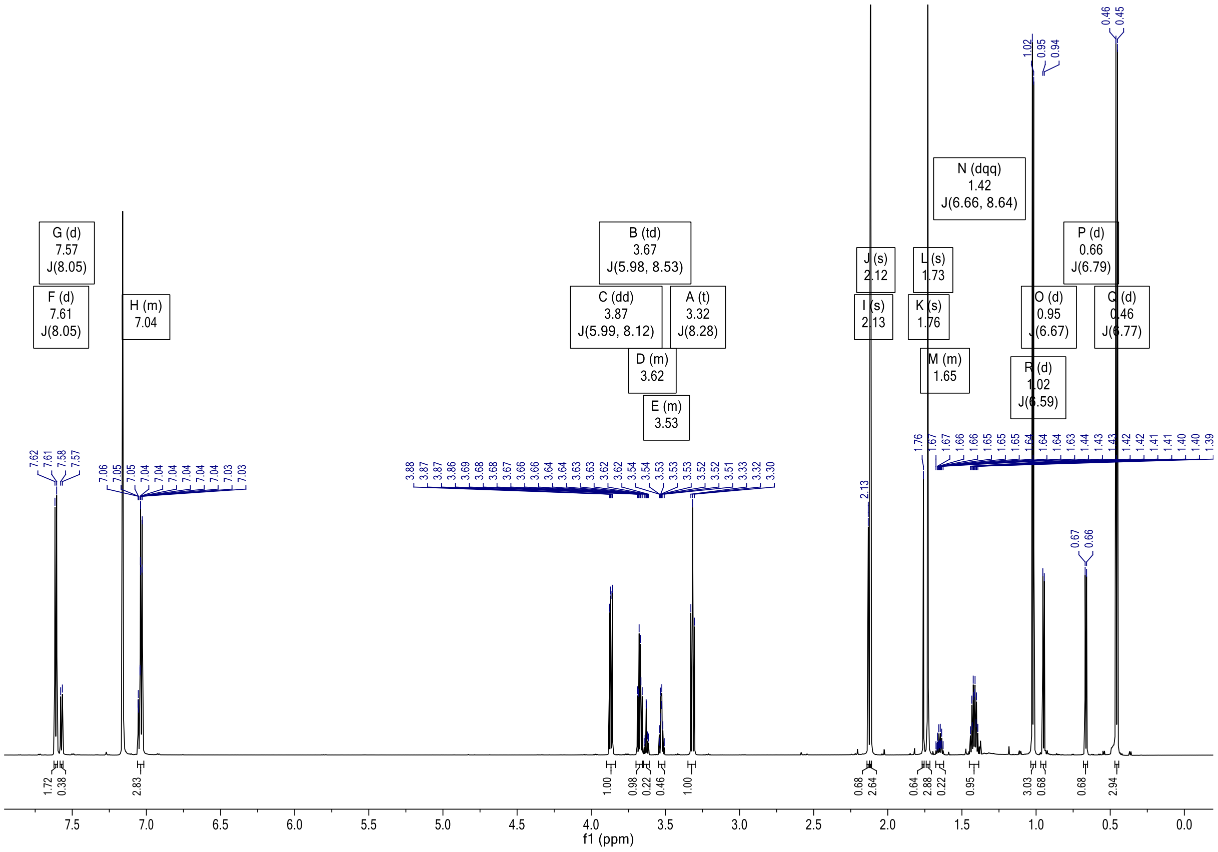

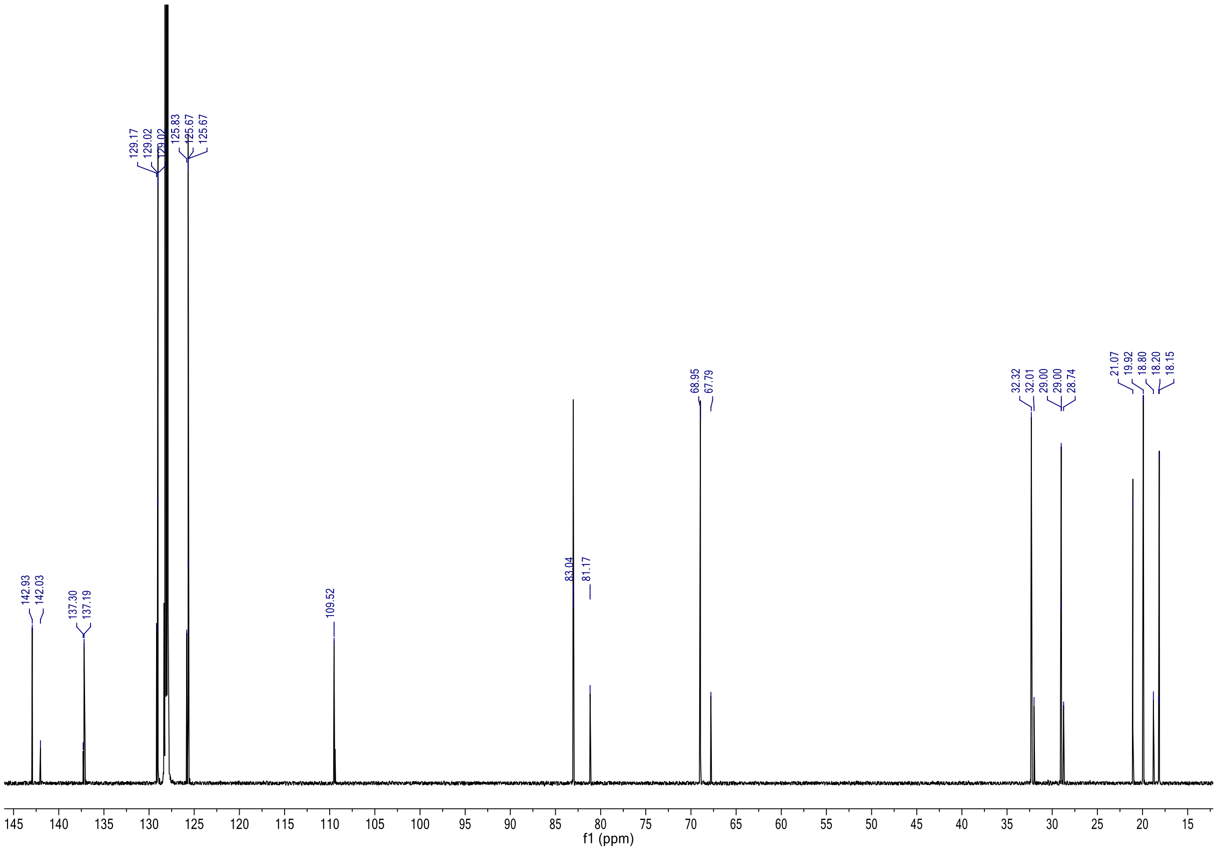

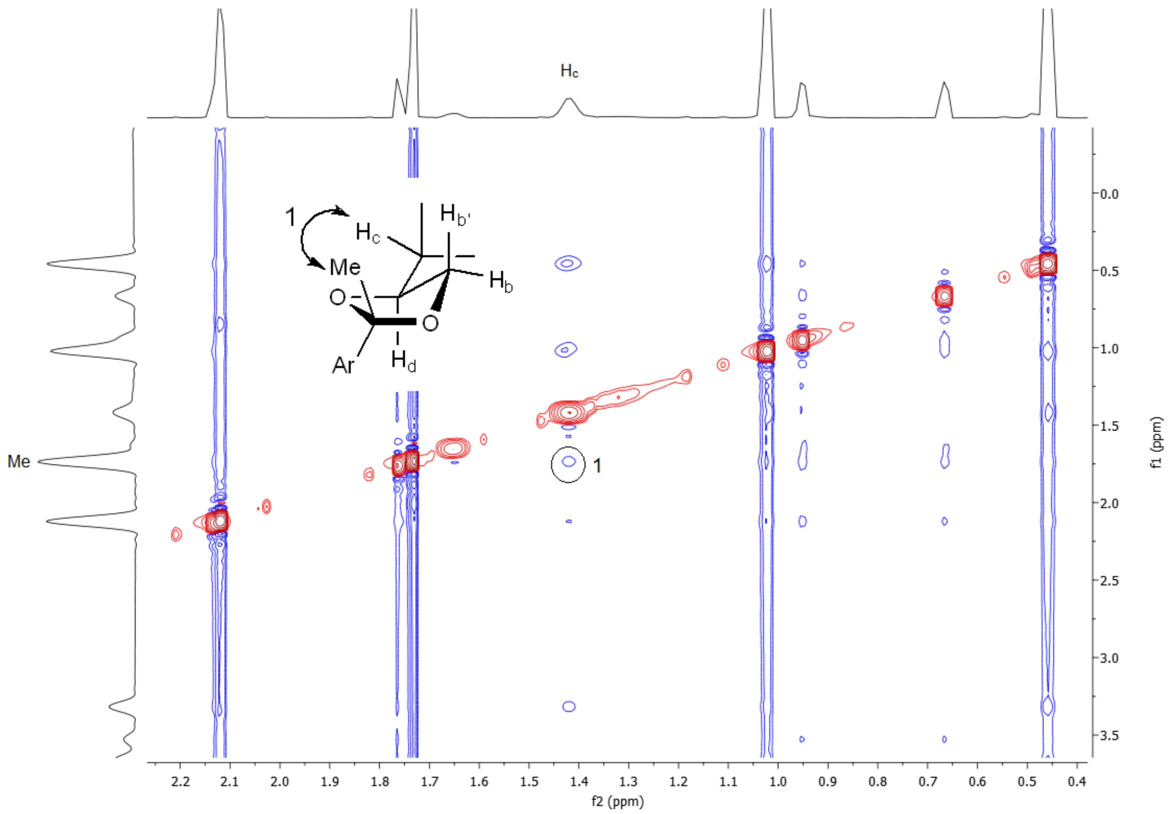


Coupling **1** in combination with the missing coupling between the protons of the Me-group and $H_d$ reveals the relative configuration as depicted.

4.2.5 Synthesis of 2-methyl-2-(4-methoxyphenyl)-4-(propan-2-yl)-1,3-dioxolane **4e**

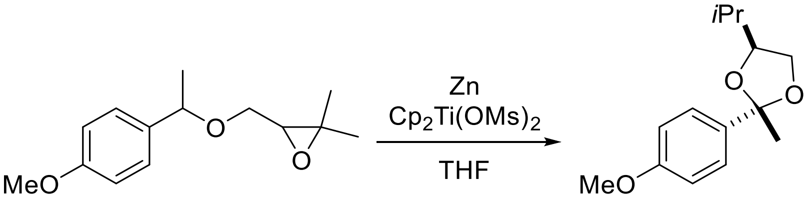


According to GP VII 155.9 mg $Cp_2Ti(OMs)_2$ (0.423 mmol, 0.10 eq.), 55.2 mg zinc dust (0.844 mmol, 0.20 eq.) and 1001.1 mg substrate **3e** (4.24 mmol, 1.00 eq.) are refluxed. Column chromatography ($SiO_2$, eluent: CH:EA, 95:5) afforded 907.7 mg **4e** (d.r. = 62:38, 3.841 mmol, 91%) as a colourless oil.

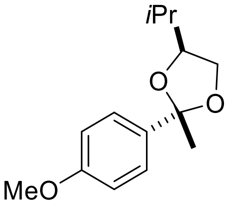


**4e**
$C_{14}H_{20}O_3$
236,31 g/mol

***trans*–Diastereomer (major): $R_f$ =** 0.58 (20% EA, 80% CH). **$^1$H-NMR (300 MHz, $C_6D_6$, RT): δ [ppm] =** 7.60 (d, $J$ = 8.7 Hz, 2H), 6.81 (d, $J$ = 8.7 Hz, 2H), 3.88 (dd, $J$ = 8.0, 6.0 Hz, 1H), 3.68 (*p*td, $J$ = 8.5, 6.0 Hz, 1H), 3.33 (*p*t, $J$ = 8.3 Hz, 1H), 3.31 (s, 3H), 1.74 (s, 3H), 1.44 (dqq, $J$ = 8.5, 6.7, 6.6 Hz, 1H), 1.03 (d, $J$ = 6.6 Hz, 3H), 0.48 (d, $J$ = 6.7 Hz, 3H). **$^{13}$C-NMR (75 MHz, $C_6D_6$, RT): δ [ppm] =**

159.8, 137.9, 126.9, 113.8, 109.5, 83.1, 69.0, 54.8, 32.4, 29.0, 19.9, 18.2. ***cis*–Diastereomer (minor): $R_f$ =** 0.63 (20% EA, 80% CH). **$^1$H-NMR (700 MHz, $C_6D_6$, RT): δ [ppm] =** 7.56 (d, *J* = 8.7 Hz, 2H), 6.83 (d, *J* = 8.7 Hz, 2H), 3.70 – 3.59 (m, 1H), 3.58 – 3.50 (m, 2H), 3.32 (s, 3H), 1.77 (s, 3H), 1.73 – 1.55 (m, 1H), 0.96 (d, *J* = 6.7 Hz, 3H), 0.68 (d, *J* = 6.8 Hz, 3H). **$^{13}$C-NMR (176 MHz, $C_6D_6$, RT): δ [ppm] =** 159.9, 137.0, 127.1, 113.9, 109.3, 81.2, 67.8, 54.8, 32.0, 28.8, 18.8, 18.2. **IR:** 2958, 1611, 1508, 1466, 1370, 1301, 1242, 1200, 1170, 1099, 1033, 1010, 916, 878, 831, 629, 578 $cm^{-1}$. **HRMS (ESI+):** *m/z* calculated for $C_{14}H_{21}O_3^+$: 237.1485 u, found: 237.1486 u.

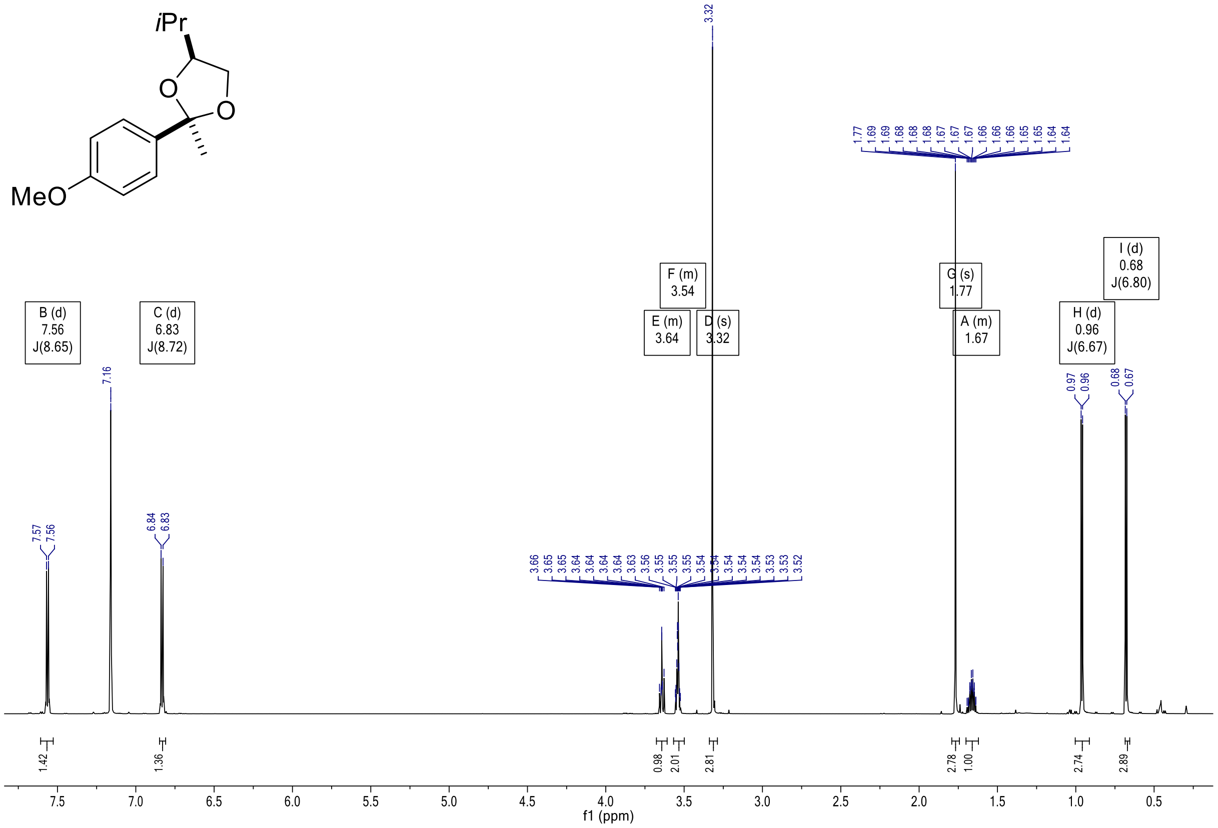

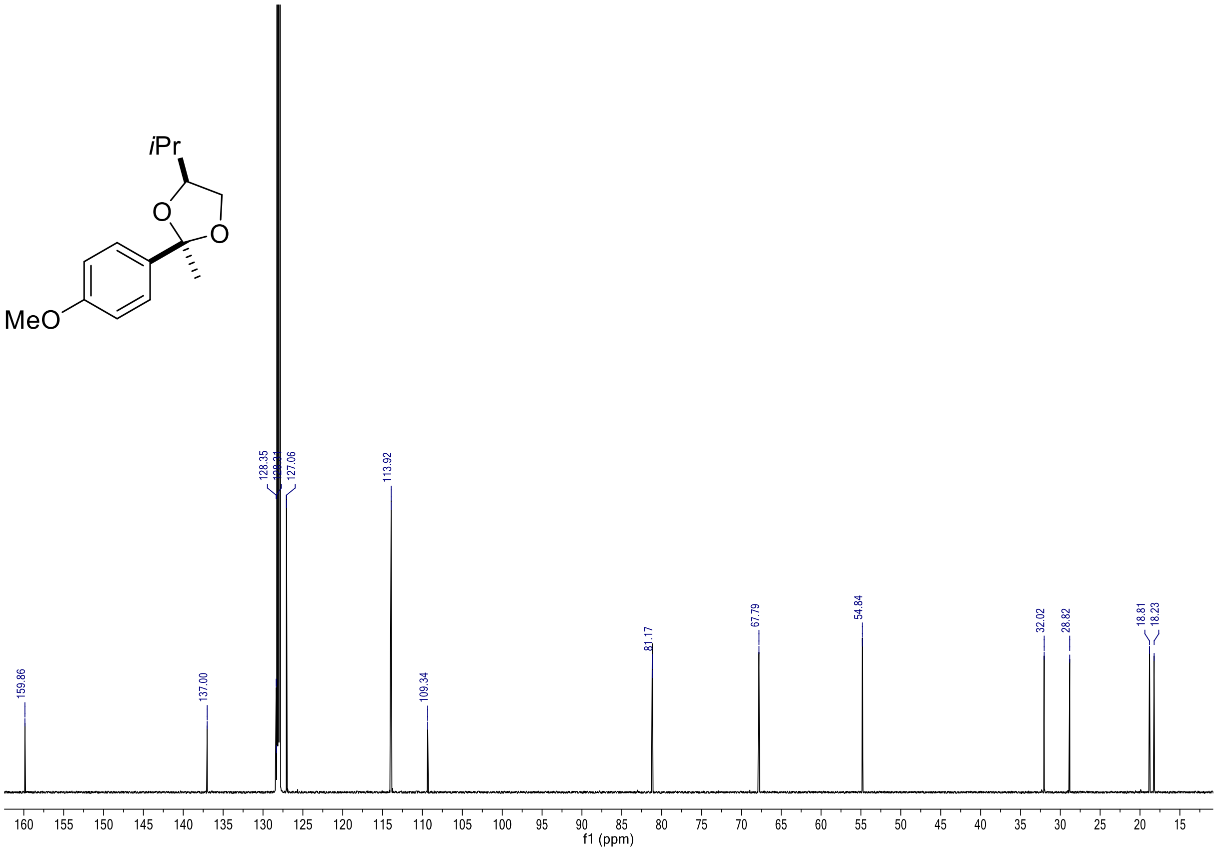

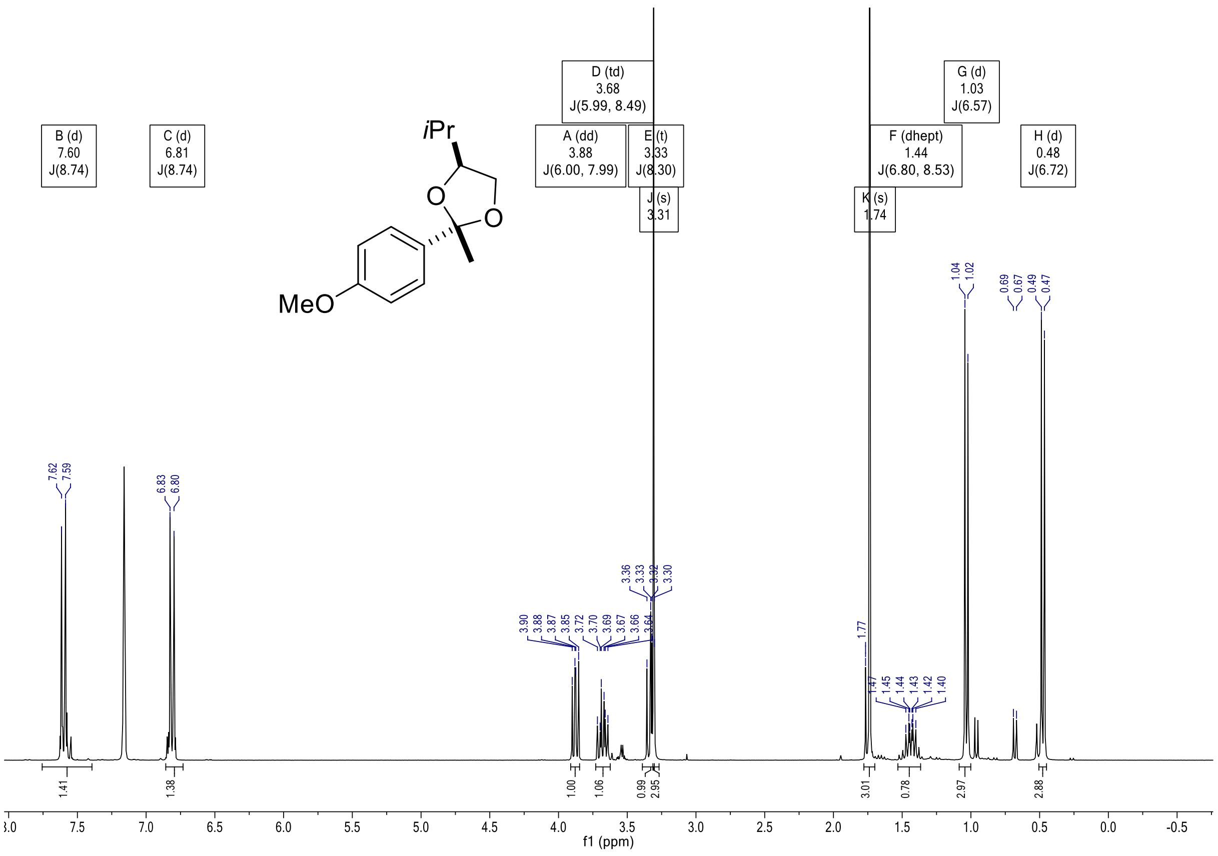

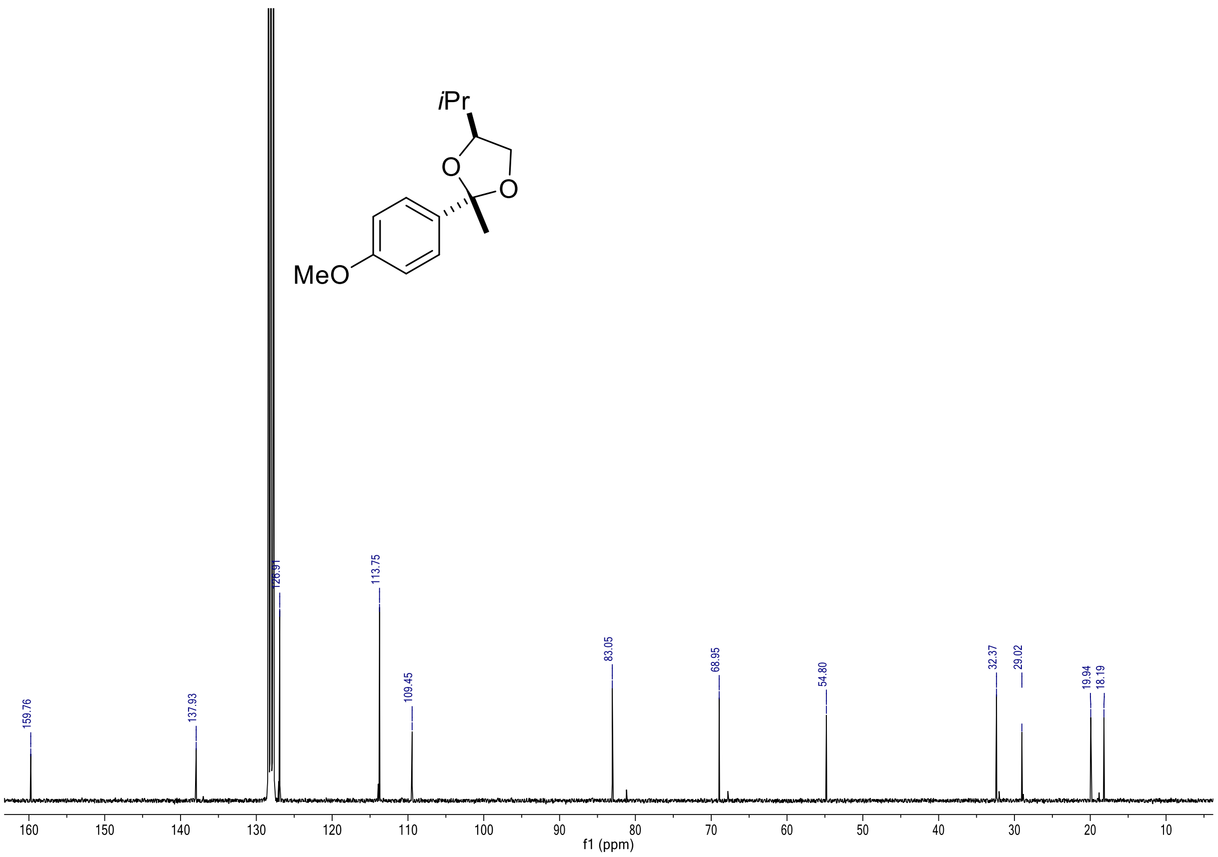


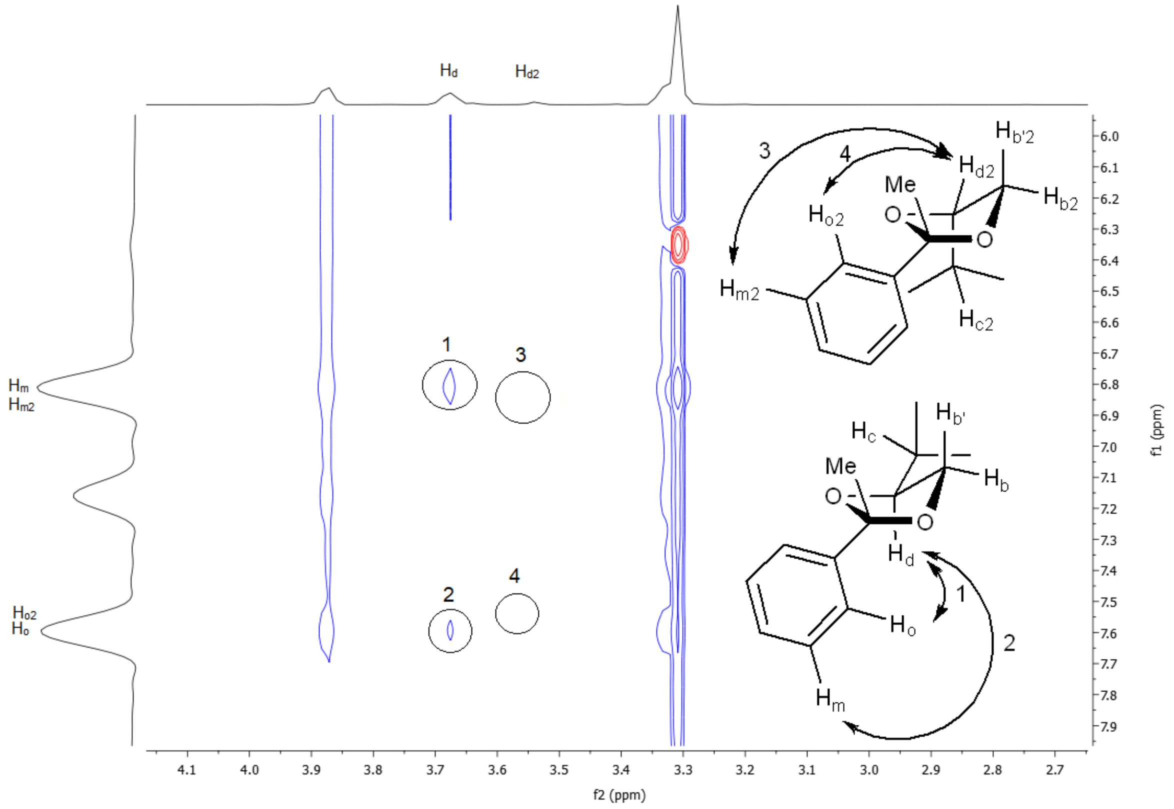


Coupling **1** and **2** in combination with the missing coupling **3** and **4** proves relative configuration of the main diastereomer as depicted in the downer structure.

## 4.3 Titanocene catalyzed synthesis of hemiaminals **6a-6f**

### 4.3.1 Synthesis of 3-methyl-2-phenyl-5-(propan-2-yl)-1,3-oxazolidine **6a**

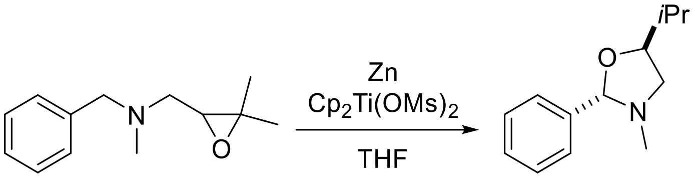


According to GP VII 18.0 mg $Cp_2Ti(OMs)_2$ (0.0489 mmol, 0.10 eq.), 6.7 mg zinc dust (0.102 mmol, 0.21 eq.) and 99.8 mg substrate **5a** (0.486 mmol, 1.00 eq.) are refluxed. Column chromatography ($SiO_2$, eluent: CH:EA:TEA, 90:5:5) was performed rapidly and afforded 81.0 mg **6a** (d.r. = 57:43, 0.395 mmol, 81%) as a colourless oil.

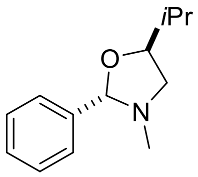


**6a**
$C_{13}H_{19}NO$
205,30 g/mol

**$R_f$ =** 0.33 (5% EA, 5% TEA, 90% CH). ***trans*–Diastereomer (major): $^1$H-NMR (700 MHz, $C_6D_6$, RT): δ [ppm] =** 7.64 – 7.56 (m, 2H), 7.23 – 7.19 (m, 2H), 7.16 – 7.12 (m, 1H), 4.59 (s, 1H), 3.93 (ddd, *J* = 9.2, 8.0, 5.5 Hz, 1H), 3.08 (dd, *J* = 8.9, 5.5 Hz, 1H), 2.09 (*p*t, *J* = 9.1 Hz, 1H), 2.02 (s, 3H), 1.67 (dqq, *J* = 8.0, 6.8, 6.6 Hz, 1H), 1.04 (d, *J* = 6.6 Hz, 3H), 0.74 (d, *J* = 6.8 Hz, 3H). **$^{13}$C-NMR (126 MHz, $C_6D_6$, RT): δ [ppm] =** 140.5, 129.0, 128.7, 128.4, 98.8, 82.9, 59.9, 37.8, 33.3, 19.6, 18.3. ***cis*-Diastereomer (minor): $^1$H-NMR (700 MHz, $C_6D_6$, RT): δ [ppm] =** 7.64 – 7.56 (m, 2H), 7.23 – 7.19 (m, 2H), 7.16 – 7.12 (m, 1H), 4.61 (s, 1H), 3.70 (ddd, *J* = 7.9, 7.3, 4.3 Hz, 1H), 2.89 (dd, *J* = 9.3, 4.3 Hz, 1H), 2.33 (dd, *J* = 9.3, 7.9 Hz, 1H), 2.02 (s, 3H), 1.81 (dqq, *J* = 7.3, 6.8, 6.7 Hz, 1H), 1.06 (d, *J* = 6.7 Hz, 3H), 0.83 (d, *J* = 6.8 Hz, 3H). **$^{13}$C-NMR (126 MHz, $C_6D_6$, RT): δ [ppm] =** 140.3, 129.0, 128.6, 128.4, 99.3, 81.8, 57.5, 38.4, 33.4, 19.0, 18.9. **IR:** 2957, 2872, 1456, 1383, 1263, 1154, 1080, 1052, 1027, 999, 932, 845, 756, 697 $cm^{-1}$. **HRMS (ESI+):** *m/z* calculated for $C_{13}H_{20}NO^+$: 206.1539 u, found: 206.1537 u.

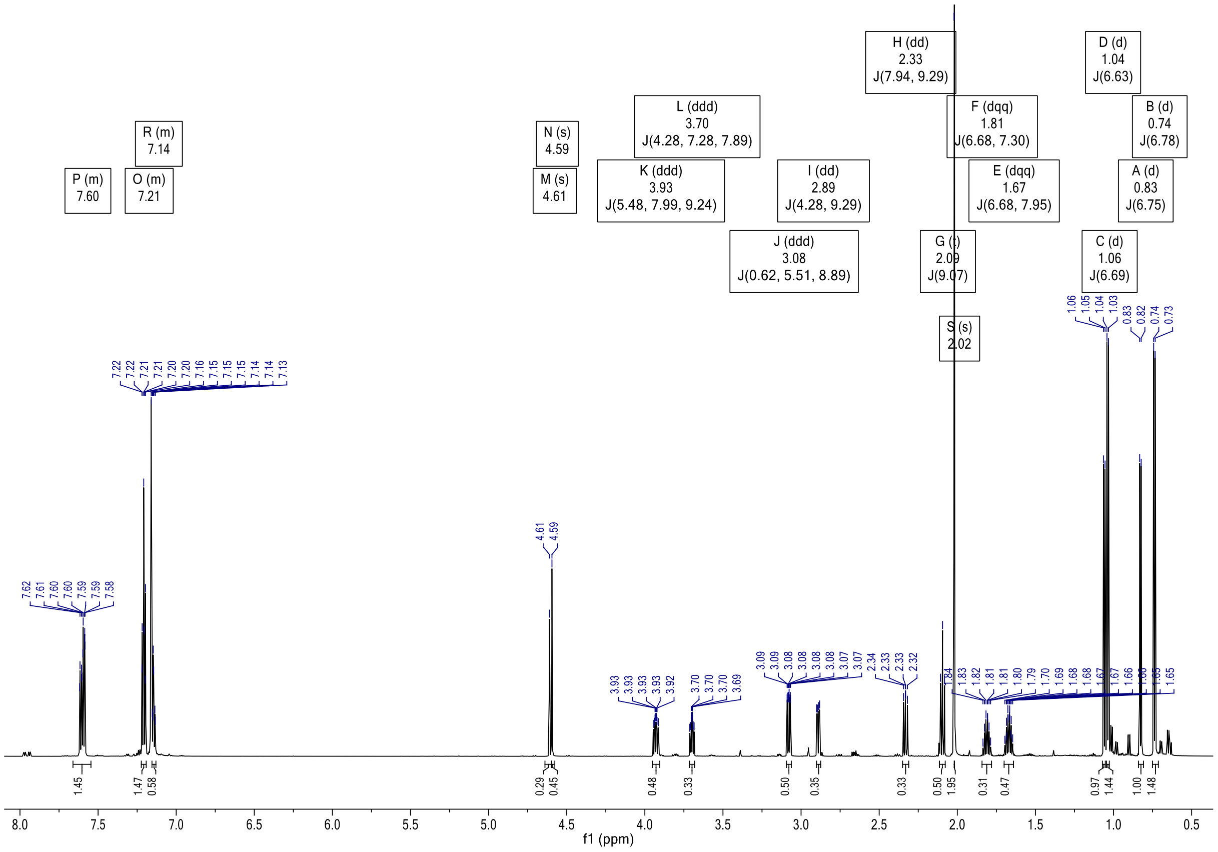

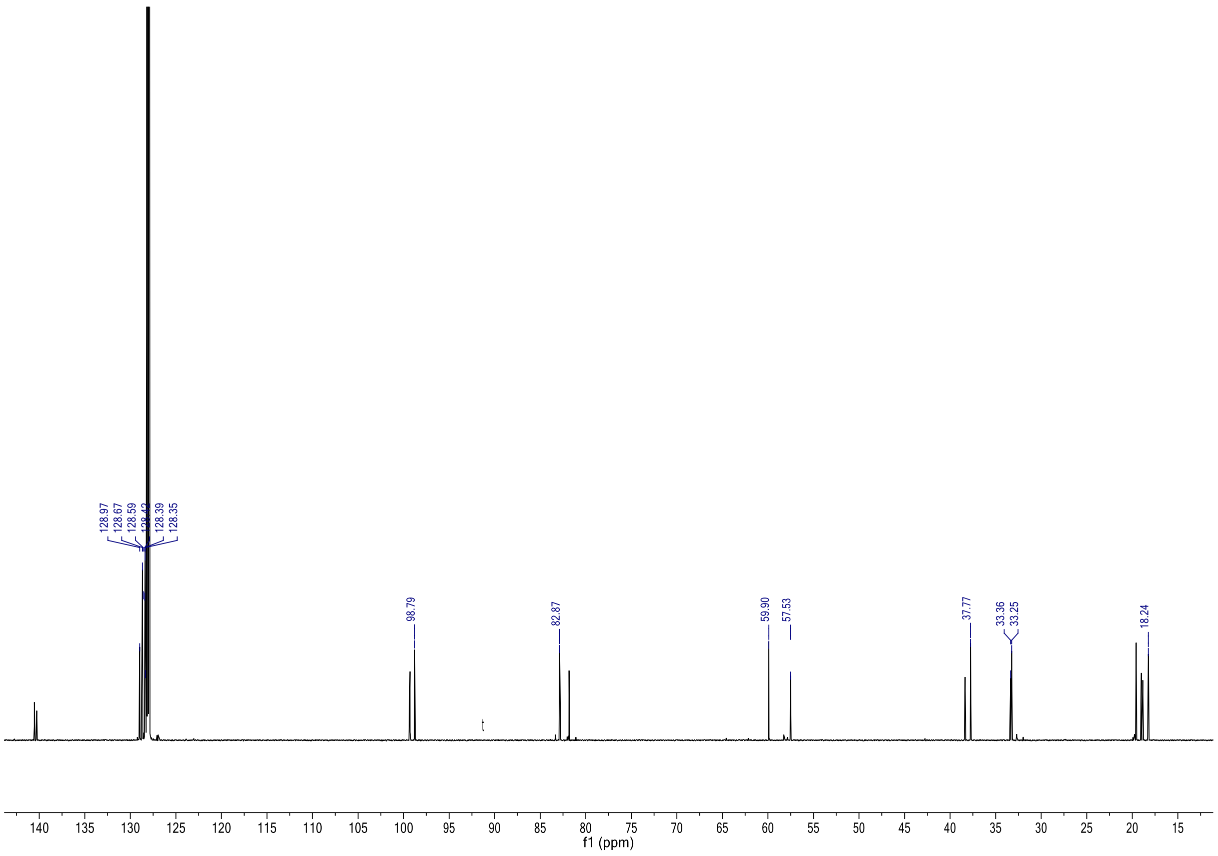

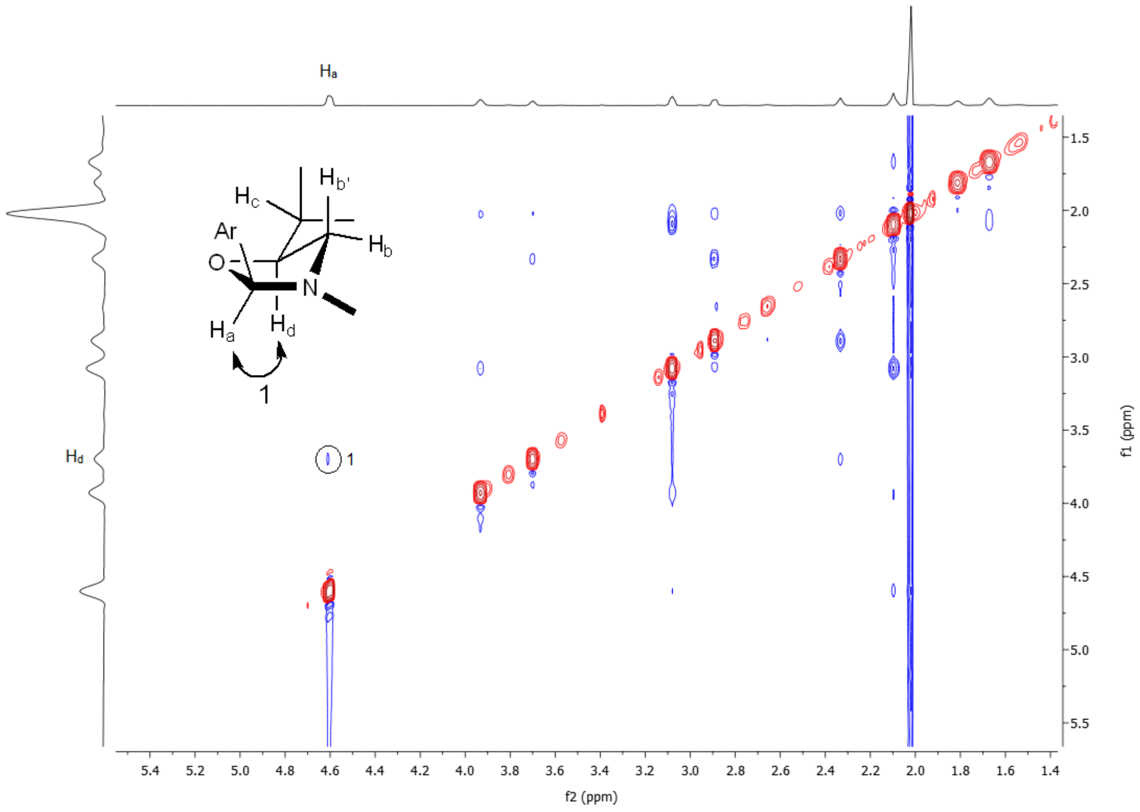


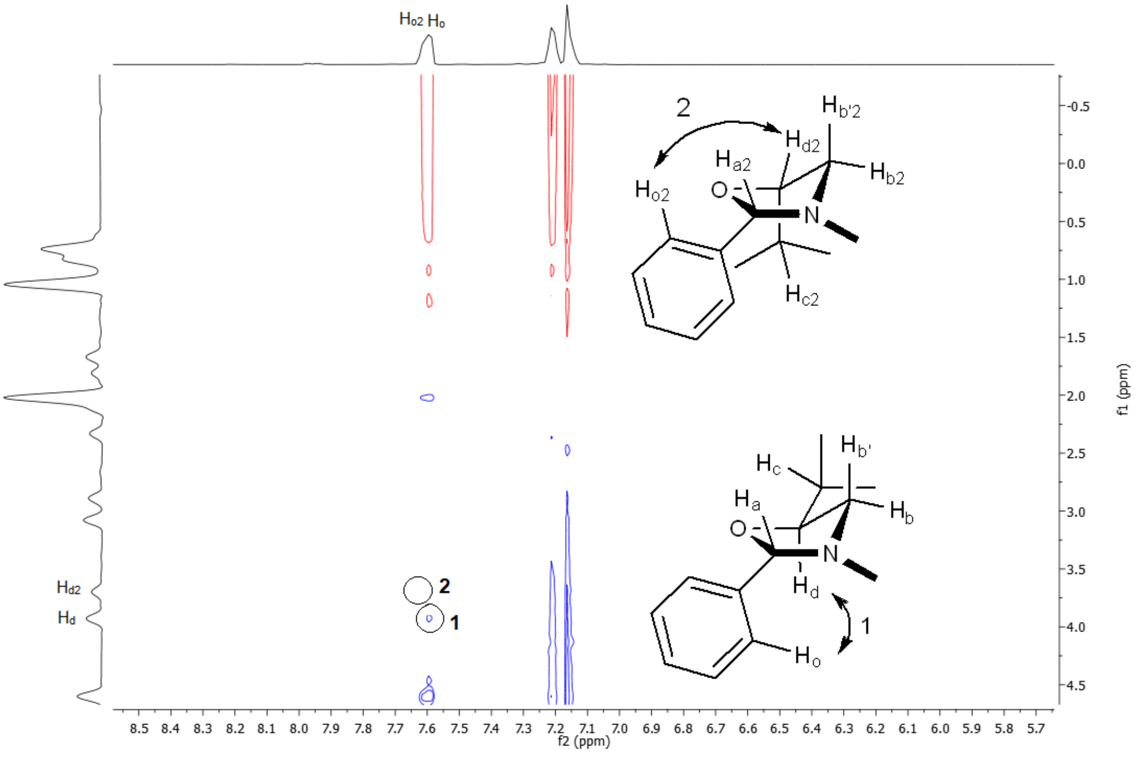


Coupling **1** and the missing coupling **2** indicate a 2,4-*trans*-configuration of the main diastereomer and a *cis*-configuration of the minor diastereomer (upper structure).

4.3.2 Synthesis of 3-benzyl-2-phenyl-5-(propan-2-yl)-1,3-oxazolidine **6b**

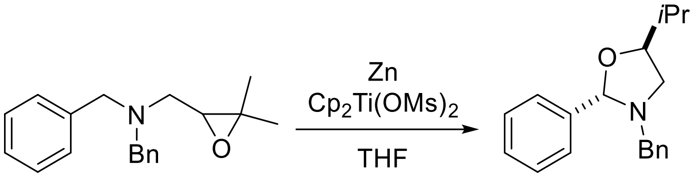


According to GP VII 27.0 mg $Cp_2Ti(OMs)_2$ (0.0733 mmol, 0.10 eq.), 9.6 mg zinc dust (0.147 mmol, 0.20 eq.) and 202.4 mg substrate **5b** (0.719 mmol, 1.00 eq.) are refluxed. Column chromatography ($SiO_2$, eluent: CH:EA:TEA, 90:5:5) afforded 152.9 mg **6b** (d.r. = 56:44, 0.544 mmol, 76%) as a colourless oil.

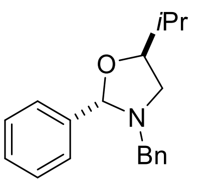


**6b**
$C_{19}H_{23}NO$
281,40 g/mol

**$R_f$ =** 0.57 (20% EA, 80% CH). ***trans*-Diastereomer (major): $^{1}$H-NMR (500 MHz, $C_6D_6$, RT): δ [ppm] =** 7.75 – 7.64 (m, 4H), 7.32 – 7.04 (m, 6H), 4.91 (s, 1H), 3.87 (ddd, *J* = 9.0, 7.9, 5.6 Hz, 1H), 3.75 – 3.68 (m, 1H), 3.15 (dd, *J* = 9.1, 5.6 Hz, 1H), 3.01 (d, *J* = 13.0 Hz, 1H), 2.10 (*p*t, *J* = 9.1 Hz, 1H), 1.79 – 1.57 (m, 1H), 1.01 (d, *J* = 6.6 Hz, 3H), 0.65 (d, *J* = 6.7 Hz, 3H). **$^{13}$C-NMR (126 MHz, $C_6D_6$, RT): δ [ppm]** = 140.7, 139.5, 129.0, 128.9, 128.8, 128.6, 128.5, 127.3, 97.3, 83.0, 56.7, 56.1, 33.3, 19.5, 18.2. ***cis*-Diastereomer (minor): $^{1}$H-NMR (500 MHz, $C_6D_6$, RT): δ [ppm] =** 7.75 – 7.64 (m, 4H), 7.32 – 7.04 (m, 6H), 4.96 (s, 1H), 3.75 – 3.68 (m, 2H), 3.10 (d, *J* = 13.3 Hz, 1H), 2.85 (dd, *J* = 9.8, 5.2 Hz, 1H), 2.41 (dd, *J* = 9.8, 7.7 Hz, 1H), 1.79 – 1.57 (m, 1H), 1.03 (d, *J* = 6.7 Hz, 3H), 0.72 (d, *J* = 6.8 Hz, 3H). **$^{13}$C-NMR (126 MHz, $C_6D_6$, RT): δ [ppm] =** 140.6, 139.7, 128.9, 128.7, 128.6, 128.6, 128.5, 127.3, 97.6, 82.0, 56.7, 55.0, 33.4, 19.1, 18.7. **IR:** 2958, 1494, 1455, 1384, 1368, 1306, 1166, 1118, 1057, 1026, 999, 932, 913, 848, 758, 738, 696, 639 $cm^{-1}$. **HRMS (ESI+):** *m/z* calculated for $C_{19}H_{24}ON^{+}$: 282.1852 u, found: 282.1855 u.

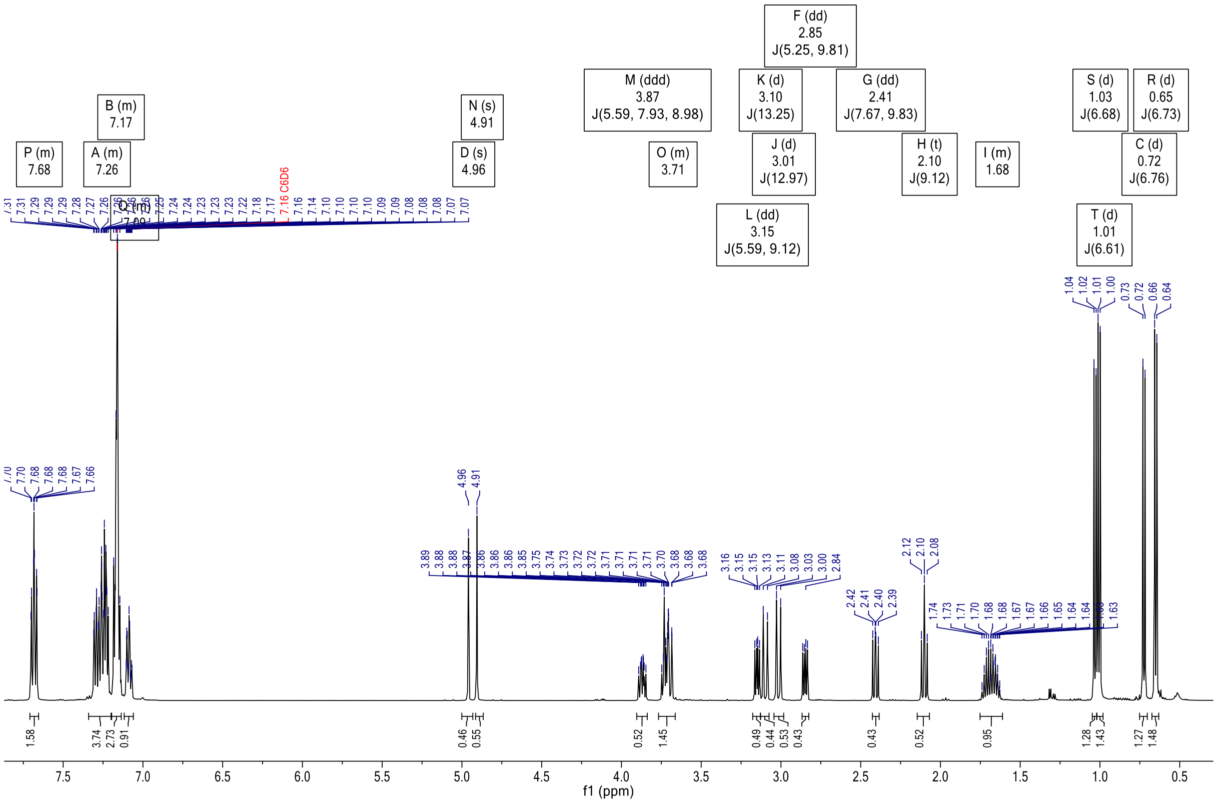

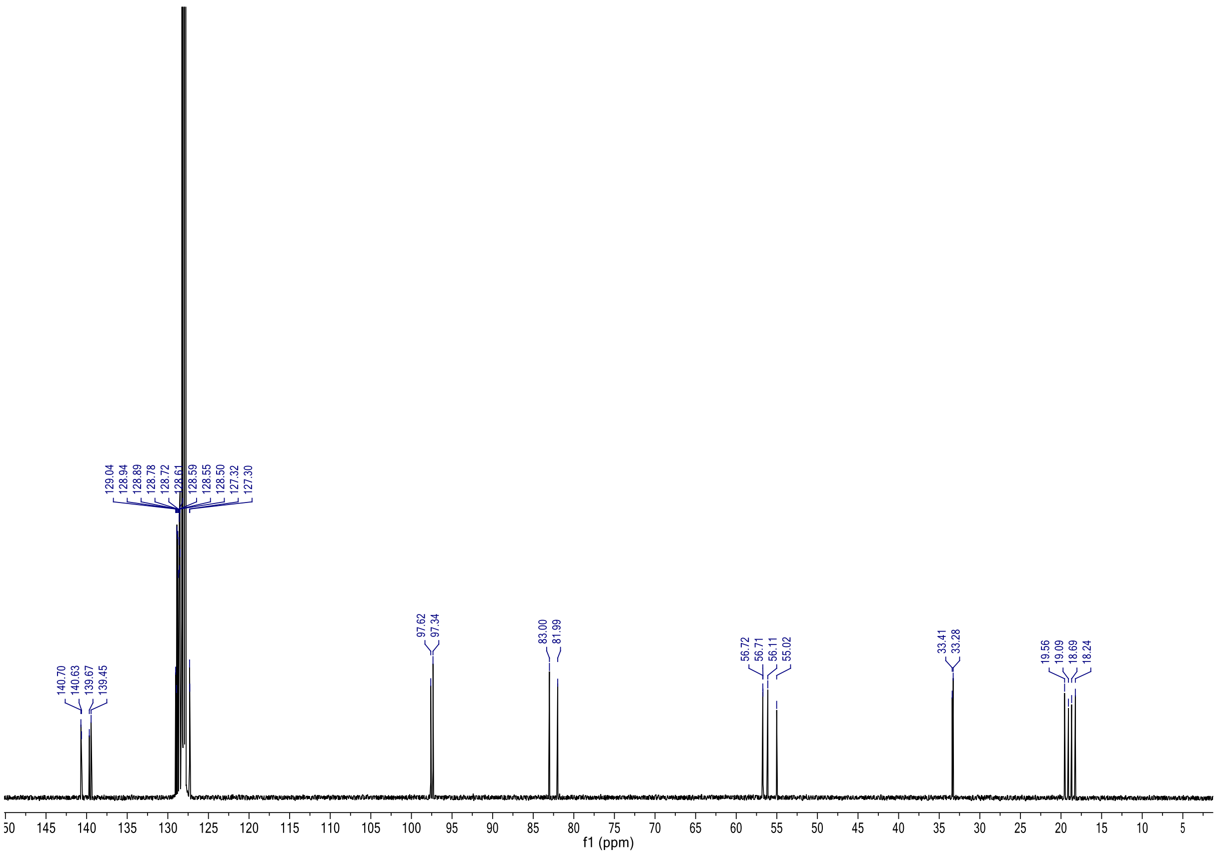

### 4.3.3 Synthesis of 3-methyl-2-phenyl-5-(propan-2-yl)-1,3-oxazolidine **6c**

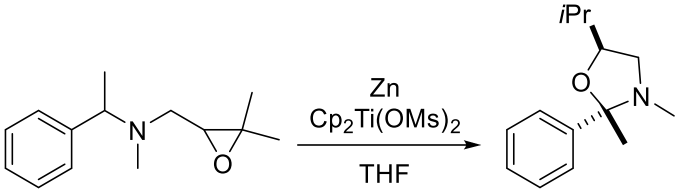


According to GP VIII 16.8 mg $Cp_2Ti(OMs)_2$ (0.0456 mmol, 0.10 eq.), 6.2 mg zinc dust (0.0948 mmol, 0.21 eq.) and 100.0 mg substrate **5c** (0.456 mmol, 1.00 eq.) are refluxed. Work up afforded 73.8 mg **6c** (d.r. = 52:48, 0.336 mmol, 74%) as a light yellow oil.

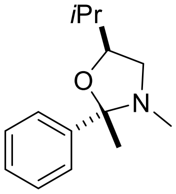


**6c**
$C_{14}H_{21}NO$
219,33 g/mol

**$R_f$ =** 0.30 (2% EA, 5% TEA, 93% CH). ***trans*-Diastereomer (major): $^1$H-NMR (500 MHz, $C_6D_6$, RT): δ [ppm] =** 7.69 – 7.66 (m, 2H), 7.27 – 7.20 (m, 2H), 7.15 – 7.08 (m, 1H), 3.54 (d*p*t, *J* = 8.1, 6.7 Hz, 1H), 2.86 (dd, *J* = 9.9, 6.7 Hz, 1H), 2.36 (dd, *J* = 9.9, 6.8 Hz, 1H), 2.20 (s, 3H), 1.67 (dqq, *J* = 8.1, 6.7, 6.6 Hz, 1H), 1.48 (s, 3H), 1.06 (d, *J* = 6.6 Hz, 3H), 0.73 (d, *J* = 6.7 Hz, 3H). **$^{13}$C-NMR (125 MHz, $C_6D_6$, RT): δ [ppm] =** 147.4, 128.1, 127.5, 126.8, 99.1, 81.1, 57.5, 37.8, 33.7, 23.7, 19.8, 18.8. ***cis*-Diastereomer (minor): $^1$H-NMR (500 MHz, $C_6D_6$, RT): δ [ppm] =** 7.78 – 7.74 (m, 2H), 7.27 – 7.20 (m, 2H), 7.15 – 7.08 (m, 1H), 3.65 (ddd, *J* = 9.1, 8.3, 6.2 Hz, 1H), 2.63 (dd, *J* = 11.0, 6.2 Hz, 1H), 2.59 (dd, *J* = 11.0, 8.3 Hz, 1H), 2.21 (s, 3H), 1.52 – 1.42 (m, 1H), 1.50 (s, 3H), 1.17 (d, *J* = 6.5 Hz, 3H), 0.54 (d, *J* = 6.7 Hz, 3H). **$^{13}$C-NMR (125 MHz, $C_6D_6$, RT): δ [ppm] =** 145.9, 128.2, 127.4, 126.7, 97.6, 80.9, 58.9, 37.8, 33.8, 24.9, 20.6, 18.6. **IR:** 2957, 2870, 1468, 1447, 1367, 1221, 1171, 1066, 1036, 1015, 977, 839, 763, 700 $cm^{-1}$. **HRMS (ESI+):** *m/z* calculated for $C_{14}H_{22}NO^+$: 220.1696 u, found: 220.1696 u.

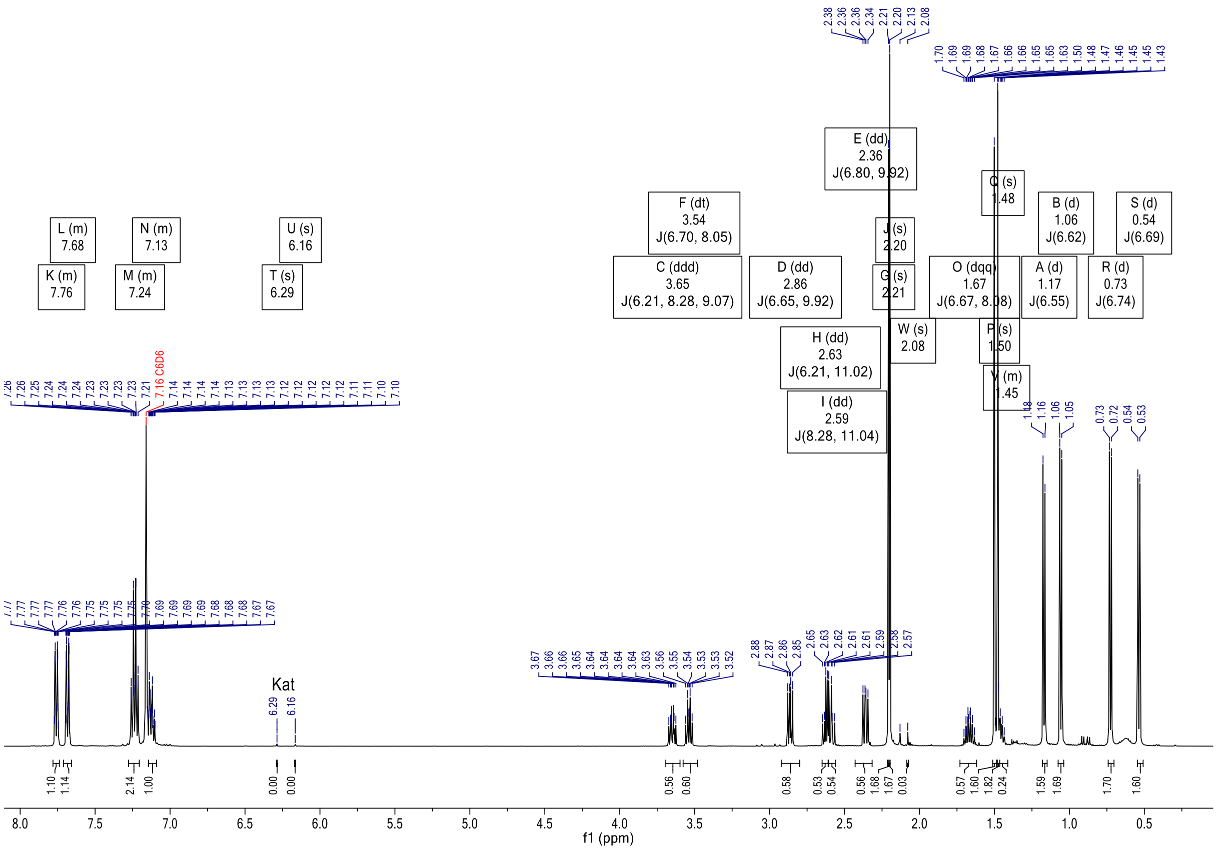

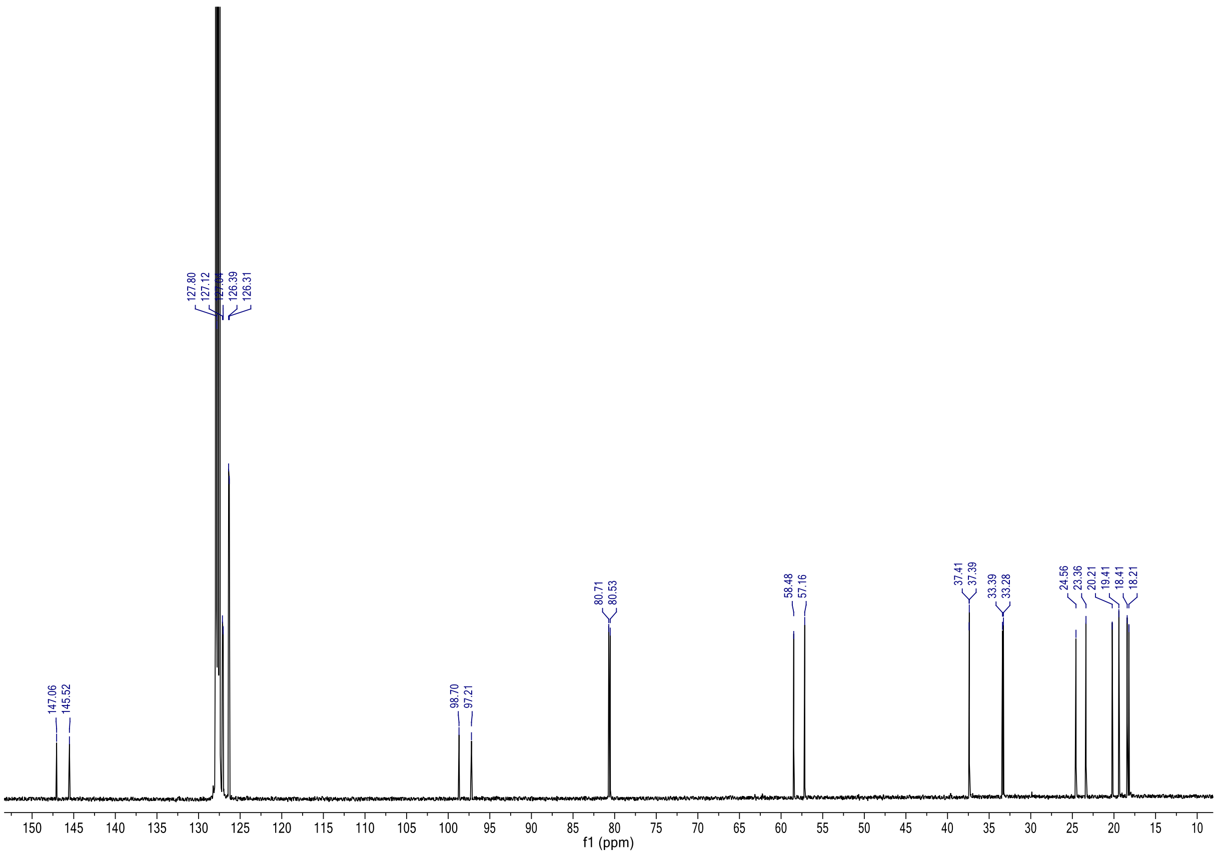

4.3.4 Synthesis of 2,3-dimethyl-2-(4-methylphenyl)-5-(propan-2-yl)-1,3-oxazolidine **6d**

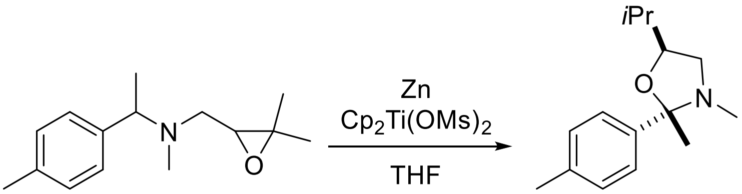


According to GP VIII 15.8 mg $Cp_2Ti(OMs)_2$ (0.0429 mmol, 0.10 eq.), 5.6 mg zinc dust (0.0857 mmol, 0.20 eq.) and 100.5 mg substrate **5d** (0.431 mmol, 1.00 eq.) are refluxed. Work up afforded 77.1 mg **6d** (d.r. = 50:50, 0.330 mmol, 77%) as a light yellow oil.

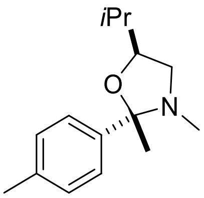


**6d**
$C_{15}H_{23}NO$
233,36 g/mol

**$R_f$ =** 0.30 (5% TEA, 5% EA, 90% CH). ***trans*-Diastereomer: $^1$H-NMR (500 MHz, $C_6D_6$, RT): δ [ppm] =** 7.63 – 7.60 (m, 2H), 7.10 – 7.07 (m, 2H), 3.59 (d*p*t, *J* = 8.1, 6.7 Hz, 1H), 2.90 (dd, *J* = 9.9, 6.7 Hz, 1H), 2.40 (dd, *J* = 9.9, 6.8 Hz, 1H), 2.22 (s, 3H), 2.16 (s, 3H), 1.69 (dqq, *J* = 8.1, 6.7, 6.6 Hz, 1H) 1.51 (s, 3H), 1.07 (d, *J* = 6.6 Hz, 3H), 0.75 (d, *J* = 6.7 Hz, 3H). **$^{13}$C-NMR (125 MHz, $C_6D_6$, RT): δ [ppm]** = 144.5, 136.7, 128.9, 126.8, 99.0, 81.1, 57.5, 37.7, 33.7, 23.8, 21.1, 19.8, 18.8. ***cis*-Diastereomer: $^1$H-NMR (500 MHz, $C_6D_6$, RT): δ [ppm] =** 7.70 – 7.67 (m, 2H), 7.08 – 7.04 (m, 2H), 3.67 (ddd, *J* = 9.0, 7.7, 6.6 Hz, 1H), 2.66 – 2.64 (m, 2H), 2.23 (s, 3H), 2.15 (s, 3H), 1.60 – 1.43 (m, 1H), 1.53 (s, 3H), 1.18 (d, *J* = 6.5 Hz, 3H), 0.57 (d, *J* = 6.7 Hz, 3H). **$^{13}$C-NMR (125 MHz, $C_6D_6$, RT): δ [ppm] =** 142.9, 136.8, 128.8, 126.7, 97.6, 80.9, 58.9, 37.8, 33.8, 24.8, 21.1, 20.6, 18.6. **IR:** 2957, 2870, 1686, 1451, 1366, 1267, 1222, 1176, 1093, 1037, 1018, 977, 815, 569 $cm^{-1}$. **HRMS (ESI+):** *m/z* calculated for $C_{15}H_{24}NO^+$: 234.1852 u, found: 234.1853 u.

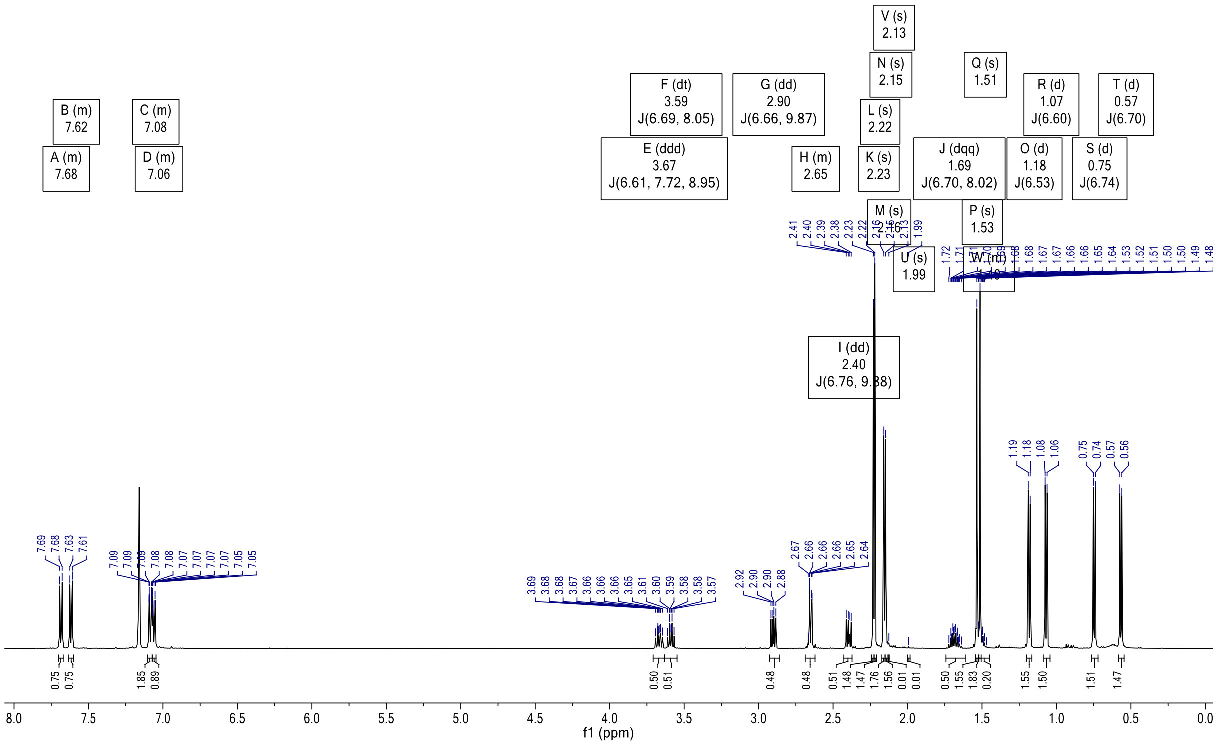

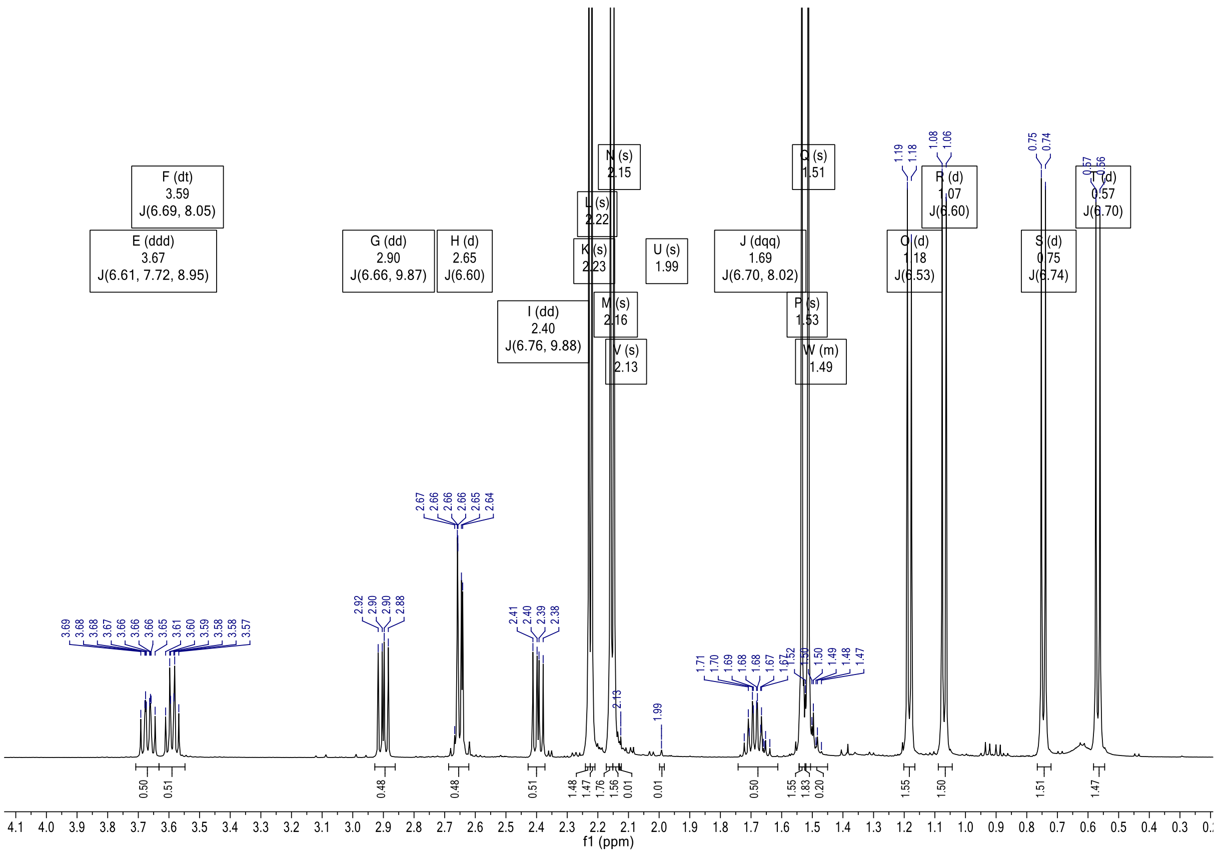

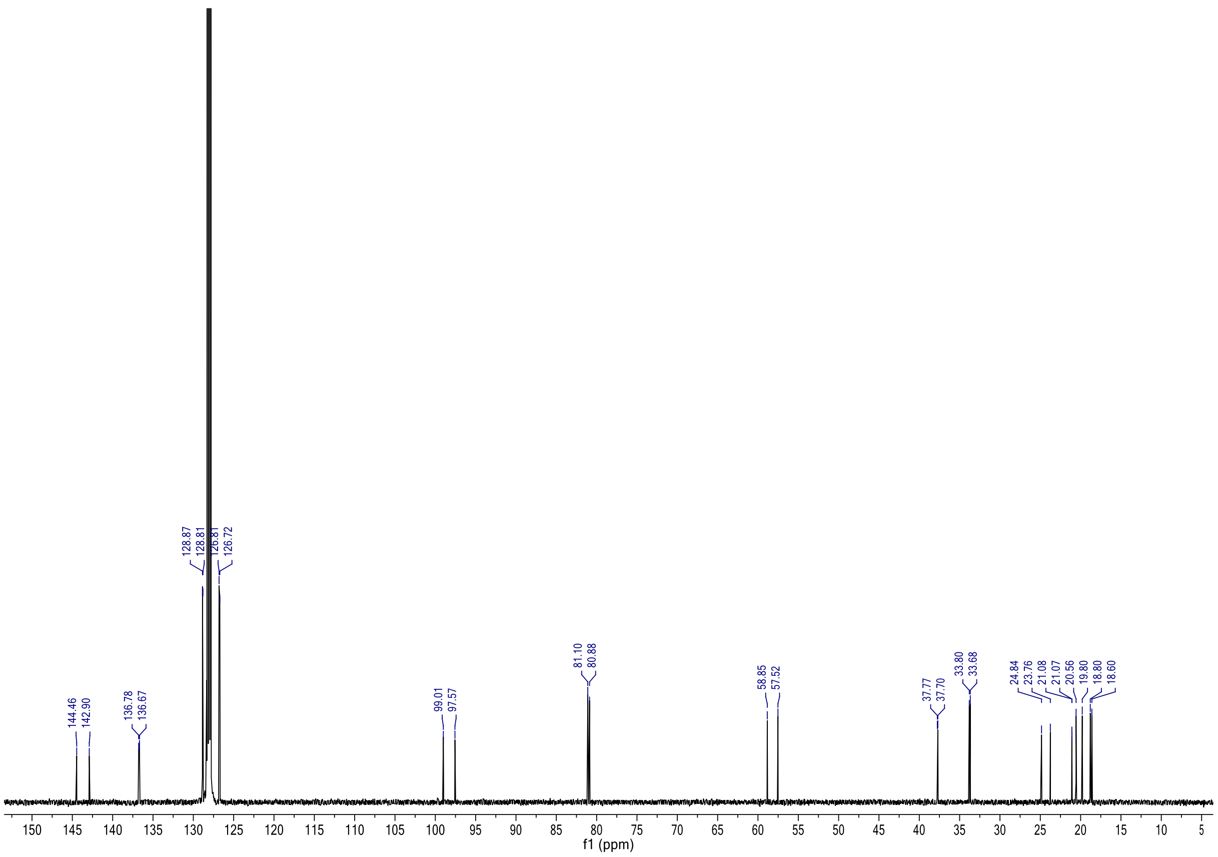

### 4.3.5 Synthesis of 2,3-dimethyl-2-(3-methoxyphenyl)-5-(propan-2-yl)-1,3-oxazolidine **6e**

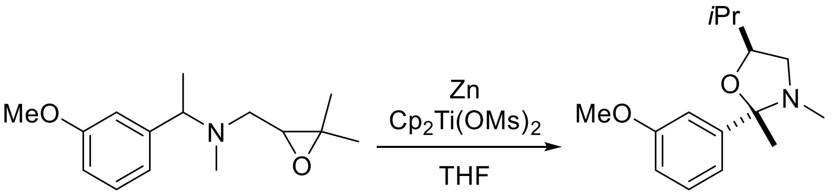

According to GP VIII14.8 mg $Cp_2Ti(OMs)_2$ (0.0402 mmol, 0.10 eq.), 5.1 mg zinc dust (0.0780 mmol, 0.19 eq.) and 100.5 mg substrate **5e** (0.403 mmol, 1.00 eq.) are refluxed. Work up afforded 72.1 mg **6e** (d.r. = 52:48, 0.289 mmol, 72%) as a light yellow oil.

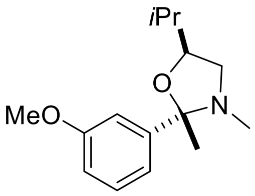

**6e**
$C_{15}H_{23}NO_2$
249,35 g/mol

**$R_f$ =** 0.29 (5% EA, 5% TEA, 90% CH). ***trans*-Diastereomer: $^1$H-NMR (500 MHz, $C_6D_6$, RT): δ [ppm] =** 7.45 (dd, *J* = 2.6, 1.6 Hz, 1H), 7.32 (ddd, *J* = 7.7, 1.6, 1.0 Hz, 1H), 7.21 – 7.14 (m, 1H), 6.78 – 6.74 (m, 1H), 3.55 (d*pt*, *J* = 8.1, 6.7 Hz, 1H), 3.40 (s, 3H), 2.90 (dd, *J* = 10.0, 6.7 Hz, 1H), 2.36 (dd, *J* = 10.0, 6.7 Hz, 1H), 2.23 (s, 3H), 1.50 (s, 3H), 1.66 (dqqq, *J* = 8.1, 6.7, 6.6 Hz, 1H), 1.05 (d, *J* = 6.6 Hz, 3H), 0.72 (d, *J* = 6.7 Hz, 3H). **$^{13}$C-NMR (125 MHz, $C_6D_6$, RT): δ [ppm] =** 160.3,

147.7, 129.2, 119.2, 112.9, 112.8, 97.7, 81.0, 57.6, 54.8, 38.0, 33.7, 23.9, 19.8, 18.8. ***cis*-Diastereomer: $^{1}$H-NMR (500 MHz, $C_6D_6$, RT): δ [ppm] =** 7.50 (dd, *J* = 2.7, 1.6 Hz, 1H), 7.39 (ddd, *J* = 7.7, 1.6, 1.0 Hz, 1H), 7.21 – 7.14 (m, 1H), 6.78 – 6.74 (m, 1H), 3.65 (ddd, *J* = 9.1, 7.7, 6.9 Hz, 1H), 3.41 (s, 3H), 2.65 – 2.62 (m, 2H), 2.23 (s, 3H), 1.53 (s, 3H), 1.56 – 1.45 (m, 1H), 1.18 (d, *J* = 6.5 Hz, 3H), 0.53 (d, *J* = 6.7 Hz, 3H). **$^{13}$C-NMR (125 MHz, $C_6D_6$, RT): δ [ppm] =** 160.1, 149.4, 129.1, 119.1, 113.0, 112.5, 99.2, 80.9, 59.0, 54.7, 37.9, 33.7, 25.1, 20.6, 18.6. **IR:** 2956, 2870, 1599, 1583, 1484, 1465, 1432, 1367, 1282, 1261, 1235, 1165, 1056, 977, 844, 781, 702, 587 $cm^{-1}$. **HRMS (ESI+):** *m/z* calculated for $C_{15}H_{24}NO_2^+$: 250.1802 u, found: 250.1799 u.

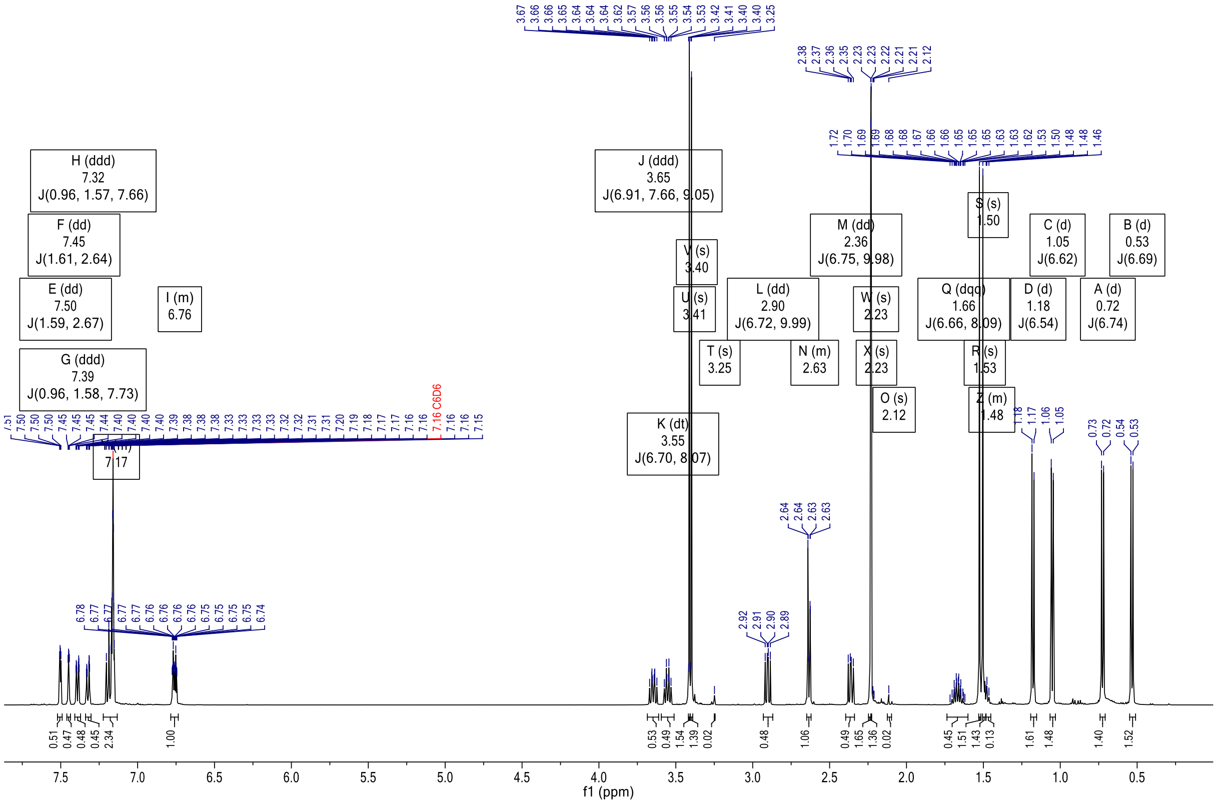

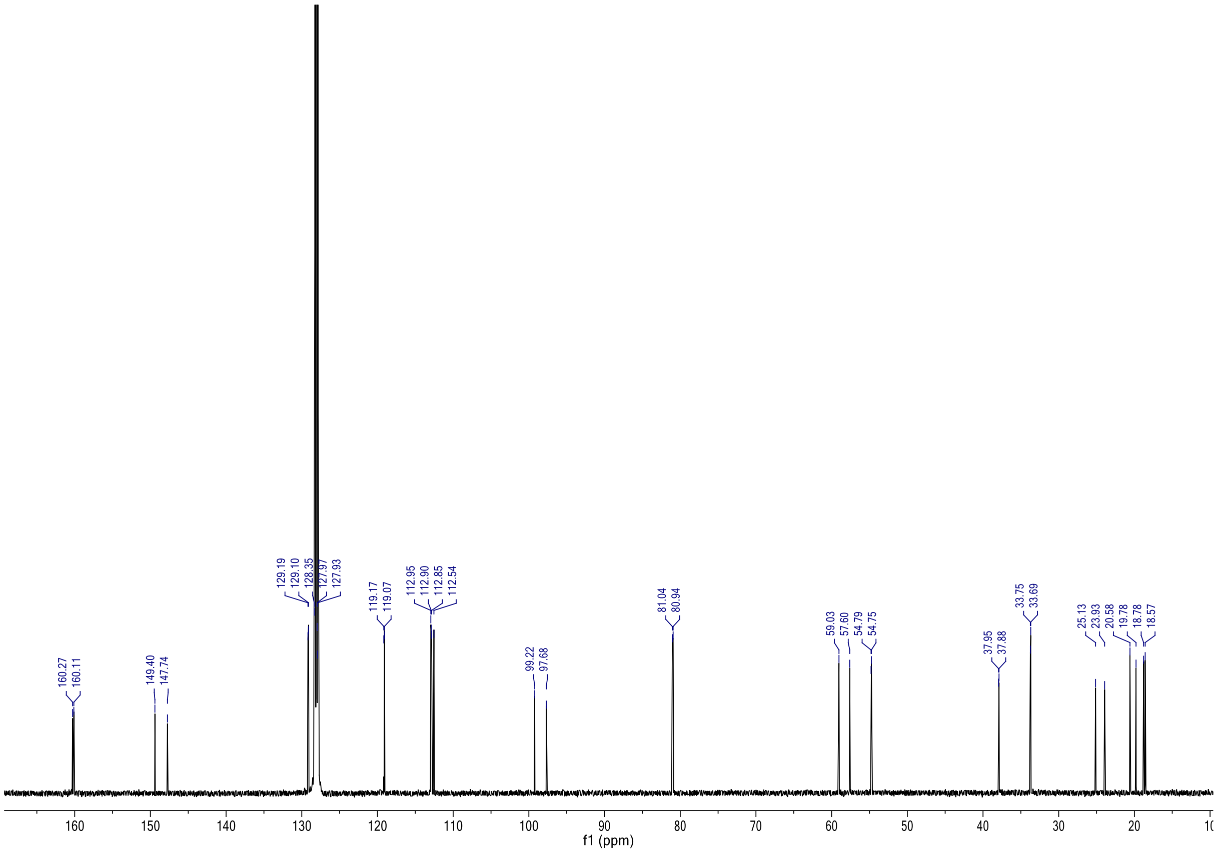


### 4.3.6 Synthesis of 2,3-dimethyl-2-(4-methoxyphenyl)-5-(propan-2-yl)-1,3-oxazolidine **6f**

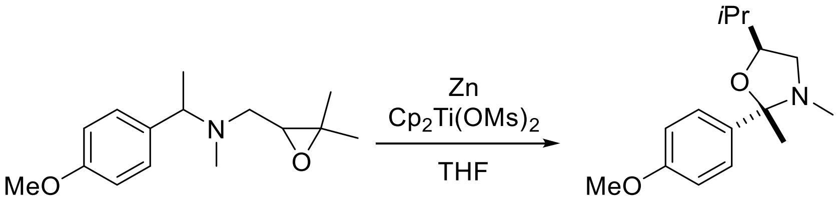


According to GP VIII 14.8 mg $Cp_2Ti(OMs)_2$ (0.0402 mmol, 0.10 eq.), 5.2 mg zinc dust (0.0795 mmol, 0.20 eq.) and 99.7 mg substrate **5f** (0.400 mmol, 1.00 eq.) are refluxed. Work up afforded 72.9 mg **6f** (d.r. = 50:50, 0.292 mmol, 73%) as a light yellow oil.

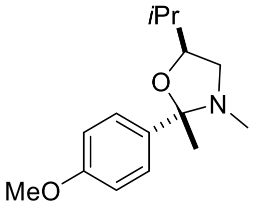


**6f**
$C_{15}H_{23}NO_2$
249,35 g/mol

**$R_f$** = 0.30 (5% EA, 5% TEA, 90% CH). ***trans*-Diastereomer: $^1$H-NMR (500 MHz, $C_6D_6$, RT): δ [ppm] =** 7.61 (d, *J* = 8.8 Hz, 2H), 6.87 (d, *J* = 8.8 Hz, 2H), 3.60 (d*p*t, *J* = 8.0, 6.7 Hz, 1H), 3.34 (s, 3H), 2.92 (dd, *J* = 9.8, 6.6 Hz, 1H), 2.40 (dd, *J* = 9.8, 6.9 Hz, 1H), 2.22 (s, 3H), 1.69 (dqq, *J* = 8.0, 6.7, 6.6 Hz, 1H), 1.51 (s, 3H), 1.08 (d, *J* = 6.6 Hz, 3H), 0.76 (d, *J* = 6.7 Hz, 3H). **$^{13}$C-NMR (125 MHz, $C_6D_6$, RT): δ [ppm] =** 159.6, 137.9, 127.9, 113.5, 97.4, 81.1, 57.5, 54.8, 37.8, 33.7, 24.6, 19.8, 18.8. ***cis*-Diastereomer: $^1$H-NMR (500 MHz, $C_6D_6$, RT):**

**δ [ppm] =** 7.67 (d, *J* = 8.8 Hz, 2H), 6.84 (d, *J* = 8.8 Hz, 2H), 3.68 (dt, *J* = 8.9, 7.1 Hz, 1H), 3.33 (s, 3H), 2.66 (d, *J* = 7.1 Hz, 2H), 2.22 (s, 3H), 1.59 – 1.47 (m, 1H), 1.54 (s, 3H), 1.19 (d, *J* = 6.6 Hz, 3H), 0.60 (d, *J* = 6.7 Hz, 3H). **$^{13}$C-NMR (125 MHz, $C_6D_6$, RT): δ [ppm] =** 159.5, 139.3, 128.0, 113.6, 98.8, 80.8, 58.7, 54.8, 37.7, 33.8, 23.6, 20.5, 18.6. **IR:** 2956, 2870, 1609, 1508, 1464, 1366, 1299, 1242, 1221, 1171, 1089, 1034, 977, 829 791, 577 cm$^{-1}$. **HRMS (ESI+):** *m/z* calculated for $C_{15}H_{24}NO_2^+$: 250.1802 u, found: 250.1798 u.

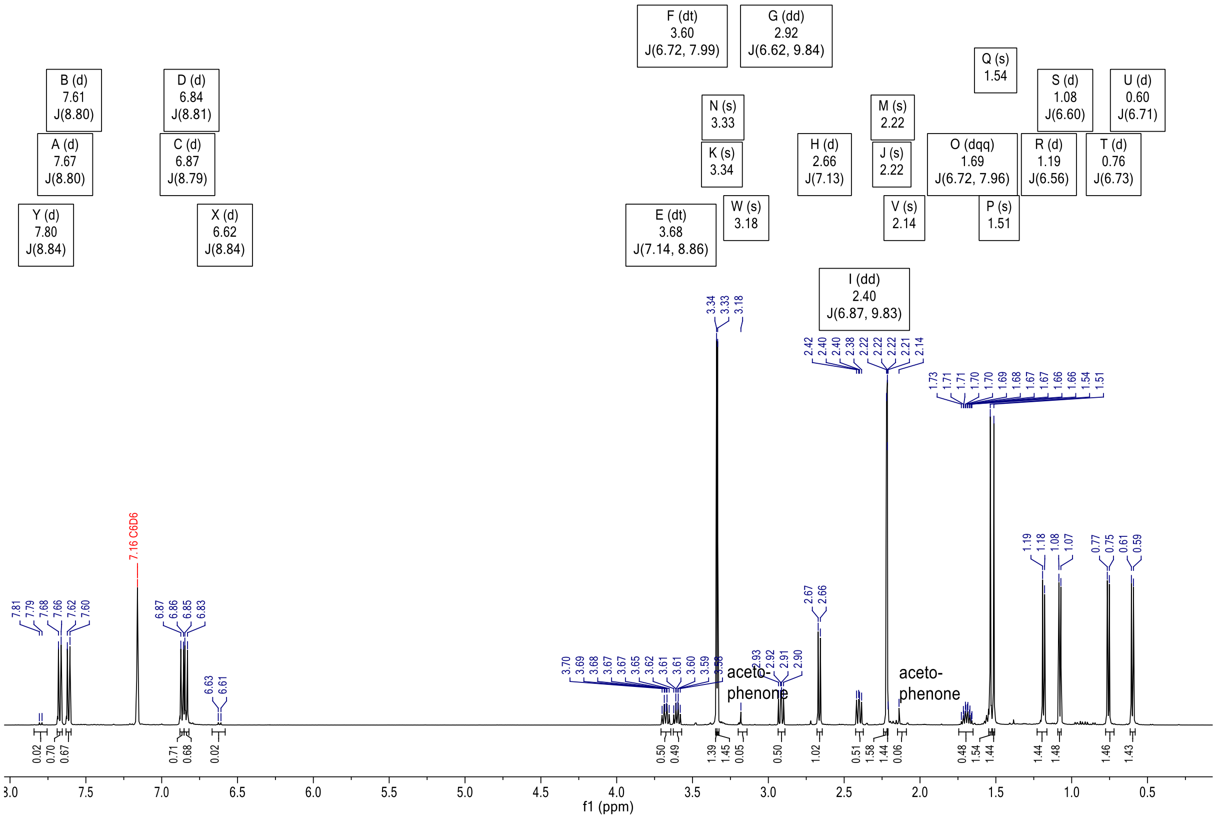

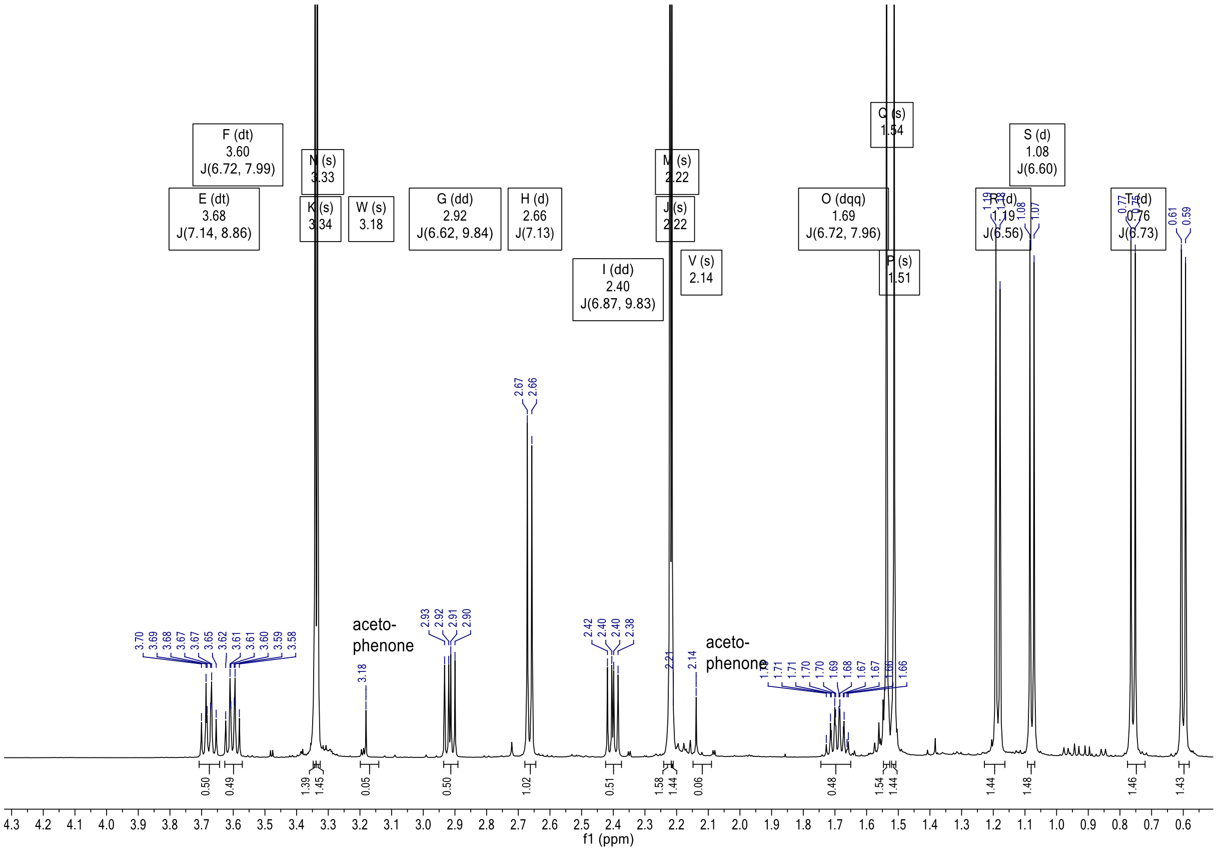

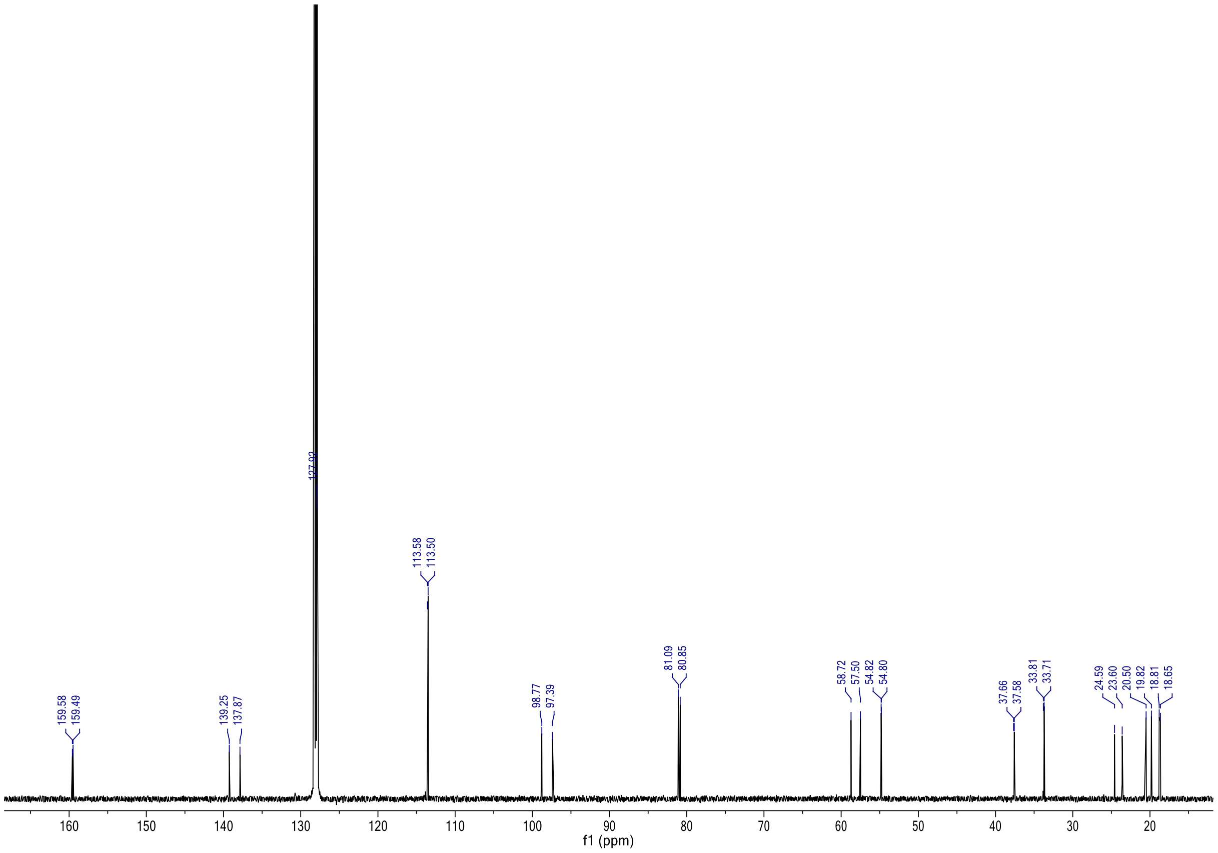

## 4.4 Catalysis with subsequent acidic workup to carbonyls **4b-2, 6e-2, 6f-2**

### 4.4.1. Synthesis of tetralon **4b-2**

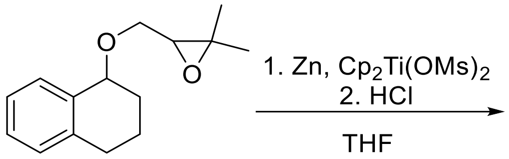


According to GP IX 32.2 mg $Cp_2Ti(OMs)_2$ (0.0874 mmol, 0.10 eq.), 11.8 mg zinc dust (0.180 mmol, 0.21 eq.) and 198.5 mg substrate **3b** (0.854 mmol, 1.00 eq.) are refluxed. Column chromatography ($SiO_2$, eluent: pentane:$Et_2O$, 96:4) afforded 87.8 mg **4b-2** (0.600 mmol, 70%) as a light yellow oil.

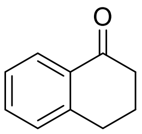

**4b-2**
$C_{10}H_{10}O$
146,19 g/mol

**$^1$H-NMR (500 MHz, $C_6D_6$, RT): δ [ppm] =** 8.30 (d, *J* = 7.7 Hz, 1H), 7.06 (*p*t, *J* = 7.3 Hz, 1H), 6.98 (*p*t, *J* = 7.4 Hz, 1H), 6.80 (d, *J* = 7.6 Hz, 1H), 2.35 – 2.29 (m, 2H), 2.29 – 2.23 (m, 2H), 1.53 – 1.44 (m, 2H). **$^{13}$C-NMR (125 MHz, $C_6D_6$, RT): δ [ppm] =** 196.5, 144.4, 133.4, 133.0, 128.8, 127.5, 126.8, 39.2, 29.7, 23.3. **IR:** 2944, 1682, 1600, 1455, 1323, 1285, 1225, 1183, 1115, 1024, 906, 896, 762, 733, 552, 486, 418 $cm^{-1}$. **HRMS (ESI+):** *m/z* calculated for $C_{10}H_{11}O^+$: 147.0804 u, found: 147.0807.

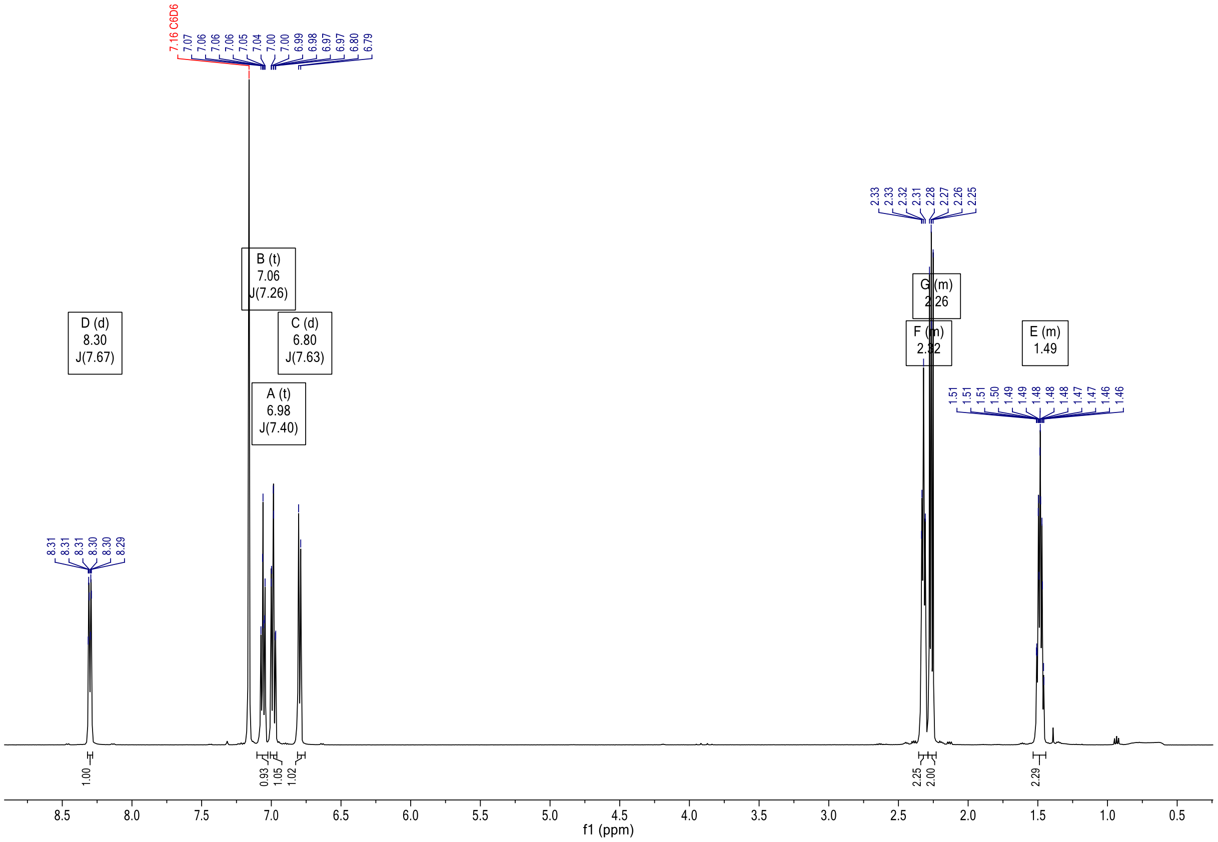

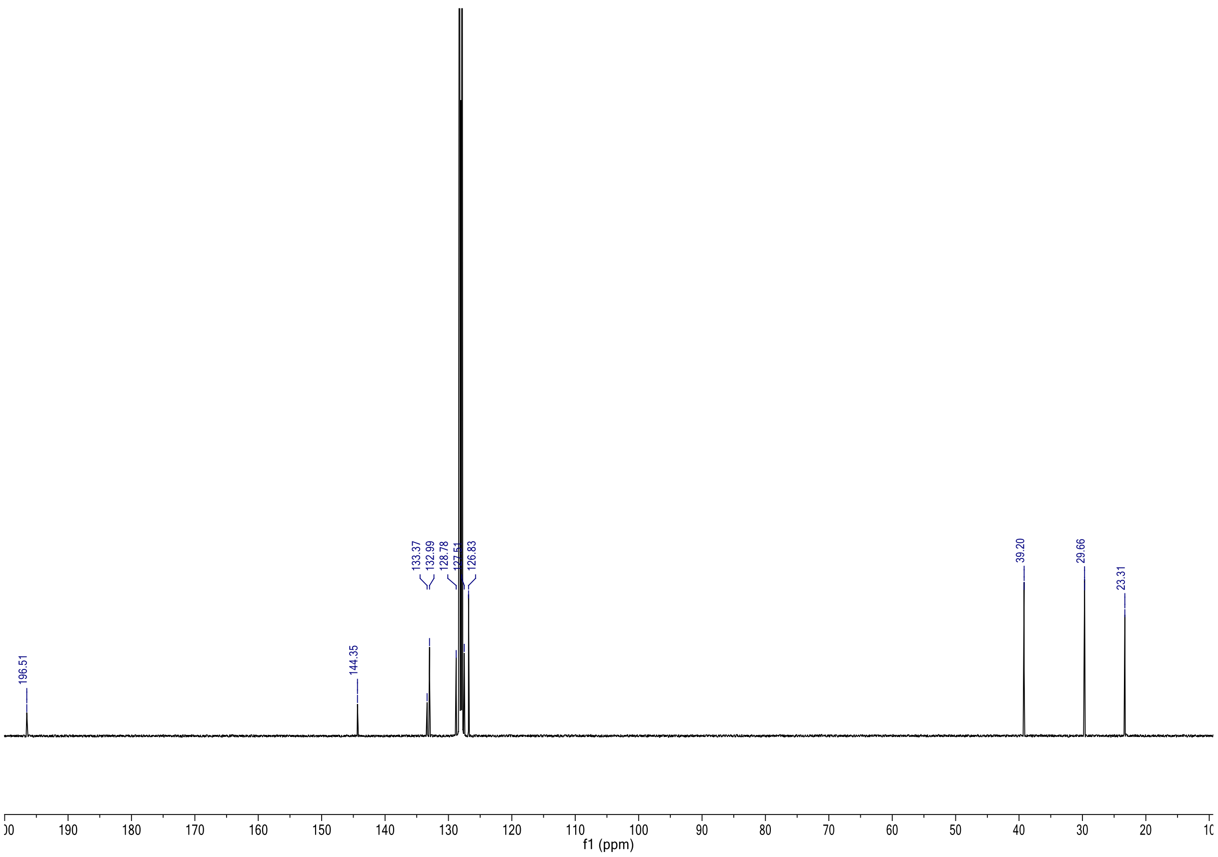


### 4.4.2 Synthesis of 3-methoxy acetophenone **6e-2**

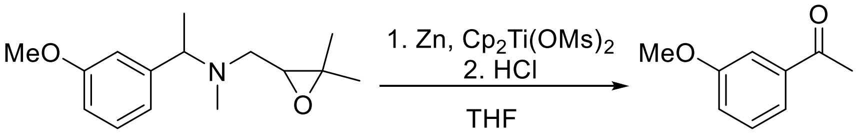


According to GP IX 15.0 mg $Cp_2Ti(OMs)_2$ (0.0407 mmol, 0.10 eq.), 5.2 mg zinc dust (0.0795 mmol, 0.20 eq.) and 100.0 mg substrate **5e** (0.401 mmol, 1.00 eq.) are refluxed. Column chromatography ($SiO_2$, eluent: pentane:$Et_2O$, 90:10) afforded 39.8 mg **6e-2** (0.265 mmol, 66%) as a light yellow oil.

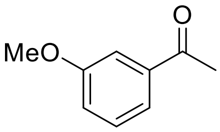


**6e-2**
$C_9H_{10}O_2$
150,18 g/mol

**$^1$H-NMR (500 MHz, $C_6D_6$, RT): δ [ppm] =** 7.59 – 7.53 (m, 1H), 7.33 – 7.28 (m, 1H), 7.03 – 6.96 (m, 1H), 6.90 – 6.84 (m, 1H), 3.27 (s, 3H), 2.13 (dd, *J* = 3.6, 1.6 Hz, 3H). **$^{13}$C-NMR (125 MHz, $C_6D_6$, RT): δ [ppm] =** 196.4, 160.3, 139.1, 129.7, 121.3, 119.6, 112.5, 54.8, 26.3. **IR:** 1681, 1593, 1583, 1485, 1428, 1356, 1272, 1221, 1180, 1075, 1036, 874, 860, 784, 686, 601, 588, 563 $cm^{-1}$. **HRMS (ESI+):** *m/z* calculated for $C_9H_{11}O_2^+$: 151.0754 u, found: 151.0754 u.

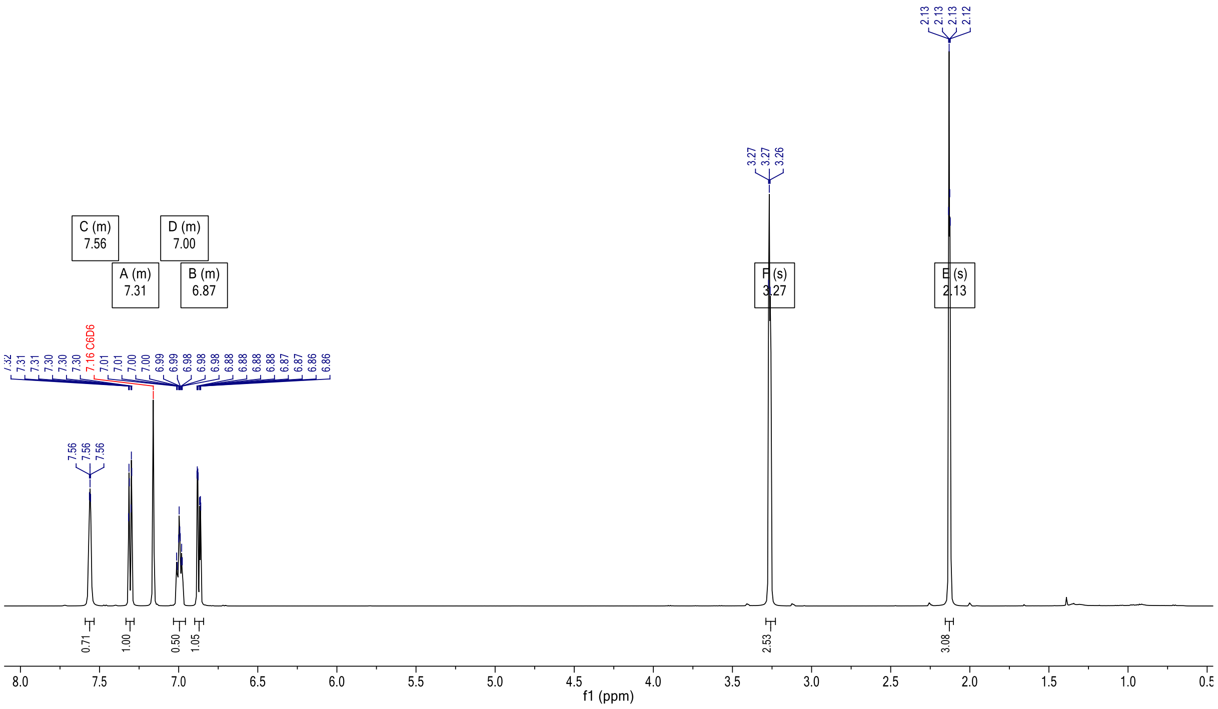
C (m)
7.56
D (m)
7.00
A (m)
7.31
B (m)
6.87
F (s)
3.27
E (s)
2.13
7.16 C6D6
0.71
1.00
0.50
1.05
2.53
3.08
f1 (ppm)

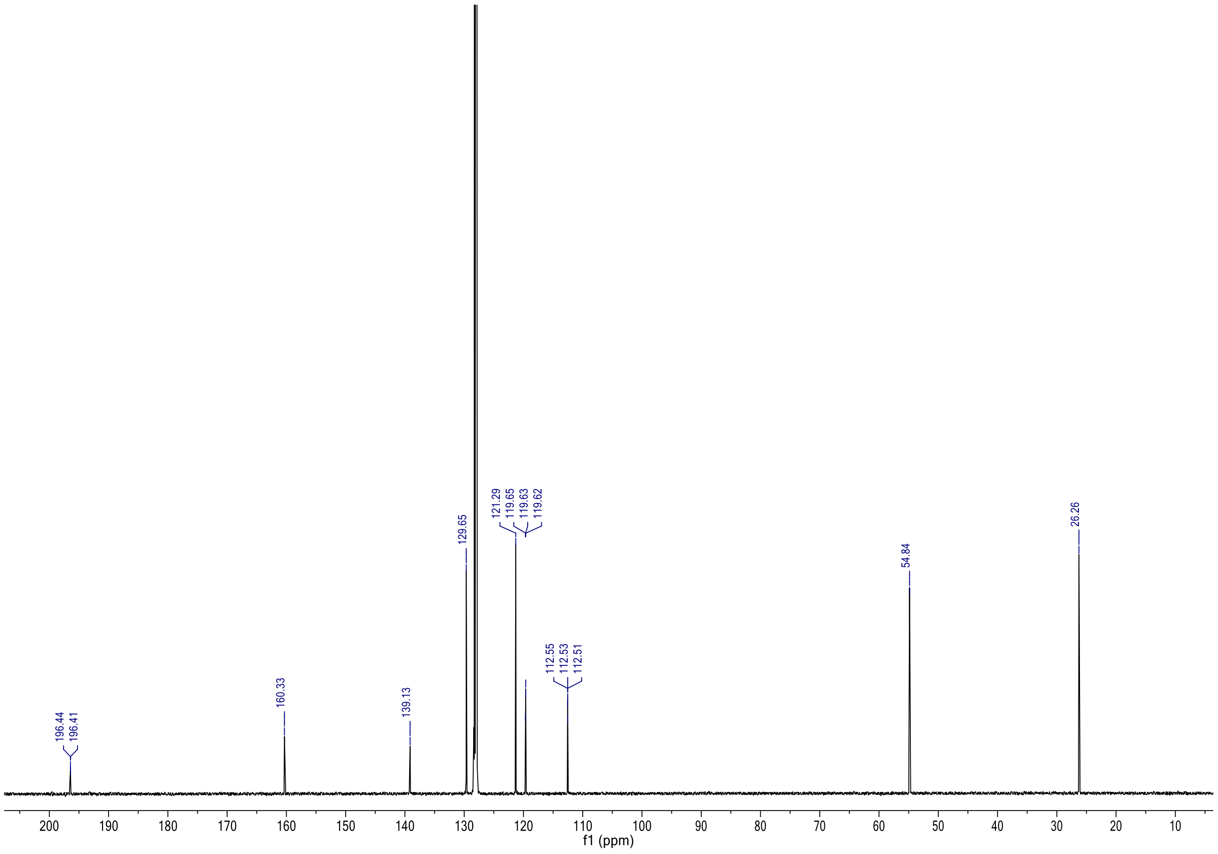
196.44
196.41
160.33
139.13
129.65
121.29
119.65
119.63
119.62
112.55
112.53
112.51
54.84
26.26
f1 (ppm)

### 4.4.3 Synthesis of 4-methoxy acetophenone **6f-2**

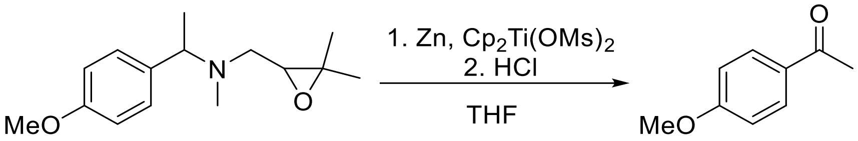


According to GP IX 29.8 mg $Cp_2Ti(OMs)_2$ (0.0809 mmol, 0.10 eq.), 10.5 mg zinc dust (0.161 mmol, 0.20 eq.) and 200.0 mg substrate **5f** (0.802 mmol, 1.00 eq.) are refluxed. Column chromatography ($SiO_2$, eluent: pentane:$Et_2O$, 90:10) afforded 84.8 mg **6f-2** (0.564 mmol, 70%) as a light yellow oil.

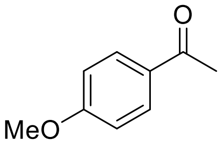


**6f-2**
$C_9H_{10}O_2$
150,18 g/mol

**$^1$H-NMR (500 MHz, $C_6D_6$, RT): δ [ppm] =** 7.80 (d, *J* = 8.9 Hz, 2H), 6.62 (d, *J* = 8.9 Hz, 2H), 3.19 (s, 3H), 2.14 (s, 3H). **$^{13}$C-NMR (125 MHz, $C_6D_6$, RT): δ [ppm] =** 195.1, 163.5, 131.0, 130.7, 113.8, 54.9, 26.0. **IR:** 1666, 1597, 1573, 1505, 1416, 1356, 1275, 1245, 1170, 1111, 1074, 1019, 953, 831, 574, 498, 462 $cm^{-1}$. **HRMS (ESI+):** *m/z* calculated for $C_9H_{11}O_2^+$: 151.0754 u, found: 151.0754 u.

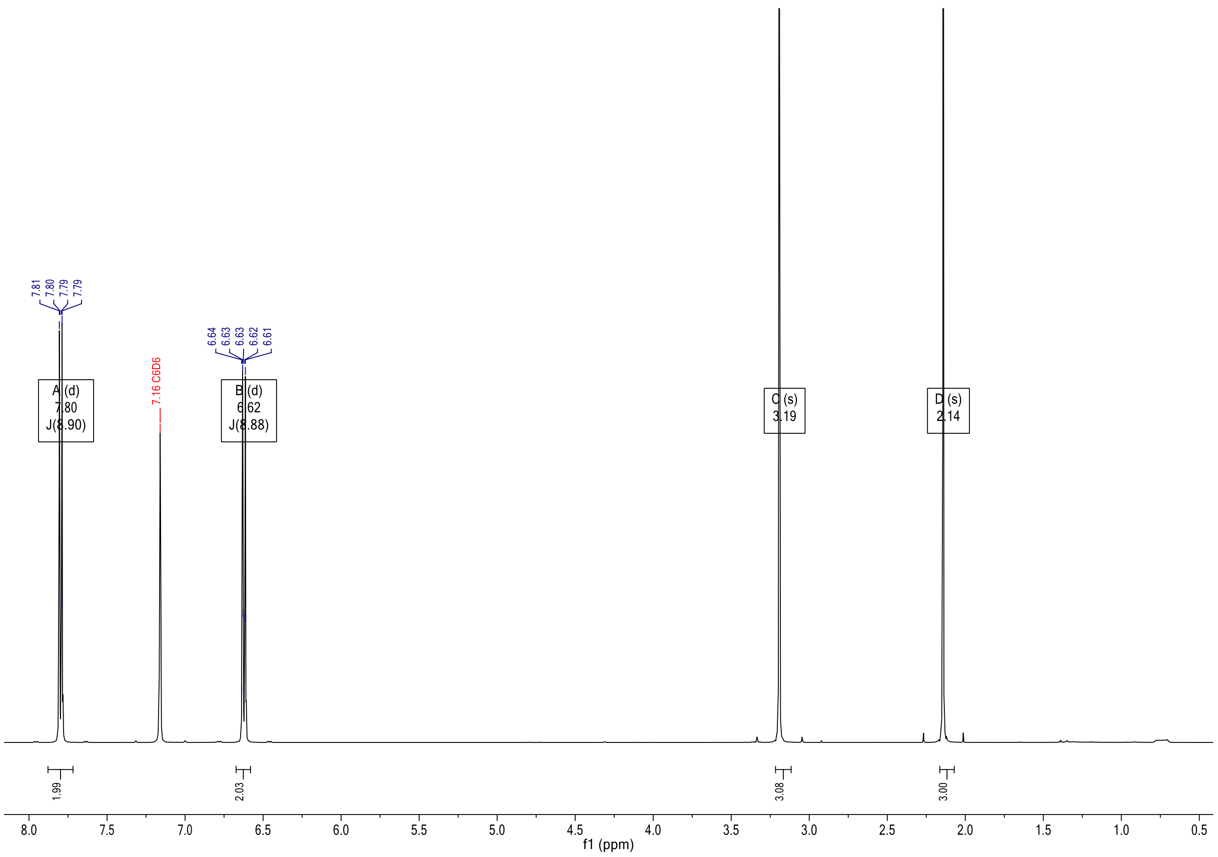
7.81
7.80
7.79
7.79
7.16 C6D6
6.64
6.63
6.63
6.62
6.61
A (d)
7.80
J(8.90)
B (d)
6.62
J(8.88)
C (s)
3.19
D (s)
2.14
1.99
2.03
3.08
3.00
8.0
7.5
7.0
6.5
6.0
5.5
5.0
4.5
4.0
3.5
3.0
2.5
2.0
1.5
1.0
0.5
f1 (ppm)

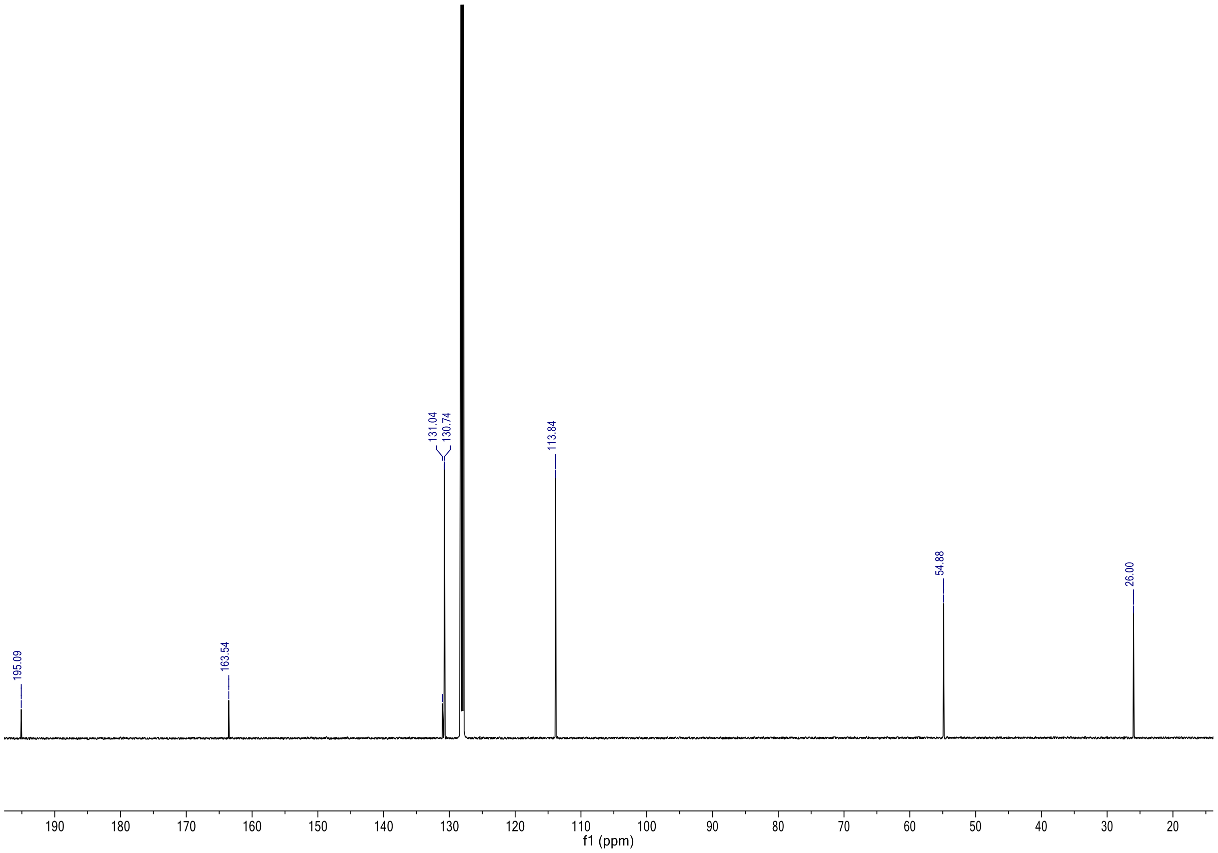
195.09
163.54
131.04
130.74
113.84
54.88
26.00
190
180
170
160
150
140
130
120
110
100
90
80
70
60
50
40
30
20
f1 (ppm)

# 5. DFT Calculations

| **Geometrie**[a)] | **d(C-O) of forming bond [pm]** |
|---|---|
| **IIa-*cis*** (radical) | 302.123 |
| **IIa-*trans*** (radical) | 313.034 |
| ***cis*-2a** (product) | 139.873 |
| ***trans*-2a** (product) | 139.257 |
| **TS2a-*cis*** (transition-state) | 241.568 |
| **TS2a-*trans*** (transition-state) | 284.959 |
| | |
| **IVc-*cis*** (radical) | 400.208 |
| **IVc-*trans*** (radical) | 331.991 |
| ***cis*-4c** (product) | 140.743 |
| ***trans*-4c** (product) | 140.224 |
| | |
| **VIa-*cis*** (radical) | 327.039 |
| **VIa-*trans*** (radical) | 267.178 |
| ***cis*-6a** (product) | 140.622 |
| ***trans*-6a** (product) | 140.560 |

a) Extracted from PBEh-3c+DCOSMO-RS(THF) optimized geometries

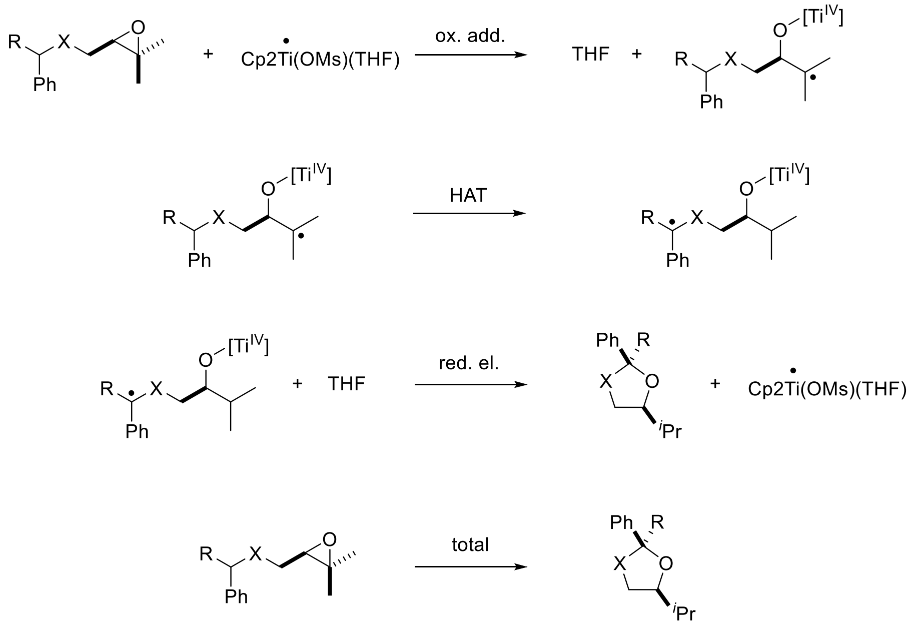


*Figure 1: Reactions.*

**Reaction energies (Acetal 2a with $Cp_2$TiOMs cat.):**

| Reaction | ΔG(PW6B95-D4/def2-QZVP)[a)] | ΔG(PW6B95-D3/def2-QZVP)[b)] | ΔH(PW6B95-D4/def2-QZVP)[a)] |
|---|---|---|---|
| oxidative addition | 13.9 | 14.0 | 10.5 |
| H-atom transfer | -15.2 | -15.3 | -18.0 |
| reductive elimination → ***cis*-2a** product | -17.8 | -17.4 | -13.6 |
| barrier reductive elimination *cis* | 16.7 | 16.3 | 15.5 |
| reductive elimination →***trans*-2a** | -18.1 | -17.7 | -13.4 |
| barrier reductive elimination *trans* | 24.8 | 24.5 | 23.6 |
| estimated barrier of **IIa**-***cis***/ **IIa**-***trans*** interconversion | 7.1 | 7.1 | 5.8 |
| total reaction (cis) | -19.1 | -18.7 | -21.1 |
| total reaction (trans) | -19.4 | -19.0 | -20.9 |

All free energies and enthalpies in kcal $mol^{-1}$. a) PW6B95-D4/def2-QZVP + COSMO-RS(THF) // PBEh-3c+DCOSMO-RS(THF), b) PW6B95-D3/def2-QZVP + COSMO-RS(THF) // PBEh-3c+DCOSMO-RS(THF).

**Reaction energies (Acetal 2a with $Cp_2$TiCl cat.):**

| Reaction | ΔG(PW6B95-D4/def2-QZVP)[a)] | ΔG(PW6B95-D3/def2-QZVP)[b)] | ΔH(PW6B95-D4/def2-QZVP)[a)] |
|---|---|---|---|
| Oxidative addition | 9.7 | 9.6 | 4.6 |
| H-atom transfer | -14.3 | -14.2 | -15.0 |
| Reductive elimination → ***cis*-2a** product | -14.5 | -14.1 | -10.7 |
| Reductive elimination → ***trans*-2a** product | -14.8 | -14.4 | -10.5 |
| Total reaction (cis) | -19.1 | -18.7 | -21.1 |
| Total reaction (trans) | -19.4 | -19.0 | -20.9 |

All free energies and enthalpies in kcal $mol^{-1}$. a) PW6B95-D4/def2-QZVP + COSMO-RS(THF) // PBEh-3c+DCOSMO-RS(THF), b) PW6B95-D3/def2-QZVP + COSMO-RS(THF) // PBEh-3c+DCOSMO-RS(THF).

**Reaction energies (Hemiaminal 6a with $Cp_2$TiOMs cat.):**

| Reaction | ΔG(PW6B95-D4/def2-QZVP)[a)] | ΔG(PW6B95-D3/def2-QZVP)[b)] | ΔH(PW6B95-D4/def2-QZVP)[a)] |
|---|---|---|---|
| Oxidative addition | 14.8 | 14.6 | 7.2 |
| H-atom transfer | -8.4 | -8.7 | -4.3 |
| Reductive elimination → cis product | -23.7 | -22.7 | -21.8 |
| Reductive elimination →trans product | -24.3 | -23.4 | -21.9 |
| Total reaction (cis) | -17.3 | -16.8 | -18.9 |
| Total reaction (trans) | -17.9 | -17.5 | -19.0 |

All free energies and enthalpies in kcal $mol^{-1}$. a) PW6B95-D4/def2-QZVP + COSMO-RS(THF) // PBEh-3c+COSMO(THF), b) PW6B95-D3/def2-QZVP + COSMO-RS(THF) // PBEh-3c+COSMO(THF).

**Cis/trans radical isomer stability:**

| ΔX = X(Cis-isomer) – X(trans-isomer) | ΔG(PW6B95-D4/def2-QZVP)[a)] | ΔG(PW6B95-D3/def2-QZVP)[b)] | ΔH(PW6B95-D4/def2-QZVP)[a)] |
|---|---|---|---|
| **IIa** (acetal) | -6.5 | -6.5 | -5.8 |
| **IVc** (ketal) | 0.6 | 0.7 | 1.1 |
| **VIa** (hemiaminal) | 0.9 | 0.9 | 0.1s |

All free energies and enthalpies in kcal $mol^{-1}$. a) PW6B95-D4/def2-QZVP + COSMO-RS(THF) // PBEh-3c+DCOSMO-RS(THF), b) PW6B95-D3/def2-QZVP + COSMO-RS(THF) // PBEh-3c+DCOSMO-RS(THF).

Isomer stability contributions to free energy (Acetal):

| | $\Delta G_{RRHO}$(PBEh-3c) | $\Delta\delta G_{solv}^{\circ}$(THF) | ΔE(PW6B95-/def2-QZVP) | ΔE(D4) | ΔG(PW6B95-D4/def2-QZVP)[a)] |
|---|---|---|---|---|---|
| (**IIa**-***cis***) – (**IIa**-***trans***) | -0.4 | -0.7 | -5.9 | 0.5 | -6.5 |

All energies in kcal $mol^{-1}$.

Stabilization energies from second-order perturbation estimates of donor-acceptor interactions in the PBEh-3c NBO basis, exemplifying the stereoelectronic gauche effect:

| Geometry | E(2) σ(C-H) → σ*(C-O) | E(2) σ(C-H) → σ*(C-O) |
|---|---|---|
| **IIa**-***cis*** (radical) | 2.8 | 3.5 |
| **IVc**-***cis*** (radical) | 2.6 | 3.0 |
| **VIa**-***cis*** (radical) | 1.7 σ(C-C) → σ*(C-N) | 1.9 |
| **TS2a**-***cis*** (transition-state) | 1.5 | 2.6 |
| **TS2a**-***trans*** (transition-state) | 1.9 | 2.8 |

All stabilization energies (E(2)) in kcal $mol^{-1}$. Since in these molecules two different gauche interactions are possible, the weaker effect is shown in the first and the stronger one in the last column.

Bond dissociation energies (BDE) of Benzylmethylether and N,N-Dimethylbenzylamine

Reaction: Dissociation of a hydrogen atom at the benzylic position

| BDE | PW6B95-D4 | B3LYP-D4 | PBE0-D4 | wB97X-D3 | PWPB95-D4 | DSD-BLYP-D3BJ |
|---|---|---|---|---|---|---|
| Benzylmethylether | 80.5 | 79.7 | 78.6 | 81.9 | 83.1 | 87.2 |
| N,N-Dimethylbenzylamine | 80.7 | 80.1 | 79.3 | 82.8 | 83.4 | 87.7 |
| Δ BDE (N,N-Dimethylbenzylamine – Benzylmethylether) | 0.2 | 0.4 | 0.7 | 0.9 | 0.3 | 0.5 |

All energies in kcal mol$^{-1}$. The basis set applied is def2-QZVP. Calculation on PBEh-3c/DCOSMO-RS(THF) geometries, thermostatistical contributions are calculated with PBEh-3c/COSMO @ 339.15 K.

Reaction-path for estimated barrier of **IIa** *cis/trans* rotation:

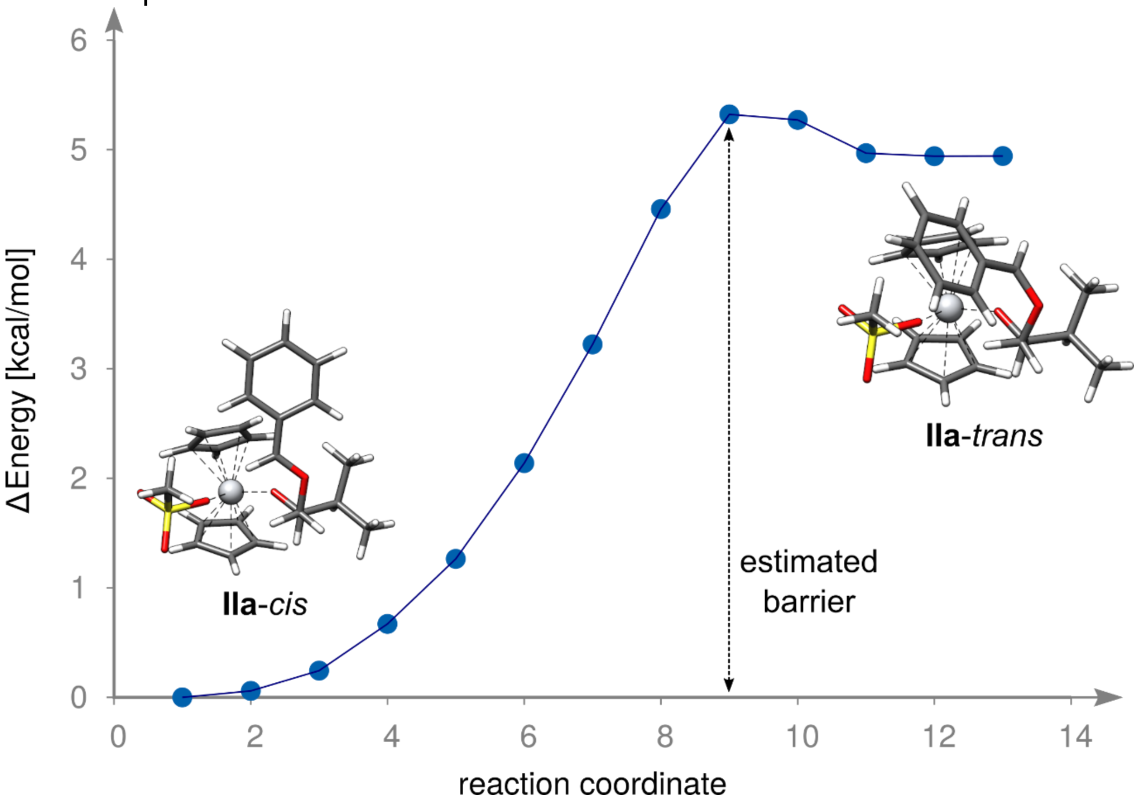


*Figure 2: PBEh-3c+COSMO(THF) reaction path of IIa-cis/trans rotation.*

Visualization of geometries:

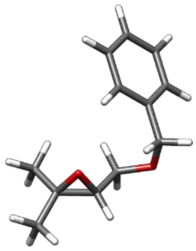

**1a**

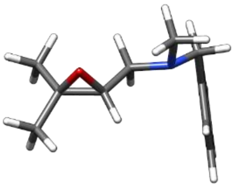

**5a**

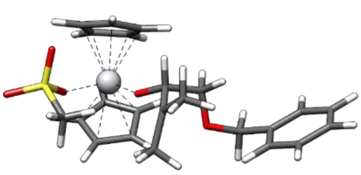

**Ia Cp2TiOMs**

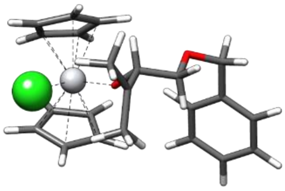

**Ia Cp2TiCl**

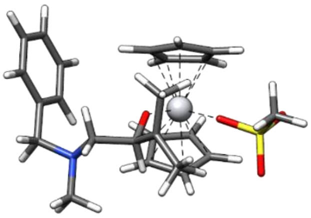

**Va Cp2TiOMs**

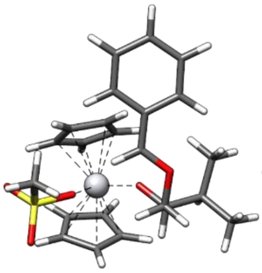

**IIa-*cis***

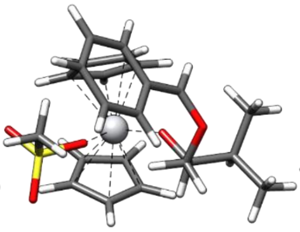

**IIa-*trans***

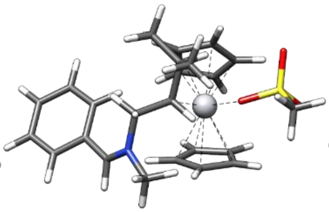

**VIa-*cis***

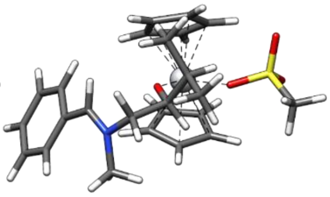

**VIa-*trans***

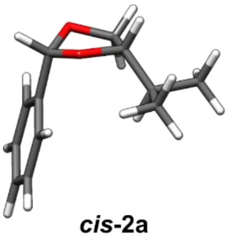

***cis*-2a**

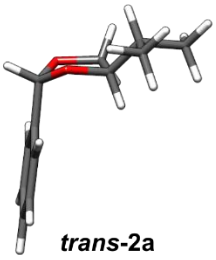

***trans*-2a**

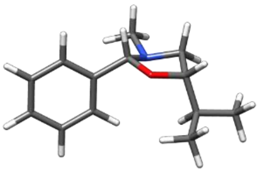

***cis*-6a**

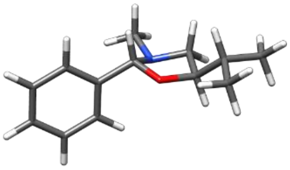

***trans*-6a**

*Figure 3: PBEh-3c+DCOSMO-RS(THF) optimized geometries.*

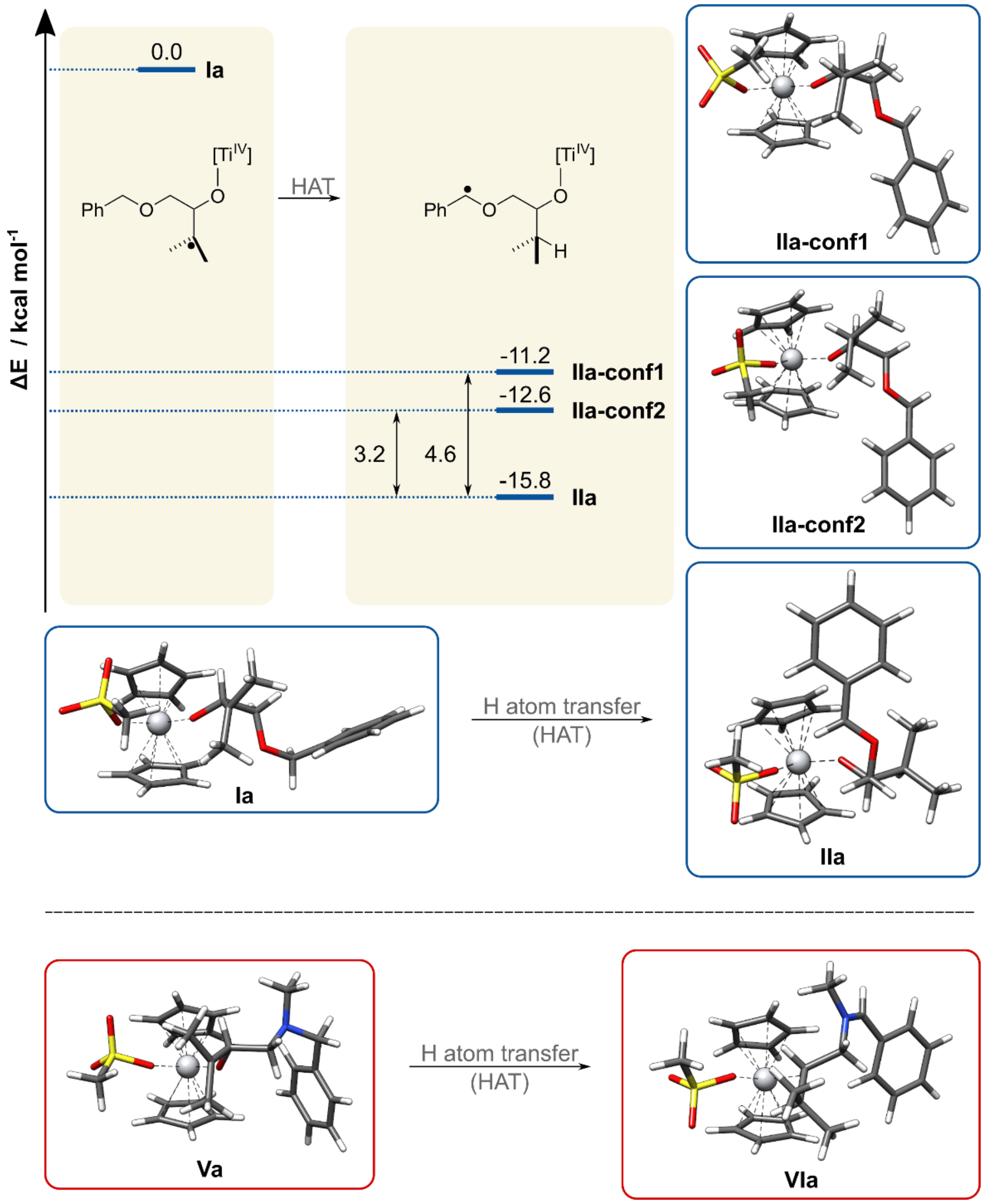


*Figure 4: Influence of different conformations search on the evaluation of the thermodynamic cycle (see scheme 2 and 4). All energies are evaluated at the PBEh-3c/DCOSMO-RS(THF) level and are given in kcal/mol.*

Applying conformational searches in the elucidation of thermodynamic cycles is essential for identifying lowest energy conformations at the correct position in the catalytic cycle. The necessity can be seen when focusing on the H atom transfer starting from **Ia** or **Va**. The HAT is an exergonic reaction forming a benzylic radical originating from a tertiary radical. If the computational chemist only investigates the hydrogen atom transfer and does not account for possible conformational relaxation the evaluated conformation is less stable by 3.2 to 4.6 kcal mol$^{-1}$. This is based on the energetic (PBEh-3c/DCOSMO-RS(THF)) comparison of structurally similar conformations between **Ia** and **IIa** (e.g. only little relaxation, meaning a simple optimization and no conformational search) with the lowest lying conformation of the benzylic radical **IIa**). The same evaluation for the hemiaminal for comparing structurally close conformations between **Va** and **VIa** against the lowest lying conformation of **VIa** shows only a small difference in stability of 0.3 kcal mol$^{-1}$. This highlights the strongly

deviating structural flexibilities even for similar systems (comparing acetal and hemiaminal) and can only be accounted for by applying robust and efficient conformational searches (for the energetic sorting of the conformer ensembles see SI figure 5). If the conformational relaxation is not accounted for e.g. for **IIa** the barrier heights of the following reductive elimination reaction would be evaluated from a false starting point. This would wrongfully shift the barrier heights and might result in false statements being made.

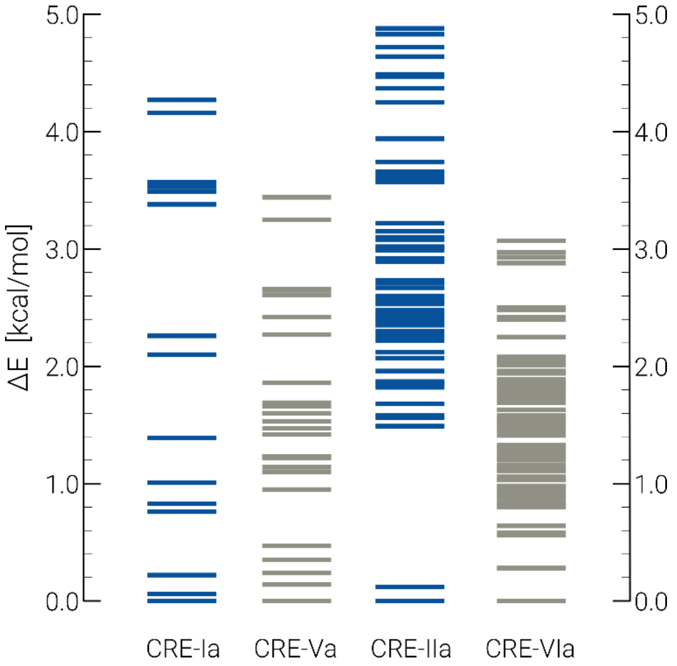


*Figure 5: Comparison of the conformer rotamer ensembles of **Ia** and **IIa** as well as **Va** and **VIa** needed for the HAT reaction. All energies are evaluated at the PBEh-3c/DCOSMO-RS(THF) level of theory.*

In SI figure 5 the PBEh-3c/DCOSMO-RS(THF) ranked conformer ensembles are shown for the acetal in blue and the hemiaminal in grey. The conformer ensemble of the aminals (**Va** and **VIa**) are denser compared to the acetal (**Ia** and **IIa**) and by visual inspection the ensembles of the aminal are structurally similar, whereas the acetal seems to be more flexible and structurally different when comparing the tertiary and benzylic radical **Ia** and **IIa**.

Geometries:

30

Name = 1a

| | | | |
|---|---|---|---|
| H | -0.6893723 | -1.0560848 | 1.5050750 |
| C | -1.5226535 | -0.7929676 | 0.8652112 |
| C | -2.4214778 | -1.7731210 | 0.4622317 |
| C | -3.4779734 | -1.4448606 | -0.3744955 |
| C | -3.6326503 | -0.1326330 | -0.8067431 |
| C | -2.7355236 | 0.8426078 | -0.3994391 |
| C | -1.6755139 | 0.5244859 | 0.4475801 |
| C | -0.6995671 | 1.5927918 | 0.8666360 |
| H | -1.2400795 | 2.4608927 | 1.2494807 |
| O | 0.0771310 | 2.0804164 | -0.2100545 |
| C | 0.9289790 | 1.1300471 | -0.8069558 |
| C | 2.0977984 | 0.7591540 | 0.0652268 |
| C | 2.8896962 | -0.4613120 | -0.1108508 |
| C | 2.5500341 | -1.4918836 | -1.1580498 |
| H | 1.4820536 | -1.5580263 | -1.3567567 |
| H | 2.8888018 | -2.4787712 | -0.8370731 |
| H | 3.0567251 | -1.2605724 | -2.0956766 |
| C | 4.3432221 | -0.4445241 | 0.2882849 |
| H | 4.9707641 | -0.2102578 | -0.5726296 |
| H | 4.6516786 | -1.4212863 | 0.6658368 |
| H | 4.5417425 | 0.2977454 | 1.0615226 |
| O | 1.9681712 | -0.3362814 | 0.9658592 |
| H | 2.6115732 | 1.6240905 | 0.4889523 |
| H | 1.3160015 | 1.6029902 | -1.7129306 |
| H | 0.3741969 | 0.2384608 | -1.1177003 |

| | | | |
|---|---|---|---|
| H | -0.0714018 | 1.2228528 | 1.6843823 |
| H | -2.8648426 | 1.8640160 | -0.7380067 |
| H | -4.4558319 | 0.1291836 | -1.4590850 |
| H | -4.1792124 | -2.2065069 | -0.6900782 |
| H | -2.2912894 | -2.7940945 | 0.7971711 |

34

Name = 5a

| | | | |
|---|---|---|---|
| H | 2.9263348 | 0.4818751 | -2.0862058 |
| C | 2.9853708 | 0.0359156 | -1.0999586 |
| C | 3.8536859 | -1.0262894 | -0.8771193 |
| C | 3.9265381 | -1.6120851 | 0.3780368 |
| C | 3.1274023 | -1.1279362 | 1.4075396 |
| C | 2.2654629 | -0.0653938 | 1.1822898 |
| C | 2.1868772 | 0.5306834 | -0.0750376 |
| C | 1.2711870 | 1.7051404 | -0.3072910 |
| N | -0.0411735 | 1.5127184 | 0.2773289 |
| C | -0.7894040 | 2.7517936 | 0.3178957 |
| H | -1.0319557 | 3.1457935 | -0.6811174 |
| H | -1.7267104 | 2.6192729 | 0.8564280 |
| H | -0.2142328 | 3.5127454 | 0.8466895 |
| C | -0.7763383 | 0.4526153 | -0.3863499 |
| C | -1.9454007 | -0.0185693 | 0.4348136 |
| C | -3.0420102 | 0.8351020 | -0.0885773 |
| C | -3.1226966 | -1.2225204 | -1.5433767 |
| H | -4.1647120 | -1.3458668 | -1.8445929 |
| H | -2.6708249 | -0.4842300 | -2.2030086 |
| H | -2.6194409 | -2.1760895 | -1.7083083 |
| C | -3.7493481 | -1.7864586 | 0.8426892 |
| H | -3.3251389 | -2.7877314 | 0.7581826 |
| H | -4.8099507 | -1.8553439 | 0.5933449 |
| H | -3.6644422 | -1.4719527 | 1.8828917 |
| O | -3.2315015 | 0.5451395 | 0.2052627 |
| H | -1.6930469 | -0.1453325 | 1.4892037 |
| H | -0.1061699 | -0.4020520 | -0.5128177 |
| H | -1.1015419 | 0.7527657 | -1.3952905 |
| H | 1.2227808 | 1.9233891 | -1.3878612 |
| H | 1.7136891 | 2.5916168 | 0.1577090 |
| H | 1.6351880 | 0.3019365 | 1.9824936 |
| H | 3.1765422 | -1.5819142 | 2.3891741 |
| H | 4.5973155 | -2.4431242 | 0.5538235 |
| H | 4.4662368 | -1.3999664 | -1.6876604 |

30

Name = *cis*-2a

| | | | |
|---|---|---|---|
| H | -1.9903974 | -1.9500789 | 0.8619570 |
| C | -2.1140216 | -0.9361818 | 0.5033980 |
| C | -3.1769658 | -0.1731688 | 0.9673547 |
| C | -3.3495364 | 1.1295243 | 0.5198866 |
| C | -2.4527123 | 1.6633738 | -0.3953040 |
| C | -1.3879841 | 0.9012697 | -0.8561908 |
| C | -1.2065478 | -0.4029773 | -0.4071004 |
| C | -0.0710862 | -1.2570839 | -0.9459589 |
| O | 0.3855946 | -2.1814088 | -0.0055262 |
| C | 1.3124992 | -1.4687036 | 0.7888680 |
| H | 0.8109407 | -0.9649066 | 1.6250680 |
| H | 2.0390224 | -2.1708364 | 1.1959428 |
| C | 1.9282060 | -0.4496071 | -0.1859019 |
| C | 2.0782779 | 0.9475378 | 0.4042994 |
| C | 3.0715623 | 0.9022697 | 1.5630135 |
| H | 4.0577409 | 0.5783428 | 1.2218028 |
| H | 2.7559183 | 0.2231143 | 2.3571918 |
| H | 3.1867059 | 1.8897570 | 2.0107892 |
| C | 2.5246388 | 1.9573658 | -0.6459279 |
| H | 3.4881294 | 1.6761650 | -1.0778291 |
| H | 1.8059608 | 2.0434315 | -1.4602762 |
| H | 2.6402674 | 2.9469917 | -0.2018023 |
| H | 1.1021947 | 1.2579578 | 0.7952650 |
| H | 2.9110305 | -0.7898459 | -0.5358683 |
| O | 1.0432613 | -0.4901050 | -1.3015231 |
| H | -0.4087248 | -1.8100923 | -1.8291333 |
| H | -0.6938656 | 1.3316113 | -1.5662802 |
| H | -2.5794277 | 2.6780766 | -0.7503169 |
| H | -4.1779898 | 1.7247033 | 0.8815738 |
| H | -3.8708522 | -0.5977631 | 1.6814675 |

33

Name = *cis*-4c

| | | | |
|---|---|---|---|
| H | -0.8216277 | -2.0336750 | -1.2431706 |
| C | 0.2227975 | -1.9729430 | -0.9635350 |
| C | 0.8959904 | -3.1298070 | -0.5998452 |
| C | 2.2353136 | -3.0729476 | -0.2370887 |
| C | 2.8921487 | -1.8509903 | -0.2418092 |
| C | 2.2159629 | -0.6920723 | -0.6020321 |
| C | 0.8740784 | -0.7424706 | -0.9627527 |
| C | 0.1229610 | 0.5028608 | -1.4118135 |
| O | 0.7753238 | 1.6494455 | -0.9204668 |
| C | -0.1503548 | 2.4330586 | -0.1972335 |
| C | -1.2220638 | 1.4287110 | 0.2108697 |
| C | -0.9872393 | 0.7592195 | 1.5667285 |
| C | -1.8987917 | -0.4486575 | 1.7514075 |
| H | -1.7611168 | -0.8815279 | 2.7439540 |
| H | -2.9508911 | -0.1647042 | 1.6600257 |
| H | -1.6953847 | -1.2303545 | 1.0208059 |
| C | -1.2206911 | 1.7705123 | 2.6866906 |
| H | -1.0440959 | 1.3128958 | 3.6604530 |
| H | -2.2513162 | 2.1338409 | 2.6763182 |
| H | -0.5635311 | 2.6384175 | 2.6152927 |
| O | -1.1809590 | 0.5274909 | -0.8826124 |
| H | -2.2174486 | 1.8863159 | 0.2138152 |
| H | -0.5702592 | 3.2283837 | -0.8238260 |
| H | 0.3635228 | 2.8993516 | 0.6444378 |

| | | | |
|---|---|---|---|
| H | 0.0541436 | 0.4197902 | 1.6178461 |
| C | 0.0321694 | 0.5494341 | -2.9304669 |
| H | 2.7440237 | 0.2518056 | -0.5969473 |
| H | 3.9362870 | -1.7961659 | 0.0387113 |
| H | 2.7623590 | -3.9746803 | 0.0467977 |
| H | 0.3731913 | -4.0776408 | -0.5984583 |
| H | 1.0293112 | 0.5448519 | -3.3703950 |
| H | -0.4892577 | 1.4506320 | -3.2575157 |
| H | -0.5045558 | -0.3183812 | -3.3141852 |

34

Name = cis-6a

| | | | |
|---|---|---|---|
| H | -2.6743835 | -0.6538864 | -2.0410591 |
| C | -2.6531228 | -0.0982359 | -1.1105151 |
| C | -3.8087573 | 0.5256339 | -0.6527960 |
| C | -3.7832921 | 1.2401462 | 0.5346725 |
| C | -2.6012808 | 1.3269524 | 1.2639110 |
| C | -1.4529644 | 0.7026875 | 0.8056391 |
| C | -1.4719284 | -0.0145112 | -0.3878331 |
| C | -0.2183549 | -0.6793084 | -0.8909614 |
| N | 0.3851692 | -1.5397983 | 0.0975684 |
| C | 1.7705243 | -1.6187944 | -0.3075104 |
| C | 2.0409324 | -0.1749766 | -0.7230406 |
| O | 0.7689490 | 0.2803713 | -1.1768025 |
| H | 2.7434698 | -0.1294943 | -1.5646157 |
| C | 2.5727221 | 0.7069341 | 0.4080487 |
| C | 2.4503373 | 2.1893052 | 0.0714368 |
| H | 3.0055141 | 2.4334638 | -0.8380546 |
| H | 2.8612684 | 2.7988084 | 0.8778546 |
| H | 1.4144852 | 2.4899964 | -0.0768477 |
| C | 4.0274158 | 0.3541588 | 0.7066376 |
| H | 4.1574396 | -0.6940768 | 0.9798725 |
| H | 4.4079970 | 0.9531383 | 1.5347294 |
| H | 4.6629138 | 0.5512044 | -0.1602473 |
| H | 1.9740994 | 0.5022426 | 1.3038760 |
| H | 1.9256122 | -2.3073087 | -1.1545238 |
| H | 2.4008618 | -1.9602182 | 0.5153977 |
| C | -0.2800262 | -2.8064248 | 0.2662904 |
| H | 0.2126648 | -3.3825223 | 1.0492327 |
| H | -0.2869496 | -3.4166074 | -0.6497787 |
| H | -1.3141122 | -2.6491527 | 0.5747358 |
| H | -0.4606947 | -1.2402597 | -1.8181863 |
| H | -0.5364337 | 0.7649926 | 1.3787107 |
| H | -2.5789634 | 1.8835536 | 2.1921331 |
| H | -4.6795483 | 1.7287527 | 0.8946384 |
| H | -4.7245643 | 0.4534526 | -1.2249889 |

35

Name = Cp2TiCl-THF

| | | | |
|---|---|---|---|
| C | -1.8305739 | 0.8710968 | -1.5332885 |
| C | -0.8304117 | 1.7510853 | -1.0802311 |
| C | 0.2667682 | 1.6538336 | -1.9505229 |
| H | -0.8874093 | 2.3755981 | -0.2007261 |
| C | -0.0753655 | 0.7360147 | -2.9744674 |
| C | -1.3662708 | 0.2532169 | -2.7165188 |
| H | -1.9005409 | -0.4809091 | -3.2995827 |
| H | -2.7832634 | 0.6917547 | -1.0596890 |
| H | 1.1966867 | 2.1964492 | -1.8659525 |
| H | 0.5497340 | 0.4541236 | -3.8083495 |
| C | 2.3234543 | -0.6583275 | -0.3528618 |
| C | 2.2495479 | -0.7012856 | -1.7561018 |
| C | 1.5841552 | -1.8913312 | -2.1285396 |
| H | 2.6403518 | 0.0446647 | -2.4309035 |
| C | 1.2825194 | -2.5989529 | -0.9507718 |
| C | 1.7200116 | -1.8371480 | 0.1438814 |
| H | 1.6049362 | -2.1056270 | 1.1834517 |
| H | 2.7883767 | 0.1214621 | 0.2319810 |
| H | 1.3669863 | -2.2121409 | -3.1367322 |
| H | 0.7525264 | -3.5355231 | -0.8954179 |
| Ti | 0.0175583 | -0.5216886 | -0.9451344 |
| C | -1.3373399 | 0.3556278 | 3.2822288 |
| C | -1.5656683 | -0.2935940 | 1.9278745 |
| O | -0.3633580 | -0.0368251 | 1.1734962 |
| C | 0.4975163 | 0.8573702 | 1.8928625 |
| C | -0.3833819 | 1.4951756 | 2.9484497 |
| H | -0.8672092 | -0.3453062 | 3.9749757 |
| H | -2.2712549 | 0.6917986 | 3.7308345 |
| H | -2.4100624 | 0.1538607 | 1.3994235 |
| H | -1.7255022 | -1.3674533 | 1.9769532 |
| H | 0.9290144 | 1.5764465 | 1.1957739 |
| H | 1.3130128 | 0.2855312 | 2.3455122 |
| H | -0.9262573 | 2.3488643 | 2.5361588 |
| H | 0.1932652 | 1.8447996 | 3.8037677 |
| Cl | -1.7525519 | -2.1726618 | -0.6618341 |

42

Name = Cp2TiOMs-THF

| | | | |
|---|---|---|---|
| C | -1.2728567 | 1.4544003 | -1.2619384 |
| C | -0.2158441 | 2.2948497 | -0.8590081 |
| C | 0.8353638 | 2.1440925 | -1.7757409 |
| H | -0.2103413 | 2.9338948 | 0.0117762 |
| C | 0.4157024 | 1.2236268 | -2.7673437 |
| C | -0.8854522 | 0.8044018 | -2.4528411 |
| H | -1.4641938 | 0.0826599 | -3.0077530 |
| H | -2.2140610 | 1.3289545 | -0.7476052 |
| H | 1.7897926 | 2.6479341 | -1.7370530 |
| H | 0.9921302 | 0.9017380 | -3.6215410 |
| C | 2.8689139 | -0.1049063 | -0.2039443 |
| C | 2.7338716 | -0.2595283 | -1.5971949 |
| C | 2.0514760 | -1.4702292 | -1.8461938 |
| H | 3.0956938 | 0.4287431 | -2.3453565 |
| C | 1.8052323 | -2.0838198 | -0.6029153 |

C 2.2968381 -1.2435630 0.4070047
H 2.2349357 -1.4346925 1.4680106
H 3.3558017 0.7177922 0.2990984
H 1.7789413 -1.8641138 -2.8134059
H 1.2918744 -3.0196290 -0.4467616
Ti 0.5457230 -0.0047433 -0.7197856
O -0.9753869 -1.3295698 -0.3460382
S -1.5334314 -2.5171321 -1.1090312
C -3.2888551 -2.2101427 -1.0788786
H -3.7933373 -3.0210304 -1.6005778
H -3.5050732 -1.2674478 -1.5773476
H -3.6344744 -2.1692816 -0.0483485
O -1.3008866 -3.7591091 -0.3731887
O -1.1094723 -2.5170140 -2.5092716
C -0.8718214 0.7818794 3.5007335
C -1.0639713 0.1429701 2.1344169
O 0.1347889 0.4524475 1.3946280
C 0.9832049 1.3234528 2.1548470
C 0.0769160 1.9362580 3.2029142
H -0.4096753 0.0788485 4.1963515
H -1.8190709 1.1027347 3.9321203
H -1.9179723 0.5692016 1.6041829
H -1.1879836 -0.9365540 2.1768673
H 1.4333857 2.0557227 1.4842838
H 1.7860080 0.7380386 2.6130958
H -0.4655098 2.7914996 2.7934074
H 0.6330766 2.2763655 4.0753260

52
Ia-Cp2TiCl
H 1.7476495 1.3824907 0.3387459
C 2.7102821 1.2063314 -0.1246696
C 3.1318339 2.0051729 -1.1772407
C 4.3604797 1.7732170 -1.7830357
C 5.1599070 0.7325595 -1.3322328
C 4.7316449 -0.0699813 -0.2821685
C 3.5081491 0.1625072 0.3354787
C 3.0674694 -0.6721681 1.5133479
O 1.6806248 -0.8735618 1.5617513
C 1.2077715 -1.8032311 0.6137871
C -0.3146116 -1.7628140 0.5909333
C -0.8803628 -2.8940525 -0.2156305
C -0.8096507 -2.8282036 -1.7014465
H 0.0667680 -2.2884780 -2.0623599
H -0.7942173 -3.8272698 -2.1426999
H -1.6896820 -2.3139582 -2.1073006
C -1.8723593 -3.8089090 0.4120144
H -1.9227700 -4.7689062 -0.1056784
H -2.8788494 -3.3715695 0.3751569
H -1.6473739 -4.0056199 1.4620839
O -0.7410008 -0.5426628 0.0560967
H -0.6563153 -1.8720561 1.6314877
H 1.5348556 -2.8174528 0.8822695
H 1.5972459 -1.5870139 -0.3895540
H 3.3346306 -0.1580078 2.4407430
H 3.6124786 -1.6265375 1.5153441
H 5.3570850 -0.8883705 0.0558079
H 6.1148612 0.5385615 -1.8033657
H 4.6891373 2.3969195 -2.6041210
H 2.5011597 2.8124136 -1.5288983
C -3.0620451 2.1092215 1.7589622
C -3.5546901 0.8140753 1.9498093
C -2.4807588 -0.0153885 2.3351110
H -4.5741691 0.4991895 1.8001303
C -1.3231362 0.7792163 2.4093801
C -1.6700958 2.0883082 2.0290133
H -1.0036101 2.9364321 1.9961935
H -3.6471509 2.9690237 1.4734996
H -2.5463100 -1.0748234 2.5301268
H -0.3275233 0.4367390 2.6482402
C -1.8416128 1.1352586 -2.2569350
C -0.5813624 1.4421305 -1.7460847
C -0.7247486 2.5145371 -0.8419338
H 0.3257011 0.8867809 -1.9241975
C -2.0771696 2.9374316 -0.8823536
C -2.7737686 2.0673667 -1.7215522
H -3.8320993 2.0944132 -1.9294300
H -2.0760451 0.3193148 -2.9215669
H 0.0682444 2.9919621 -0.2859332
H -2.4935270 3.7722898 -0.3421121
Ti -1.9763995 0.7421443 0.1234289
Cl -3.7345638 -0.6749720 -0.6264423

59
Ia-Cp2TiOMs
H 4.3696375 1.5274871 1.0139609
C 5.0882396 0.9145015 0.4832694
C 6.2482213 1.4918744 -0.0103338
C 7.1803832 0.7170541 -0.6907867
C 6.9406777 -0.6362635 -0.8781609
C 5.7729015 -1.2097278 -0.3895541
C 4.8403357 -0.4445759 0.3006436
C 3.5890062 -1.0744997 0.8616553
O 2.4368386 -0.2987841 0.6544515
C 2.0351281 -0.2442155 -0.6884743
C 0.8197830 0.6726837 -0.8388701
C 1.1281316 2.0675457 -0.3727180
C 1.0726630 2.4278954 1.0711195
H 0.7829802 3.4741374 1.2012315
H 0.3693074 1.8119492 1.6290132
H 2.0532877 2.3170337 1.5502257
C 1.9476017 2.9223406 -1.2764016

H 1.7399134 2.7293097 -2.3306362
H 3.0242883 2.7578593 -1.1283768
H 1.7707767 3.9836379 -1.0888175
O -0.2834810 0.1548059 -0.1741785
H 0.6237964 0.7156297 -1.9243665
H 1.7954415 -1.2529607 -1.0563316
H 2.8342330 0.1472319 -1.3328454
H 3.6859430 -1.1883372 1.9442958
H 3.4672270 -2.0843289 0.4445407
H 5.5864812 -2.2653774 -0.5509208
H 7.6577449 -1.2461153 -1.4124872
H 8.0864223 1.1679020 -1.0743775
H 6.4282457 2.5489208 0.1381554
C -2.9410971 -2.4430267 -1.3755691
C -3.1027202 -1.1802339 -1.9650032
C -1.8468144 -0.7374350 -2.4160765
H -4.0284235 -0.6337511 -2.0341893
C -0.9080218 -1.7503145 -2.1359463
C -1.5756106 -2.8011260 -1.4858745
H -1.1306005 -3.7291046 -1.1614566
H -3.7273813 -3.0415248 -0.9418446
H -1.6421508 0.2156584 -2.8776035
H 0.1438784 -1.7288725 -2.3752110
C -2.0960849 -2.8233821 1.4222922
C -2.7566122 -1.7301375 1.9820796
C -1.7726181 -0.7857476 2.3881704
H -3.8250109 -1.6171752 2.0879051
C -0.5177250 -1.3037036 2.0741080
C -0.7067178 -2.5354387 1.4117253
H 0.0755020 -3.1840583 1.0456879
H -2.5597105 -3.7190065 1.0399630
H -1.9695201 0.1741492 2.8402227
H 0.4285621 -0.8035115 2.2008406
Ti -1.6830461 -0.9411694 -0.0322301
O -3.0579630 0.4673540 0.2706874
S -3.5196042 1.8151828 -0.2882023
C -2.3564650 2.9735232 0.3927346
H -2.6648384 3.9741398 0.0951845
H -1.3645235 2.7622038 0.0003587
H -2.3535980 2.8973460 1.4779348
O -4.8330819 2.1164364 0.2691596
O -3.4040449 1.8879668 -1.7428250

52
Name = IIa-cis-Cp2TiCl
H -3.9150342 -1.1944042 -0.0906548
C -4.0270040 -0.1184751 -0.1028635
C -5.1003656 0.4634142 0.5431766
C -5.2709639 1.8474122 0.5498225
C -4.3364605 2.6511172 -0.1066673
C -3.2596508 2.0872801 -0.7567251
C -3.0708469 0.6826394 -0.7759526
C -1.9604947 0.1446901 -1.4418403
O -1.8255160 -1.1853682 -1.5418156
C -0.5275918 -1.6838516 -1.8084263
C 0.3443054 -1.7518237 -0.5569567
C -0.3402020 -2.4401832 0.6338818
C 0.6137448 -2.5610648 1.8187707
H 1.4573928 -3.2148405 1.5807294
H 1.0116454 -1.5955531 2.1299081
H 0.1014926 -2.9965211 2.6779452
C -0.8741474 -3.8189242 0.2538257
H -0.0868160 -4.4478720 -0.1697656
H -1.6884017 -3.7673383 -0.4684167
H -1.2596217 -4.3324196 1.1355801
O 0.7347920 -0.4658659 -0.2046256
H 1.2198516 -2.3610559 -0.8448507
H -0.0257098 -1.0725591 -2.5665186
H -0.6612594 -2.6835824 -2.2220106
H -1.1848381 -1.8144380 0.9403583
H -1.2091469 0.7730882 -1.9100522
H -2.5482824 2.7233595 -1.2707756
H -4.4578584 3.7270760 -0.1099526
H -6.1167896 2.2927744 1.0567798
H -5.8185671 -0.1676045 1.0518860
C 2.3614281 2.4820883 1.4455812
C 1.8341515 3.1636165 0.3443085
C 0.4864504 2.7401348 0.1808297
H 2.3466906 3.8956611 -0.2617028
C 0.2022930 1.7931566 1.1624088
C 1.3778140 1.5789661 1.9147311
H 1.4872332 0.8976120 2.7460486
H 3.3520744 2.6036748 1.8548397
H -0.1887710 3.0818339 -0.5862435
H -0.7219590 1.2451580 1.2573503
C 3.9908383 0.6085162 -1.5119466
C 3.4322083 -0.6785289 -1.3506214
C 3.4440626 -0.9836704 0.0200919
H 3.0611474 -1.3087632 -2.1445579
C 3.9551963 0.1272302 0.7128906
C 4.3124359 1.1091340 -0.2465514
H 4.7576020 2.0708393 -0.0438286
H 4.1203314 1.1287771 -2.4464924
H 3.0844891 -1.8929428 0.4765984
H 4.0961615 0.1945580 1.7808181
Ti 1.9439787 0.8497446 -0.2668516
Cl 1.3464875 1.5740980 -2.4614944

59
Name = IIa-cis-Cp2TiOMs
H 4.5629589 1.4298006 -0.7458385
C 4.5955423 0.4490932 -0.2906363

| | | | |
|---|---|---|---|
| C | 5.7040522 | 0.0742782 | 0.4427849 |
| C | 5.7770266 | -1.1809821 | 1.0462354 |
| C | 4.7090376 | -2.0686241 | 0.9015391 |
| C | 3.5961171 | -1.7113258 | 0.1718837 |
| C | 3.5023772 | -0.4389768 | -0.4465510 |
| C | 2.3427029 | -0.1109438 | -1.1611648 |
| O | 2.2428441 | 1.0650098 | -1.7912063 |
| C | 0.9605642 | 1.4291845 | -2.2644537 |
| C | 0.0792657 | 2.1191541 | -1.2270735 |
| C | 0.5750661 | 3.4745303 | -0.6967783 |
| C | 0.7477518 | 4.4846246 | -1.8268518 |
| H | -0.1340303 | 4.5337733 | -2.4693529 |
| H | 0.9128208 | 5.4838744 | -1.4223721 |
| H | 1.6073893 | 4.2471080 | -2.4564386 |
| C | 1.8267702 | 3.4048052 | 0.1722020 |
| H | 1.9807797 | 4.3611644 | 0.6749794 |
| H | 2.7203263 | 3.1976101 | -0.4153230 |
| H | 1.7474526 | 2.6370752 | 0.9413979 |
| O | -0.1611582 | 1.2558423 | -0.1659876 |
| H | -0.8494566 | 2.3278301 | -1.7863055 |
| H | 0.4324963 | 0.5463576 | -2.6420598 |
| H | 1.1178772 | 2.0994541 | -3.1105221 |
| H | -0.2403879 | 3.8382655 | -0.0589745 |
| H | 1.5101628 | -0.8012308 | -1.2355956 |
| H | 2.7769778 | -2.4138757 | 0.0661361 |
| H | 4.7527343 | -3.0463469 | 1.3643469 |
| H | 6.6498431 | -1.4636300 | 1.6192286 |
| H | 6.5275577 | 0.7691415 | 0.5503334 |
| C | -0.0531075 | -1.4729885 | 1.8610403 |
| C | 0.6110140 | -0.2488613 | 1.9817527 |
| C | -0.2514490 | 0.6491891 | 2.6448044 |
| H | 1.5807204 | -0.0123996 | 1.5729145 |
| C | -1.4315346 | -0.0478673 | 2.9737995 |
| C | -1.3285181 | -1.3524752 | 2.4567752 |
| H | -2.0742145 | -2.1296470 | 2.5190205 |
| H | 0.3325203 | -2.3586743 | 1.3824217 |
| H | -0.0383992 | 1.6817140 | 2.8791468 |
| H | -2.2644675 | 0.3500107 | 3.5316589 |
| C | -3.6850852 | 0.1479480 | 1.1299727 |
| C | -3.5837252 | -0.2739782 | -0.1973283 |
| C | -3.0514306 | 0.7915783 | -0.9628679 |
| H | -3.8777632 | -1.2401679 | -0.5736139 |
| C | -2.8476994 | 1.8768946 | -0.1002380 |
| C | -3.1945542 | 1.4750323 | 1.2039096 |
| H | -3.1561137 | 2.0966727 | 2.0854624 |
| H | -4.0636485 | -0.4397238 | 1.9516595 |
| H | -2.8370702 | 0.7624806 | -2.0203807 |
| H | -2.4579147 | 2.8471911 | -0.3677502 |
| Ti | -1.3535521 | 0.1492929 | 0.6003545 |
| O | -0.9560023 | -1.2696059 | -0.7561757 |
| S | -1.4982387 | -2.6301689 | -1.1971979 |
| C | -0.0289453 | -3.4364499 | -1.7959574 |
| H | 0.6920995 | -3.5414341 | -0.9880488 |
| H | 0.4031962 | -2.8597325 | -2.6104957 |
| H | -0.3072999 | -4.4240630 | -2.1586974 |
| O | -2.4070023 | -2.4789650 | -2.3287706 |
| O | -1.9991025 | -3.3921897 | -0.0571746 |

59

Name = IIa-trans-Cp2TiOMs

| | | | |
|---|---|---|---|
| H | 2.8066297 | -0.2911627 | -2.8814822 |
| C | 3.2010467 | -0.7966537 | -2.0097216 |
| C | 3.8943970 | -1.9812663 | -2.1662653 |
| C | 4.4317938 | -2.6455383 | -1.0652225 |
| C | 4.2714173 | -2.0986049 | 0.2086640 |
| C | 3.5856713 | -0.9141813 | 0.3797628 |
| C | 3.0259568 | -0.2295275 | -0.7255479 |
| C | 2.3436179 | 0.9843714 | -0.5175695 |
| O | 1.7511405 | 1.6594561 | -1.5228955 |
| C | 0.4475810 | 1.2460791 | -1.9195231 |
| C | -0.6958321 | 1.7498212 | -1.0457078 |
| C | -0.7704857 | 3.2703501 | -0.8232238 |
| C | -0.7562977 | 4.0274039 | -2.1474098 |
| H | -1.4848328 | 3.6258064 | -2.8550630 |
| H | -1.0018059 | 5.0781126 | -1.9883010 |
| H | 0.2260422 | 3.9927756 | -2.6217039 |
| C | 0.2686338 | 3.8321690 | 0.1423327 |
| H | 0.0197785 | 4.8654500 | 0.3909803 |
| H | 1.2691912 | 3.8318796 | -0.2877005 |
| H | 0.2984196 | 3.2707269 | 1.0760212 |
| O | -0.7058574 | 1.0890852 | 0.1815317 |
| H | -1.5864685 | 1.4817507 | -1.6398232 |
| H | 0.3907099 | 0.1540832 | -1.9719355 |
| H | 0.3091924 | 1.6311300 | -2.9311390 |
| H | -1.7518645 | 3.4406113 | -0.3630498 |
| H | 2.3321906 | 1.4595837 | 0.4589074 |
| H | 3.4752045 | -0.4967626 | 1.3739177 |
| H | 4.6886862 | -2.6046621 | 1.0698744 |
| H | 4.9717664 | -3.5736392 | -1.1967896 |
| H | 4.0225388 | -2.3953321 | -3.1583997 |
| C | 0.0586061 | -1.2900076 | 2.5406754 |
| C | 0.3782405 | 0.0710312 | 2.4950616 |
| C | -0.7075508 | 0.7954376 | 3.0274925 |
| H | 1.2703986 | 0.4959092 | 2.0677015 |
| C | -1.6840856 | -0.1331145 | 3.4360001 |
| C | -1.2270279 | -1.4247753 | 3.1056229 |
| H | -1.7516779 | -2.3535743 | 3.2683077 |
| H | 0.6762167 | -2.0978396 | 2.1834692 |
| H | -0.7757224 | 1.8693775 | 3.1176084 |
| H | -2.6152086 | 0.1069448 | 3.9246672 |
| C | -3.8154531 | -0.8335591 | 1.5983457 |
| C | -3.5266200 | -1.4380642 | 0.3736654 |

| | | | |
|---|---|---|---|
| C | -3.3009896 | -0.4161409 | -0.5825860 |
| H | -3.5021491 | -2.4996249 | 0.1876049 |
| C | -3.4628113 | 0.8148743 | 0.0624240 |
| C | -3.7307194 | 0.5689565 | 1.4244965 |
| H | -3.9115918 | 1.3211322 | 2.1775687 |
| H | -4.0447044 | -1.3497272 | 2.5173699 |
| H | -3.0395990 | -0.5701280 | -1.6182842 |
| H | -3.3655928 | 1.7925001 | -0.3838379 |
| Ti | -1.5541955 | -0.2385796 | 1.0568938 |
| O | -0.6931776 | -1.5941516 | -0.1098211 |
| S | -0.7724021 | -3.0912341 | -0.4100661 |
| C | 0.8802074 | -3.4367324 | -0.9612495 |
| H | 1.5921353 | -3.1877610 | -0.1774761 |
| H | 1.0998222 | -2.8448732 | -1.8458640 |
| H | 0.9464253 | -4.4963774 | -1.2000108 |
| O | -1.6784220 | -3.3440741 | -1.5274197 |
| O | -1.0205130 | -3.8591397 | 0.8081221 |

62

Name = IVc-cis

| | | | |
|---|---|---|---|
| H | 3.2761652 | -1.0502594 | 1.4435651 |
| C | 4.1389615 | -1.2065012 | 0.8112474 |
| C | 4.8527496 | -2.3816658 | 0.9338845 |
| C | 5.9742637 | -2.6274475 | 0.1445610 |
| C | 6.3691738 | -1.6605016 | -0.7783980 |
| C | 5.6680701 | -0.4795329 | -0.9144863 |
| C | 4.5231012 | -0.2083335 | -0.1229023 |
| C | 3.7827376 | 0.9910303 | -0.2533437 |
| O | 2.6865844 | 1.0707624 | 0.5293761 |
| C | 1.8060445 | 2.1693372 | 0.4281097 |
| C | 0.5111367 | 1.8368307 | 1.1558454 |
| C | 0.6893532 | 1.4643027 | 2.6359971 |
| C | -0.6712203 | 1.2526598 | 3.2939991 |
| H | -0.5503536 | 0.9861596 | 4.3452086 |
| H | -1.2689992 | 2.1678038 | 3.2570267 |
| H | -1.2420446 | 0.4625932 | 2.8100297 |
| C | 1.4815798 | 2.5279162 | 3.3907358 |
| H | 1.5044406 | 2.2985948 | 4.4567284 |
| H | 1.0269444 | 3.5160271 | 3.2824251 |
| H | 2.5165061 | 2.5950668 | 3.0551559 |
| O | -0.1447330 | 0.8117543 | 0.4946158 |
| H | -0.0823265 | 2.7684223 | 1.1232488 |
| H | 1.5686661 | 2.3890829 | -0.6170848 |
| H | 2.2532538 | 3.0713092 | 0.8600121 |
| H | 1.2440724 | 0.5217910 | 2.6806079 |
| C | 4.0988496 | 2.0982833 | -1.1985186 |
| H | 6.0070960 | 0.2385040 | -1.6488003 |
| H | 7.2362774 | -1.8334987 | -1.4038161 |
| H | 6.5275151 | -3.5517154 | 0.2452505 |
| H | 4.5308141 | -3.1208291 | 1.6570750 |
| C | -3.0201072 | 1.0665094 | -2.1189456 |
| C | -3.3770228 | 1.5103753 | -0.8335701 |
| C | -2.3732257 | 2.3704740 | -0.3662850 |
| H | -4.2535938 | 1.1997508 | -0.2892365 |
| C | -1.3971857 | 2.4898182 | -1.3805956 |
| C | -1.7989995 | 1.6942039 | -2.4666578 |
| H | -1.2674522 | 1.5834403 | -3.3992598 |
| H | -3.5889514 | 0.3835175 | -2.7320274 |
| H | -2.3425394 | 2.8336540 | 0.6090765 |
| H | -0.4941888 | 3.0809825 | -1.3335054 |
| C | -1.5228772 | -1.9749786 | -1.6970552 |
| C | -0.5111467 | -2.0695304 | -0.6996702 |
| C | 0.5519329 | -1.2558819 | -1.0863589 |
| H | -0.5738875 | -2.6472671 | 0.2091869 |
| C | 0.1912411 | -0.5978735 | -2.2827313 |
| C | -1.0706142 | -1.1042715 | -2.6892301 |
| H | -1.6044157 | -0.8302221 | -3.5859072 |
| H | -2.4681396 | -2.4960233 | -1.6952946 |
| H | 1.4475200 | -1.0720064 | -0.5147154 |
| H | 0.7992993 | 0.1081457 | -2.8305418 |
| Ti | -1.3414665 | 0.2105420 | -0.6905265 |
| O | -2.5073064 | -0.5842405 | 0.7134910 |
| S | -3.6598398 | -1.5812979 | 0.8453917 |
| C | -4.4001097 | -1.0622231 | 2.3775299 |
| H | -5.2411580 | -1.7206447 | 2.5862079 |
| H | -4.7456754 | -0.0345744 | 2.2827939 |
| H | -3.6647301 | -1.1346883 | 3.1760012 |
| O | -3.1590119 | -2.9404040 | 1.0249251 |
| O | -4.6442105 | -1.3956754 | -0.2190857 |
| H | 5.0993831 | 1.9936879 | -1.6096989 |
| H | 4.0574948 | 3.0761210 | -0.7117586 |
| H | 3.4052844 | 2.1369790 | -2.0462378 |

62

Name = IVc-trans

| | | | |
|---|---|---|---|
| H | 2.1544123 | 1.0711748 | -2.0913393 |
| C | 2.9237280 | 0.3629639 | -2.3624004 |
| C | 3.2482404 | 0.2116567 | -3.6955747 |
| C | 4.2495890 | -0.6718290 | -4.0943467 |
| C | 4.9264229 | -1.4010964 | -3.1182624 |
| C | 4.6132805 | -1.2610734 | -1.7812120 |
| C | 3.5940710 | -0.3741865 | -1.3507140 |
| C | 3.2385815 | -0.2397262 | 0.0136183 |
| O | 2.1348757 | 0.5012687 | 0.2468179 |
| C | 1.7296857 | 0.8002493 | 1.5686268 |
| C | 0.2877456 | 1.2924500 | 1.5234405 |
| C | -0.2345950 | 1.6372827 | 2.9278102 |
| C | -1.6777899 | 2.1283043 | 2.8623876 |
| H | -2.0488093 | 2.3454827 | 3.8650452 |
| H | -1.7484052 | 3.0531004 | 2.2833923 |
| H | -2.3448657 | 1.3963625 | 2.4113093 |
| C | 0.6374829 | 2.6864158 | 3.6162596 |

H 0.1750544 3.0058549 4.5508085
H 0.7556170 3.5747453 2.9910291
H 1.6324892 2.3178839 3.8651614
O -0.5005735 0.3066359 0.9526780
H 0.2526330 2.2155901 0.9199288
H 2.3846063 1.5684364 1.9894246
H 1.7825129 -0.0814961 2.2154019
H -0.2100772 0.7178682 3.5243016
C 3.9343120 -0.9085547 1.1478443
H 5.1550601 -1.8611141 -1.0633353
H 5.7061912 -2.0945716 -3.4071616
H 4.4981003 -0.7884451 -5.1406050
H 2.7183873 0.7959770 -4.4375635
C -2.3490976 0.2503994 -2.4731642
C -2.9305867 1.1106833 -1.5268410
C -1.9146223 1.8894059 -0.9534686
H -3.9754909 1.1343666 -1.2653919
C -0.6962724 1.5314959 -1.5655605
C -0.9609493 0.5248681 -2.5085125
H -0.2332873 0.0637479 -3.1586700
H -2.8783371 -0.4664395 -3.0815792
H -2.0419995 2.6128065 -0.1625724
H 0.2726835 1.9503359 -1.3424973
C -1.9256620 -2.7354500 -0.8519446
C -1.2644155 -2.6938063 0.4080750
C 0.0378166 -2.2467681 0.1952216
H -1.7047351 -2.9474940 1.3590201
C 0.1833542 -1.9445535 -1.1759180
C -1.0197717 -2.3174488 -1.8255404
H -1.2110813 -2.2581033 -2.8854131
H -2.9396387 -3.0578308 -1.0300723
H 0.7781598 -2.0790699 0.9604155
H 1.0794506 -1.5778145 -1.6544237
Ti -1.4242170 -0.4111720 -0.3948337
O -3.0988813 -0.5058982 0.6882944
S -4.4545024 -1.2109743 0.6839900
C -5.4153971 -0.1425227 1.7332071
H -6.4172656 -0.5588152 1.8172848
H -5.4676327 0.8517351 1.2956842
H -4.9576717 -0.0912315 2.7182824
O -4.3720807 -2.5139476 1.3372735
O -5.0540179 -1.1959367 -0.6483234
H 4.8881573 -1.3235634 0.8365896
H 3.3509396 -1.7320900 1.5736716
H 4.1490890 -0.2165249 1.9649459

13
Name = THF
C -0.0358205 1.0292969 -0.2873202
C -1.2997371 0.3015638 0.1511085
O -1.0032968 -1.0854418 0.0889409
C 0.3946011 -1.2823372 -0.0646182
C 1.0491627 0.0694721 0.1893150
H 0.0431588 2.0254992 0.1472826
H -0.0043259 1.1342344 -1.3741916
H -2.1576740 0.5192520 -0.4899297
H -1.5716967 0.5819420 1.1759995
H 0.6132708 -1.6343740 -1.0799948
H 0.7328912 -2.0532315 0.6323811
H 1.9933284 0.1812351 -0.3431217
H 1.2461381 0.2128890 1.2541484

30
Name = trans-2a
H -1.9263518 -2.1749909 -0.1517087
C -2.1597942 -1.1304014 0.0078097
C -3.2845708 -0.7862626 0.7464415
C -3.5969622 0.5483231 0.9631501
C -2.7786928 1.5380949 0.4346839
C -1.6555538 1.1940344 -0.3039916
C -1.3345359 -0.1432814 -0.5203623
C -0.1260633 -0.5075072 -1.3661736
O 0.3866370 -1.7666165 -1.0349425
C 1.3709314 -1.5626195 -0.0339308
H 1.0309182 -1.9308097 0.9371962
H 2.2715325 -2.1155369 -0.3156854
C 1.6019715 -0.0392314 -0.0169514
C 3.0599650 0.3874604 -0.0550668
C 3.1958347 1.8978529 -0.2137165
H 2.7451156 2.2548203 -1.1388734
H 4.2475267 2.1876741 -0.2245778
H 2.7184111 2.4225521 0.6173185
C 3.7633082 -0.0841390 1.2150759
H 3.7218860 -1.1680223 1.3370679
H 4.8160794 0.1990465 1.1992134
H 3.3142804 0.3674957 2.1026521
H 3.5303086 -0.1019489 -0.9161743
H 1.1248669 0.4032158 0.8699317
O 0.9244080 0.3893303 -1.1888450
H -0.3970337 -0.5123783 -2.4272606
H -1.0225994 1.9746628 -0.7062910
H -3.0137161 2.5817063 0.5999503
H -4.4721659 0.8162614 1.5407421
H -3.9162874 -1.5643020 1.1554319

33
Name = trans-4c
H 2.7196702 0.6157797 -1.7983773
C 1.8844220 0.1070872 -2.2636827
C 1.7862903 0.0843025 -3.6470461
C 0.7183109 -0.5581489 -4.2605296
C -0.2476105 -1.1762364 -3.4797227

| | | | |
|---|---|---|---|
| C | -0.1473979 | -1.1539284 | -2.0939137 |
| C | 0.9183802 | -0.5117337 | -1.4742629 |
| C | 1.0732587 | -0.5186681 | 0.0439171 |
| O | 1.5109297 | 0.7318219 | 0.4995034 |
| C | 0.3510798 | 1.5329125 | 0.5696077 |
| C | -0.7579470 | 0.5347641 | 0.9490069 |
| C | -1.1933842 | 0.6046979 | 2.4102152 |
| C | -2.0525111 | -0.5943188 | 2.7969490 |
| H | -2.3644099 | -0.5198032 | 3.8399387 |
| H | -2.9580295 | -0.6402316 | 2.1865767 |
| H | -1.5181146 | -1.5354310 | 2.6774686 |
| C | -1.9529872 | 1.9031269 | 2.6674568 |
| H | -2.2487589 | 1.9748034 | 3.7144805 |
| H | -2.8631775 | 1.9500256 | 2.0651831 |
| H | -1.3597359 | 2.7898277 | 2.4391793 |
| O | -0.1623213 | -0.7253588 | 0.6739205 |
| H | -1.6383235 | 0.6735341 | 0.3075460 |
| H | 0.1406775 | 2.0187030 | -0.3894757 |
| H | 0.5120778 | 2.3092427 | 1.3183444 |
| H | -0.2880178 | 0.6005495 | 3.0294087 |
| C | 2.0520603 | -1.5897171 | 0.4833579 |
| H | -0.9080708 | -1.6395443 | -1.4967730 |
| H | -1.0845696 | -1.6775064 | -3.9486047 |
| H | 0.6405845 | -0.5758646 | -5.3398918 |
| H | 2.5445181 | 0.5705387 | -4.2475145 |
| H | 1.6998108 | -2.5727642 | 0.1727472 |
| H | 2.1650156 | -1.5842686 | 1.5679133 |
| H | 3.0282807 | -1.4281932 | 0.0270737 |

34

Name = trans-6a

| | | | |
|---|---|---|---|
| H | 1.9956526 | 0.3349504 | 2.3177412 |
| C | 2.3961311 | 0.3175780 | 1.3104836 |
| C | 3.7437436 | 0.5862248 | 1.0986624 |
| C | 4.2577020 | 0.5648389 | -0.1888186 |
| C | 3.4219097 | 0.2730203 | -1.2622994 |
| C | 2.0796325 | 0.0054896 | -1.0474075 |
| C | 1.5583879 | 0.0259686 | 0.2435235 |
| C | 0.0970225 | -0.2480920 | 0.4722881 |
| N | -0.3521473 | -1.4558206 | -0.1794286 |
| C | 0.0632295 | -2.6714317 | 0.4739418 |
| H | -0.2906286 | -3.5344136 | -0.0897356 |
| H | 1.1516956 | -2.7251742 | 0.5098516 |
| H | -0.3169849 | -2.7600567 | 1.5030809 |
| C | -1.7817516 | -1.2771450 | -0.2873438 |
| H | -2.2053102 | -1.9213472 | -1.0596011 |
| C | -1.8878129 | 0.2085334 | -0.6241122 |
| C | -3.1202510 | 0.8952619 | -0.0507409 |
| C | -3.0944204 | 2.3982485 | -0.3057491 |
| H | -3.0732018 | 2.6135186 | -1.3769635 |
| H | -3.9855320 | 2.8740295 | 0.1065832 |
| H | -2.2252367 | 2.8727173 | 0.1481961 |
| C | -4.3809122 | 0.2708046 | -0.6428229 |
| H | -4.4007981 | 0.3839957 | -1.7293943 |
| H | -5.2749243 | 0.7536728 | -0.2468433 |
| H | -4.4606136 | -0.7941787 | -0.4185096 |
| H | -3.1255239 | 0.7250428 | 1.0327666 |
| H | -1.8826033 | 0.3507165 | -1.7139298 |
| O | -0.6862856 | 0.7691822 | -0.0998087 |
| H | -2.3060877 | -1.4992953 | 0.6594723 |
| H | -0.0902887 | -0.2904939 | 1.5662254 |
| H | 1.4321192 | -0.2324803 | -1.8818523 |
| H | 3.8216763 | 0.2535870 | -2.2681371 |
| H | 5.3062908 | 0.7727526 | -0.3589336 |
| H | 4.3882949 | 0.8116969 | 1.9383933 |

59

Name = TS2a-cis

| | | | |
|---|---|---|---|
| H | 4.0910455 | 0.2715983 | 0.3327318 |
| C | 3.8114381 | -0.7654379 | 0.4602509 |
| C | 4.5759010 | -1.5846145 | 1.2682407 |
| C | 4.2347114 | -2.9255339 | 1.4313330 |
| C | 3.1285005 | -3.4565982 | 0.7812349 |
| C | 2.3535824 | -2.6407389 | -0.0227058 |
| C | 2.6836195 | -1.2906949 | -0.1889892 |
| C | 1.8752117 | -0.5250307 | -1.0966708 |
| O | 2.3027893 | 0.5944132 | -1.5191661 |
| C | 1.3661863 | 1.3860352 | -2.2621586 |
| C | 0.2564515 | 1.8537426 | -1.2873380 |
| C | 0.6078615 | 3.1422482 | -0.5168538 |
| C | 0.7957343 | 4.3302973 | -1.4581693 |
| H | -0.0109329 | 4.3997581 | -2.1916973 |
| H | 0.8052078 | 5.2702373 | -0.9020316 |
| H | 1.7393716 | 4.2746576 | -2.0083519 |
| C | 1.7872012 | 2.9997125 | 0.4348514 |
| H | 1.8754139 | 3.8863339 | 1.0650521 |
| H | 2.7342527 | 2.8923004 | -0.0985824 |
| H | 1.6641128 | 2.1408102 | 1.0937296 |
| O | -0.0549860 | 0.7958624 | -0.4924474 |
| H | -0.5636625 | 2.1286323 | -1.9782644 |
| H | 0.9341940 | 0.7603886 | -3.0455261 |
| H | 1.9386433 | 2.1896961 | -2.7171538 |
| H | -0.2760622 | 3.3506964 | 0.0966879 |
| H | 1.0187108 | -0.9975004 | -1.5701096 |
| H | 1.5016595 | -3.0511845 | -0.5474027 |
| H | 2.8775937 | -4.5016398 | 0.9004705 |
| H | 4.8395224 | -3.5596738 | 2.0668314 |
| H | 5.4451226 | -1.1871937 | 1.7743581 |
| C | -0.6131732 | -1.4659444 | 1.9466256 |
| C | 0.0475755 | -0.2427179 | 2.1150765 |
| C | -0.8755979 | 0.6924301 | 2.6233839 |
| H | 1.0701428 | -0.0352021 | 1.8438730 |

| | | | |
|---|---|---|---|
| C | -2.1017970 | 0.0183521 | 2.8103005 |
| C | -1.9515806 | -1.3081669 | 2.3672823 |
| H | -2.7172941 | -2.0674712 | 2.3474218 |
| H | -0.1790783 | -2.3668706 | 1.5416858 |
| H | -0.6729128 | 1.7279833 | 2.8549787 |
| H | -3.0018819 | 0.4466756 | 3.2230150 |
| C | -4.1250565 | 0.5318697 | 0.6146265 |
| C | -3.8833448 | 0.1129603 | -0.6998367 |
| C | -3.0653420 | 1.0773454 | -1.3231800 |
| H | -4.2578032 | -0.7899405 | -1.1526896 |
| C | -2.8356022 | 2.1156924 | -0.3958254 |
| C | -3.4754558 | 1.7748298 | 0.8045229 |
| H | -3.4801378 | 2.3652007 | 1.7087485 |
| H | -4.7051890 | -0.0032775 | 1.3516293 |
| H | -2.6974931 | 1.0323255 | -2.3372598 |
| H | -2.2748011 | 3.0202453 | -0.5729521 |
| Ti | -1.7236098 | 0.1196748 | 0.4380719 |
| O | -1.3418086 | -1.5172676 | -0.8658234 |
| S | -2.1966400 | -2.7171219 | -1.2034279 |
| C | -1.0140176 | -4.0527489 | -1.2795701 |
| H | -0.5428245 | -4.1777868 | -0.3064395 |
| H | -0.2646003 | -3.8458564 | -2.0408147 |
| H | -1.5423368 | -4.9683863 | -1.5367359 |
| O | -2.7776983 | -2.5970148 | -2.5420492 |
| O | -3.1390371 | -3.0613908 | -0.1367912 |

59

Name = TS2a-trans

| | | | |
|---|---|---|---|
| H | 2.9180905 | -0.1820563 | -2.5173341 |
| C | 3.1744240 | -0.7915375 | -1.6622595 |
| C | 3.8253885 | -1.9895146 | -1.8685917 |
| C | 4.2076590 | -2.7742905 | -0.7843026 |
| C | 3.9576549 | -2.3539313 | 0.5165081 |
| C | 3.3393786 | -1.1388312 | 0.7330809 |
| C | 2.9220097 | -0.3532818 | -0.3515433 |
| C | 2.4843065 | 0.9758020 | -0.0562852 |
| O | 2.0053175 | 1.8292448 | -0.8526072 |
| C | 0.9297657 | 1.5289606 | -1.7763749 |
| C | -0.3611090 | 1.8252769 | -0.9910253 |
| C | -0.6341836 | 3.3307937 | -0.7648666 |
| C | -0.6910850 | 4.1167882 | -2.0706837 |
| H | -1.3254970 | 3.6261308 | -2.8126937 |
| H | -1.0990732 | 5.1147173 | -1.9038541 |
| H | 0.2982604 | 4.2493964 | -2.5147634 |
| C | 0.2819022 | 4.0071254 | 0.2499575 |
| H | -0.1346141 | 4.9730099 | 0.5422387 |
| H | 1.2786787 | 4.1990344 | -0.1494797 |
| H | 0.3818001 | 3.4043431 | 1.1525533 |
| O | -0.3532793 | 1.1169521 | 0.1636513 |
| H | -1.1274686 | 1.4877064 | -1.7201809 |
| H | 0.9446447 | 0.4772875 | -2.0562612 |
| H | 1.0840455 | 2.1577808 | -2.6498210 |
| H | -1.6397130 | 3.3555279 | -0.3313380 |
| H | 2.7523981 | 1.3904530 | 0.9139957 |
| H | 3.1879290 | -0.7880526 | 1.7458273 |
| H | 4.2666894 | -2.9614379 | 1.3553779 |
| H | 4.7131905 | -3.7151024 | -0.9566051 |
| H | 4.0424610 | -2.3152956 | -2.8762724 |
| C | -0.2190955 | -1.5860699 | 2.5355185 |
| C | 0.2297893 | -0.2570278 | 2.5887175 |
| C | -0.8171093 | 0.5495071 | 3.0810105 |
| H | 1.1976213 | 0.1004300 | 2.2820280 |
| C | -1.9108072 | -0.2931526 | 3.3414560 |
| C | -1.5504192 | -1.6121186 | 2.9848370 |
| H | -2.1825846 | -2.4851836 | 3.0406617 |
| H | 0.3365976 | -2.4347984 | 2.1699303 |
| H | -0.7819369 | 1.6187447 | 3.2269325 |
| H | -2.8654311 | 0.0184387 | 3.7372273 |
| C | -3.9450839 | -0.6843791 | 1.1945822 |
| C | -3.5534865 | -1.2701718 | -0.0170380 |
| C | -3.1537856 | -0.2327059 | -0.8894009 |
| H | -3.5675406 | -2.3240493 | -0.2419801 |
| C | -3.3197594 | 0.9883093 | -0.2128794 |
| C | -3.7816142 | 0.7154850 | 1.0843476 |
| H | -3.9953019 | 1.4466596 | 1.8500319 |
| H | -4.3040847 | -1.2154591 | 2.0629926 |
| H | -2.7803753 | -0.3630628 | -1.8935122 |
| H | -3.1124841 | 1.9695133 | -0.6089251 |
| Ti | -1.5613143 | -0.2874978 | 0.9395283 |
| O | -0.4827125 | -1.5295490 | -0.4008004 |
| S | -0.7957775 | -2.9507295 | -0.8049266 |
| C | 0.8065625 | -3.6260326 | -1.1947705 |
| H | 1.4507884 | -3.5658307 | -0.3207653 |
| H | 1.2501857 | -3.0697444 | -2.0159197 |
| H | 0.6824805 | -4.6674881 | -1.4856100 |
| O | -1.5790632 | -3.0033420 | -2.0435010 |
| O | -1.3242294 | -3.7516946 | 0.3041802 |

59

Name = est. TSrot

| | | | |
|---|---|---|---|
| H | 3.5546556 | 0.0470548 | -2.6742453 |
| C | 3.8312487 | -0.6406574 | -1.8863411 |
| C | 4.7947780 | -1.6015427 | -2.1222281 |
| C | 5.1704121 | -2.4989819 | -1.1232158 |
| C | 4.5657439 | -2.4180549 | 0.1321525 |
| C | 3.6028167 | -1.4641136 | 0.3834856 |
| C | 3.2011215 | -0.5513274 | -0.6221977 |
| C | 2.1956433 | 0.3871829 | -0.3391486 |
| O | 1.8071032 | 1.2897151 | -1.2588679 |
| C | 0.4678848 | 1.1992431 | -1.7314350 |
| C | -0.6124365 | 1.8633853 | -0.8835398 |
| C | -0.4169165 | 3.3553817 | -0.5679377 |
| C | -0.1778484 | 4.1686150 | -1.8364260 |

| | | | |
|---|---|---|---|
| H | -0.9259086 | 3.9575769 | -2.6038423 |
| H | -0.2264234 | 5.2362972 | -1.6189070 |
| H | 0.8065089 | 3.9706399 | -2.2644262 |
| C | 0.6528518 | 3.6486548 | 0.4799326 |
| H | 0.5886458 | 4.6922971 | 0.7928547 |
| H | 1.6574291 | 3.4833038 | 0.0925765 |
| H | 0.5280108 | 3.0322936 | 1.3700649 |
| O | -0.8537336 | 1.1665531 | 0.3036686 |
| H | -1.4864018 | 1.8026143 | -1.5530330 |
| H | 0.1961987 | 0.1514198 | -1.8871521 |
| H | 0.4700968 | 1.6813613 | -2.7105673 |
| H | -1.3764377 | 3.6830128 | -0.1486466 |
| H | 1.7262811 | 0.4255599 | 0.6359527 |
| H | 3.1408015 | -1.4108486 | 1.3629969 |
| H | 4.8528490 | -3.1083271 | 0.9151117 |
| H | 5.9251923 | -3.2490001 | -1.3183629 |
| H | 5.2636561 | -1.6564307 | -3.0966274 |
| C | -0.6227795 | -1.4177796 | 2.6212672 |
| C | -0.1612484 | -0.0946372 | 2.6681562 |
| C | -1.2322137 | 0.7283150 | 3.0665233 |
| H | 0.8232589 | 0.2392858 | 2.3868131 |
| C | -2.3520080 | -0.0954412 | 3.2842004 |
| C | -1.9815119 | -1.4254776 | 2.9888883 |
| H | -2.6174001 | -2.2959169 | 3.0402173 |
| H | -0.0527214 | -2.2824769 | 2.3233101 |
| H | -1.2037197 | 1.8025653 | 3.1709994 |
| H | -3.3194133 | 0.2350878 | 3.6287205 |
| C | -4.2141195 | -0.6800554 | 1.1014468 |
| C | -3.7444044 | -1.2808206 | -0.0691265 |
| C | -3.3295223 | -0.2554583 | -0.9579018 |
| H | -3.7171784 | -2.3412099 | -0.2639886 |
| C | -3.5505064 | 0.9710721 | -0.3239906 |
| C | -4.0495085 | 0.7187330 | 0.9702627 |
| H | -4.3152456 | 1.4688172 | 1.7000230 |
| H | -4.6092897 | -1.1944256 | 1.9632770 |
| H | -2.8985522 | -0.4103933 | -1.9350591 |
| H | -3.3377593 | 1.9491653 | -0.7263797 |
| Ti | -1.8727809 | -0.1792473 | 0.9523857 |
| O | -0.8468056 | -1.4844686 | -0.1401066 |
| S | -0.9175166 | -2.9448230 | -0.5900077 |
| C | 0.7961469 | -3.3182782 | -0.8824118 |
| H | 1.3643500 | -3.1855671 | 0.0347625 |
| H | 1.1901610 | -2.6581600 | -1.6516890 |
| H | 0.8668009 | -4.3526874 | -1.2128819 |
| O | -1.6245018 | -3.0565221 | -1.8621950 |
| O | -1.3938337 | -3.8020733 | 0.4928356 |

63

Name = Va-Cp2TiOMs

| | | | |
|---|---|---|---|
| H | -5.4414090 | 1.1813401 | -1.3714457 |
| C | -5.0253738 | 0.2686745 | -0.9599486 |
| C | -5.2721921 | -0.9456290 | -1.5913360 |
| C | -4.7480907 | -2.1182368 | -1.0691186 |
| C | -3.9729567 | -2.0686821 | 0.0849784 |
| C | -3.7215842 | -0.8549780 | 0.7037543 |
| C | -4.2542344 | 0.3273450 | 0.1944091 |
| C | -4.0182717 | 1.6287206 | 0.9144012 |
| N | -2.6076966 | 1.9087157 | 1.1070106 |
| C | -2.4050097 | 2.9071641 | 2.1353260 |
| H | -2.8557019 | 3.8815302 | 1.8900649 |
| H | -2.8302119 | 2.5666162 | 3.0801933 |
| H | -1.3401280 | 3.0751732 | 2.3014184 |
| C | -1.9390905 | 2.2781862 | -0.1306502 |
| C | -0.4547419 | 1.8941144 | -0.1406384 |
| C | 0.3176784 | 2.6551104 | -1.1765564 |
| C | 0.2592552 | 2.2382480 | -2.6028047 |
| H | 1.0583602 | 1.5254968 | -2.8451123 |
| H | -0.6825884 | 1.7505270 | -2.8584517 |
| H | 0.3893171 | 3.0910781 | -3.2728841 |
| C | 1.3562571 | 3.6181192 | -0.7172507 |
| H | 2.2000625 | 3.1030157 | -0.2399107 |
| H | 0.9676963 | 4.3159922 | 0.0307801 |
| H | 1.7576974 | 4.2107733 | -1.5404064 |
| O | -0.3432354 | 0.5162526 | -0.3753698 |
| H | -0.0298096 | 2.1565526 | 0.8407984 |
| H | -2.0358402 | 3.3597440 | -0.3199551 |
| H | -2.4185943 | 1.7708515 | -0.9709605 |
| H | -4.4819938 | 1.5660918 | 1.9033493 |
| H | -4.5400024 | 2.4449402 | 0.3877214 |
| H | -3.1106493 | -0.8165283 | 1.5969168 |
| H | -3.5626297 | -2.9820006 | 0.4983311 |
| H | -4.9407672 | -3.0657959 | -1.5554960 |
| H | -5.8738175 | -0.9726045 | -2.4910405 |
| C | 1.3088602 | 0.1722845 | 2.1813682 |
| C | -0.0455629 | -0.1686771 | 2.2550389 |
| C | -0.1478996 | -1.5726151 | 2.1595633 |
| H | -0.8877371 | 0.5080892 | 2.2791683 |
| C | 1.1632947 | -2.1023399 | 2.1075734 |
| C | 2.0578718 | -1.0305255 | 2.0893460 |
| H | 3.1320540 | -1.1113867 | 2.0376759 |
| H | 1.7221754 | 1.1690278 | 2.1761843 |
| H | -1.0631450 | -2.1431596 | 2.2011102 |
| H | 1.4240398 | -3.1479879 | 2.0683398 |
| C | -0.7382204 | -2.0056437 | -1.4005757 |
| C | -0.2320024 | -2.9445489 | -0.4808666 |
| C | 1.1595316 | -3.0692241 | -0.7047553 |
| H | -0.8110041 | -3.5041822 | 0.2372666 |
| C | 1.5015823 | -2.2039690 | -1.7546503 |
| C | 0.3344763 | -1.5420936 | -2.1810188 |
| H | 0.2814085 | -0.7786400 | -2.9409483 |
| H | -1.7607310 | -1.6644914 | -1.4534781 |
| H | 1.8398945 | -3.7196743 | -0.1779066 |

| | | | |
|---|---|---|---|
| H | 2.4979211 | -2.0537885 | -2.1388164 |
| Ti | 0.7157384 | -0.8482617 | 0.0995519 |
| O | 2.2987933 | 0.1655109 | -0.5646318 |
| S | 3.8093771 | 0.2760715 | -0.3958368 |
| C | 4.2797031 | 1.1152833 | -1.8929885 |
| H | 5.3589921 | 1.2536817 | -1.8821741 |
| H | 3.7876411 | 2.0839456 | -1.9411229 |
| H | 3.9968870 | 0.5135435 | -2.7536593 |
| O | 4.4401688 | -1.0415708 | -0.4036208 |
| O | 4.1490050 | 1.1473804 | 0.7264757 |

63

Name = VIa-cis-Cp2TiOMs

| | | | |
|---|---|---|---|
| H | 2.2597560 | 0.0243743 | -1.0745019 |
| C | 3.3187043 | 0.1824996 | -0.9081616 |
| C | 4.0716442 | 0.9519891 | -1.7781743 |
| C | 5.4088815 | 1.2179829 | -1.5113273 |
| C | 5.9982401 | 0.7352768 | -0.3495103 |
| C | 5.2497788 | -0.0165978 | 0.5375137 |
| C | 3.9150203 | -0.3308019 | 0.2487682 |
| C | 3.2596252 | -1.1588339 | 1.2435629 |
| N | 2.3682130 | -2.0746801 | 1.1099094 |
| C | 1.9336633 | -2.8344324 | 2.2779572 |
| H | 2.3433624 | -2.4074023 | 3.1878274 |
| H | 0.8480445 | -2.8258352 | 2.3338099 |
| H | 2.2691713 | -3.8648168 | 2.1738108 |
| C | 1.7359951 | -2.4819785 | -0.1406595 |
| C | 0.3238979 | -1.8624537 | -0.2860610 |
| C | -0.3665577 | -2.4378304 | -1.5428137 |
| C | -0.7110377 | -3.9163414 | -1.3862494 |
| H | -1.3464694 | -4.2542803 | -2.2062683 |
| H | 0.1748211 | -4.5563296 | -1.3887000 |
| H | -1.2531763 | -4.1042217 | -0.4566121 |
| C | 0.4095940 | -2.1906437 | -2.8314709 |
| H | 1.3116538 | -2.8046037 | -2.8973815 |
| H | 0.7064751 | -1.1462973 | -2.9260674 |
| H | -0.2024817 | -2.4355048 | -3.7010539 |
| O | 0.4443912 | -0.5062952 | -0.2874827 |
| H | -0.2733544 | -2.2307221 | 0.5697043 |
| H | 1.6906305 | -3.5717877 | -0.1144117 |
| H | 2.3822595 | -2.1924142 | -0.9667954 |
| H | -1.3106211 | -1.8918551 | -1.6088445 |
| H | 3.6376607 | -1.0467667 | 2.2538344 |
| H | 5.7067591 | -0.3771338 | 1.4510146 |
| H | 7.0333464 | 0.9567992 | -0.1288643 |
| H | 5.9876974 | 1.8195222 | -2.1997485 |
| H | 3.6075447 | 1.3607243 | -2.6658820 |
| C | -1.2785000 | 1.0179253 | 2.6429845 |
| C | -0.0034130 | 0.4474719 | 2.4837660 |
| C | 0.8607614 | 1.4381971 | 1.9691218 |
| H | 0.2508796 | -0.5793954 | 2.6959346 |
| C | 0.1108816 | 2.6148939 | 1.8063953 |
| C | -1.2171787 | 2.3516403 | 2.2126432 |
| H | -2.0418952 | 3.0478490 | 2.1911664 |
| H | -2.1672711 | 0.5090564 | 2.9805433 |
| H | 1.9056553 | 1.3203347 | 1.7236395 |
| H | 0.4860434 | 3.5552342 | 1.4316466 |
| C | -2.3113260 | 2.1386323 | -1.2041137 |
| C | -1.6382731 | 1.1978788 | -2.0198570 |
| C | -0.2932797 | 1.5889063 | -2.1135509 |
| H | -2.0815938 | 0.3241735 | -2.4709855 |
| C | -0.1130549 | 2.7423816 | -1.3334778 |
| C | -1.3725690 | 3.0953207 | -0.7924996 |
| H | -1.5731695 | 3.9474851 | -0.1611977 |
| H | -3.3588214 | 2.1241350 | -0.9446327 |
| H | 0.4829580 | 1.0597491 | -2.6437466 |
| H | 0.8147403 | 3.2773971 | -1.1917795 |
| Ti | -0.7555054 | 0.9725792 | 0.2304460 |
| O | -2.1796342 | -0.5493830 | 0.3929804 |
| S | -3.6848111 | -0.6119065 | 0.3513037 |
| C | -4.0194750 | -2.1784748 | 1.1389963 |
| H | -5.0955023 | -2.3405118 | 1.1557314 |
| H | -3.6372544 | -2.1663275 | 2.1574483 |
| H | -3.5405969 | -2.9782621 | 0.5777815 |
| O | -4.1910952 | -0.7143242 | -1.0199822 |
| O | -4.3123738 | 0.4259017 | 1.1738097 |

63

VIa-trans-Cp2TiOMs

| | | | |
|---|---|---|---|
| H | 3.9541983 | -1.3786100 | 2.5969922 |
| C | 3.9796630 | -0.3569894 | 2.2465368 |
| C | 4.7030428 | 0.5792362 | 2.9653315 |
| C | 4.7886503 | 1.8921363 | 2.5199652 |
| C | 4.1751541 | 2.2679996 | 1.3316136 |
| C | 3.4723625 | 1.3318487 | 0.5966208 |
| C | 3.3412598 | 0.0181307 | 1.0606250 |
| C | 2.5518093 | -0.8613409 | 0.2222970 |
| N | 1.8837240 | -1.9098728 | 0.5521690 |
| C | 1.6525791 | -2.3626966 | 1.9151195 |
| H | 1.6954928 | -1.5351487 | 2.6175914 |
| H | 0.6629133 | -2.8083967 | 1.9635792 |
| H | 2.3841507 | -3.1194520 | 2.1933480 |
| C | 1.0659771 | -2.5180217 | -0.4830531 |
| C | -0.2443356 | -1.6765187 | -0.6754459 |
| C | -0.8768521 | -2.0823285 | -2.0284313 |
| C | -1.2206111 | -3.5688274 | -2.0758481 |
| H | -1.8334310 | -3.7954319 | -2.9497792 |
| H | -0.3322649 | -4.2023336 | -2.1403035 |
| H | -1.7864240 | -3.8784547 | -1.1938188 |
| C | -0.0427423 | -1.6688864 | -3.2352765 |
| H | 0.8723495 | -2.2576717 | -3.3402397 |
| H | 0.2372017 | -0.6178014 | -3.1804599 |

| | | | |
|---|---|---|---|
| H | -0.6125722 | -1.8110499 | -4.1551454 |
| O | 0.0509786 | -0.3557327 | -0.5705460 |
| H | -0.9455968 | -2.0192820 | 0.1106208 |
| H | 0.8528178 | -3.5465563 | -0.1988093 |
| H | 1.6523428 | -2.5396312 | -1.4003787 |
| H | -1.8128700 | -1.5251590 | -2.0786068 |
| H | 2.5392728 | -0.6226711 | -0.8323091 |
| H | 2.9997336 | 1.6180680 | -0.3346179 |
| H | 4.2540774 | 3.2860344 | 0.9756358 |
| H | 5.3469758 | 2.6202123 | 3.0930984 |
| H | 5.2048905 | 0.2816039 | 3.8757326 |
| C | -2.1799407 | 0.9401257 | 2.1295713 |
| C | -0.9751838 | 0.2209157 | 2.1946524 |
| C | 0.0900781 | 1.1455428 | 2.1282677 |
| H | -0.8906159 | -0.8533089 | 2.2625134 |
| C | -0.4653921 | 2.4309040 | 2.0120936 |
| C | -1.8726608 | 2.3029003 | 1.9971233 |
| H | -2.5878001 | 3.1054253 | 1.8978836 |
| H | -3.1726235 | 0.5200144 | 2.1245540 |
| H | 1.1439160 | 0.9194103 | 2.1636374 |
| H | 0.0899882 | 3.3542474 | 1.9474382 |
| C | -1.8890985 | 2.8788898 | -1.5710247 |
| C | -1.1265081 | 1.9594325 | -2.3290175 |
| C | 0.2227765 | 2.1114160 | -1.9664797 |
| H | -1.5213654 | 1.2489496 | -3.0388429 |
| C | 0.2996377 | 3.0896645 | -0.9620919 |
| C | -1.0091525 | 3.5785015 | -0.7347867 |
| H | -1.2839399 | 4.3480357 | -0.0294524 |
| H | -2.9578141 | 3.0171777 | -1.6237709 |
| H | 1.0478882 | 1.5273186 | -2.3443996 |
| H | 1.1969315 | 3.4291902 | -0.4651238 |
| Ti | -1.0056004 | 1.2365779 | -0.0288360 |
| O | -2.6567824 | 0.0603493 | -0.5298698 |
| S | -4.0865861 | 0.3455691 | -0.9133335 |
| C | -4.8971707 | -1.1963194 | -0.5221073 |
| H | -5.9532390 | -1.1107894 | -0.7705909 |
| H | -4.7892743 | -1.4082503 | 0.5394973 |
| H | -4.4517225 | -2.0016278 | -1.1025782 |
| O | -4.2339725 | 0.5459282 | -2.3569262 |
| O | -4.6986907 | 1.3774044 | -0.0718079 |

# 6. References


[1] S. Estopina-Duran, J. L. Donnelly, B. E. McLean, M. B. Hockin, A. M. Z. Slawin, J. E. Taylor, *Chem. A Eur. J.* **2019** *25*, 3950-3956.

[2] V. Ivasyshyn, H. Smit, R. C. Chiechi, *ACS Omega* **2019**, *4*, 14140-14150.

[3] Y. Qin, L. Zhang, J. Lv, S. Luo, J.-P. Cheng, *Org. Lett.* **2015***, 17*, 1469-1472.

[4] F. C. Pigge, J. J. Coniglio, S. Fang, *Organometallics*, **2002**, *21*, 4505-4512.

[5] S. Hitoshi, S. Kazuki, K. Noboru, M. Tetsuhiro, T. Kaname, S. Hiroyuki, H. Koji, H. Akira, S. Motoki, K. Kaichiro, I. Yoshihide, H. Daisuke, K. Yasushi, K. Mio, K. Masaharu, K. Masahiro, K. Toshiro, T. Hideki, T. Makoto, I. Masanori, Y. Mitsutaka, *Org. Process Res. Dev.* **2005**, *9*, 278–287.

[6] V. Cesar, Y. Zhang, W. Kosmik, A. Zielinski, A.A. Rajkiewicz, M. Ruamps, S. Bastin, N. Lugan, G. Lavignem, K. Grela, *Chem. Eur. J*. **2017**, *23*, 1950-1955.

[7] L. Alcaraz, K. Cox, A. P. Cridland, E. Kinchin, J. Morris, S. P. Thompson, *Org. Lett.* **2005**, *7*, 1399-1401.

[8] P. V. Sri Ramya, *Eur. J. Med. Chem.* **2017**, *127*, 100-114.

[9] J. C. Walters, A. F. Tierno, A. H. Dubin, S. E. Wengryniuk, *Eur. J. Org. Chem.* **2018**, *12*, 1460-1464.

[10] A. B. Skov, S. L. Broman, A. S. Gertsen, J. Elm, M. Jevric, M. Cacciarini, A. Kadziola, K. Mikkelsen, B. Nielsen, *Chem. Eur. J.* **2016**, *22*, 14567-14575.

[11] E. Fernandez-Mateos, B. Macia, D. J. Ramon, M. Yus, *Eur. J. Org.Chem.* **2011**, *34*, 6851-6855.

[12] S. Roesner, D. J. Blair, V. K. Aggarwal, *Chem. Sci*. **2015**, *6*, 3718-3723.

[13] C. Ho Oh, C. Yun Rhim, C. Ho You, J. Rai Cho, *Synth. Commun*. **2003**, *33*, 4297-4302.

[14] M. Karikomi, K. Arai, T. Toda, *Tetrahedron Letters* **1997**, *38*, 6059-6062.

[15] C. Wang, A. Pettman, J. Basca, J. Xiao, *Angew. Chem. Int. Ed.* **2010**, *49*, 7548-7552; *Angew. Chem.* **2010**, *122*, 7710-7714.

[16] E. Salomo, A. Gallen, G. Sciortino, G. Ujaque, A. Grabulosa, A. Lledos, A. Riera, X. Verdaguer, *J. Am. Chem. Soc.* **2018**, *140*, 16967–16970.

[17] V. N. Wakchaure, P. S. J. Kaib, M. Leutzsch, B. List, *Angew. Chem. Int. Ed.* **2015**, *54*, 11852-11856; *Angew. Chem*. **2015**, *127*, 12019-12023.

[18] G. A. Luinstra, *J. Organomet. Chem.* **1996**, *157*, 209-215.

[19] R. B. Richrath, T. Olyschläger, S. Hildebrandt, D. G. Enny, G. Finau, R. A. II Flowers, A. Gansäuer, *Chem. Eur. J*. **2018**, *24*, 6371-6379.
